# Copper-catalyzed oxidative C(sp^3^)–H/C(sp^2^)–H cross-coupling *en route* to carbocyclic rings†
†Electronic supplementary information (ESI) available. See DOI: 10.1039/c7sc00250e



**DOI:** 10.1039/c7sc00250e

**Published:** 2017-03-15

**Authors:** Rui Wang, Yan Li, Ruo-Xing Jin, Xi-Sheng Wang

**Affiliations:** a Department of Chemistry , University of Science and Technology of China , 96 Jinzhai Road , Hefei , Anhui 230026 , China . Email: xswang77@ustc.edu.cn

## Abstract

Copper-catalyzed selective coupling of C(sp^3^)–H bonds with C(sp^2^)–H bonds has been developed. An aniline module was used as a directing group to generate an aminyl radical, which selectively cleaves the secondary and tertiary C(sp^3^)–H bonds *via* a 1,5-HAT process to forge six-membered carbocyclic rings.

## Abstract 2

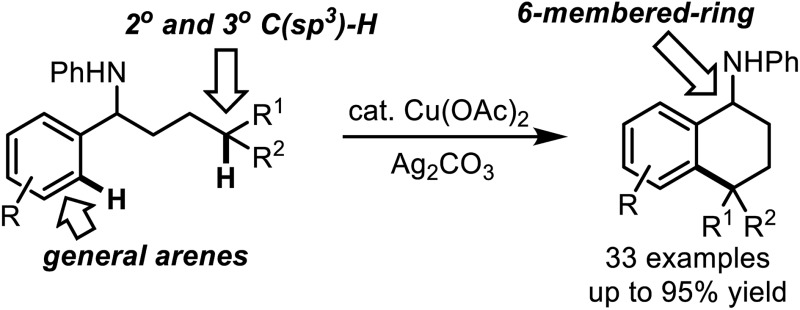
Copper-catalyzed C(sp^3^)–H/C(sp^2^)–H cross-coupling *via* directed 1,5-hydrogen atom transfer has been developed to construct 6-membered carbocyclic rings.

## 

The selective construction of C–C bonds, the essential link in organic molecules, in more efficient ways is always the central topic in synthetic chemistry.^1^ In the past several decades, transition-metal-catalyzed C–C bond construction *via* the activation of ubiquitous C–H bonds has attracted increasing attention, due to the atom and step economy.^2^ While the recent fast developments in this area offered various transformations involving mainly activation of one C–H bond,^3^ C–C bond formation *via* direct C–H/C–H cross-coupling is no doubt the most efficient and ideal method. Inspired by this strategy, cross-dehydrogenative coupling (CDC), termed by Li, was developed and used for the construction of diverse functional molecules.^4^ However, at least one relatively active C–H bond, such as C–H bonds adjacent to heteroatoms and carbonyl groups, or at the benzylic and allylic positions, is typically required in these reactions, and the direct cross-coupling of two inert C–H bonds still faces many challenges from issues such as reactivity and regioselectivity.

Transition-metal-catalyzed oxidative coupling of inert C–H bonds has emerged as a powerful method to construct C–C bonds in an intra- and inter-molecular manner. While the coupling of both aryl C–H bonds has been well documented for biaryl bond formation,^5^ the cross-coupling of inert aromatic and aliphatic C–H bonds selectively still remains an unsolved problem. For instance, Li and co-workers reported an intermolecular CDC of arenes with simple unactivated alkanes in 2008.^6^ While the selective activation of C(sp^2^)–H bonds could be realized using pyridine as the *ortho*-directing group, the regioselective activation of inert C(sp^3^)–H bonds was still an unsurmountable problem in this transformation. Only two examples of intramolecular C(sp^2^)–C(sp^3^) couplings involving Pd(ii)-catalyzed tandem activation of C(sp^2^)–H and C(sp^3^)–H bonds successively have been reported,^7^ in which only relatively active heteroaryl C(sp^2^)–H and primary C(sp^3^)–H bonds were compatible to produce five-membered-ring products accordingly (path A, Scheme 1). Different from the metal-catalyzed C(sp^2^)–H/C(sp^3^)–H oxidative couplings that were initiated from C(sp^2^)–H bond activation, we conceive that the C(sp^3^)–H bond could be cleaved firstly *via* radical hydrogen-atom abstraction,^8,9^ and the alkyl radical is then trapped by aryl rings to produce the final C(sp^2^)–C(sp^3^) coupling product after the following oxidation and deprotonation. While the inert C(sp^3^)–H bonds are almost impossible to distinguish from other aliphatic C–H bonds on the alkyl side chain, the 1,*n*-hydrogen-atom-transfer (1,*n*-HAT)^9^ strategy offers us a reliable solution, in which heteroatoms might be installed on the starting materials to generate the heteroatom radicals for directed selective cleavage of inert C(sp^3^)–H bonds (path B, Scheme 1).^10^

**Scheme 1:**
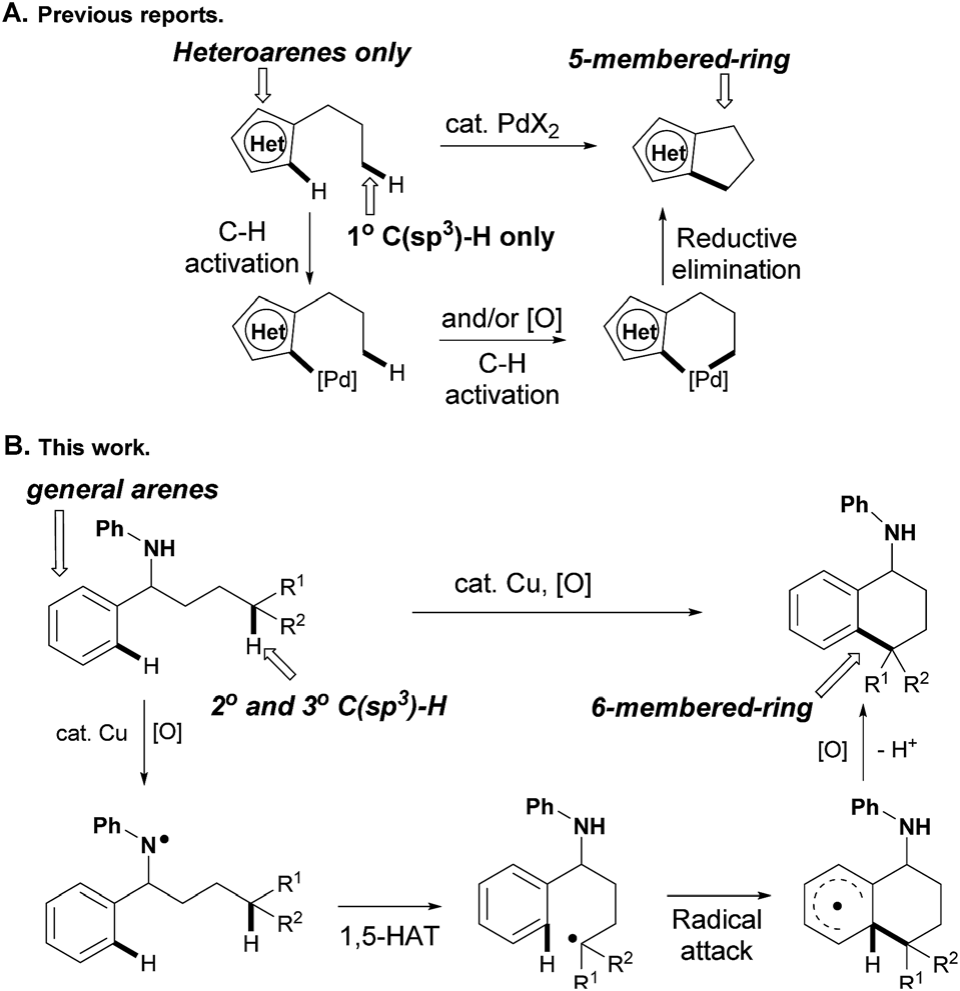
Transition-metal-catalyzed intramolecular C(sp^3^)–H/C(sp^2^)–H cross-coupling.

Herein we report a copper-catalyzed intramolecular cross-coupling of inert aliphatic and aryl C–H bonds with yields as high as 95%. An aniline module was used as a directing group to generate the aminyl radical, which selectively cleaves the secondary and tertiary C(sp^3^)–H bonds *via* a 1,5-HAT process to produce 6-membered-ring products. The initial nitrogen radical could be generated with the assistance of a catalytic amount of Cu(OAc)_2_ directly from an N–H bond in aniline,^11^ and pre-functionalized or *in situ* generated N–X bonds were not required.^8^

Our initial study commenced with **1a** as the pilot substrate in the presence of a catalytic amount of Cu(OAc)_2_. After careful reaction optimization (Tables S1–S4, see ESI†), we found that the combination of **1a** with Cu(OAc)_2_ (20 mol%) and Ag_2_CO_3_ (1.5 equiv.) in 1,2-dichloroethane (DCE, 2.0 mL) at 135 °C for 25 h afforded the desired product **2a** with the best yield (92%, Table 1, entry 1). Control experiments and the effects of each parameter were then examined (Table 1). It is revealed that Cu(OAc)_2_ as the catalyst together with Ag_2_CO_3_ as the oxidant was crucial for this reaction. No product could be observed in the absence of Cu(OAc)_2_ (entry 2), and other copper salts failed to catalyze the reaction. If a stoichiometric amount of Cu(OAc)_2_ (3 equiv.) was used without the addition of Ag_2_CO_3_, only a trace amount of **2a** was detected (entry 5). Variation of the reaction temperature decreased the yield slightly (entries 9–10). When the reaction was quenched at 12 h, the desired product **2a** was isolated in 77% yield (entry 11). Upon reducing the catalyst loading of Cu(OAc)_2_ or the amount of oxidant Ag_2_CO_3_, the yields decreased only slightly, furnishing **2a** in 83% yield even with only 5 mol% of Cu(OAc)_2_ (entry 13) and also in 83% yield with 1 equivalent of Ag_2_CO_3_ (entry 14).

**Table 1:** Control experiments and the effects of each reaction parameter^*a*^

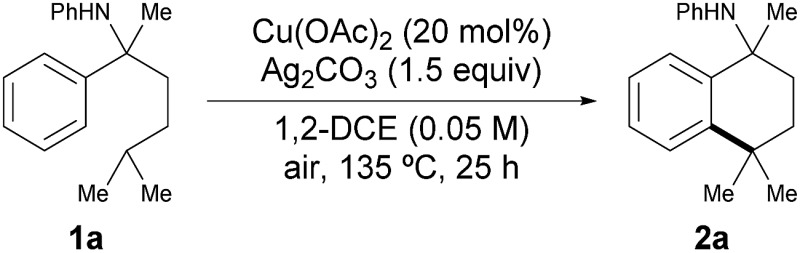		
Entry	Variation from the standard conditions	Yield (%)
1	None	92
2	Without Cu(OAc)_2_	n.d.
3	Cu(OTf)_2_ instead of Cu(OAc)_2_	n.d.
4	Cu(acac)_2_ instead of Cu(OAc)_2_	Trace
5	Cu(OAc)_2_ instead of Ag_2_CO_3_	Trace
6	AgOAc instead of Ag_2_CO_3_	38
7	O_2_ instead of Ag_2_CO_3_	18
8	TEMPO instead of Ag_2_CO_3_	n.d.
9	120 °C instead of 135 °C	60
10	150 °C instead of 135 °C	78
11	12 h instead of 25 h	77
12	10 mol% instead of 20 mol% Cu(OAc)_2_	87
13	5 mol% instead of 20 mol% Cu(OAc)_2_	83
14	100 mol% instead of 150 mol% Ag_2_CO_3_	83

^*a*^Unless otherwise noted, the reaction conditions were as follows: **1a** (0.1 mmol), Cu(OAc)_2_ (20 mol%) and Ag_2_CO_3_ (1.5 equiv.) in 1,2-DCE (2.0 mL) at 135 °C for 25 h. Isolated yields were reported. n.d., not detected.

With the optimized conditions in hand, we next sought to analyze the scope of this protocol (Table 2). With regards to the substituent effect on a phenyl ring, various substituted groups were compatible with this transformation. As for the substituents on the *para*-site of the phenyl ring, both electron-donating and -withdrawing groups were well tolerated with moderate to excellent yields, including alkyl (**2b–d**), phenyl (**2e**), methylthio (**2f**), halogen (**2g–i**) and trifluoromethyl (**2j**). In particular, the presence of halogen atoms (Br, Cl, and F) in products **2** offers the potential for further synthetic elaboration *via* known transition-metal-catalyzed coupling methods. Generally, some anilines, **1**, with different substituents, such as methyl, cyclopropyl, chloro and trifluoromethyl, at the *meta*-site on the phenyl ring could be cyclized completely at the less sterically hindered position with good yields (**2k–n**), which can be explained by the steric effect of such substituents.

**Table 2:** Substrate scope^*a*^

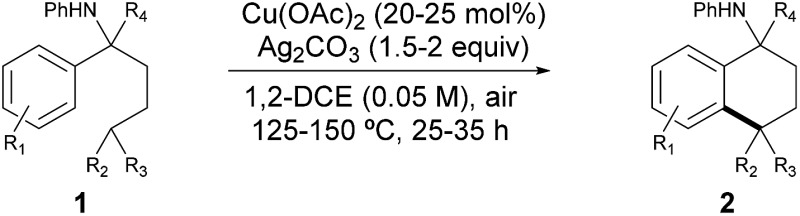
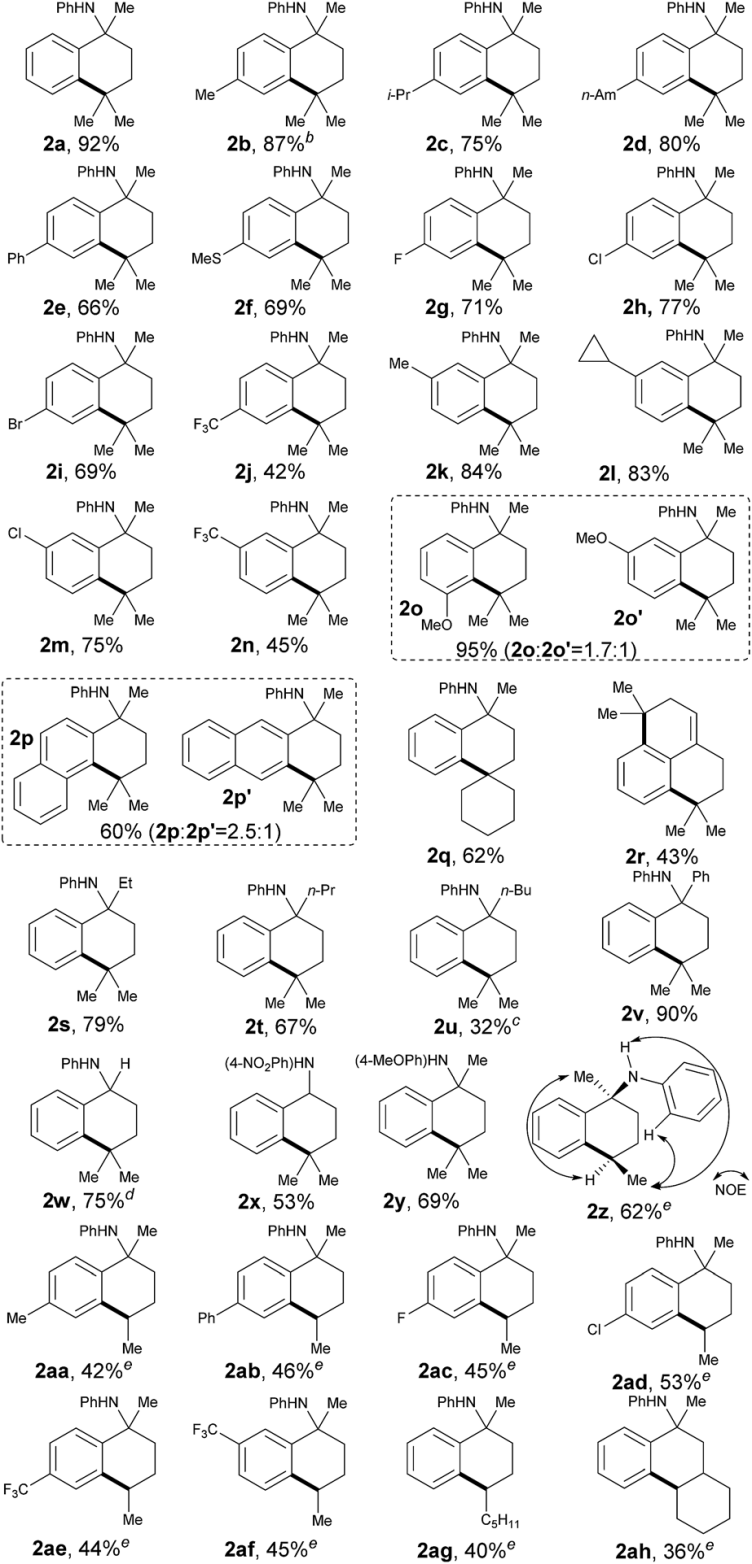

^*a*^Reaction conditions: for **2a–q**, **2s–t**, and **2v–y**, reactions were performed on **1** (0.1 mmol), Cu(OAc)_2_ (20 mol%) and Ag_2_CO_3_ (1.5 equiv.) in 1,2-DCE (2.0 mL) at 135 °C for 25 h; for **2r**, **2u**, and **2z–ah**, reactions were performed on **1** (0.1 mmol), Cu(OAc)_2_ (25 mol%) and Ag_2_CO_3_ (2 equiv.) in 1,2-DCE (2.0 mL) at 150 °C for 35 h. Isolated yields were reported.

^*b*^20 h.

^*c*^135 °C.

^*d*^125 °C.

^*e*^Single diastereoisomer.

Yet intriguingly, the *meta*-MeO-substituted aniline, **1o**, afforded both 5- and 7-methoxyl cyclic products in an excellent combined yield (95%, **2o** and **2o′**), but with a compromised selectivity to give the cyclized product at the more sterically hindered position as the major isomer. Even though this selectivity was not significant (1.7 : 1), it indicated that the electronic effect also plays an important role in this transformation. The cyclization of naphthalen-2-yl-derived aniline, **1p**, also proceeded smoothly to give a mixture of both tetrahydrophenanthren and tetrahydroanthracen-type products with a good total yield (60%, **2p** and **2p′**). Similar to the *meta*-methoxy substrates, aniline **2p** that cyclized at the relatively more sterically hindered position was the major isomer. The selective activation of the C(sp^3^)–H bond in the cycloalkanes was also successful for cross-coupling of the phenyl ring, providing a synthetically difficult spiro skeleton in 62% yield (**2q**). A symmetric aniline with two of the same C(sp^3^)–H bonds suitable for 1,5-hydrogen-atom abstraction in both alkyl side chains was also tested in this catalytic system. It was interesting to find that both C(sp^3^)–H bonds coupled with the *ortho*-C(sp^2^)–H bonds on the phenyl ring smoothly, followed by *in situ* elimination of aniline to afford an attractive 2,3,5,6-tetrahydro-1*H*-phenalene structure (**2r**).

The substituents at the α-position of the nitrogen atom had an obvious effect on the transformation. While the corresponding yields decreased along with the increase of steric hindrance of the alkyl groups (**2s–u**), the cyclization of *N*-α,α-biphenyl aniline proceeded quite smoothly with excellent yield (90%, **2v**). It is important to point out that the hydrogen atom at the α-position of the nitrogen atom, well known to be activated *via* a radical path, could be well tolerated in this reaction (75%, **2w**), which further indicated that the regioselectivity of this method was determined by a 1,5-HAT process. In order to demonstrate the synthetic potential of this method, we tried to synthesize **2x**, an analogue of **UCI-30002**, which has been proven to play an inhibition role on several acetylcholine receptors.^12^ As expected, **2x** could be obtained in 53% yield under our standard conditions. While aniline **1x** could be prepared from readily available compounds like benzonitrile and 4-nitroaniline through a simple and easy-to-handle process (see ESI,† pp. 18), this result definitely showed the feasibility of variation on the directing aniline part. As a common removable protecting group on a nitrogen atom, *para*-MeOPh (PMP) was also well compatible with this method (69%, **2y**).^15^

It is also important to note that the secondary C(sp^3^)–H bonds could also be selectively activated to furnish the corresponding cyclized products as single diastereoisomers in acceptable yields (40–62%, **2z–ag**, for details, see ESI†). A number of substituents were readily compatible with this protocol. Remarkably, the subjection of substrate **1ah**, which possesses a tertiary C(sp^3^)–H bond at the 4-position and a secondary C(sp^3^)–H center at the 5-position to the nitrogen atom, to the standard conditions only produced the 5-position-reacting product **2ah**. This result also clearly showed that the site-selectivity of C(sp^3^)–H bond activation on the alkyl chain was determined by 1,5-HAT solely, which further illustrates the excellent site-selectivity of our methodology.

However, the selective cross-coupling of a primary C(sp^3^)–H bond (**1ai**, see ESI†) to an intramolecular phenyl ring provided only a trace amount of the corresponding product in this reaction. Taking into account all of the above results, the reactivity of aliphatic C–H bonds paralleled the thermodynamic stability of the carbon radicals produced and followed the order: tertiary > secondary > primary. Notably, as for the anilines bearing both tertiary C(sp^3^)–H and secondary or primary C(sp^3^)–H bonds (for primary C–H, see **1s**; for secondary C–H, see **1t**), the coupling reaction happened at the tertiary C–H position exclusively, which showed excellent chemoselectivity of this protocol for inert C(sp^3^)–H bond activation.

A series of control experiments were designed to probe the possible radical pathway. Firstly, some free radical inhibitors, including TEMPO, BHT and galvinoxyl, showed strong inhibition effects even with only a sub-stoichiometric amount (Table S5, see ESI†). It’s worth noting that the addition of 1.5 equivalents of TEMPO to the standard conditions afforded alkene **3a** in 49% yield (Scheme 2A). As alkene **3a** could be produced from the coupling adduct **3a′** *via* elimination,^13^ this result was definitely consistent with our hypothesis about the radical path. To further verify the radical process, vinyl cyclopropane **1aj** and cyclopropyl aniline **1ak** were designed to trap the corresponding nitrogen and carbon radical one by one.^14^ Indeed, the reaction of **1aj** gave the ring-opened products **2aj-1** and **2aj-2** in 14% and 18% yield, respectively (Scheme 2B). Moreover, while the subjection of **1ak** to the standard conditions afforded none of the desired alkene, **2ak′**, tricyclic compound **2ak** was obtained successfully, albeit with low yield (10%, Scheme 2C). Given that **2ak** could be transformed from **2ak′** *via* 5-*exo*-trig cyclization^14^ followed by sequenced radical cyclization and oxidation (Fig. S2, see ESI†), **2ak′** was synthesized and subjected to the reaction system, furnishing **2ak** in 51% yield accordingly, as shown in Fig. S2.† All these results clearly indicated that the corresponding nitrogen radical and carbon radical were involved in the catalytic cycle, thus supporting the selective radical C(sp^3^)–H activation path as in our design. Finally, no reaction occurred and all the starting material remained when tertiary amine **1al** was subjected to the standard conditions (Scheme 2D), which showed the key role of a hydrogen atom on the nitrogen for radical generation.

**Scheme 2:**
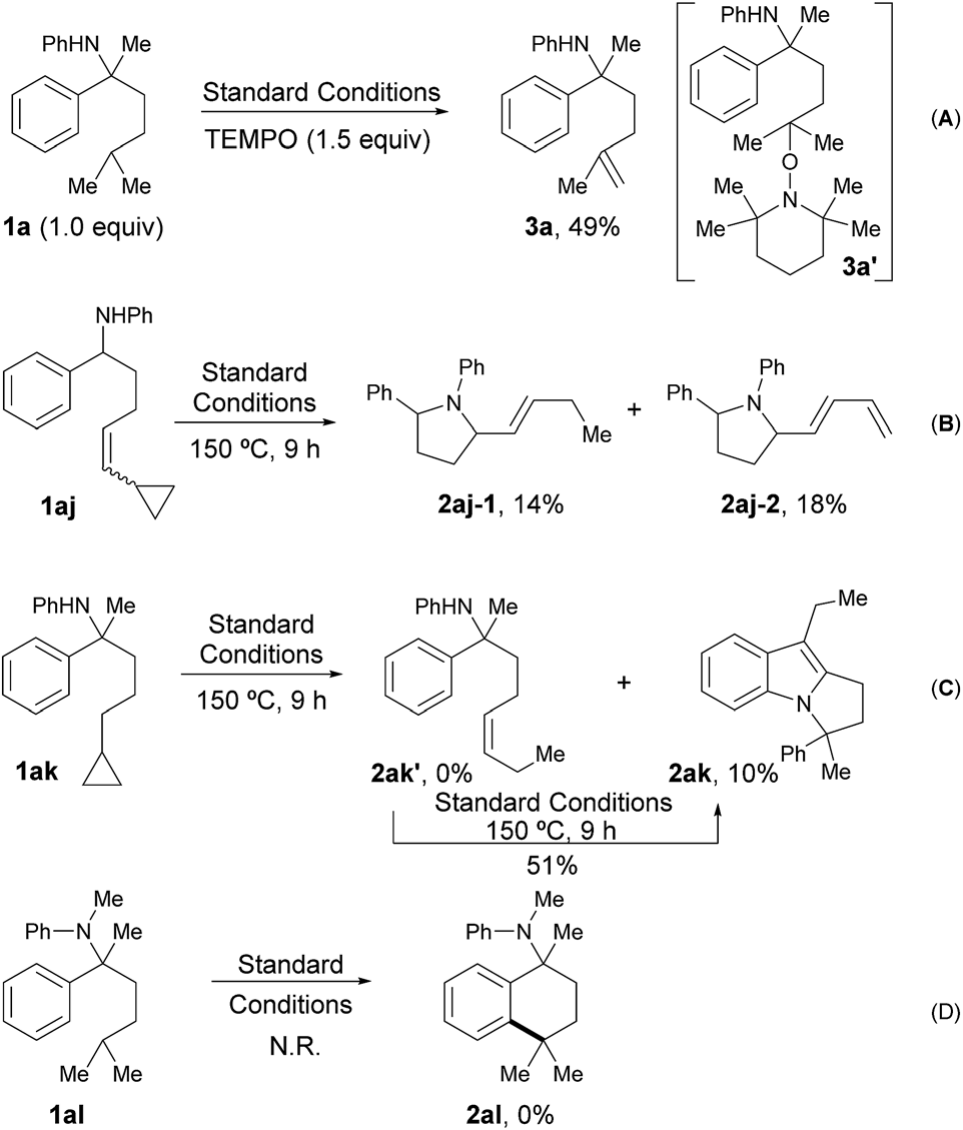
Control experiments designed to probe the radical path.

Based on all of the results above and the previous reports, a plausible mechanism for the amine-directed selective activation of inert C(sp^3^)–H bonds for C(sp^3^)–H/C(sp^2^)–H cross-coupling is outlined in Scheme 3. The initial interaction of Cu(OAc)_2_ with amine **1** generates the nitrogen radical **I**,^11^ which selectively abstracts the hydrogen atom at the remote aliphatic C–H bond *via* a 1,5-hydrogen-atom transfer to afford the carbon center radical **II**. The carbon radical **II** is subsequently captured by a phenyl ring, and the final 6-membered product **2** is afforded after the following oxidation and deprotonation.

**Scheme 3:**
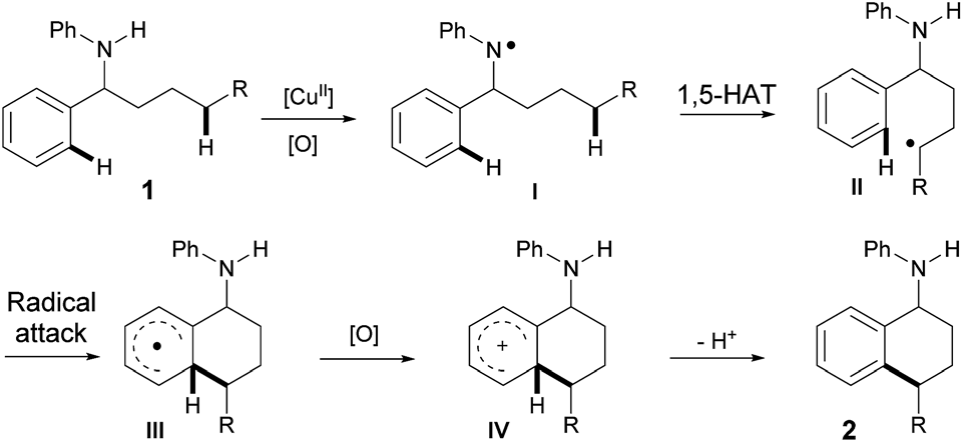
Proposed mechanism.

## Conclusions

In summary, we have developed a copper-catalyzed amine-directed selective activation of C(sp^3^)–H bonds for cross-coupling of inert C(sp^3^)–H and C(sp^2^)–H bonds. The catalytic cycle was initiated from the selective cleavage of inert secondary or tertiary C(sp^3^)–H bonds *via* a 1,5-HAT process, affording the 6-membered-ring products with high efficiency and broad scope. Further efforts to develop more novel transformations with this method are still ongoing in our lab.
