# Supplementary material for: Copper-catalyzed oxidative C(sp3)–H/C(sp2)–H cross-coupling en route to carbocyclic rings
Source: Chem Sci. 2017 Mar 15;8(5):3838–42. doi: 10.1039/c7sc00250e (PMC5577718; doi:10.1039/c7sc00250e)
Supplement: Supplementary file 1 [file SC-008-C7SC00250E-s001.pdf]

Electronic Supplementary Material (ESI) for Chemical Science.
This journal is © The Royal Society of Chemistry 2017

Supplementary Materials for

Copper-catalyzed oxidative $C(sp^3)$-H/$C(sp^2)$-H cross-coupling en route to carbocyclic rings

Rui Wang, Yan Li, Ruo-Xing Jin, and Xi-Sheng Wang*

Department of Chemistry, University of Science and Technology of China, 96 Jinzhai Road, Hefei, Anhui 230026, China

*Correspondence to: xswang77@ustc.edu.cn.

Table of Contents

I. General Information……………………………………………..S2

II. Preparation of Substrates……………………………………S3

III. Copper-catalyzed oxidative C(sp3)-H/ C(sp2)-H cross-coupling en route to carbocyclic rings…………………………………………S27

Reaction Optimization Details…………………………S27

Substrate Screening……………………………………….S29

IV. Mechanism Details……………………………………………….S46

V. References…………………………………………………………….S48

VI. Spectroscopic Data (NMR Spectrum)…………………………..S49

I. General Information: Instrumentation, Materials.

Instrumentation. ^{1}H NMR spectra were recorded at ambient temperature on Bruker-400 (400 MHz) spectrometers and are referenced relative to the residual protons in $CDCl_3$ at δ 7.26 ppm or $(CD_3)_2SO$-d_6 at δ 2.50 ppm. Data for ^{1}H NMR are reported as follows: chemical shift (ppm), multiplicity (s = singlet, d = doublet, t = triplet, q = quartet, m = multiplet, ap = apparent), integration, and coupling constant (Hz). ^{13}C NMR spectra were recorded at ambient temperature on Bruker-400 (100 MHz) spectrometers and are referenced relative to $CDCl_3$ at δ 77.16 ppm or $(CD_3)_2SO$-d_6 at δ 39.25 ppm. The ^{13}C NMR spectra were obtained with 1H decoupling. Data for ^{13}C NMR are reported in terms of chemical shift and multiplicity where appropriate. High resolution mass spectra were recorded on P-SIMS-Gly of BrukerDaltonics Inc. using ESI-TOF (electrospray ionization-time of flight).

Materials. Copper diacetate was purchased from Shanghai DEMO Medical Tech Co., Ltd. and used as received. Silver carbonate was purchased from Adamas Reagent Co., Ltd. and used as recieved. 1,2-dichloroethane was purchased from Sinopharm Chemical Reagent Co., Ltd. Other commercial reagents were purchased from commercial suppliers and used without further purification.

II. Preparation of Substrates.

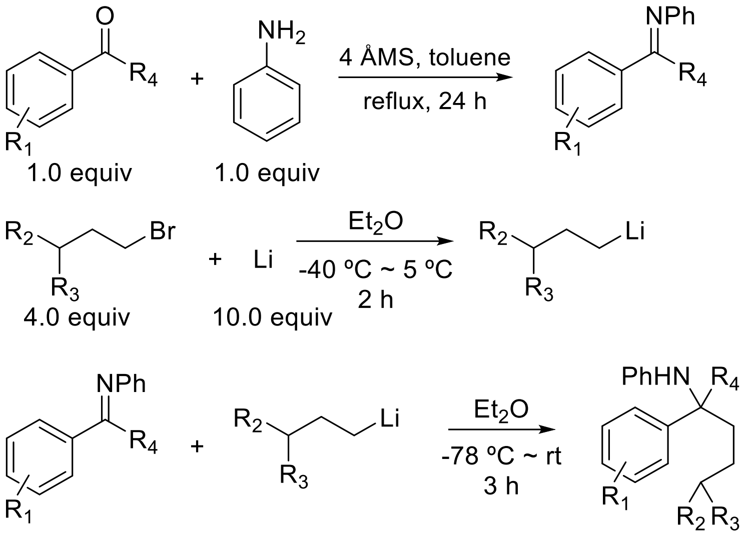

(1)
(2)
(3)
1

Genaral procedure (A) for the synthesis of substrates.

Step 1: Preparation of imine. To an oven-dried round-bottom bottle equipped with a magnetic stir bar was added ketone (1.0 equiv), aniline (1.0 equiv), 4 ÅMS (0.2 g/mmol) and toluene (2.0 M). The mixture was then reflux for 24 h. After completion, it was allowed to cool to room temperature, and was directly filtered through a short pad of Celite®, washed with EtOAc. The filtrate was concentrated under vacuum and was used directly.

Step 2: Preparation of lithium reagent. The bromide (4.0 equiv) in Et_2O (4.0 M) was added dropwise to a vigorously stirred suspension of lithium rods (10.0 equiv) in Et_2O (4.0 M) at -40 °C under N_2 atmosphere. And the mixture was allowed to warm up to 5 °C and stirred for 2 h. The resulting blackish suspension was then used imediately.

Step 3: Preparation of the substates. The freshly prepared lithium reagent was added dropwise to a vigorously stirred solution of imine in Et_2O (1.0 M) at -78 °C under N_2 atmosphere and stirred at room temperature for 3 h (but with **1g**, **1j**, **1n**, the stirring temperature was -40°C). After completion, the reaction was quenched with water. The resulting aqueous layer was extracted with EtOAc for 3 times and the combined organic layer was washed with brine, dried over Na_2SO_4 and concentrated under vacuum. The crude was purified by column chromatography on silica gel to give the substrate **1**.

***N*-(5-methyl-2-phenylhexan-2-yl)aniline (1a)**

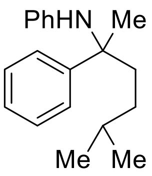

Prepared according to general procedure (A) from acetophenone and 1-bromo-3-methylbutane to provide the title compound **1a** as a colorless oil (1.17 g, 4.4 mmol, 88% total yield). ^{1}H NMR (400 MHz, $CDCl_3$) δ 7.46 (d, *J* = 7.6 Hz, 2H), 7.31 (t, *J* = 7.5 Hz, 2H), 7.21 (t, *J* = 6.9 Hz, 1H),

6.98 (t, J = 7.7 Hz, 2H), 6.58 (t, J = 7.3 Hz, 1H), 6.31 (d, J = 7.9 Hz, 2H), 3.99 (s, 1H), 1.83 (dtd, J = 19.6, 13.2, 8.4 Hz, 2H), 1.62 (s, 3H), 1.51 – 1.32 (m, 1H), 1.09 (dd, J = 15.9, 7.4 Hz, 2H), 0.81 (d, J = 7.0 Hz, 3H), 0.80 (d, J = 7.0 Hz, 3H).; ^{13}C NMR (101 MHz, $CDCl_3$) δ 146.8, 146.2, 128.8, 128.5, 126.3, 126.3, 117.0, 115.4, 58.4, 42.4, 32.7, 28.4, 25.7, 22.8, 22.7. HRMS (ESI) calcd. for $C_{19}H_{26}N$ $[M+H]^+$ *m/z* 268.2065, found 268.2062.

***N*-(5-methyl-2-(p-tolyl)hexan-2-yl)aniline (1b)**

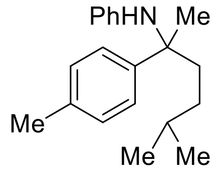

Prepared according to general procedure (A) from 1-(p-tolyl)ethan-1-one and 1-bromo-3-methylbutane to provide the title compound **1b** as a colorless oil (1.26 g, 4.5 mmol, 90% total yield). ^{1}H NMR (400 MHz, $CDCl_3$) δ 7.35 (d, J = 8.2 Hz, 2H), 7.13 (d, J = 8.4 Hz, 2H), 7.04 – 6.95 (m, 2H), 6.60 (t, J = 7.2 Hz, 1H), 6.40 – 6.29 (m, 2H), 4.00 (s, 1H), 2.35 (s, 3H), 1.83 (dddd, J = 36.0, 13.2, 9.3, 7.5 Hz, 2H), 1.62 (s, 3H), 1.51 – 1.35 (m, 1H), 1.11 (dt, J = 9.5, 7.1 Hz, 2H), 0.84 (d, J = 6.7 Hz, 3H), 0.81 (d, J = 6.7 Hz, 3H).; ^{13}C NMR (101 MHz, $CDCl_3$) δ 146.3, 143.8, 135.7, 129.2, 128.7, 126.2, 117.0, 115.4, 58.2, 42.4, 32.7, 28.4, 25.8, 22.8, 22.7, 21.1. HRMS (ESI) calcd. for $C_{20}H_{28}N$ $[M+H]^+$ *m/z* 282.2222, found 282.2222.

***N*-(2-(4-isopropylphenyl)-5-methylhexan-2-yl)aniline (1c)**

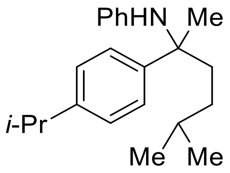

Prepared according to general procedure (A) from 1-(4-isopropylphenyl)ethan-1-one and 1-bromo-3-methylbutane to provide the title compound **1c** as a colorless oil (1.21 g, 3.9 mmol, 78% total yield). ^{1}H NMR (400 MHz, $CDCl_3$) δ 7.39 (d, J = 7.8 Hz, 2H), 7.19 (d, J = 7.8 Hz, 2H), 7.02 (t, J = 7.5 Hz, 2H), 6.62 (t, J = 7.3 Hz, 1H), 6.37 (d, J = 8.0 Hz, 2H), 4.02 (s, 1H), 3.04 – 2.83 (m, 1H), 1.87 (dtd, J = 21.6, 13.0, 6.2 Hz, 2H), 1.64 (s, 3H), 1.54 – 1.37 (m, 1H), 1.28 (d, J = 6.9 Hz, 6H), 1.15 (dd, J = 15.9, 7.7 Hz, 2H), 0.86 (d, J = 7.0 Hz, 3H), 0.83 (d, J = 7.0 Hz, 3H).; ^{13}C NMR (101 MHz, $CDCl_3$) δ 146.7, 146.4, 144.2, 128.8, 126.4, 126.1, 116.9, 115.4, 58.2, 42.1, 33.7, 32.7, 28.4, 26.0, 24.1, 22.8, 22.7. HRMS (ESI) calcd. for $C_{22}H_{32}N$ $[M+H]^+$ *m/z* 310.2535, found 310.2537.

***N*-(5-methyl-2-(4-pentylphenyl)hexan-2-yl)aniline (1d)**

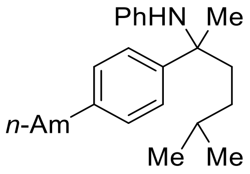

Prepared according to general procedure (A) from 1-(4-pentylphenyl)ethan-1-one and

1-bromo-3-methylbutane to provide the title compound **1d** as a colorless oil (1.08 g, 3.2 mmol, 64% total yield). ^{1}H NMR (400 MHz, $CDCl_3$) δ 7.38 (d, *J* = 7.9 Hz, 2H), 7.15 (d, *J* = 7.8 Hz, 2H), 7.02 (t, *J* = 7.7 Hz, 2H), 6.62 (t, *J* = 7.3 Hz, 1H), 6.36 (d, *J* = 8.0 Hz, 2H), 4.02 (s, 1H), 2.62 (t, *J* = 7.8 Hz, 2H), 2.02 – 1.74 (m, 2H), 1.74 – 1.57 (m, 5H), 1.49 – 1.41 (m, 1H), 1.40 – 1.30 (m, 4H), 1.14 (dd, *J* = 15.8, 7.3 Hz, 2H), 0.92 (t, *J* = 6.6 Hz, 3H), 0.85 (d, *J* = 7.4 Hz, 3H), 0.83 (d, *J* = 7.4 Hz, 3H).; ^{13}C NMR (101 MHz, $CDCl_3$) δ 146.3, 144.0, 140.8, 128.7, 128.4, 126.1, 116.9, 115.4, 58.2, 42.3, 35.6, 32.7, 31.8, 31.2, 28.4, 25.8, 22.8, 22.7, 22.7, 14.2. HRMS (ESI) calcd. for $C_{24}H_{36}N$ $[M+H]^+$ *m/z* 338.2848, found 338.2844.

***N*-(2-([1,1'-biphenyl]-4-yl)-5-methylhexan-2-yl)aniline (1e)**

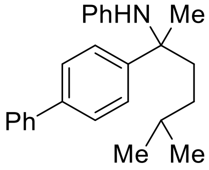

Prepared according to general procedure (A) from 1-([1,1'-biphenyl]-4-yl)ethan-1-one and 1-bromo-3-methylbutane to provide the title compound **1e** as a white solid (1.65 g, 4.8 mmol, 96% total yield). ^{1}H NMR (400 MHz, $CDCl_3$) δ 7.69 – 7.62 (m, 2H), 7.62 – 7.52 (m, 4H), 7.50 – 7.41 (m, 2H), 7.39 – 7.31 (m, 1H), 7.08 – 6.98 (m, 2H), 6.68 – 6.56 (m, 1H), 6.46 – 6.35 (m, 2H), 4.06 (s, 1H), 2.03 – 1.79 (m, 2H), 1.69 (s, 3H), 1.54 – 1.40 (m, 1H), 1.17 (dt, *J* = 9.0, 6.8 Hz, 2H), 0.87 (d, *J* = 6.6 Hz, 3H), 0.84 (d, *J* = 6.6 Hz, 3H).; ^{13}C NMR (101 MHz, $CDCl_3$) δ 146.2, 146.0, 140.9, 139.0, 128.8, 128.8, 127.2, 127.1, 127.1, 126.7, 117.1, 115.4, 58.3, 42.3, 32.7, 28.4, 25.8, 22.8, 22.7. HRMS (ESI) calcd. for $C_{25}H_{30}N$ $[M+H]^+$ *m/z* 344.2378, found 344.2379.

***N*-(5-methyl-2-(4-(methylthio)phenyl)hexan-2-yl)aniline (1f)**

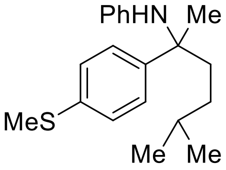

Prepared according to general procedure (A) from 1-(4-(methylthio)phenyl)ethan-1-one and 1-bromo-3-methylbutane to provide the title compound **1f** as a yellow oil (0.80 g, 2.6 mmol, 52% total yield). ^{1}H NMR (400 MHz, $CDCl_3$) δ 7.39 (d, *J* = 8.1 Hz, 2H), 7.21 (d, *J* = 8.1 Hz, 2H), 7.00 (t, *J* = 7.7 Hz, 2H), 6.61 (t, *J* = 7.3 Hz, 1H), 6.33 (d, *J* = 7.9 Hz, 2H), 3.99 (s, 1H), 2.49 (s, 3H), 1.94 – 1.70 (m, 2H), 1.61 (s, 3H), 1.50 – 1.34 (m, 1H), 1.09 (dd, *J* = 16.0, 7.4 Hz, 2H), 0.83 (d, *J* = 7.2 Hz, 3H), 0.81 (d, *J* = 7.2 Hz, 3H).; ^{13}C NMR (101 MHz, $CDCl_3$) δ 146.1, 143.8, 135.9, 128.8, 126.9, 126.6, 117.1, 115.4, 58.2, 42.5, 32.7, 28.4, 25.6, 22.8, 22.7, 15.9. HRMS (ESI) calcd. for $C_{20}H_{28}NS$ $[M+H]^+$ *m/z* 314.1942, found 314.1940.

***N*-(2-(4-fluorophenyl)-5-methylhexan-2-yl)aniline (1g)**

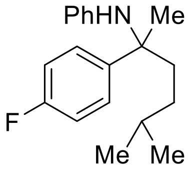

Prepared according to general procedure (A) from 1-(4-fluorophenyl)ethan-1-one and 1-bromo-3-methylbutane to provide the title compound **1g** as a yellow oil (0.97 g, 3.4 mmol, 67% total yield). ^{1}H NMR (400 MHz, $CDCl_3$) δ 7.49 – 7.38 (m, 2H), 7.07 – 6.96 (m, 4H), 6.70 – 6.57 (m, 1H), 6.38 – 6.27 (m, 2H), 4.00 (s, 1H), 1.83 (dddd, *J* = 19.0, 13.2, 9.6, 7.2 Hz, 2H), 1.63 (s, 3H), 1.51 – 1.34 (m, 1H), 1.19 – 1.01 (m, 2H), 0.84 (d, *J* = 6.8 Hz, 3H), 0.82 (d, *J* = 6.8 Hz, 3H).; ^{13}C NMR (101 MHz, $CDCl_3$) δ 161.6 (d, *J* = 245.4 Hz), 146.0, 142.5 (d, *J* = 3.0 Hz), 128.8, 128.0 (d, *J* = 7.1 Hz), 117.3, 115.4, 115.2 (d, *J* = 21.2 Hz), 58.1, 42.6, 32.7, 28.4, 25.7, 22.7, 22.6. HRMS (ESI) calcd. for $C_{19}H_{25}NF$ $[M+H]^+$ *m/z* 286.1971, found 286.1967.

_N_-(2-(4-chlorophenyl)-5-methylhexan-2-yl)aniline (1h)

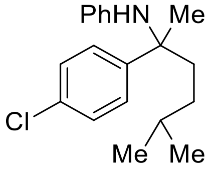

Prepared according to general procedure (A) from 1-(4-chlorophenyl)ethan-1-one and 1-bromo-3-methylbutane to provide the title compound **1h** as a colorless oil (1.14 g, 3.8 mmol, 76% total yield). ^{1}H NMR (400 MHz, $CDCl_3$) δ 7.43 – 7.35 (m, 2H), 7.33 – 7.26 (m, 2H), 7.05 – 6.96 (m, 2H), 6.62 (tt, *J* = 7.3, 1.1 Hz, 1H), 6.34 – 6.26 (m, 2H), 3.98 (s, 1H), 1.91 – 1.69 (m, 2H), 1.61 (s, 3H), 1.49 – 1.35 (m, 1H), 1.07 (ddd, *J* = 10.9, 8.5, 6.4 Hz, 2H), 0.82 (d, *J* = 6.6 Hz, 3H), 0.80 (d, *J* = 6.6 Hz, 3H).; ^{13}C NMR (101 MHz, $CDCl_3$) δ 145.9, 145.4, 132.1, 128.9, 128.6, 127.9, 117.3, 115.4, 58.1, 42.5, 32.6, 28.4, 25.6, 22.7, 22.6. HRMS (ESI) calcd. for $C_{19}H_{25}NCl$ $[M+H]^+$ *m/z* 302.1676, found 302.1677.

_N_-(5-methyl-2-(4-(trifluoromethyl)phenyl)hexan-2-yl)aniline (1j)

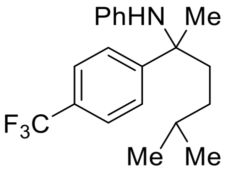

Prepared according to general procedure (A) from 1-(4-(trifluoromethyl)phenyl)ethan-1-one and 1-bromo-3-methylbutane to provide the title compound **1j** as a yellow oil (1.17 g, 3.5 mmol, 70% total yield). ^{1}H NMR (400 MHz, $CDCl_3$) δ 7.67 – 7.55 (m, 4H), 7.03 (t, *J* = 7.8 Hz, 2H), 6.65 (t, *J* = 7.3 Hz, 1H), 6.31 (d, *J* = 7.9 Hz, 2H), 4.04 (s, 1H), 1.87 (dtd, *J* = 17.0, 13.2, 8.3 Hz, 2H), 1.67 (s, 3H), 1.50 – 1.40 (m, 1H), 1.11 (dd, *J* = 16.2, 7.2 Hz, 2H), 0.85 (d, *J* = 6.7 Hz, 3H), 0.83 (d, *J* = 6.7 Hz, 3H).; ^{13}C NMR (101 MHz, $CDCl_3$) δ 151.2, 145.7, 128.9, 128.7 (q, *J* = 32.3 Hz), 126.7, 125.5 (q, *J* = 4.0 Hz), 124.5 (q, *J* = 273.7 Hz), 117.5, 115.4, 58.5, 42.3, 32.6, 28.4, 25.7, 22.7, 22.6. HRMS (ESI) calcd. for $C_{20}H_{25}NF_3$ $[M+H]^+$ *m/z* 336.1939, found 336.1938.

N-(5-methyl-2-(m-tolyl)hexan-2-yl)aniline (1k)

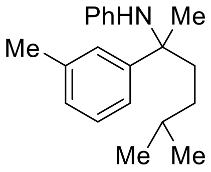

Prepared according to general procedure (A) from 1-(m-tolyl)ethan-1-one and 1-bromo-3-methylbutane to provide the title compound **1k** as a colorless oil (1.12 g, 4.0 mmol, 80% total yield). ^{1}H NMR (400 MHz, $CDCl_3$) δ 7.31 – 7.26 (m, 2H), 7.25 – 7.18 (m, 1H), 7.09 – 6.95 (m, 3H), 6.65 – 6.55 (m, 1H), 6.38 – 6.29 (m, 2H), 3.99 (s, 1H), 2.36 (s, 3H), 1.93 – 1.76 (m, 2H), 1.62 (s, 3H), 1.44 (dt, *J* = 13.3, 6.6 Hz, 1H), 1.12 (dt, *J* = 9.4, 7.1 Hz, 2H), 0.84 (d, J = 6.6 Hz, 3H), 0.82 (d, J = 6.6 Hz, 3H).; ^{13}C NMR (101 MHz, $CDCl_3$) δ 147.0, 146.3, 137.9, 128.7, 128.3, 127.1, 126.9, 123.4, 117.0, 115.5, 58.3, 42.2, 32.7, 28.4, 25.8, 22.8, 22.7, 21.9. HRMS (ESI) calcd. for $C_{20}H_{28}N$ $[M+H]^+$ *m/z* 282.2222, found 282.2221.

N-(2-(3-chlorophenyl)-5-methylhexan-2-yl)aniline (1m)

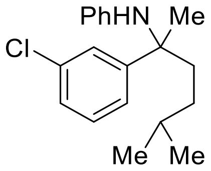

Prepared according to general procedure (A) from 1-(3-chlorophenyl)ethan-1-one and 1-bromo-3-methylbutane to provide the title compound **1m** as a colorless oil (0.81 g, 2.7 mmol, 54% total yield). ^{1}H NMR (400 MHz, $CDCl_3$) δ 7.45 (t, *J* = 1.9 Hz, 1H), 7.36 (dt, *J* = 7.5, 1.6 Hz, 1H), 7.23 (d, *J* = 7.3 Hz, 1H), 7.20 (dt, *J* = 8.0, 1.7 Hz, 1H), 7.00 (dd, *J* = 8.4, 7.0 Hz, 2H), 6.62 (t, *J* = 7.3 Hz, 1H), 6.30 (d, *J* = 8.0 Hz, 2H), 3.97 (s, 1H), 1.81 (dtd, *J* = 19.5, 13.3, 8.5 Hz, 2H), 1.60 (s, 3H), 1.49 – 1.35 (m, 1H), 1.08 (dd, *J* = 15.9, 7.5 Hz, 2H), 0.82 (d, *J* = 6.7 Hz, 3H), 0.80 (d, *J* = 6.7 Hz, 3H).; ^{13}C NMR (101 MHz, $CDCl_3$) δ 149.4, 145.8, 134.5, 129.8, 128.9, 126.6, 126.5, 124.6, 117.4, 115.5, 58.3, 42.2, 32.6, 28.4, 25.6, 22.7, 22.6. HRMS (ESI) calcd. for $C_{19}H_{25}NCl$ $[M+H]^+$ *m/z* 302.1676, found 302.1672.

N-(5-methyl-2-(3-(trifluoromethyl)phenyl)hexan-2-yl)aniline (1n)

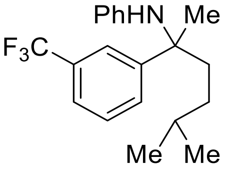

Prepared according to general procedure (A) from 1-(3-(trifluoromethyl)phenyl)ethan-1-one and 1-bromo-3-methylbutane to provide the title compound **1n** as a yellow oil (0.70 g, 2.1 mmol, 42% total yield). ^{1}H NMR (400 MHz, $CDCl_3$) δ 7.79 (dd, *J* = 1.8, 0.9 Hz, 1H), 7.75 (dt, *J* = 7.8, 1.5 Hz, 1H), 7.61 – 7.54 (m, 1H), 7.48 (t, *J* = 7.8 Hz, 1H), 7.11 – 7.02 (m, 2H), 6.69 (tq, *J* = 7.4, 1.1 Hz, 1H), 6.41 – 6.32 (m, 2H), 4.07 (s, 1H), 1.92 (dddd, *J* = 20.6, 16.8, 13.3, 8.3 Hz, 2H), 1.71 (s, 3H), 1.55 – 1.45 (m, 1H), 1.17 (dd, *J* = 16.2, 7.3 Hz, 2H), 0.89 (d, *J* = 6.6 Hz, 3H), 0.87 (d, *J* = 6.6 Hz, 3H).; ^{13}C NMR (101 MHz, $CDCl_3$) δ 148.3, 145.7, 130.8 (q, *J* = 32.3 Hz),

130.0, 129.0, 128.9, 124.5 (q, *J* = 272.7 Hz), 123.4 (q, *J* = 4.0 Hz), 122.9 (q, *J* = 4.0 Hz), 117.6, 115.5, 58.4, 42.1, 32.6, 28.3, 25.8, 22.7, 22.6. HRMS (ESI) calcd. for $C_{20}H_{25}NF_3$ $[M+H]^+$ *m/z* 336.1939, found 336.1935.

***N*-(2-(3-methoxyphenyl)-5-methylhexan-2-yl)aniline (1o)**

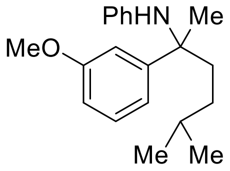

Prepared according to general procedure (A) from 1-(3-methoxyphenyl)ethan-1-one and 1-bromo-3-methylbutane to provide the title compound **1o** as a colorless oil (1.40 g, 4.7 mmol, 94% total yield). ^{1}H NMR (400 MHz, $CDCl_3$) δ 7.25 (t, *J* = 7.9 Hz, 1H), 7.09 – 7.03 (m, 2H), 7.03 – 6.96 (m, 2H), 6.78 (dd, *J* = 8.1, 2.3 Hz, 1H), 6.64 – 6.55 (m, 1H), 6.34 (dd, *J* = 8.5, 1.1 Hz, 2H), 3.98 (s, 1H), 3.79 (s, 3H), 1.94 – 1.72 (m, 2H), 1.61 (s, 3H), 1.48 – 1.38 (m, 1H), 1.10 (dt, *J* = 8.6, 7.4 Hz, 2H), 0.83 (d, *J* = 7.0 Hz, 3H), 0.81 (d, *J* = 7.0 Hz, 3H).; ^{13}C NMR (101 MHz, $CDCl_3$) δ 159.8, 148.8, 146.2, 129.4, 128.7, 118.8, 117.1, 115.4, 112.6, 111.1, 58.4, 55.3, 42.3, 32.7, 28.4, 25.6, 22.7, 22.6. HRMS (ESI) calcd. for $C_{20}H_{28}NO$ $[M+H]^+$ *m/z* 298.2171, found 298.2167.

***N*-(5-methyl-2-(naphthalen-2-yl)hexan-2-yl)aniline (1p)**

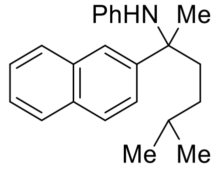

Prepared according to general procedure (A) from 1-(naphthalen-2-yl)ethan-1-one and 1-bromo-3-methylbutane to provide the title compound **1p** as a yellow solid (0.41 g, 1.3 mmol, 26% total yield). ^{1}H NMR (400 MHz, $CDCl_3$) δ 7.95 – 7.79 (m, 4H), 7.72 (dd, *J* = 8.6, 1.9 Hz, 1H), 7.55 – 7.46 (m, 2H), 7.04 – 6.94 (m, 2H), 6.62 (tt, *J* = 7.2, 1.1 Hz, 1H), 6.43 – 6.36 (m, 2H), 4.11 (s, 1H), 2.04 – 1.90 (m, 2H), 1.77 (s, 3H), 1.52 – 1.42 (m, 1H), 1.26 – 1.06 (m, 2H), 0.85 (d, *J* = 6.6 Hz, 3H), 0.84 (d, *J* = 6.6 Hz, 3H).; ^{13}C NMR (101 MHz, $CDCl_3$) δ 146.3, 144.7, 133.6, 132.4, 128.8, 128.3, 128.3, 127.6, 125.9, 125.6, 125.2, 124.6, 117.2, 115.5, 58.6, 42.4, 32.8, 28.4, 25.5, 22.7, 22.7. HRMS (ESI) calcd. for $C_{23}H_{28}N$ $[M+H]^+$ *m/z* 318.2222, found 318.2223.

***N*-(4-cyclohexyl-2-phenylbutan-2-yl)aniline (1q)**

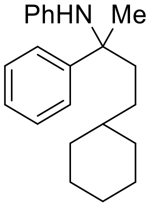

Prepared according to general procedure (A) from acetophenone and (2-bromoethyl)cyclohexane to provide the title compound **1q** as a colorless oil (1.07 g, 3.5

mmol, 70% total yield). ^{1}H NMR (400 MHz, $CDCl_3$) δ 7.51 – 7.45 (m, 2H), 7.33 (t, *J* = 7.6 Hz, 2H), 7.24 (t, *J* = 7.3 Hz, 1H), 7.00 (t, *J* = 7.7 Hz, 2H), 6.61 (t, *J* = 7.3 Hz, 1H), 6.33 (d, *J* = 8.0 Hz, 2H), 4.02 (s, 1H), 1.99 – 1.77 (m, 2H), 1.74 – 1.57 (m, 8H), 1.25 – 1.02 (m, 6H), 0.94 – 0.69 (m, 2H).; ^{13}C NMR (101 MHz, $CDCl_3$) δ 146.8, 146.1, 128.7, 128.4, 126.3, 126.2, 117.0, 115.3, 58.3, 41.8, 38.0, 33.4, 33.3, 31.2, 26.7, 26.4, 26.4, 25.6. HRMS (ESI) calcd. for $C_{22}H_{30}N$ $[M+H]^+$ *m/z* 308.2378, found 308.2375.

N-(6-methyl-3-phenylheptan-3-yl)aniline (1s)

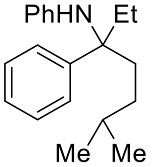

Prepared according to general procedure (A) from propiophenone and 1-bromo-3-methylbutane to provide the title compound **1s** as a colorless oil (1.35 g, 4.8 mmol, 96% total yield). ^{1}H NMR (400 MHz, $CDCl_3$) δ 7.52 – 7.44 (m, 2H), 7.38 – 7.29 (m, 2H), 7.25 – 7.19 (m, 1H), 7.02 – 6.92 (m, 2H), 6.58 (tt, *J* = 7.4, 1.1 Hz, 1H), 6.35 – 6.25 (m, 2H), 3.96 (s, 1H), 2.18 – 1.74 (m, 4H), 1.48 – 1.32 (m, 1H), 1.12 – 0.92 (m, 2H), 0.85 – 0.70 (m, 9H).; ^{13}C NMR (101 MHz, $CDCl_3$) δ 146.1, 146.1, 128.8, 128.3, 126.8, 126.3, 116.9, 115.2, 60.9, 35.1, 32.2, 29.7, 28.3, 22.8, 22.6, 7.8. HRMS (ESI) calcd. for $C_{20}H_{28}N$ $[M+H]^+$ *m/z* 282.2222, found 282.2218.

N-(7-methyl-4-phenyloctan-4-yl)aniline (1t)

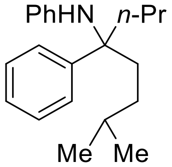

Prepared according to general procedure (A) from 1-phenylbutan-1-one and 1-bromo-3-methylbutane to provide the title compound **1t** as a colorless oil (0.90 g, 3.1 mmol, 62% total yield). ^{1}H NMR (400 MHz, $CDCl_3$) δ 7.51 – 7.44 (m, 2H), 7.36 – 7.29 (m, 2H), 7.25 – 7.19 (m, 1H), 7.01 – 6.92 (m, 2H), 6.61 – 6.55 (m, 1H), 6.33 – 6.25 (m, 2H), 3.95 (s, 1H), 2.10 – 1.91 (m, 2H), 1.92 – 1.71 (m, 2H), 1.44 – 1.35 (m, 1H), 1.24 – 1.10 (m, 2H), 1.10 – 0.99 (m, 2H), 0.88 – 0.71 (m, 9H).; ^{13}C NMR (101 MHz, $CDCl_3$) δ 146.3, 146.2, 128.8, 128.3, 126.7, 126.3, 116.9, 115.2, 60.8, 40.5, 35.4, 32.2, 28.4, 22.8, 22.6, 16.6, 14.6. HRMS (ESI) calcd. for $C_{21}H_{30}N$ $[M+H]^+$ *m/z* 296.2378, found 296.2380.

N-(2-methyl-5-phenylnonan-5-yl)aniline (1u)

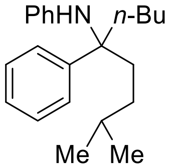

Prepared according to general procedure (A) from 1-phenylpentan-1-one and

1-bromo-3-methylbutane to provide the title compound **1u** as a white solid (1.21 g, 3.9 mmol, 78% total yield). ^{1}H NMR (400 MHz, $CDCl_3$) δ 7.52 – 7.44 (m, 2H), 7.33 (t, *J* = 7.6 Hz, 2H), 7.23 (t, *J* = 7.3 Hz, 1H), 6.97 (t, *J* = 7.8 Hz, 2H), 6.58 (t, *J* = 7.3 Hz, 1H), 6.30 (d, *J* = 8.0 Hz, 2H), 3.96 (s, 1H), 2.12 – 1.94 (m, 2H), 1.93 – 1.78 (m, 2H), 1.45 – 1.34 (m, 1H), 1.31 – 1.18 (m, 2H), 1.18 – 1.08 (m, 2H), 1.09 – 1.00 (m, 2H), 0.88 – 0.70 (m, 9H).; ^{13}C NMR (101 MHz, $CDCl_3$) δ 146.3, 146.2, 128.7, 128.3, 126.8, 126.29, 116.9, 115.2, 60.7, 37.7, 35.4, 32.2, 28.4, 25.5, 23.1, 22.8, 22.6, 14.2. HRMS (ESI) calcd. for $C_{22}H_{32}N$ $[M+H]^+$ *m/z* 310.2535, found 310.2535.

N-(4-methyl-1,1-diphenylpentyl)aniline (1v)

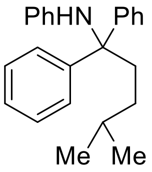

Prepared according to general procedure (A) from benzophenone and 1-bromo-3-methylbutane to provide the title compound **1v** as a white solid (0.63 g, 1.9 mmol, 38% total yield). ^{1}H NMR (400 MHz, $CDCl_3$) δ 7.59 – 7.51 (m, 4H), 7.36 – 7.27 (m, 4H), 7.23 – 7.16 (m, 2H), 7.05 – 6.95 (m, 2H), 6.64 (tt, *J* = 7.3, 1.1 Hz, 1H), 6.45 – 6.36 (m, 2H), 4.53 (s, 1H), 2.62 – 2.47 (m, 2H), 1.51 – 1.43 (m, 1H), 1.13 – 1.00 (m, 2H), 0.78 (d, *J* = 6.6 Hz, 6H); ^{13}C NMR (101 MHz,) δ 146.2, 146.1, 128.7, 128.5, 126.8, 126.5, 117.8, 116.2, 65.2, 35.4, 32.7, 28.4, 22.7. HRMS (ESI) calcd. for $C_{24}H_{28}N$ $[M+H]^+$ *m/z* 330.2222, found 330.2218.

4-methoxy-N-(5-methyl-2-phenylhexan-2-yl)aniline (1y)

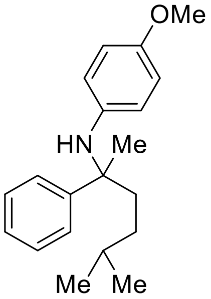

Prepared according to general procedure (A) from acetophenone, aniline and 1-bromo-3-methylbutane to provide the title compound **1y** as a colorless oil (1.31 g, 4.4 mmol, 88% total yield). ^{1}H NMR (400 MHz, $CDCl_3$) δ 7.49 (dd, *J* = 8.3, 1.1 Hz, 2H), 7.34 (t, *J* = 7.6 Hz, 2H), 7.26 – 7.20 (m, 1H), 6.71 – 6.56 (m, 2H), 6.30 (d, *J* = 8.9 Hz, 2H), 3.73 (s, 1H), 3.68 (s, 3H), 2.00 – 1.71 (m, 2H), 1.59 (s, 3H), 1.49 – 1.36 (m, 1H), 1.19 – 1.02 (m, 2H), 0.83 (d, *J* = 6.8 Hz, 3H), 0.81 (d, *J* = 6.8 Hz, 3H).; ^{13}C NMR (101 MHz, $CDCl_3$) δ 152.0, 147.2, 140.2, 128.4, 126.4, 126.3, 117.0, 114.4, 58.6, 55.7, 42.3, 32.8, 28.4, 25.7, 22.8, 22.7. HRMS (ESI) calcd. for $C_{20}H_{28}N$ $[M+H]^+$ *m/z* 298.2171, found 298.2168.

N-(2-phenyldecan-2-yl)aniline (1ag)

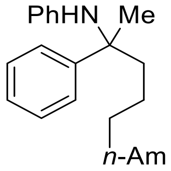

Prepared according to general procedure (A) from acetophenone and 1-bromooctane to provide the title compound **1ag** as a colorless oil (1.08 g, 3.5 mmol, 70% total yield). ^{1}H NMR (400 MHz, $CDCl_3$) δ 7.46 (d, *J* = 7.8 Hz, 2H), 7.31 (t, *J* = 7.6 Hz, 2H), 7.25 – 7.18 (m, 1H), 6.98 (t, *J* = 7.6 Hz, 2H), 6.58 (t, *J* = 7.3 Hz, 1H), 6.31 (d, *J* = 8.0 Hz, 2H), 4.01 (s, 1H), 1.95 – 1.72 (m, 2H), 1.63 (s, 3H), 1.34 – 1.12 (m, 12H), 0.86 (t, *J* = 6.8 Hz, 3H).; ^{13}C NMR (101 MHz, $CDCl_3$) δ 146.8, 146.2, 128.8, 128.5, 126.3, 126.3, 117.0, 115.4, 58.4, 44.7, 32.0, 30.1, 29.6, 29.4, 25.7, 23.8, 22.8, 14.3. HRMS (ESI) calcd. for $C_{22}H_{32}N$ $[M+H]^+$ *m/z* 310.2535, found 310.2530.

***N*-(1-cyclohexyl-2-phenylpropan-2-yl)aniline (1ah)**

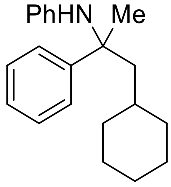

Prepared according to general procedure (A) from acetophenone and (bromomethyl)cyclohexane to provide the title compound **1ah** as a colorless oil (1.35 g, 4.6 mmol, 92% total yield). ^{1}H NMR (400 MHz, $CDCl_3$) δ 7.54 – 7.45 (m, 2H), 7.37 – 7.29 (m, 2H), 7.26 – 7.21 (m, 1H), 7.05 – 6.96 (m, 2H), 6.66 – 6.57 (m, 1H), 6.38 – 6.29 (m, 2H), 4.05 (s, 1H), 1.75 (dd, *J* = 7.9, 4.8 Hz, 2H), 1.70 (s, 3H), 1.64 – 1.51 (m, 3H), 1.50 – 1.31 (m, 3H), 1.24 – 1.01 (m, 3H), 0.98 – 0.82 (m, 2H).; ^{13}C NMR (101 MHz, $CDCl_3$) δ 146.7, 146.2, 128.8, 128.4, 126.4, 126.4, 117.0, 115.5, 59.0, 53.1, 35.6, 35.4, 33.5, 26.6, 26.5, 26.3, 25.5. HRMS (ESI) calcd. for $C_{21}H_{28}N$ $[M+H]^+$ *m/z* 294.2222, found 294.2222.

***N*-(2-phenylpentan-2-yl)aniline (1ai)**

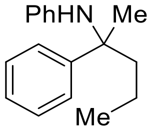

Prepared according to general procedure (A) from acetophenone and 1-bromopropane to provide the title compound **1ai** as a colorless oil (0.76 g, 3.2 mmol, 64% total yield). ^{1}H NMR (400 MHz, $CDCl_3$) δ 7.49 – 7.43 (m, 2H), 7.37 – 7.27 (m, 2H), 7.24 – 7.18 (m, 1H), 7.04 – 6.93 (m, 2H), 6.59 (tq, *J* = 7.4, 1.0 Hz, 1H), 6.31 (dq, *J* = 7.7, 0.9 Hz, 2H), 4.01 (s, 1H), 2.00 – 1.69 (m, 2H), 1.64 (s, 3H), 1.37 – 1.15 (m, 2H), 0.84 (t, *J* = 7.3 Hz, 3H).; ^{13}C NMR (101 MHz, $CDCl_3$) δ 146.7, 146.2, 128.8, 128.5, 126.4, 126.3, 117.0, 115.3, 58.5, 47.1, 25.8, 17.2, 14.6. HRMS (ESI) calcd. for $C_{17}H_{22}N$ $[M+H]^+$ *m/z* 240.1752, found 240.1748.

***N*-(6-methyl-2-phenylheptan-2-yl)aniline (1am)**

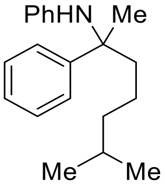

Prepared according to general procedure (A) from acetophenone and 1-bromo-4-methylpentane to provide the title compound **1am** as a colorless oil (1.29 g, 4.6 mmol, 92% total yield). ^{1}H NMR (400 MHz, $CDCl_3$) δ 7.51 (dd, *J* = 8.3, 1.1 Hz, 2H), 7.35 (t, *J* = 7.6 Hz, 2H), 7.26 (tt, *J* = 6.5, 1.1 Hz, 1H), 7.09 – 6.97 (m, 2H), 6.63 (t, *J* = 7.3 Hz, 1H), 6.40 – 6.29 (m, 2H), 4.05 (s, 1H), 1.99 – 1.75 (m, 2H), 1.68 (s, 3H), 1.50 (dp, *J* = 13.3, 6.7 Hz, 1H), 1.26 (dddt, *J* = 13.5, 11.7, 5.6, 2.8 Hz, 2H), 1.17 – 1.06 (m, 2H), 0.83 (dd, *J* = 6.6, 3.2 Hz, 6H).; ^{13}C NMR (101 MHz, $CDCl_3$) δ 146.8, 146.2, 128.8, 128.5, 126.3, 126.3, 117.0, 115.4, 58.5, 44.8, 39.3, 27.8, 25.7, 22.7, 22.6, 21.5. HRMS (ESI) calcd. for $C_{17}H_{22}N$ $[M+H]^+$ *m/z* 282.2222, found 282.2221.

Genaral procedure (B) for the synthesis of the substrates.

Step 1: Preparation of imine. The imine was prepared according to *Step 1* of general procedure (A).

Step 2: Preparation of the substates. Butyllithium (2.5 M in hexane, 4.0 equiv) was added dropwise to a vigorously stirred solution of imine in Et_2O (1.0 M) at -78 °C under N_2 atmosphere and stirred at room temperature for 3 h (but with **1ac**, **1ae**, **1af**, the stirring temperature was -40°C). After completion, the reaction was quenched with water. The resulting aqueous layer was extracted with EtOAc for 3 times and the combined organic layer was washed with brine, dried over Na_2SO_4 and concentrated under vacuum. The crude was purified by column chromatography on silica gel to give the substrate **1**.

***N*-(2-phenylhexan-2-yl)aniline (1z)**

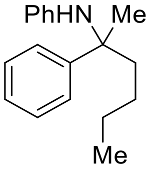

Prepared according to general procedure (B) from acetophenone to provide the title compound **1z** as a colorless oil (1.16 g, 4.6 mmol, 92% total yield). ^{1}H NMR (400 MHz, $CDCl_3$) δ 7.58 – 7.48 (m, 2H), 7.37 (dd, *J* = 10.7, 4.6 Hz, 2H), 7.33 – 7.27 (m, 1H), 7.10 – 7.00 (m, 2H), 6.65 (t, *J* = 7.3 Hz, 1H), 6.37 (dd, *J* = 8.6, 1.2 Hz, 2H), 4.07 (s, 1H), 2.01 – 1.77 (m, 2H), 1.70 (s, 3H), 1.39 – 1.15 (m, 4H), 0.89 (t, *J* = 6.9 Hz, 3H).; ^{13}C NMR (101 MHz, $CDCl_3$) δ 146.8, 146.2, 128.8, 128.5, 126.3, 126.3, 117.0, 115.4, 58.4, 44.6, 26.0, 25.7, 23.1, 14.2. HRMS (ESI) calcd. for $C_{18}H_{24}N$ $[M+H]^+$ *m/z* 254.1909, found 254.1904.

***N*-(2-(p-tolyl)hexan-2-yl)aniline (1aa)**

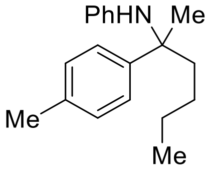

Prepared according to general procedure (B) from 1-(p-tolyl)ethan-1-one to provide the title compound **1aa** as a colorless oil (1.01 g, 3.8 mmol, 76% total yield). ^{1}H NMR (400 MHz, $CDCl_3$) δ 7.35 (d, *J* = 8.2 Hz, 2H), 7.13 (d, *J* = 8.0 Hz, 2H), 7.03 – 6.95 (m, 2H), 6.59 (td, *J* = 7.4, 1.0 Hz, 1H), 6.34 (dd, *J* = 8.6, 1.0 Hz, 2H), 4.00 (s, 1H), 2.34 (s, 3H), 1.83 (dtd, *J* = 19.8, 13.3, 7.8 Hz, 2H), 1.62 (s, 3H), 1.34 – 1.13 (m, 4H), 0.84 (t, *J* = 7.0 Hz, 3H).; ^{13}C NMR (101 MHz, $CDCl_3$) δ 146.3, 143.8, 135.8, 129.2, 128.8, 126.2, 116.9, 115.4, 58.2, 44.6, 26.0, 25.8, 23.2, 21.1, 14.2. HRMS (ESI) calcd. for $C_{19}H_{26}N$ $[M+H]^+$ *m/z* 268.2065, found 268.2063.

_N_-(2-([1,1'-biphenyl]-4-yl)hexan-2-yl)aniline (1ab)

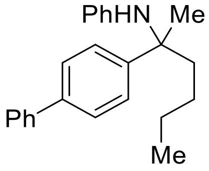

Prepared according to general procedure (B) from 1-([1,1'-biphenyl]-4-yl)ethan-1-one to provide the title compound **1ab** as a white solid (1.02 g, 3.1 mmol, 62% total yield). ^{1}H NMR (400 MHz, $CDCl_3$) δ 7.70 – 7.61 (m, 2H), 7.61 – 7.51 (m, 4H), 7.45 (dd, *J* = 10.5, 4.8 Hz, 2H), 7.40 – 7.30 (m, 1H), 7.11 – 6.94 (m, 2H), 6.63 (t, *J* = 7.3 Hz, 1H), 6.39 (d, *J* = 8.0 Hz, 2H), 4.06 (s, 1H), 2.01 – 1.78 (m, 2H), 1.69 (s, 3H), 1.39 – 1.17 (m, 4H), 0.87 (t, *J* = 6.7 Hz, 3H).; ^{13}C NMR (101 MHz, $CDCl_3$) δ 146.2, 146.0, 141.0, 139.0, 128.8, 127.2, 127.1, 127.1, 126.8, 117.1, 115.4, 58.3, 44.5, 26.0, 25.8, 23.2, 14.2. HRMS (ESI) calcd. for $C_{24}H_{28}N$ $[M+H]^+$ *m/z* 330.2222, found 330.2221.

_N_-(2-(4-fluorophenyl)hexan-2-yl)aniline (1ac)

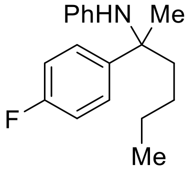

Prepared according to general procedure (B) from 1-(4-fluorophenyl)ethan-1-one to provide the title compound **1ac** as a yellow oil (1.00 g, 3.7 mmol, 74% total yield). ^{1}H NMR (400 MHz, $CDCl_3$) δ 7.55 – 7.38 (m, 2H), 7.02 (dd, *J* = 12.4, 4.8 Hz, 4H), 6.72 – 6.54 (m, 1H), 6.33 (dd, *J* = 8.6, 1.0 Hz, 2H), 4.02 (s, 1H), 2.02 – 1.71 (m, 2H), 1.65 (s, 3H), 1.37 – 1.05 (m, 4H), 0.86 (t, *J* = 7.0 Hz, 3H).; ^{13}C NMR (101 MHz, CDCl3) δ 161.6 (d, *J* = 244.4 Hz), 146.0, 142.4 (d, *J* = 3.0 Hz), 128.8, 127.9 (d, *J* = 8.1 Hz), 117.2, 115.4, 115.2 (d, *J* = 21.2 Hz), 58.1, 44.7, 26.0, 25.7, 23.1, 14.1. HRMS (ESI) calcd. for $C_{18}H_{23}NF$ $[M+H]^+$ *m/z* 272.1815, found 272.1813.

_N_-(2-(4-chlorophenyl)hexan-2-yl)aniline (1ad)

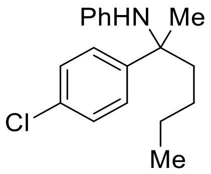

Prepared according to general procedure (B) from 1-(4-chlorophenyl)ethan-1-one to provide the title compound **1ad** as a colorless oil (0.92 g, 3.2 mmol, 64% total yield). ^{1}H NMR (400 MHz, $CDCl_3$) δ 7.42 (dd, *J* = 7.2, 1.5 Hz, 2H), 7.31 (dd, *J* = 5.0, 3.8 Hz, 2H), 7.03 (td, *J* = 7.4, 2.2 Hz, 2H), 6.69 – 6.61 (m, 1H), 6.33 (dd, *J* = 8.6, 1.0 Hz, 2H), 4.02 (s, 1H), 1.96 – 1.73 (m, 2H), 1.65 (s, 3H), 1.33 – 1.15 (m, 4H), 0.87 (t, *J* = 7.1 Hz, 3H).; ^{13}C NMR (101 MHz, $CDCl_3$) δ 145.9, 145.4, 132.2, 128.9, 128.6, 127.9, 117.3, 115.4, 58.2, 44.5, 26.0, 25.6, 23.1, 14.1. HRMS (ESI) calcd. for $C_{18}H_{23}NCl$ $[M+H]^+$ *m/z* 288.1519, found 288.1513.

_N_-(2-(4-(trifluoromethyl)phenyl)hexan-2-yl)aniline (1ae)

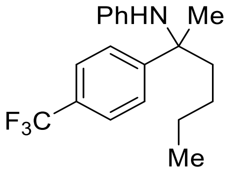

Prepared according to general procedure (B) from 1-(4-(trifluoromethyl)phenyl)ethan-1-one to provide the title compound **1ae** as a yellow oil (1.00 g, 3.1 mmol, 62% total yield). ^{1}H NMR (400 MHz, $CDCl_3$) δ 7.64 – 7.55 (m, 4H), 7.06 – 6.96 (m, 2H), 6.63 (ddt, *J* = 8.4, 7.4, 1.1 Hz, 1H), 6.33 – 6.25 (m, 2H), 4.04 (s, 1H), 1.97 – 1.71 (m, 2H), 1.66 (s, 3H), 1.35 – 1.11 (m, 4H), 0.85 (t, *J* = 7.0 Hz, 3H).; ^{13}C NMR (101 MHz, $CDCl_3$) δ 151.1, 145.7, 128.9, 128.7 (q, *J* = 32.3 Hz), 126.7, 125.5 (q, *J* = 4.0 Hz), 124.5 (q, *J* = 272.7 Hz), 117.5, 115.4, 58.5, 44.4, 25.9, 25.7, 23.1, 14.1. HRMS (ESI) calcd. for $C_{19}H_{23}NF_3$ $[M+H]^+$ *m/z* 322.1783, found 322.1778.

_N_-(2-(3-(trifluoromethyl)phenyl)hexan-2-yl)aniline (1af)

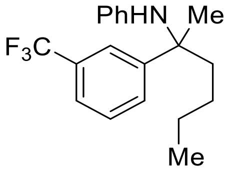

Prepared according to general procedure (B) from 1-(3-(trifluoromethyl)phenyl)ethan-1-one to provide the title compound **1af** as a yellow oil (0.55 g, 1.7 mmol, 34% total yield). ^{1}H NMR (400 MHz, $CDCl_3$) δ 7.74 – 7.64 (m, 2H), 7.51 (d, *J* = 7.7 Hz, 1H), 7.43 (t, *J* = 7.7 Hz, 1H), 7.05 – 6.96 (m, 2H), 6.63 (tt, *J* = 7.3, 1.1 Hz, 1H), 6.34 – 6.25 (m, 2H), 4.02 (s, 1H), 1.99 – 1.73 (m, 2H), 1.66 (s, 3H), 1.32 – 1.12 (m, 4H), 0.84 (t, *J* = 7.0 Hz, 3H).; ^{13}C NMR (101 MHz, $CDCl_3$) δ 148.2, 145.7, 130.8 (q, *J* = 32.3 Hz), 130.0, 129.0, 128.9, 124.5 (q, *J* = 273.7 Hz), 123.4 (q, *J* = 4.0 Hz), 122.9 (q, *J* = 4.0 Hz), 117.5, 115.5, 58.4, 44.2, 25.9, 25.8, 23.0, 14.1. HRMS (ESI) calcd. for $C_{19}H_{23}NF_3$ $[M+H]^+$ *m/z* 322.1783, found 322.1778.

Synthesis of _N_-(2-(3-cyclopropylphenyl)-5-methylhexan-2-yl)aniline (1l)

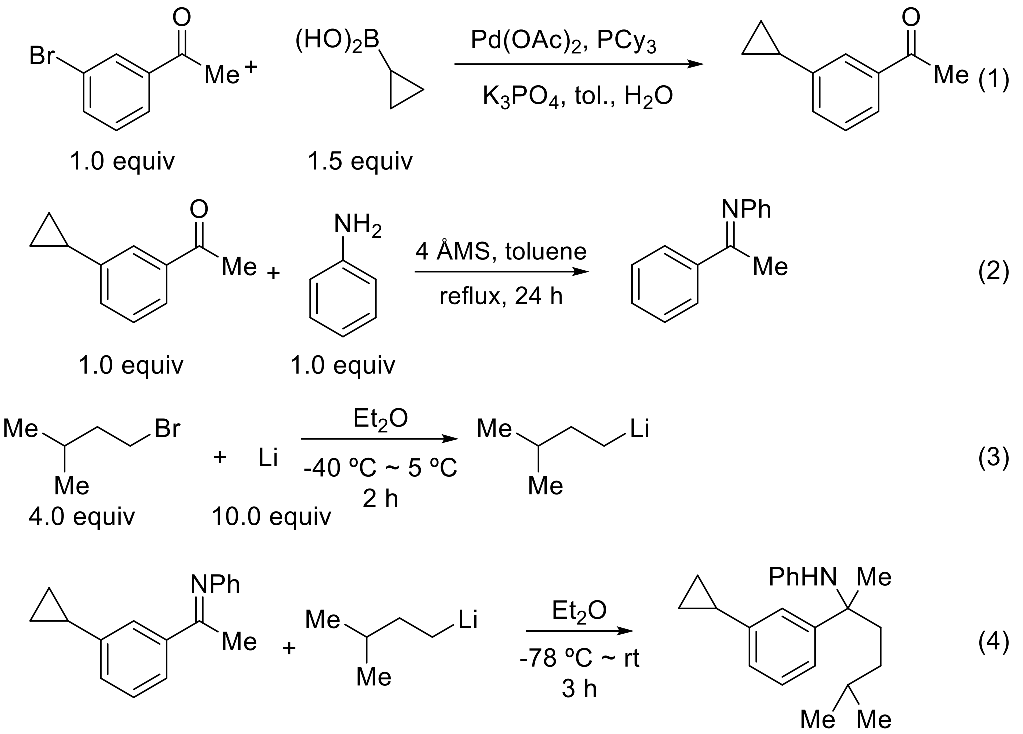

Step 1: Preparation of 1-(3-cyclopropylphenyl)ethan-1-one. The ketone was prepared according to a patent[1]. To a mixture of 1-(3-bromophenyl)ethan-1-one (10.0 mmol, 1.0 equiv), cyclopropylboronic acid (1.5 equiv), K_3PO_4 (3.0 equiv) and PCy_3 (0.2 equiv) in toluene/H_2O (0.2 M, 100:1) was added $Pd(OAc)_2$ (0.1 equiv) under N_2. The reaction was heated at 100 °C for 15 h. After completion, it was allowed to cool to rt and washed with water. The aqueous layer was extracted with EtOAc for 3 times and the combined organic layer was washed with brine, dried over Na_2SO_4. After concentrated under vaccum, the crude was purified by column chromatography on silica gel to provide the ketone as a colorless oil (1.55 g, 9.7 mmol, 97% total yield).

Step 2: Preparation of N,1-diphenylethan-1-imine. The imine was prepared according to *Step 1* of general procedure (A).

Step 3: Preparation of the lithium reagent. The lithium reagent was prepared according to *Step 2* of general procedure (A).

*Step 4: Preparation of N-(2-(3-cyclopropylphenyl)-5-methylhexan-2-yl)aniline (**1l**).* Prepared according to *Step 3* of general procedure (A) to provide the title compound **1l** as a colorless oil (1.35 g, 4.4 mmol, 88% total yield). ^{1}H NMR (400 MHz, $CDCl_3$) δ 7.28 – 7.24 (m, 1H), 7.24 – 7.16 (m, 2H), 7.05 – 6.96 (m, 2H), 6.94 – 6.85 (m, 2H), 6.65 – 6.57 (m, 1H), 6.35 (dd, J = 8.6, 1.0 Hz, 2H), 3.99 (s, 1H), 1.97 – 1.74 (m, 3H), 1.62 (s, 3H), 1.50 – 1.38 (m, 1H), 1.21 – 1.04 (m, 2H), 1.01 – 0.89 (m, 2H), 0.84 (d, J = 6.6 Hz, 3H), 0.82 (d, J = 6.6 Hz, 3H), 0.71 – 0.64 (m, 2H).; ^{13}C NMR (101 MHz, $CDCl_3$) δ 146.9, 146.3, 144.0, 128.8, 128.3, 123.9, 123.5, 123.1, 117.0, 115.5, 58.4, 42.2, 32.7, 28.4, 25.9, 22.8, 22.7, 15.8, 9.5, 9.5. HRMS (ESI) calcd. for $C_{22}H_{30}N$ $[M+H]^+$ *m/z* 308.2378, found 308.2368.

Synthesis of *N*-(2-(4-bromophenyl)-5-methylhexan-2-yl)aniline (1i).

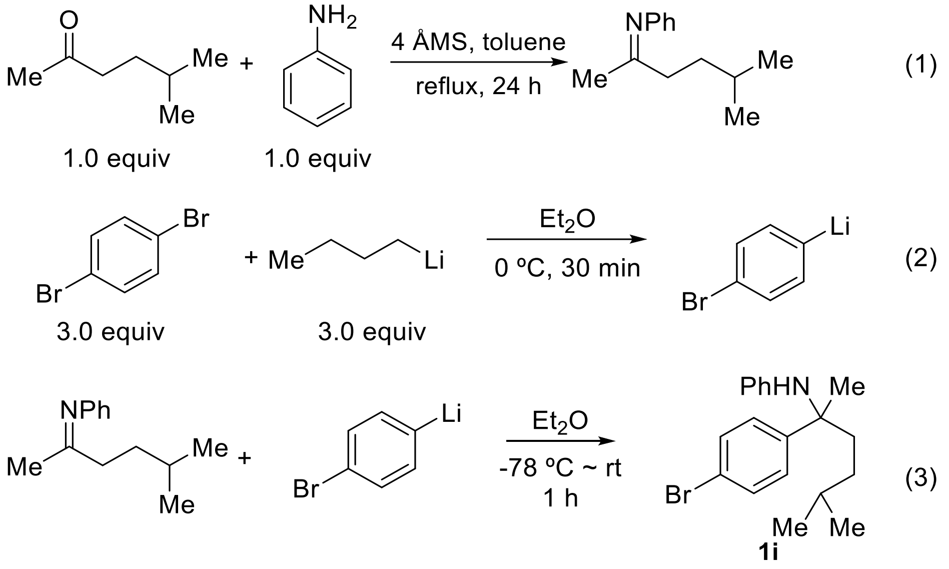

Step 1: Preparation of 5-methyl-N-phenylhexan-2-imine. The imine was prepared according to *Step 1* of general procedure (A).

Step 2: Preparation of (4-bromophenyl)lithium. Butyllithium (2.5 M in hexane, 3.0 equiv) was added dropwise to a vigorously stirred solution of 1,4-dibromobenzene in Et_2O (1.0 M) at 0 °C under N_2 atmosphere and stirred for another 30 min. After completion, the resulting blackish suspension was then used imediately.

*Step 3: Preparation of **1i**.* The freshly prepared lithium reagent was added dropwise to a vigorously stirred solution of imine in Et_2O (1.0 M) at -78 °C under N_2 atmosphere and stirred at room temperature for 1 h. After completion, the reaction was quenched with water. The resulting aqueous layer was extracted with EtOAc for 3 times and the combined organic layer was washed with brine, dried over Na_2SO_4 and concentrated under vacuum. The crude was purified by column chromatography on silica gel to provide the substrates **1i** as a white solid (0.69 g, 2.0 mmol, 10% total yield). ^{1}H NMR (400 MHz, $CDCl_3$) δ 7.47 – 7.36 (m, 2H), 7.32 (dd, *J* = 9.1, 2.4 Hz, 2H), 6.98 (tt, *J* = 7.8, 2.3 Hz, 2H), 6.60 (tt, *J* = 7.4, 1.8 Hz, 1H), 6.29 (dt, *J* = 8.9, 1.9 Hz, 2H), 3.95 (s, 1H), 1.94 – 1.66 (m, 2H), 1.59 (s, 3H), 1.49 – 1.32 (m, 1H), 1.13 – 0.99 (m, 2H), 0.81 (d, *J* = 6.6 Hz, 3H), 0.79 (d, *J* = 6.6 Hz, 3H). ^{13}C NMR (101 MHz, $CDCl_3$) ^{13}C NMR (101 MHz, $CDCl_3$) δ 146.0, 145.8, 131.5, 128.8, 128.3, 120.3, 117.3, 115.4, 58.2, 42.4, 32.6, 28.3, 25.5, 22.7, 22.6. HRMS (ESI) calcd. for $C_{19}H_{25}NBr$ $[M+H]^+$ *m/z* 346.1170, found 346.1166.

Synthesis of *N*-(2,8-dimethyl-5-phenylnonan-5-yl)aniline (1r).

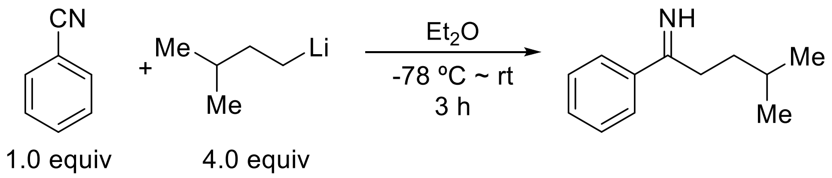

(1)

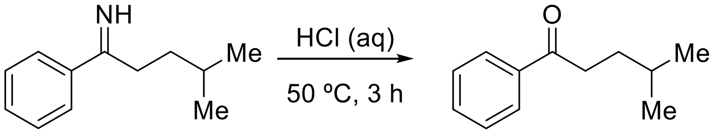

(2)

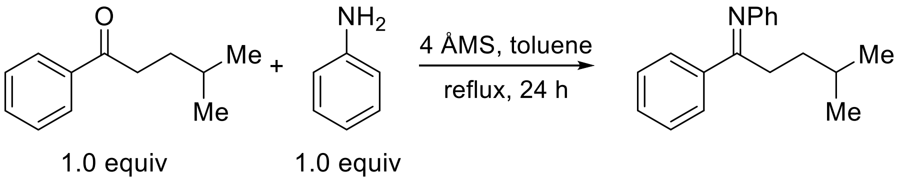

(3)

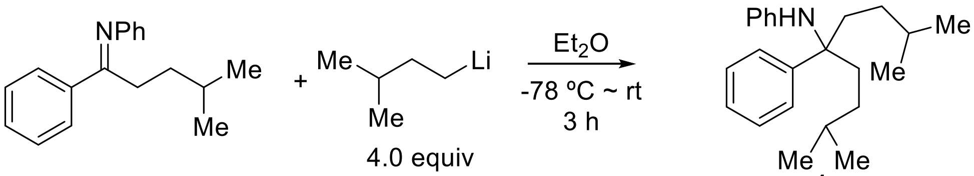

(4)

1r

Step 1: Preparation of 4-methyl-1-phenylpentan-1-imine. The freshly prepared isopentyllithium was added dropwise to a vigorously stirred solution of benzonitrile in Et_2O (1.0 M) at -78 °C under N_2 atmosphere and stirred at room temperature for 3 h. After completion, the reaction was quenched with water. The resulting aqueous layer was extracted with EtOAc for 3 times and the combined organic layer was washed with brine, dried over Na_2SO_4 and concentrated under vacuum. The crude was used directly.

Step 2: Hydrolysis of 4-methyl-1-phenylpentan-1-imine. The prepared crude of *Step 1* was mixed with dilute hydrochloric acid and stirred at 50 °C for 3 h. After completion, EtOAc was added and the organic layer was separated, the aqueous layer was extracted with EtOAc for 3 times and the combined organic layer was washed with brine, dried over Na_2SO_4 and concentrated under vacuum. The crude was used directly without further purification.

Step 3: Preparation of 4-methyl-N,1-diphenylpentan-1-imine. To an oven-dried round-bottom bottle equipped with a magnetic stir bar was added 4-methyl-1-phenylpentan-1-one (1.0 equiv), aniline (1.0 equiv), 4 ÅMS (0.2 g/mmol) and toluene (2.0 M). The mixture was then reflux for 24 h. After completion, it was allowed to cool to room temperature, and was directly filtered through a short pad of Celite®, washed with EtOAc. The filtrate was concentrated under vacuum and was used directly.

*Step 4: Preparation of N-(2,8-dimethyl-5-phenylnonan-5-yl)aniline (**1r**).* The freshly prepared lithium reagent was added dropwise to a vigorously stirred solution of 4-methyl-N,1-diphenylpentan-1-imine in Et_2O (1.0 M) at -78 °C under N_2 atmosphere and stirred at room temperature for 3 h. After completion, the reaction was quenched with water.

The resulting aqueous layer was extracted with EtOAc for 3 times and the combined organic layer was washed with brine, dried over Na_2SO_4 and concentrated under vacuum. The crude was purified by column chromatography on silica gel to provide the substrates **1r** as a colorless oil (0.65 g, 2.0 mmol, 40% total yield). ^{1}H NMR (400 MHz, $CDCl_3$) δ 7.53 – 7.43 (m, 2H), 7.33 (t, *J* = 7.6 Hz, 2H), 7.23 (t, *J* = 7.3 Hz, 1H), 7.03 – 6.91 (m, 2H), 6.58 (t, *J* = 7.3 Hz, 1H), 6.30 (d, *J* = 8.0 Hz, 2H), 3.95 (s, 1H), 2.01 (ddd, *J* = 13.2, 11.1, 5.7 Hz, 2H), 1.85 (ddd, *J* = 13.2, 10.8, 5.8 Hz, 2H), 1.49 – 1.32 (m, 2H), 1.13 – 0.94 (m, 4H), 0.82 (d, *J* = 6.7 Hz, 6H), 0.77 (d, *J* = 6.6 Hz, 6H).; ^{13}C NMR (101 MHz, $CDCl_3$) δ 146.4, 146.2, 128.7, 128.3, 126.8, 126.3, 116.9, 115.3, 60.7, 35.4, 32.2, 28.4, 22.8, 22.6. HRMS (ESI) calcd. for $C_{23}H_{34}N$ $[M+H]^+$ *m/z* 324.2691, found 324.2692.

Synthesis of *N*-(4-methyl-1-phenylpentyl)aniline (1w).

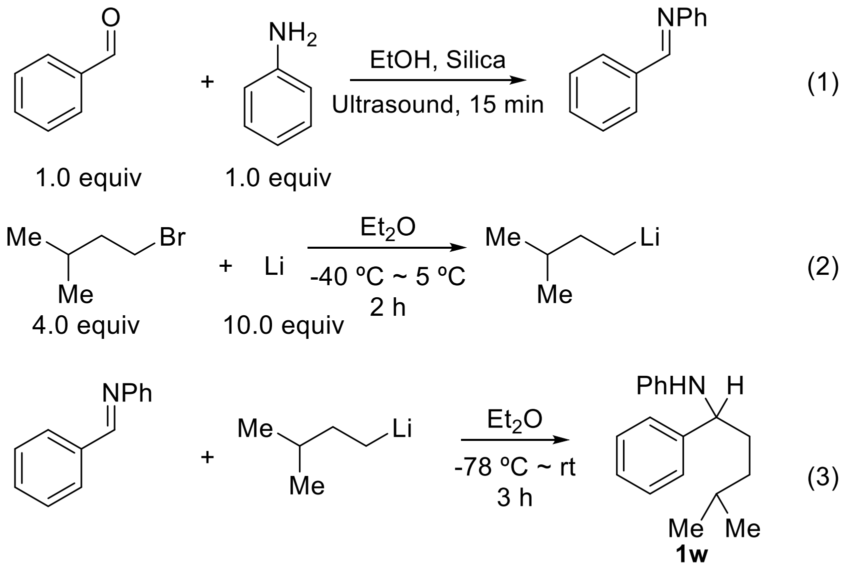

Step 1: Preparation of N,1-diphenylmethanimine. The imine was prepared according to the method developped by Guzen[2]. To an oven-dried round-bottom bottle was added benzaldehyde (1.0 equiv), aniline (1.0 equiv), silica (0.3 g/mmol) and EtOH (0.3 M). The mixture was then placed under ultrasound irradiation for 15 min. After completion, it was filtered, washed with EtOAc. The filtrate was concentrated under vacuum and was used directly.

Step 2: Preparation of lithium reagent. The lithium reagent was prepared according to *Step 2* of general procedure (A).

*Step 3: Preparation of the N-(4-methyl-1-phenylpentyl)aniline (**1w**).* The same as *Step 3* of general procedure (A) used N,1-diphenylmethanimine to provide the title compound **1w** as a colorless oil (0.83 g, 3.3 mmol, 66% total yield). ^{1}H NMR (400 MHz, $CDCl_3$) δ 7.36 – 7.26 (m, 4H), 7.25 – 7.17 (m, 1H), 7.13 – 7.02 (m, 2H), 6.62 (t, *J* = 7.3 Hz, 1H), 6.50 (d, *J* = 8.0 Hz, 2H), 4.25 (t, *J* = 6.8 Hz, 1H), 4.04 (s, 1H), 1.87 – 1.68 (m, 2H), 1.64 – 1.43 (m, 1H), 1.40 – 1.12 (m, 2H), 0.88 (d, J = 6.4 Hz, 3H), 0.86 (d, J = 6.4 Hz, 3H).; ^{13}C NMR (101 MHz, $CDCl_3$) δ 147.6, 144.5, 129.2, 128.7, 127.0, 126.5, 117.2, 113.3, 58.6, 37.0, 35.6, 28.1, 22.7, 22.6. HRMS (ESI) calcd. for $C_{18}H_{24}N$ $[M+H]^+$ *m/z* 254.1909, found 254.1905.

Synthesis of *N*-(4-methyl-1-phenylpentyl)-4-nitroaniline (1x).

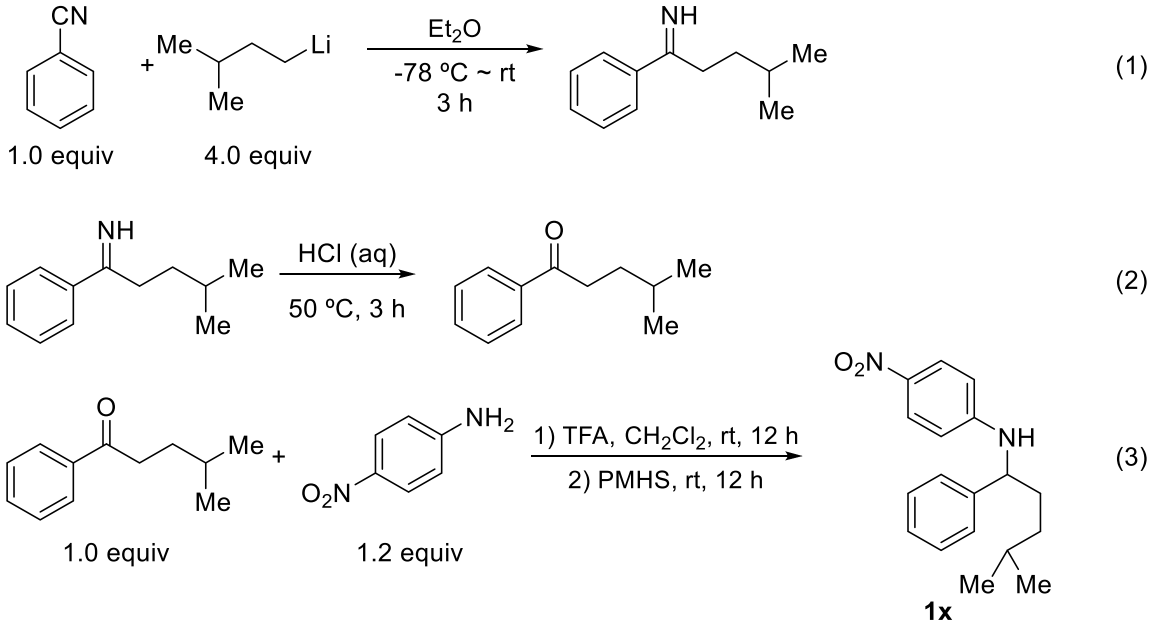

Step 1: Preparation of 4-methyl-1-phenylpentan-1-imine. The freshly prepared isopentyllithium was added dropwise to a vigorously stirred solution of benzonitrile in Et_2O (1.0 M) at -78 °C under N_2 atmosphere and stirred at room temperature for 3 h. After completion, the reaction was quenched with water. The resulting aqueous layer was extracted with EtOAc for 3 times and the combined organic layer was washed with brine, dried over Na_2SO_4 and concentrated under vacuum. The crude was used directly.

Step 2: Hydrolysis of 4-methyl-1-phenylpentan-1-imine. The prepared crude of *Step 1* was mixed with dilute hydrochloric acid and stirred at 50 °C for 3 h. After completion, EtOAc was added and the organic layer was separated, the aqueous layer was extracted with EtOAc for 3 times and the combined organic layer was washed with brine, dried over Na_2SO_4 and concentrated under vacuum. The crude was used directly without further purification.

*Step 3: Preparation of N-(4-methyl-1-phenylpentyl)-4-nitroaniline (**1x**).* The substrate **1x** was prepared according to the method developped by Patel[3]. To a stirred solution of 4-methyl-1-phenylpentan-1-one (1.0 equiv) and 4-nitroaniline (1.2 equiv) in dichloromethane (2.0 M) was added TFA (1.0 M). The mixture was stirred at room temperature for 12 h. PMHS [2.0 equiv of – MeSi(H)O– unit, average Mn: 1700–3200] was then added and the resulting mixture was again stirred for 12 h at rt. After completion, the reaction mixture was basified with 1 M aq sodium hydroxide to pH 8 and extracted with dichloromethane for 3 times. The combined organic layer was washed with brine, dried over Na_2SO_4 and concentrated under vacuum. The crude was purified by column chromatography on silica gel to provide the substrates **1y** as a yellow solid (0.75 g, 2.5 mmol, 25% total yield). ^{1}H NMR (400 MHz, $CDCl_3$) δ 8.06 – 7.92 (m, 2H), 7.38 – 7.31 (m, 2H), 7.31 – 7.23 (m, 3H), 6.51 – 6.39 (m, 2H), 4.90 (d, *J* = 6.1 Hz, 1H), 4.36 (q, *J* = 6.6 Hz, 1H), 1.91 – 1.77 (m, 2H), 1.63 – 1.47 (m, 1H), 1.40 – 1.10 (m,

2H), 0.89 (d, J = 6.6 Hz, 3H), 0.87 (d, J = 6.6 Hz, 3H).; ^{13}C NMR (101 MHz, $CDCl_3$) δ 152.6, 142.5, 138.2, 129.0, 127.7, 126.4, 126.3, 111.9, 58.5, 36.6, 35.4, 28.1, 22.7, 22.6. HRMS (ESI) calcd. for $C_{18}H_{23}N_2O_2$ $[M+H]^+$ *m/z* 299.1760, found 299.1759.

Synthesis of *N*-(5-cyclopropyl-1-phenylpent-4-en-1-yl)aniline (1aj).

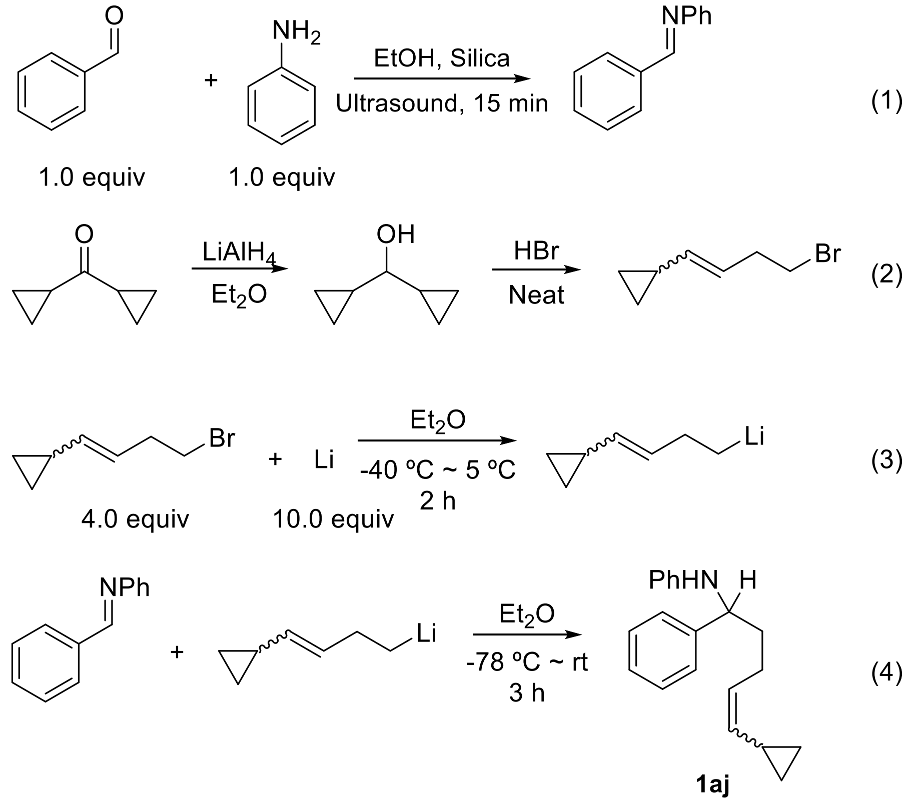

Step 1: Preparation of N,1-diphenylmethanimine. The imine was prepared according to the method developped by Guzen[1]. To an oven-dried round-bottom bottle was added benzaldehyde (1.0 equiv), aniline (1.0 equiv), Silica (0.3 g/mmol) and EtOH (0.3 M). The mixture was then placed under ultrasound irradiation for 15 min. After completion, it was filtered, washed with EtOAc. The filtrate was concentrated under vacuum and was used directly.

Step 2: Preparation of (4-bromobut-1-en-1-yl)cyclopropane. The bromide was prepared according to the method developped by Fifera[4] and Kataoka[5]. Dicyclopropylmethanone (1.0 equiv) dissolved in Et_2O (1.0 M) was added dropwise to a vigorously stirred suspension of $LiAlH_4$ (1.2 equiv) in Et_2O (1.0 M) at 0 °C under N_2 atmosphere and stirred for another 1 h at that temperature. After completion, the reaction was quenched with the sequential dropwise addition of water (0.05 mL/mmol), 10% NaOH (0.05 mL/mmol) and water (0.15 mL/mmol). The system was then filtered through Celite®, diluted with Et_2O, washed with water and the organic layer was dried over Na_2SO_4 and concentrated under vacuum. The crude was used directly. At 0 °C under N_2 atmosphere, HBr (5.0 equiv) was added to a vigorously stirred neat phase of the newly prepared dicyclopropylmethanol and stirred for 15 min. After completion, the system was extracted with hexane (0.2 mL/mmol) for 3 times and the combined organic layer was washed with brine and $NaHCO_3$ (aq.), dried over Na_2SO_4. After evaporation, the

crude was distilled (104~105 °C, 17 mmHg) to give 7.0 g (90%) a mixture of (*E*)- and (*Z*)-(4-bromobut-1-en-1-yl)cyclopropane.

Step 3: Preparation of (4-cyclopropylbut-3-en-1-yl)lithium. The lithium reagent was prepared according to *Step 2* of general procedure (A).

*Step 4: Preparation of N-(5-cyclopropyl-1-phenylpent-4-en-1-yl)aniline (**1aj**).* Prepared according to *Step 2* of general procedure (A) to provide the substrates **1aj** as a colorless oil (0.55 g, 2.0 mmol, 66%). ^{1}H NMR (400 MHz, $CDCl_3$) δ 7.40 – 7.27 (m, 4.6H), 7.25 – 7.18 (m, 1.15H), 7.14 – 7.04 (m, 2.3H), 6.65 – 6.62 (m, 1.15H), 6.56 – 6.48 (m, 2.3H), 5.52 (dt, *J* = 15.1, 6.7 Hz, 1H), 5.43 – 5.22 (m, 0.15H), 4.97 (dd, *J* = 15.3, 8.6 Hz, 1H), 4.79 (dd, *J* = 19.3, 8.9 Hz, 0.15H), 4.38 (t, *J* = 6.9 Hz, 0.15H), 4.33 (t, *J* = 6.8 Hz, 1H), 4.11 (s, 1.15H), 2.28 – 2.25 (m, 0.3H), 2.18 – 2.00 (m, 2H), 1.97 – 1.76 (m, 2.3H), 1.48 – 1.41 (m, 0.15H), 1.41 – 1.28 (m, 1H), 0.77 – 0.58 (m, 2.15H), 0.39 – 0.23 (m, 2.15H).; ^{13}C NMR (101 MHz, $CDCl_3$) δ 147.5, 147.5, 144.1, 135.2, 135.1, 129.1, 128.6, 126.9, 126.8, 126.8, 126.5, 126.4, 117.1, 113.3, 113.2, 57.8, 57.7, 38.8, 38.6, 29.3, 24.5, 13.6, 9.8, 6.9, 6.9, 6.5, 6.4. HRMS (ESI) calcd. for $C_{20}H_{24}N$ $[M+H]^+$ *m/z* 278.1909, found 278.1904.

Synthesis of *N*-(5-cyclopropyl-2-phenylpentan-2-yl)aniline (1ak).

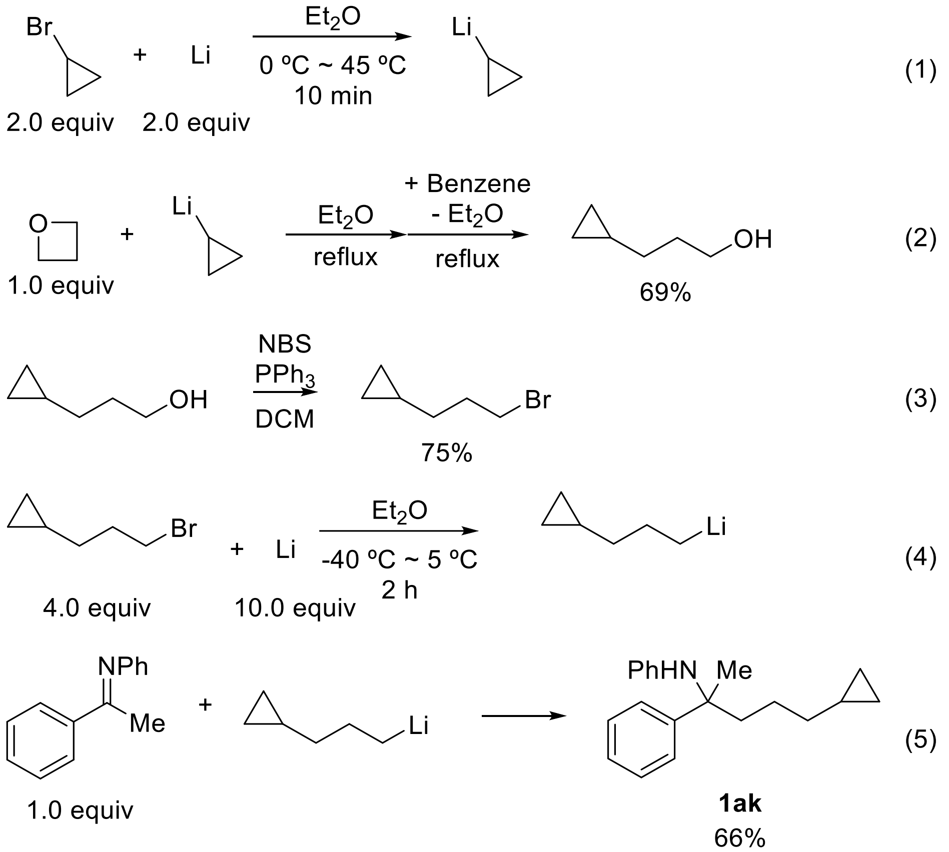

Step 1 & 2: Preparation of 3-cyclopropylpropan-1-ol. The alcohol was prepared according to the method developped by Wagner[6]. Bromocyclopropane (2.0 equiv) in Et_2O (5.0 M) was added dropwise to a vigorously stirred suspension of lithium shot (2.0 eqiv) in Et_2O (5.0 M) under an argon atmosphere. The resulting brown suspension of cyclopropyllithium and lithium

bromide was refluxed an additional 10 min and was then cooled in an ice bath. A solution of trimethylene oxide (1.0 equiv) in Et_2O (5.0 M) was added dropwise over 5 min. The resulting solution was allowed to stand at room temperature for 10 min and was then refluxed for 1 h. After 1.2 mL/mmol of benzene was added, the ether was distilled off and the solution was refluxed for 3.5 h. The cooled solution was treated with saturated aqueous ammonium chloride. The resulting aqueous layer was extracted once with Et_2O. The combined benzene and Et_2O solution was dried over Na_2SO_4. After evaporation, the crude was distilled (76 °C, 17 Torr) to give the 3-cyclopropylpropan-1-ol as a colorless oil (1.3 g, 13 mmol, 69%).

Step 3: Preparation of (3-bromopropyl)cyclopropane. The bromide was prepared according to the method developped by a patent[7]. To a solution of 3-cyclopropyl-propan-1-ol (1.0 equiv) in CH_2Cl_2 (0.5 M) was added PPh_3 (1.0 equiv) and NBS (1.0 equiv) at -20 °C. The reaction mixture was stirred at -20 °C to rt for overnight. The reaction was quenched by addition of water and extracted with CH_2Cl_2. The combined organic layer was washed with brine, dried over Na_2SO_4. After evaporation, the crude was purified by column chromatography on silica gel to provide the bromide as a colorless oil (1.70 g, 10.5 mmol, 75%).

Step 4: Preparation of (3-cyclopropylpropyl)lithium. The lithium reagent was prepared according to *Step 2* of general procedure (A).

*Step 5: Preparation of N-(5-cyclopropyl-2-phenylpentan-2-yl)aniline (**1ak**).* Prepared according to *Step 3* of general procedure (A) to provide the substrates **1ak** as a colorless oil (0.56 g, 2.0 mmol, 66%). ^{1}H NMR (400 MHz, $CDCl_3$) δ 7.51 – 7.45 (m, 2H), 7.33 (dd, J = 8.5, 6.9 Hz, 2H), 7.26 – 7.19 (m, 1H), 7.08 – 6.93 (m, 2H), 6.60 (tt, J = 7.3, 1.1 Hz, 1H), 6.41 – 6.29 (m, 2H), 4.03 (s, 1H), 2.08 – 1.76 (m, 2H), 1.66 (s, 3H), 1.38 – 1.29 (m, 2H), 1.15 – 1.09 (m, 2H), 0.66 – 0.50 (m, 1H), 0.42 – 0.28 (m, 2H), -0.02 – -0.16 (m, 2H).; ^{13}C NMR (101 MHz, $CDCl_3$) δ 146.8, 146.2, 128.8, 128.5, 126.4, 126.3, 117.0, 115.4, 58.5, 44.5, 35.1, 25.8, 23.9, 10.9, 4.6, 4.6. HRMS (ESI) calcd. for $C_{20}H_{26}N$ $[M+H]^+$ *m/z* 280.2065, found 280.2063.

Synthesis of (*Z*)-*N*-(2-phenyloct-5-en-2-yl)aniline (1ak').

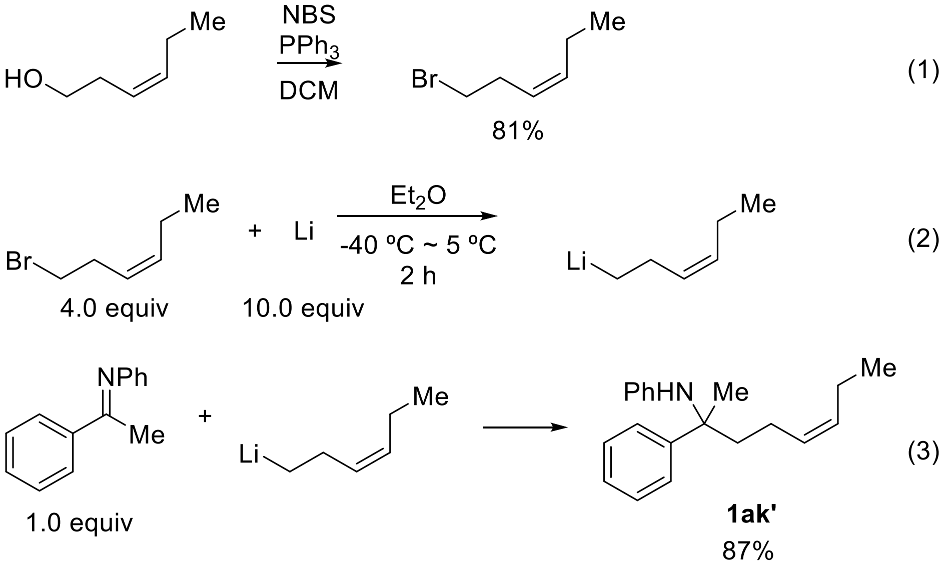

Step 1: Preparation of (Z)-1-bromohex-3-ene. The bromide was prepared according to *Step 3* of the synthesis of **1ak** using (*Z*)-hex-3-en-1-ol to provide the bromide as a colorless oil (13.2 g, 81 mmol, 81%).

Step 4: Preparation of (Z)-hex-3-en-1-yllithium. The lithium reagent was prepared according to *Step 2* of general procedure (A).

*Step 5: Preparation of (Z)-N-(2-phenyloct-5-en-2-yl)aniline (**1ak'**)*. Prepared according to *Step 3* of general procedure (A) to provide the substrates **1ak'** as a colorless oil (2.43 g, 8.7 mmol, 87%). ^{1}H NMR (400 MHz, $CDCl_3$) δ 7.48 (d, *J* = 7.6 Hz, 2H), 7.33 (t, *J* = 7.6 Hz, 2H), 7.24 (t, *J* = 7.3 Hz, 1H), 7.00 (t, *J* = 7.9 Hz, 2H), 6.61 (t, *J* = 7.3 Hz, 1H), 6.33 (d, *J* = 7.8 Hz, 2H), 5.37 – 5.24 (m, 2H), 4.07 (s, 1H), 2.16 – 1.76 (m, 6H), 1.66 (s, 3H), 0.88 (t, *J* = 7.5 Hz, 3H).; ^{13}C NMR (101 MHz, $CDCl_3$) δ 146.6, 146.1, 132.4, 128.8, 128.5, 128.4, 126.5, 126.2, 117.2, 115.4, 58.5, 43.8, 26.3, 21.8, 20.5, 14.4. HRMS (ESI) calcd. for $C_{20}H_{25}NNa$ $[M+Na]^+$ *m/z* 302.1885, found 302.1889.

Synthesis of *N*-methyl-N-(5-methyl-2-phenylhexan-2-yl)aniline (1al).

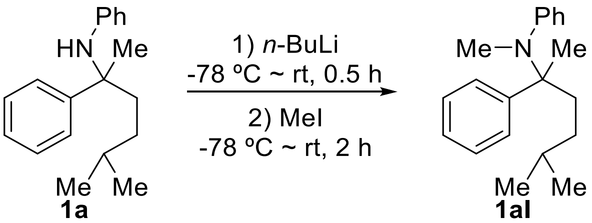

1a **1al**

Butyllithium (2.5 M in hexane, 3.0 equiv) was added dropwise to a vigourously stirred solution of **1a** in Et_2O (1.0 M) at -78 °C under N_2 atmosphere and stirred for another 30 min at rt, then MeI (3.0 equiv) in Et_2O (1.0 M) was added to the above mixture at -78 °C under N_2 atmosphere and stirred for another 2 h. After completion, the reaction was quenched with water. The resulting aqueous layer was extracted with EtOAc for 3 times and the combined organic layer was washed with brine, dried over Na_2SO_4 and concentrated under vacuum. The crude was purified by column chromatography on silica gel to provide the substrates **1al** as a colorless oil (1.21 g, 4.3 mmol, 86%). ^{1}H NMR (400 MHz, $CDCl_3$) δ 7.44 – 7.38 (m, 2H), 7.31 (dd, *J* = 8.5, 6.9 Hz, 2H), 7.25 – 7.19 (m, 1H), 7.18 – 7.12 (m, 2H), 6.96 – 6.86 (m, 3H), 2.79 (s, 3H), 1.99 – 1.78 (m, 2H), 1.51 (s, 3H), 1.40 – 1.30 (m, 1H), 1.07 – 0.81 (m, 2H), 0.76 (d, *J* = 6.6 Hz, 3H), 0.73 (d, *J* = 6.6 Hz, 3H).; ^{13}C NMR (101 MHz, $CDCl_3$) δ 150.4, 147.5, 128.1, 128.0, 126.9, 126.2, 124.4, 121.4, 63.6, 39.6, 37.5, 33.7, 28.6, 22.7, 22.7, 22.4. HRMS (ESI) calcd. for $C_{20}H_{28}N$ $[M+H]^+$ *m/z* 282.2222, found 282.2217.

Synthesis of 4-methyl-1-phenylpentan-1-amine (1an).

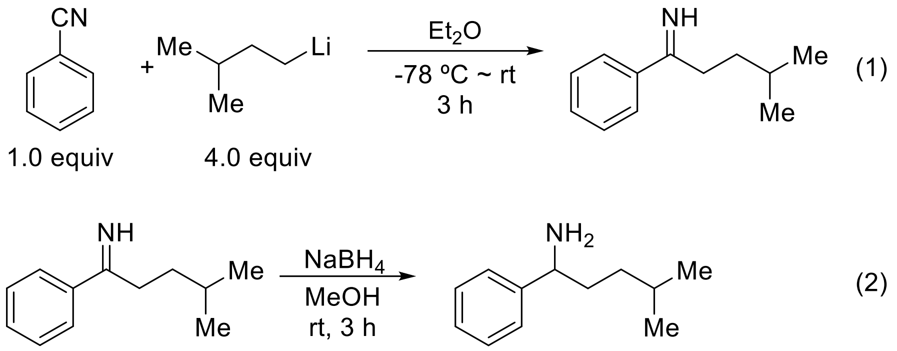

1an

Step 1: Preparation of 4-methyl-1-phenylpentan-1-imine. The same as *Step 1* from the synthesis of **1v**.

Step 2: Reduction of imine. The prepared crude of *Step 1* was dissolved in MeOH (0.5 M) and cooled to 0 °C. $NaBH_4$ (3.0 equiv) was added in several portions and the resulting mixture was allowed to warm to room temperature and stirred for 3 h. After completion, the reaction was quenched with saturated aqueous solution of ammonium chloride. The mixture was diluted with DCM and separated. The aqueous phase was extracted with DCM for 3 times and the combined organic layer was washed with brine, dried over Na_2SO_4 and concentrated under vacuum. The crude was purified by column chromatography on silica gel to provide the substrates **1an** as a colorless oil (4.67 g, 26.4 mmol, 66%). ^{1}H NMR (400 MHz, $CDCl_3$) δ 7.34 – 7.28 (m, 4H), 7.25 – 7.21 (m, 1H), 3.83 (t, *J* = 6.9 Hz, 1H), 1.65 (dtd, *J* = 8.7, 6.7, 1.4 Hz, 2H), 1.55 – 1.49 (m, 3H), 1.24 (ddt, *J* = 13.2, 10.0, 6.9 Hz, 1H), 1.07 (ddt, *J* = 13.3, 9.0, 6.7 Hz, 1H), 0.86 (d, *J* = 6.4 Hz, 3H), 0.85 (d, *J* = 6.4 Hz, 3H).; ^{13}C NMR (101 MHz, $CDCl_3$) δ 146.9, 128.5, 126.9, 126.4, 56.7, 37.6, 35.9, 28.2, 22.7, 22.7. HRMS (ESI) calcd. for $C_{20}H_{28}N$ $[M+H]^+$ *m/z* 178.1596, found 178.1593.

Synthesis of *N*-(4-methyl-1-phenylpentyl)acetamide (1ap).

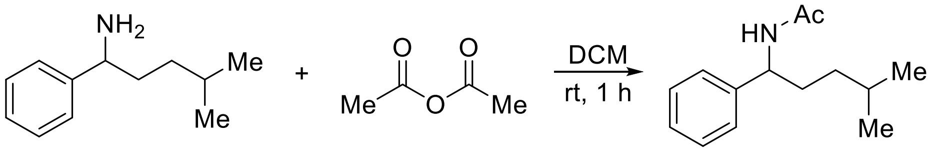

1ap

1an was dissolved in DCM (0.2 M) and cooled to 0 °C, acetic anhydride (1.2 equiv) was added dropwise. Then the system was allowed to warm to room temperature and stirred for 1 h. After completion, the reaction was quenched with water. The mixture was diluted with DCM and separated. The aqueous phase was extracted with DCM for 3 times and the combined organic layer was washed with brine, dried over Na_2SO_4 and concentrated under vacuum. The crude was purified by column chromatography on silica gel to provide the substrates **1ap** as a colorless oil (2.11 g, 9.6 mmol, 96%). ^{1}H NMR (400 MHz, $CDCl_3$) δ 7.40 – 7.15 (m, 5H), 6.55 – 6.51 (m, 1H), 4.90 (q, *J* = 7.6 Hz, 1H), 1.93 (d, *J* = 1.8 Hz, 3H), 1.76 (tq, *J* = 13.6, 7.5 Hz, 2H), 1.53 (tt, *J* = 13.1, 6.6 Hz, 1H), 1.30 – 1.02 (m, 2H), 0.85 (d, *J* = 6.6 Hz, 6H).; ^{13}C NMR (101 MHz, $CDCl_3$) δ 169.5, 142.7, 128.6, 127.2, 126.6, 53.8, 35.4, 34.2, 27.9, 23.3, 22.6, 22.5. HRMS (ESI) calcd. for $C_{20}H_{28}N$ $[M+H]^+$ *m/z* 220.1701, found 220.1700.

Synthesis of *N*-(4-methyl-1-phenylpentyl)acetamide (1aq).

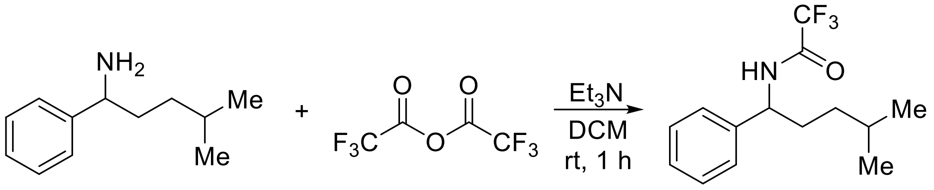

1aq

1an and Et_3N (1.2 equiv) were dissolved in DCM (0.2 M) and cooled to 0 °C, Trifluoroacetic anhydride (1.2 equiv) was added dropwise. Then the system was allowed to warm to room temperature and stirred for 1 h. After completion, the reaction was quenched with water. The mixture was diluted with DCM and separated. The aqueous phase was extracted with DCM for 3 times and the combined organic layer was washed with brine, dried over Na_2SO_4 and concentrated under vacuum. The crude was purified by column chromatography on silica gel to provide the substrates **1aq** as a white solid (1.19 g, 4.4 mmol, 88%). ^{1}H NMR (400 MHz, $CDCl_3$) δ 7.43 – 7.35 (m, 2H), 7.34 – 7.31 (m, 1H), 7.31 – 7.27 (m, 2H), 6.69 (s, 1H), 4.93 (q, *J* = 7.7 Hz, 1H), 1.96 – 1.80 (m, 2H), 1.56 (dp, *J* = 13.3, 6.7 Hz, 1H), 1.17 (dddt, *J* = 45.0, 13.3, 10.0, 6.5 Hz, 2H), 0.87 (d, *J* = 6.6 Hz, 6H).; ^{13}C NMR (101 MHz, $CDCl_3$) δ 156.6 (q, *J* = 37.1 Hz), 140.3, 129.1, 128.2, 126.7, 116.0 (q, *J* = 289.2 Hz), 54.9, 35.2, 33.5, 27.9, 22.6, 22.5. HRMS (ESI) calcd. for $C_{20}H_{28}N$ $[M+H]^+$ *m/z* 274.1419, found 274.1420.

Synthesis of 4-methyl-*N*-(4-methyl-1-phenylpentyl)benzenesulfonamide (1ar).

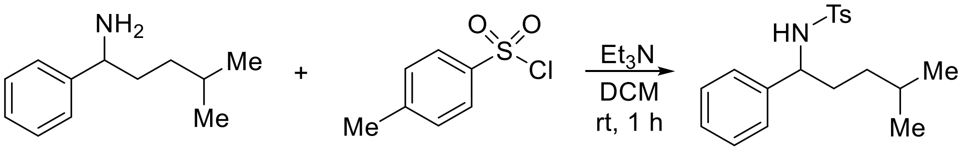

1ar

1an and Et_3N (1.2 equiv) were dissolved in DCM (0.2 M) and cooled to 0 °C, Tosyl chloride (1.2 equiv) was added dropwise. Then the system was allowed to warm to room temperature and stirred for 1 h. After completion, the reaction was quenched with water. The mixture was diluted with DCM and separated. The aqueous phase was extracted with DCM for 3 times and the combined organic layer was washed with brine, dried over Na_2SO_4 and concentrated under vacuum. The crude was purified by column chromatography on silica gel to provide the substrates **1ar** as a white solid (1.45 g, 4.4 mmol, 92%). ^{1}H NMR (400 MHz, $CDCl_3$) δ 7.54 (d, *J* = 8.3 Hz, 2H), 7.18 – 7.05 (m, 5H), 7.01 (dd, *J* = 6.5, 3.0 Hz, 2H), 5.44 (d, *J* = 7.7 Hz, 1H), 4.22 (q, *J* = 7.4 Hz, 1H), 2.34 (s, 3H), 1.89 – 1.56 (m, 2H), 1.42 (dp, *J* = 13.2, 6.6 Hz, 1H), 1.21 – 1.07 (m, 1H), 1.03 – 0.87 (m, 1H), 0.77 (d, *J* = 6.6 Hz, 3H), 0.76 (d, *J* = 6.6 Hz, 3H).; ^{13}C NMR (101 MHz, $CDCl_3$) δ 142.9, 141.2, 137.8, 129.3, 128.4, 127.2, 127.1, 126.6, 58.7, 35.6, 35.0, 27.7, 22.5, 21.5. HRMS (ESI) calcd. for $C_{20}H_{28}N$ $[M+H]^+$ *m/z* 332.1684, found 332.1681.

Synthesis of *N*-ethyl-4,4-dimethyl-1,2,3,4-tetrahydronaphthalen-1-amine (1ao).

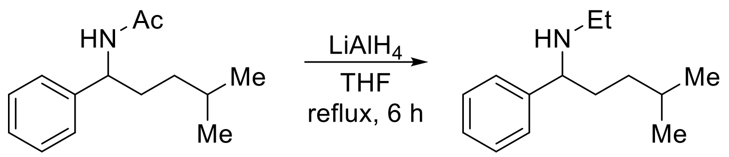

1ao

1ap was dissolved in THF (0.1 M) and cooled to 0 °C, lithium aluminium hydride (5.0 equiv) was added in several portions. Then the system was allowed to reflux for 6 h. After completion, the reaction was quenched with aqueous saturated sodium potassium tartrate solution. The mixture was diluted with EtOAc and separated. The aqueous phase was extracted with EtOAc for 3 times and the combined organic layer was washed with brine, dried over Na_2SO_4 and concentrated under vacuum. The crude was purified by column chromatography on silica gel to provide the substrates **1ao** as a colorless oil (0.84 g, 4.1 mmol, 82%). ^{1}H NMR (400 MHz, $CDCl_3$) δ 7.38 – 7.17 (m, 5H), 3.58 – 3.45 (m, 1H), 2.58 – 2.33 (m, 2H), 1.83 – 1.55 (m, 2H), 1.48 (tq, *J* = 13.1, 6.6 Hz, 1H), 1.31 (s, 1H), 1.16 (tt, *J* = 11.7, 5.8 Hz, 1H), 1.05 (t, *J* = 7.1 Hz, 3H), 1.01 – 0.93 (m, 1H), 0.83 (d, *J* = 6.4 Hz, 3H), 0.82 (d, *J* = 6.4 Hz, 3H).; ^{13}C NMR (101 MHz, $CDCl_3$) δ 144.7, 128.3, 127.3, 126.9, 64.0, 42.0, 36.2, 35.6, 28.2, 22.7, 22.6, 15.5. HRMS (ESI) calcd. for $C_{20}H_{28}N$ $[M+H]^+$ *m/z* 206.1909, found 206.1907.

III. Copper-catalyzed oxidative C(sp^3)-H/ C(sp^2)-H cross-coupling en route to carbocyclic rings

Reaction Optimization

Table S1 | Copper Catalyst Screening

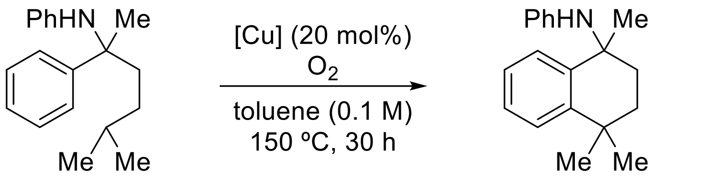

1a **2a**

entry	[Cu]	yield %[b]	entry	[Cu]	yield %[b]
1	CuCl	0	7	$CuBr_2$	0
2	CuBr	0	8	$CuSO_4$	0
3	CuCN	0	9	$Cu(OTf)_2$	0
4	Cu_2O	0	10	$Cu(BF_4)_2$	0
5	$CuCl_2$	0	11	$Cu(acac)_2$	12
6	Cu	9	12	$Cu(TFA)_2$	0

[a]Conditions: **1a** (0.1 mmol, 1 equiv), Cat. (20 mol%), solvent (0.1 M), 150 °C, 30 h. [b]Determined by TLC and isolated yield.

Table S2 | Oxidant Screening

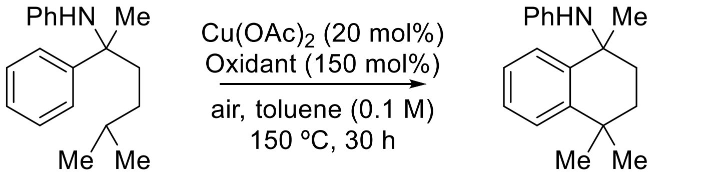

1a **2a**

entry	Oxidant	yield %[b]	entry	Oxidant	yield %[b]
1	TEMPO	0	8	Selectfluor	0
2	PIDA	0	9	DDQ	0
3	$(NH_4)_2S_2O_8$	0	10	$FeCl_3$	0
4	$Na_2S_2O_8$	0	11	Ag_2CO_3	49
5	$Ce(SO_4)_2$	0	12	AgOAc	38
6	BQ	0	13	$AgNO_3$	0
7	NFSI	0	14	Ag_2O	9
			15	BPO	0

[a]Conditions: **1a** (0.1 mmol, 1 equiv), Cat. (20 mol%), oxidant (150 mol%), solvent (0.1 M), 150 °C, 30 h. [b]Determined by TLC and isolated yield.

Table S3 | Solvent Screening

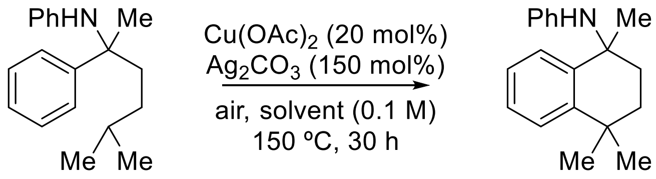

1a **2a**

entry	solvent	yield %[b]	entry	solvent	yield %[b]
1	EtOAc	0	9	EtOH	0
2	$CHCl_3$	0	10	*o*-xylene	trace
3	CCl_4	0	11	*m*-xylene	trace
4	1,2-DCE	72	12	*p*-xylene	trace
5	MeCN	0	13	mesitylene	0
6	DMSO	0	14	PhCl	trace
7	DMF	0	15	$PhCF_3$	55
8	Acetone	0	16	Dioxane	0

[a]Conditions: **1a** (0.1 mmol, 1 equiv), Cat. (20 mol%), oxidant (150 mol%), solvent (0.1 M), 150 °C, 30 h. [b]Determined by TLC and isolated yield.

Table S4 | Final Variation

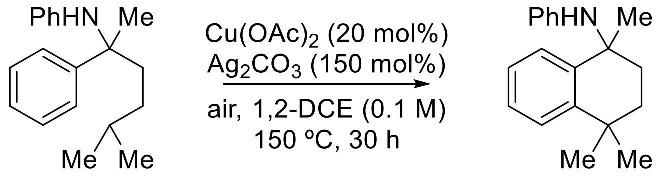

1a **2a**

entry	variation	yield %[b]
1	135 °C	81
2	135 °C, 0.5 M 1,2-DCE	85
3	135 °C, 0.5 M 1,2-DCE, 25 h	92
4	entry 3, 10% Cu(OAc)2	87
5	entry 3, 5% Cu(OAc)2	83
6	entry 3, 100% Ag2CO3	83
7	entry 3, 200% Ag2CO3	85
8	entry 3, 12 h	77
9	entry 3, 120 °C	60

[a]Conditions: **1a** (0.1 mmol, 1 equiv), Cat. (20 mol%), oxidant (150 mol%), solvent (0.1 M), 150 °C, 30 h. [b]Determined by TLC and isolated yield.

Substrate Screening

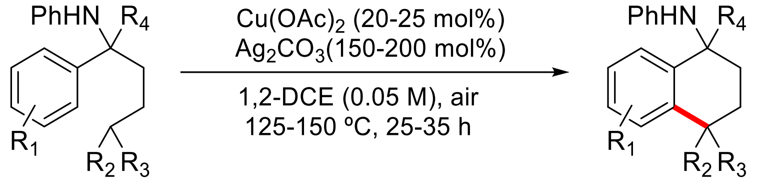

Genaral procedure (C) for the copper-catalyzed oxidative C(sp3)-H/ C(sp2)-H cross-coupling en route to carbocyclic rings. To an oven-dried 35 mL screw-cap sealed tube equipped with a magnetic stir bar was added $Cu(OAc)_2$ (20 mol%), Ag_2CO_3 (150 mol%), substrate **1** (0.1 mmol, 1.0 equiv) and 1,2-dichloroethane (2.0 mL) at air atmosphere. The vessel was then sealed with a Teflon screw-cap and placed into a preheated oil bath at 135 °C for 25 h. After completion, the reaction mixture was allowed to cool to room temperature, and was directly filtered through a short pad of silica gel washed with EtOAc. The filtrate was concentrated under vacuum and purified by chromatography on silica gel to obtain the corresponding product **2**.

1,4,4-trimethyl-*N*-phenyl-1,2,3,4-tetrahydronaphthalen-1-amine (2a)

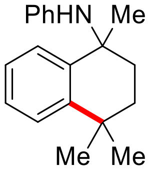

Prepared according to general procedure (C) using **1a** to provide the title compound **2a** as a colorless oil (24.4 mg, 0.92 mmol, 92%). ^{1}H NMR (400 MHz, $CDCl_3$) δ 7.59 (dd, *J* = 7.9, 1.5 Hz, 1H), 7.39 (dd, *J* = 7.9, 1.4 Hz, 1H), 7.23 (td, *J* = 7.6, 1.5 Hz, 1H), 7.14 (td, *J* = 7.5, 1.3 Hz, 1H), 7.05 – 6.96 (m, 2H), 6.66 – 6.57 (m, 1H), 6.33 – 6.24 (m, 2H), 4.04 (s, 1H), 2.72 (td, *J* = 13.9, 3.4 Hz, 1H), 1.98 (td, *J* = 14.0, 3.2 Hz, 1H), 1.72 (dt, *J* = 13.8, 3.8 Hz, 1H), 1.57 (s, 3H), 1.50 (dt, *J* = 13.6, 3.7 Hz, 1H), 1.41 (s, 3H), 1.35 (s, 3H).; ^{13}C NMR (101 MHz, $CDCl_3$) δ 145.9, 144.7, 141.2, 128.9, 126.9, 126.9, 126.8, 126.4, 117.2, 115.3, 55.8, 36.4, 34.0, 33.9, 32.1, 31.6, 30.1. HRMS (ESI) calcd. for $C_{19}H_{24}N$ $[M+H]^+$ *m/z* 266.1909, found 266.1907.

1,4,4,6-tetramethyl-*N*-phenyl-1,2,3,4-tetrahydronaphthalen-1-amine (2b)

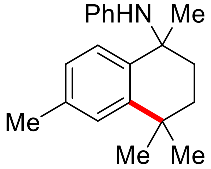

Prepared according to general procedure (C) (but for 20 h) using **1b** to provide the title compound **2b** as a colorless oil (24.3 mg, 0.87 mmol, 87%). ^{1}H NMR (400 MHz, $CDCl_3$) δ 7.46 (d, *J* = 8.0 Hz, 1H), 7.18 (d, *J* = 1.8 Hz, 1H), 7.05 – 6.98 (m, 2H), 6.96 (dd, *J* = 8.1, 1.8 Hz, 1H), 6.62 (tt, *J* = 7.3, 1.1 Hz, 1H), 6.34 – 6.27 (m, 2H), 4.02 (s, 1H), 2.68 (td, *J* = 13.8, 3.3 Hz, 1H), 2.35 (s, 3H), 1.95 (td, *J* = 14.0, 3.3 Hz, 1H), 1.70 (ddd, *J* = 13.8, 4.3, 3.4 Hz, 1H), 1.55 (s, 3H), 1.48 (ddd, *J* = 13.6, 4.4, 3.3 Hz, 1H), 1.39 (s, 3H), 1.33 (s, 3H).; ^{13}C NMR (101 MHz, $CDCl_3$) δ

146.0, 144.4, 138.2, 136.2, 128.8, 127.5, 127.4, 126.8, 117.1, 115.3, 55.7, 36.5, 34.0, 33.9, 32.1, 31.6, 30.1, 21.5. HRMS (ESI) calcd. for $C_{20}H_{26}N$ $[M+H]^+$ *m/z* 280.2065, found 280.2065.

6-isopropyl-1,4,4-trimethyl-*N*-phenyl-1,2,3,4-tetrahydronaphthalen-1-amine (2c)

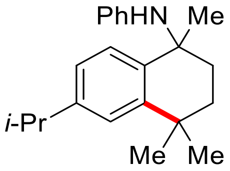

Prepared according to general procedure (C) using **1c** to provide the title compound **2c** as a colorless oil (23.0 mg, 0.75 mmol, 75%). ^{1}H NMR (400 MHz, $CDCl_3$) δ 7.47 (d, *J* = 8.1 Hz, 1H), 7.20 (d, *J* = 1.9 Hz, 1H), 7.04 – 6.96 (m, 3H), 6.61 (tt, *J* = 7.2, 1.1 Hz, 1H), 6.33 – 6.26 (m, 2H), 4.00 (s, 1H), 2.89 (hept, *J* = 6.9 Hz, 1H), 2.66 (td, *J* = 13.7, 3.3 Hz, 1H), 1.93 (td, *J* = 13.8, 3.2 Hz, 1H), 1.69 (ddd, *J* = 13.8, 4.6, 3.3 Hz, 1H), 1.57 (s, 3H), 1.48 (ddd, *J* = 13.5, 4.6, 3.2 Hz, 1H), 1.39 (s, 3H), 1.33 (s, 3H), 1.27 (d, *J* = 7.0 Hz, 3H), 1.26 (d, *J* = 7.0 Hz, 3H).; ^{13}C NMR (101 MHz, $CDCl_3$) δ 145.9, 144.6, 141.3, 128.8, 127.1, 126.8, 126.0, 117.1, 115.5, 58.0, 46.8, 35.8, 34.1, 32.2, 32.1, 26.2, 17.0, 14.7. HRMS (ESI) calcd. for $C_{22}H_{30}N$ $[M+H]^+$ *m/z* 308.2378, found 308.2377.

1,4,4-trimethyl-6-pentyl-*N*-phenyl-1,2,3,4-tetrahydronaphthalen-1-amine (2d)

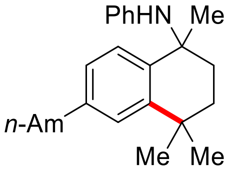

Prepared according to general procedure (C) using **1d** to provide the title compound **2d** as a colorless oil (26.8 mg, 0.80 mmol, 80%). ^{1}H NMR (400 MHz, $CDCl_3$) δ 7.45 (d, *J* = 8.0 Hz, 1H), 7.15 (d, *J* = 1.8 Hz, 1H), 7.02 – 6.96 (m, 2H), 6.94 (dd, *J* = 8.0, 1.9 Hz, 1H), 6.60 (tt, *J* = 7.4, 1.1 Hz, 1H), 6.31 – 6.24 (m, 2H), 4.00 (s, 1H), 2.65 (td, *J* = 13.7, 3.3 Hz, 1H), 2.58 (dd, *J* = 8.9, 6.7 Hz, 2H), 1.93 (td, *J* = 13.8, 3.2 Hz, 1H), 1.72 – 1.65 (m, 1H), 1.65 – 1.59 (m, 2H), 1.55 (s, 3H), 1.47 (ddd, *J* = 13.5, 4.4, 3.2 Hz, 1H), 1.37 (s, 3H), 1.36 – 1.33 (m, 4H), 1.32 (s, 3H), 0.91 (t, *J* = 8.0 Hz, 3H).; ^{13}C NMR (101 MHz, $CDCl_3$) δ 146.0, 144.4, 141.2, 138.4, 128.8, 126.7, 126.7, 117.1, 115.4, 55.7, 36.5, 35.9, 34.0, 33.7, 32.1, 31.8, 31.5, 31.2, 30.2, 22.7, 14.2. HRMS (ESI) calcd. for $C_{24}H_{34}N$ $[M+H]^+$ *m/z* 336.2691, found 336.2688.

1,4,4-trimethyl-*N*,6-diphenyl-1,2,3,4-tetrahydronaphthalen-1-amine (2e)

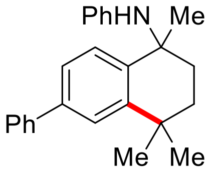

Prepared according to general procedure (C) using **1e** to provide the title compound **2e** as a colorless oil (22.5 mg, 0.66 mmol, 66%). ^{1}H NMR (400 MHz, $CDCl_3$) δ 7.72 – 7.62 (m, 4H), 7.48 (t, *J* = 7.5 Hz, 2H), 7.44 – 7.35 (m, 2H), 7.10 – 7.01 (m, 2H), 6.66 (t, *J* = 7.3 Hz, 1H), 6.36

(d, J = 8.0 Hz, 2H), 4.09 (s, 1H), 2.76 (td, J = 13.9, 3.2 Hz, 1H), 2.04 (td, J = 14.1, 3.1 Hz, 1H), 1.77 (dt, J = 14.0, 3.8 Hz, 1H), 1.63 (s, 3H), 1.54 (dt, J = 13.7, 3.8 Hz, 1H), 1.50 (s, 3H), 1.42 (s, 3H).; ^{13}C NMR (101 MHz, $CDCl_3$) δ 145.8, 145.0, 141.4, 140.4, 139.5, 128.9, 128.9, 128.8, 127.4, 127.2, 125.7, 125.3, 117.3, 115.4, 55.8, 36.4, 34.2, 33.8, 32.1, 31.6, 30.1. HRMS (ESI) calcd. for $C_{25}H_{28}N$ $[M+H]^+$ *m/z* 342.2222, found 342.2217.

1,4,4-trimethyl-6-(methylthio)-*N*-phenyl-1,2,3,4-tetrahydronaphthalen-1-amine (2f)

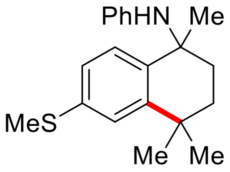

Prepared according to general procedure (C) using **1f** to provide the title compound **2f** as a white solid (21.5 mg, 0.69 mmol, 69%). ^{1}H NMR (400 MHz, DMSO-d_6) δ 6.47 (d, J = 8.3 Hz, 1H), 6.35 (d, J = 2.0 Hz, 1H), 6.12 (dd, J = 8.3, 1.9 Hz, 1H), 6.01 (t, J = 7.7 Hz, 2H), 5.54 (t, J = 7.3 Hz, 1H), 5.39 (d, J = 8.0 Hz, 2H), 4.97 (s, 1H), 1.74 – 1.60 (m, 1H), 1.58 (s, 3H), 1.05 (td, J = 14.2, 3.0 Hz, 1H), 0.77 (dt, J = 14.5, 3.8 Hz, 1H), 0.58 (s, 3H), 0.53 – 0.49 (m, 1H), 0.48 (s, 3H), 0.42 (s, 3H).; ^{13}C NMR (101 MHz, DMSO-d_6) δ 146.0, 144.6, 138.2, 135.2, 127.9, 126.7, 123.8, 123.6, 115.2, 114.1, 54.2, 35.5, 33.4, 32.8, 31.4, 30.6, 28.5, 14.4. HRMS (ESI) calcd. for $C_{20}H_{26}NS$ $[M+H]^+$ *m/z* 312.1786, found 312.1790.

6-fluoro-1,4,4-trimethyl-*N*-phenyl-1,2,3,4-tetrahydronaphthalen-1-amine (2g)

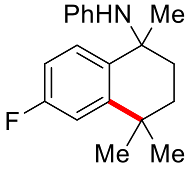

Prepared according to general procedure (C) using **1g** to provide the title compound **2g** as a yellow oil (20.1 mg, 0.71 mmol, 71%). ^{1}H NMR (400 MHz, $CDCl_3$) δ 7.53 (dd, J = 8.8, 6.2 Hz, 1H), 7.07 – 6.95 (m, 3H), 6.81 (td, J = 8.4, 2.7 Hz, 1H), 6.66 – 6.58 (m, 1H), 6.25 (dd, J = 8.5, 1.2 Hz, 2H), 3.99 (s, 1H), 2.66 (td, J = 13.9, 3.3 Hz, 1H), 1.95 (td, J = 14.1, 3.3 Hz, 1H), 1.70 (dt, J = 13.9, 3.7 Hz, 1H), 1.52 (s, 3H), 1.46 (ddd, J = 13.7, 4.2, 3.3 Hz, 1H), 1.36 (s, 3H), 1.31 (s, 3H).; ^{13}C NMR (101 MHz, $CDCl_3$) δ 161.9 (d, J = 244.4 Hz), 147.1 (d, J = 7.1 Hz), 145.7, 136.9 (d, J = 3.0 Hz), 128.9, 128.8 (d, J = 8.1 Hz), 117.4, 115.4, 113.8 (d, J = 21.2 Hz), 113.2 (d, J = 20.2 Hz), 55.6, 36.2, 34.4, 34.0, 32.0, 31.4, 23.0. HRMS (ESI) calcd. for $C_{19}H_{23}NF$ $[M+H]^+$ *m/z* 284.1815, found 284.1812.

6-chloro-1,4,4-trimethyl-*N*-phenyl-1,2,3,4-tetrahydronaphthalen-1-amine (2h)

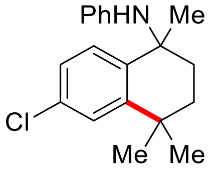

Prepared according to general procedure (C) using **1h** to provide the title compound **2h** as a

yellow solid (23.0 mg, 0.77 mmol, 77%). ^{1}H NMR (400 MHz, $CDCl_3$) δ 7.50 (d, *J* = 8.5 Hz, 1H), 7.32 (d, *J* = 2.2 Hz, 1H), 7.08 (dd, *J* = 8.5, 2.2 Hz, 1H), 7.04 – 6.96 (m, 2H), 6.62 (t, *J* = 7.3 Hz, 1H), 6.25 (dd, *J* = 8.5, 1.2 Hz, 2H), 3.99 (s, 1H), 2.67 (td, *J* = 14.0, 3.4 Hz, 1H), 1.95 (td, *J* = 14.1, 3.3 Hz, 1H), 1.69 (dt, *J* = 13.9, 3.7 Hz, 1H), 1.51 (s, 3H), 1.45 (dt, *J* = 13.7, 3.7 Hz, 1H), 1.37 (s, 3H), 1.31 (s, 3H).; ^{13}C NMR (101 MHz, $CDCl_3$) δ 146.8, 145.6, 139.9, 132.6, 128.9, 128.6, 127.0, 126.8, 117.5, 115.4, 55.6, 36.2, 34.4, 33.9, 31.9, 31.5, 29.9. HRMS (ESI) calcd. for $C_{19}H_{23}NCl$ $[M+H]^+$ *m/z* 300.1519, found 300.1511.

6-bromo-1,4,4-trimethyl-*N*-phenyl-1,2,3,4-tetrahydronaphthalen-1-amine (2i)

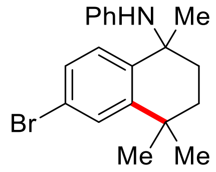

Prepared according to general procedure (C) using **1i** to provide the title compound **2i** as a white solid (23.8 mg, 0.69 mmol, 69%). ^{1}H NMR (400 MHz, $CDCl_3$) δ 7.48 (d, *J* = 2.1 Hz, 1H), 7.45 (d, *J* = 8.5 Hz, 1H), 7.23 (dd, *J* = 8.5, 2.1 Hz, 1H), 7.05 – 6.98 (m, 2H), 6.63 (t, *J* = 7.3 Hz, 1H), 6.29 – 6.23 (m, 2H), 4.00 (s, 1H), 2.68 (td, *J* = 14.0, 3.3 Hz, 1H), 1.95 (td, *J* = 14.1, 3.2 Hz, 1H), 1.70 (dt, *J* = 13.9, 3.6 Hz, 1H), 1.52 (s, 3H), 1.46 (dt, *J* = 13.6, 3.6 Hz, 1H), 1.38 (s, 3H), 1.32 (s, 3H).; ^{13}C NMR (101 MHz, $CDCl_3$) δ 147.2, 145.5, 140.4, 130.0, 129.7, 129.0, 128.9, 120.9, 117.5, 115.4, 55.6, 36.2, 34.3, 33.9, 31.9, 31.5, 29.8. HRMS (ESI) calcd. for $C_{19}H_{23}NBr$ $[M+H]^+$ *m/z* 344.1014, found 344.1011.

1,4,4-trimethyl-*N*-phenyl-6-(trifluoromethyl)-1,2,3,4-tetrahydronaphthalen-1-amine (2j)

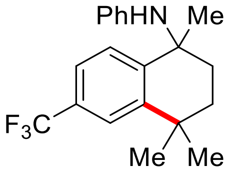

Prepared according to general procedure (C) using **1j** to provide the title compound **2j** as a colorless oil (14.0 mg, 0.42 mmol, 42%). ^{1}H NMR (400 MHz, $CDCl_3$) δ 7.69 (d, *J* = 8.3 Hz, 1H), 7.63 – 7.58 (m, 1H), 7.35 (dd, *J* = 8.3, 1.9 Hz, 1H), 7.04 – 6.96 (m, 2H), 6.67 – 6.58 (m, 1H), 6.27 – 6.19 (m, 2H), 4.04 (s, 1H), 2.72 (td, *J* = 14.0, 3.4 Hz, 1H), 1.98 (td, *J* = 14.2, 3.3 Hz, 1H), 1.73 (dt, *J* = 13.9, 3.7 Hz, 1H), 1.54 (s, 3H), 1.49 (dt, *J* = 13.7, 3.6 Hz, 1H), 1.42 (s, 3H), 1.34 (s, 3H).; ^{13}C NMR (101 MHz, $CDCl_3$) δ 145.5, 145.4, 145.4, 129.1 (q, *J* = 32.3 Hz), 129.0, 127.5, 127.3 (q, *J* = 273.7 Hz), 124.0 (q, *J* = 4.0 Hz), 123.2 (q, *J* = 4.0 Hz), 117.7, 115.4, 55.8, 36.1, 34.3, 33.9, 31.9, 31.6, 29.8. HRMS (ESI) calcd. for $C_{20}H_{23}NF_3$ $[M+H]^+$ *m/z* 334.1783, found 334.1778.

1,4,4,7-tetramethyl-*N*-phenyl-1,2,3,4-tetrahydronaphthalen-1-amine (2k)

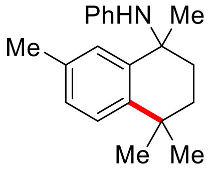

Prepared according to general procedure (C) using **1k** to provide the title compound **2k** as a colorless oil (23.4 mg, 0.84 mmol, 84%). ^{1}H NMR (400 MHz, $CDCl_3$) δ 7.44 (s, 1H), 7.30 (d, J = 8.0 Hz, 1H), 7.09 – 7.02 (m, 3H), 6.69 – 6.60 (m, 1H), 6.34 (dd, J = 8.6, 1.0 Hz, 2H), 4.02 (s, 1H), 2.71 (td, J = 13.8, 3.1 Hz, 1H), 2.29 (s, 3H), 1.96 (td, J = 13.9, 3.0 Hz, 1H), 1.77 – 1.66 (m, 1H), 1.58 (s, 3H), 1.53 – 1.43 (m, 1H), 1.40 (s, 3H), 1.34 (s, 3H).; ^{13}C NMR (101 MHz, $CDCl_3$) δ 146.1, 141.7, 141.1, 135.7, 128.8, 127.9, 127.1, 126.8, 117.2, 115.6, 55.8, 36.4, 33.9, 33.7, 32.1, 31.6, 30.2, 21.3. HRMS (ESI) calcd. for $C_{20}H_{26}N$ $[M+H]^+$ *m/z* 280.2065, found 280.2062.

7-cyclopropyl-1,4,4-trimethyl-*N*-phenyl-1,2,3,4-tetrahydronaphthalen-1-amine (2l)

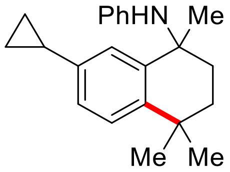

Prepared according to general procedure (C) using **1l** to provide the title compound **2l** as a colorless oil (25.3 mg, 0.83 mmol, 83%). ^{1}H NMR (400 MHz, $CDCl_3$) δ 7.36 (d, J = 2.0 Hz, 1H), 7.28 (d, J = 8.2 Hz, 1H), 7.06 – 6.99 (m, 2H), 6.90 (dd, J = 8.2, 2.0 Hz, 1H), 6.64 (t, J = 7.3 Hz, 1H), 6.32 (dd, J = 8.6, 1.0 Hz, 2H), 3.97 (s, 1H), 2.68 (td, J = 13.8, 3.2 Hz, 1H), 2.01 – 1.87 (m, 1H), 1.86 – 1.76 (m, 1H), 1.73 – 1.65 (m, 1H), 1.57 (s, 3H), 1.48 (ddd, J = 13.5, 4.2, 3.3 Hz, 1H), 1.38 (s, 3H), 1.31 (s, 3H), 0.88 (ddd, J = 8.4, 3.7, 1.8 Hz, 2H), 0.69 – 0.62 (m, 1H), 0.61 – 0.54 (m, 1H).; ^{13}C NMR (101 MHz, $CDCl_3$) δ 146.1, 141.8, 141.7, 141.1, 128.8, 126.9, 124.3, 123.7, 117.3, 115.6, 55.9, 36.3, 33.9, 33.8, 32.1, 31.6, 30.3, 15.2, 9.4, 9.1. HRMS (ESI) calcd. for $C_{22}H_{28}N$ $[M+H]^+$ *m/z* 306.2222, found 306.2213.

7-chloro-1,4,4-trimethyl-*N*-phenyl-1,2,3,4-tetrahydronaphthalen-1-amine (2m)

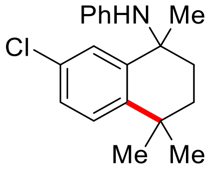

Prepared according to general procedure (C) using **1m** to provide the title compound **2m** as a yellow solid (22.4 mg, 0.75 mmol, 75%). ^{1}H NMR (400 MHz, $CDCl_3$) δ 7.58 (d, J = 2.4 Hz, 1H), 7.30 (d, J = 8.5 Hz, 1H), 7.18 (dd, J = 8.5, 2.4 Hz, 1H), 7.06 – 6.99 (m, 2H), 6.65 (tt, J = 7.2, 1.1 Hz, 1H), 6.29 (dd, J = 8.5, 0.7 Hz, 2H), 3.99 (s, 1H), 2.69 (td, J = 14.0, 3.4 Hz, 1H), 1.94 (td, J = 14.1, 3.3 Hz, 1H), 1.70 (dt, J = 13.9, 3.8 Hz, 1H), 1.53 (s, 3H), 1.45 (dt, J = 13.7, 3.7 Hz, 1H), 1.37 (s, 3H), 1.31 (s, 3H).; ^{13}C NMR (101 MHz, $CDCl_3$) δ 145.6, 143.6, 143.2, 132.2, 128.9, 128.6, 127.2, 126.7, 117.6, 115.6, 55.8, 36.1, 33.9, 33.9, 32.0, 31.5, 29.8. HRMS (ESI) calcd. for $C_{19}H_{23}NCl$ $[M+H]^+$ *m/z* 300.1519, found 300.1523.

1,4,4-trimethyl-*N*-phenyl-7-(trifluoromethyl)-1,2,3,4-tetrahydronaphthalen-1-amine (2n)

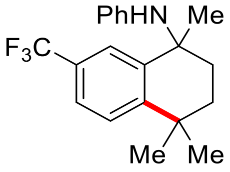

Prepared according to general procedure (C) using **1n** to provide the title compound **2n** as a colorless oil (15.0 mg, 0.45 mmol, 45%). ^{1}H NMR (400 MHz, $CDCl_3$) δ 7.90 – 7.83 (m, 1H), 7.45 (dt, *J* = 8.4, 5.2 Hz, 2H), 7.05 – 6.94 (m, 2H), 6.69 – 6.59 (m, 1H), 6.28 – 6.19 (m, 2H), 3.99 (s, 1H), 2.69 (td, *J* = 14.0, 3.4 Hz, 1H), 1.97 (ddd, *J* = 14.2, 10.9, 3.4 Hz, 1H), 1.72 (dt, *J* = 14.0, 3.7 Hz, 1H), 1.54 (s, 3H), 1.48 (ddd, *J* = 13.6, 4.0, 3.3 Hz, 1H), 1.39 (d, *J* = 5.3 Hz, 3H), 1.32 (s, 3H).; ^{13}C NMR (101 MHz, $CDCl_3$) δ 148.7, 145.5, 142.5, 128.9, 128.7 (q, *J* = 32.3 Hz), 124.4 (q, *J* = 272.7 Hz), 124.0 (q, *J* = 4.0 Hz), 123.6 (q, *J* = 4.0 Hz), 127.6, 117.9, 115.7, 55.9, 36.0, 34.4, 34.0, 31.9, 31.4, 29.8. HRMS (ESI) calcd. for $C_{20}H_{23}NF_3$ $[M+H]^+$ *m/z* 334.1783, found 334.1780.

5-methoxy-1,4,4-trimethyl-*N*-phenyl-1,2,3,4-tetrahydronaphthalen-1-amine (2o)

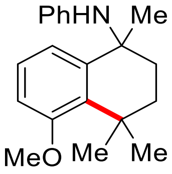

Prepared according to general procedure (C) using **1o** to provide the title compound **2o** as a white solid (17.7 mg, 0.60 mmol, 60%). ^{1}H NMR (400 MHz, $CDCl_3$) δ 7.27 (dd, *J* = 6.4, 1.5 Hz, 1H), 7.12 (t, *J* = 8.0 Hz, 1H), 7.03 – 6.96 (m, 2H), 6.77 (dd, *J* = 8.1, 1.3 Hz, 1H), 6.63 – 6.56 (m, 1H), 6.27 – 6.20 (m, 2H), 4.05 (s, 1H), 3.87 (s, 3H), 2.67 (td, *J* = 13.9, 3.2 Hz, 1H), 1.97 (td, *J* = 14.2, 3.1 Hz, 1H), 1.65 (dt, *J* = 13.8, 3.6 Hz, 1H), 1.59 (s, 3H), 1.50 (s, 3H), 1.46 – 1.38 (m, 4H).; ^{13}C NMR (101 MHz, $CDCl_3$) δ 159.1, 146.2, 143.9, 133.2, 129.1, 127.5, 120.0, 117.4, 115.5, 110.2, 56.4, 55.5, 39.3, 34.4, 33.9, 30.8, 30.2, 27.0. HRMS (ESI) calcd. for $C_{20}H_{26}NO$ $[M+H]^+$ *m/z* 296.2014, found 296.2013.

7-methoxy-1,4,4-trimethyl-*N*-phenyl-1,2,3,4-tetrahydronaphthalen-1-amine (2o')

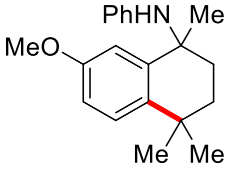

Prepared according to general procedure (C) using **1o** to provide the title compound **2o'** as a white solid (10.3 mg, 0.35 mmol, 35%). ^{1}H NMR (400 MHz, $CDCl_3$) δ 7.27 (d, *J* = 9.7 Hz, 1H), 7.11 (d, *J* = 2.7 Hz, 1H), 6.99 (t, *J* = 7.7 Hz, 2H), 6.78 (dd, *J* = 8.7, 2.7 Hz, 1H), 6.60 (t, *J* = 7.3 Hz, 1H), 6.28 (d, *J* = 8.0 Hz, 2H), 3.98 (s, 1H), 3.70 (s, 3H), 2.66 (td, *J* = 13.9, 3.2 Hz, 1H), 1.91 (td, *J* = 14.0, 3.1 Hz, 1H), 1.66 (dt, *J* = 13.9, 3.9 Hz, 1H), 1.54 (s, 3H), 1.44 (dt, *J* = 13.7, 3.7 Hz, 1H), 1.34 (s, 3H), 1.28 (s, 3H).; ^{13}C NMR (101 MHz, $CDCl_3$) δ 158.1, 145.9, 142.8, 137.0, 128.8, 128.0, 117.3, 115.6, 113.3, 111.1, 56.1, 55.3, 36.4, 33.9, 33.5, 32.3, 31.7, 30.2. HRMS (ESI) calcd. for $C_{20}H_{26}NO$ $[M+H]^+$ *m/z* 296.2014, found 296.2015.

1,4,4-trimethyl-*N*-phenyl-1,2,3,4-tetrahydrophenanthren-1-amine (2p) +
1,4,4-trimethyl-*N*-phenyl-1,2,3,4-tetrahydroanthracen-1-amine (2p')

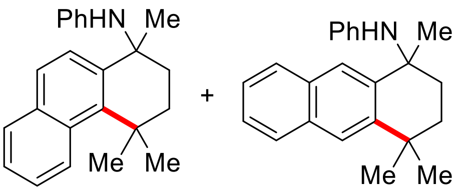

Prepared according to general procedure (C) using **1p** to provide the title compound **2p** and **2p'** as a mixture (**2p**:**2p'**=2.5:1, yellow solid, 18.9 mg, 0.60 mmol, 60%). ^{1}H NMR (400 MHz, $CDCl_3$) δ 8.54 (d, *J* = 8.7 Hz, 2.5H), 8.12 (s, 1H), 7.89 (s, 1H), 7.81 (dd, *J* = 8.3, 4.5 Hz, 5H), 7.72 (d, *J* = 8.1 Hz, 1H), 7.65 (d, *J* = 8.7 Hz, 2.5H), 7.55 – 7.33 (m, 7.5H), 6.98 (dd, *J* = 11.4, 4.6 Hz, 2.5H), 6.93 (dd, *J* = 11.2, 4.6 Hz, 5H), 6.60 (dt, *J* = 14.6, 7.3 Hz, 3.5H), 6.35 (d, *J* = 8.0 Hz, 2H), 6.23 (d, *J* = 8.0 Hz, 5H), 4.13 (s, 3.5H), 2.91 – 2.76 (m, 3.5H), 2.18 – 2.02 (m, 3.5H), 1.86 (dt, *J* = 14.0, 3.8 Hz, 2.5H), 1.82 – 1.76 (m, 8.5H), 1.74 (s, 7.5H), 1.70 (s, 7.5H), 1.64 (s, 3H), 1.59 – 1.50 (m, 6.5H), 1.44 (s, 3H).; ^{13}C NMR (101 MHz, $CDCl_3$) δ 145.9, 145.8, 143.8, 140.3, 140.2, 139.6, 134.3, 132.7, 132.3, 132.1, 129.4, 128.9, 128.9, 128.1, 127.8, 127.3, 127.3, 125.8, 125.6, 125.5, 125.5, 125.2, 124.7, 124.6, 117.3, 115.7, 115.1, 56.6, 56.3, 41.1, 36.4, 35.1, 34.9, 34.4, 33.5, 32.9, 32.8, 32.5, 30.1, 29.3, 28.1. HRMS (ESI) calcd. for $C_{23}H_{26}N$ $[M+H]^+$ *m/z* 316.2065, found 316.2067.

4'-methyl-*N*-phenyl-3',4'-dihydro-2'*H*-spiro[cyclohexane-1,1'-naphthalen]-4'-amine (2q)

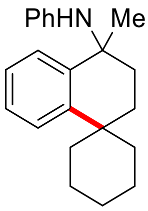

Prepared according to general procedure (C) using **1q** to provide the title compound **2q** as a colorless oil (18.9 mg, 0.62 mmol, 62%). ^{1}H NMR (400 MHz, $CDCl_3$) δ 7.59 (dd, *J* = 7.8, 1.5 Hz, 1H), 7.49 (dd, *J* = 8.1, 1.3 Hz, 1H), 7.27 – 7.21 (m, 1H), 7.13 (td, *J* = 7.5, 1.3 Hz, 1H), 7.02 – 6.94 (m, 2H), 6.60 (tt, *J* = 7.4, 1.1 Hz, 1H), 6.27 – 6.21 (m, 2H), 4.02 (s, 1H), 2.58 (td, *J* = 13.9, 3.2 Hz, 1H), 2.35 (ddd, *J* = 14.1, 4.5, 3.2 Hz, 1H), 2.09 (td, *J* = 12.6, 4.4 Hz, 1H), 1.96 – 1.90 (m, 1H), 1.78 (d, *J* = 12.2 Hz, 1H), 1.70 – 1.42 (m, 11H), 1.42 – 1.25 (m, 1H).; ^{13}C NMR (101 MHz, $CDCl_3$) δ 145.9, 145.4, 142.0, 128.8, 127.0, 126.8, 126.7, 126.4, 117.2, 115.4, 55.6, 40.2, 37.2, 37.1, 33.7, 29.5, 27.9, 26.3, 22.4, 21.8. HRMS (ESI) calcd. for $C_{22}H_{28}N$ $[M+H]^+$ *m/z* 306.2222, found 306.2221.

1-ethyl-4,4-dimethyl-*N*-phenyl-1,2,3,4-tetrahydronaphthalen-1-amine (2s)

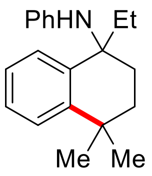

Prepared according to general procedure (C) using **1s** to provide the title compound **2s** as a yellow solid (22.0 mg, 0.79 mmol, 79%). ^{1}H NMR (400 MHz, $CDCl_3$) δ 7.51 (dd, *J* = 7.8, 1.6 Hz, 1H), 7.39 (dd, *J* = 7.9, 1.4 Hz, 1H), 7.22 (ddd, *J* = 7.9, 7.2, 1.5 Hz, 1H), 7.11 (ddd, *J* = 7.9,

7.1, 1.4 Hz, 1H), 7.03 – 6.96 (m, 2H), 6.60 (tt, *J* = 7.3, 1.1 Hz, 1H), 6.29 (ddd, *J* = 7.9, 2.3, 1.2 Hz, 2H), 4.04 (s, 1H), 2.68 – 2.48 (m, 1H), 1.98 – 1.75 (m, 3H), 1.71 – 1.58 (m, 2H), 1.39 (s, 3H), 1.34 (s, 3H), 1.02 (t, *J* = 7.5 Hz, 3H).; ^{13}C NMR (101 MHz, $CDCl_3$) δ 145.9, 144.8, 141.1, 128.8, 127.2, 127.1, 126.8, 126.0, 117.1, 115.5, 57.9, 36.7, 35.7, 34.1, 32.2, 32.1, 25.6, 8.3. HRMS (ESI) calcd. for $C_{20}H_{26}N$ $[M+H]^+$ *m/z* 280.2065, found 280.2064.

4,4-dimethyl-*N*-phenyl-1-propyl-1,2,3,4-tetrahydronaphthalen-1-amine (2t)

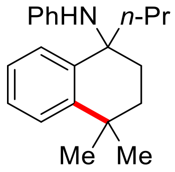

Prepared according to general procedure (C) using **1t** to provide the title compound **2t** as a yellow solid (19.6 mg, 0.67 mmol, 67%). ^{1}H NMR (400 MHz, $CDCl_3$) δ 7.53 (dd, *J* = 7.9, 1.5 Hz, 1H), 7.40 (dd, *J* = 7.9, 1.4 Hz, 1H), 7.25 – 7.20 (m, 1H), 7.15 – 7.09 (m, 1H), 7.05 – 6.96 (m, 2H), 6.61 (tt, *J* = 7.2, 1.1 Hz, 1H), 6.32 – 6.25 (m, 2H), 4.08 (s, 1H), 2.60 (dt, *J* = 13.2, 3.9 Hz, 1H), 1.93 (dt, *J* = 15.6, 3.9 Hz, 1H), 1.88 – 1.73 (m, 2H), 1.72 – 1.60 (m, 2H), 1.60 – 1.43 (m, 2H), 1.41 (s, 3H), 1.35 (s, 3H), 0.98 (t, *J* = 7.2 Hz, 3H).; ^{13}C NMR (101 MHz, $CDCl_3$) δ 145.9, 144.6, 141.3, 128.8, 127.1, 126.8, 126.0, 117.1, 115.5, 58.0, 46.8, 35.8, 34.1, 32.2, 32.1, 26.2, 17.0, 14.7. HRMS (ESI) calcd. for $C_{21}H_{28}N$ $[M+H]^+$ *m/z* 294.2222, found 294.2219.

4,4-dimethyl-*N*,1-diphenyl-1,2,3,4-tetrahydronaphthalen-1-amine (2v)

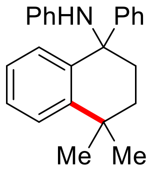

Prepared according to general procedure (C) using **1v** to provide the title compound **2v** as a white solid (29.4 mg, 0.90 mmol, 90%). ^{1}H NMR (400 MHz, $CDCl_3$) δ 7.43 (dd, *J* = 8.0, 1.3 Hz, 1H), 7.35 – 7.22 (m, 6H), 7.18 (dd, *J* = 7.9, 1.5 Hz, 1H), 7.10 – 7.00 (m, 3H), 6.63 (tt, *J* = 7.3, 1.1 Hz, 1H), 6.44 – 6.37 (m, 2H), 4.36 (s, 1H), 2.97 (ddd, *J* = 13.5, 10.4, 2.8 Hz, 1H), 2.16 (ddd, *J* = 13.8, 8.0, 2.8 Hz, 1H), 1.64 (ddd, *J* = 13.8, 8.0, 2.8 Hz, 1H), 1.44 (ddd, *J* = 13.6, 10.4, 2.8 Hz, 1H), 1.38 (s, 3H), 1.32 (s, 3H).; ^{13}C NMR (101 MHz, $CDCl_3$) δ 148.1, 146.1, 145.5, 139.7, 129.5, 128.8, 128.3, 127.8, 127.5, 127.1, 127.0, 126.2, 117.2, 115.8, 63.0, 35.1, 34.1, 32.1, 32.0, 29.2. HRMS (ESI) calcd. for $C_{24}H_{26}N$ $[M+H]^+$ *m/z* 328.2065, found 328.2063.

4,4-dimethyl-*N*-phenyl-1,2,3,4-tetrahydronaphthalen-1-amine (2w)

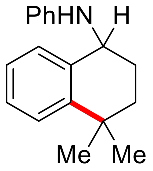

Prepared according to general procedure (C) (but at 125 °C) using **1w** to provide the title compound **2w** as a colorless oil (18.8 mg, 0.75 mmol, 75%). ^{1}H NMR (400 MHz, $CDCl_3$) δ

7.39 (ddd, *J* = 7.6, 4.7, 1.3 Hz, 2H), 7.27 (td, *J* = 7.6, 1.5 Hz, 1H), 7.24 – 7.19 (m, 2H), 7.17 (td, *J* = 7.4, 1.4 Hz, 1H), 6.76 – 6.67 (m, 3H), 4.61 (t, *J* = 5.1 Hz, 1H), 3.88 (s, 1H), 2.12 – 1.92 (m, 2H), 1.87 (ddd, *J* = 13.2, 10.2, 2.9 Hz, 1H), 1.64 (ddd, *J* = 13.6, 7.8, 3.0 Hz, 1H), 1.37 (s, 3H), 1.31 (s, 3H).; ^{13}C NMR (101 MHz, $CDCl_3$) δ 147.6, 146.4, 137.2, 129.5, 129.3, 127.7, 126.8, 126.1, 117.2, 112.9, 52.1, 35.3, 34.0, 31.8, 31.6, 25.4. HRMS (ESI) calcd. for $C_{18}H_{22}N$ $[M+H]^+$ *m/z* 252.1752, found 252.1750.

4,4-dimethyl-*N*-(4-nitrophenyl)-1,2,3,4-tetrahydronaphthalen-1-amine (2x)

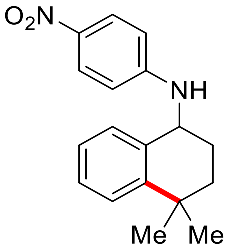

Prepared according to general procedure (C) using **1x** to provide the title compound **2x** as a yellow solid (15.1 mg, 0.51 mmol, 51%). ^{1}H NMR (400 MHz, $CDCl_3$) δ 8.19 – 8.05 (m, 2H), 7.40 (dd, *J* = 7.9, 1.3 Hz, 1H), 7.35 – 7.27 (m, 1H), 7.17 (ddd, *J* = 8.1, 7.0, 1.4 Hz, 1H), 6.67 – 6.55 (m, 2H), 4.69 (t, *J* = 5.2 Hz, 1H), 2.10 (dddd, *J* = 13.3, 10.2, 4.7, 3.0 Hz, 1H), 2.02 – 1.91 (m, 1H), 1.83 (ddd, *J* = 13.3, 10.2, 2.9 Hz, 1H), 1.68 (ddd, *J* = 13.7, 7.9, 3.0 Hz, 1H), 1.36 (s, 3H), 1.30 (s, 3H).; ^{13}C NMR (101 MHz, $CDCl_3$) δ 152.5, 146.5, 138.1, 135.1, 129.0, 128.4, 127.2, 126.8, 126.4, 111.3, 52.1, 35.1, 34.0, 31.7, 31.5, 25.3. HRMS (ESI) calcd. for $C_{18}H_{21}N_2O_2$ $[M+H]^+$ *m/z* 297.1603, found 297.1591.

***N*-(4-methoxyphenyl)-1,4,4-trimethyl-1,2,3,4-tetrahydronaphthalen-1-amine (2y)**

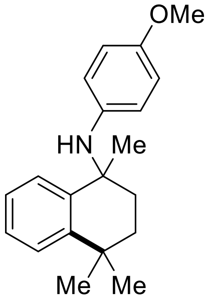

Prepared according to general procedure (C) using **1y** to provide the title compound **2y** as a colorless solid (22.4 mg, 0.69 mmol, 69%). ^{1}H NMR (400 MHz, $CDCl_3$) δ 7.64 (dd, *J* = 7.8, 1.4 Hz, 1H), 7.37 (dd, *J* = 7.8, 1.2 Hz, 1H), 7.23 (td, *J* = 7.6, 1.5 Hz, 1H), 7.19 – 7.12 (m, 1H), 6.69 – 6.57 (m, 2H), 6.31 – 6.21 (m, 2H), 3.72 (s, 1H), 3.69 (s, 3H), 2.59 (td, *J* = 13.7, 3.2 Hz, 1H), 1.94 (td, *J* = 13.8, 3.0 Hz, 1H), 1.70 (dt, *J* = 13.7, 3.6 Hz, 1H), 1.54 (s, 3H), 1.52 – 1.43 (m, 1H), 1.39 (s, 3H), 1.31 (s, 3H).; ^{13}C NMR (101 MHz, $CDCl_3$) δ 152.2, 144.8, 141.6, 139.9, 127.1, 126.8, 126.3, 117.3, 114.4, 56.1, 55.7, 36.3, 34.1, 33.7, 32.0, 31.6, 30.4. HRMS (ESI) calcd. for $C_{20}H_{26}N$ $[M+H]^+$ *m/z* 296.2014, found 296.2013.

Genaral procedure (D) for the copper-catalyzed oxidative C(sp3)-H/ C(sp2)-H cross-coupling en route to carbocyclic rings. To an oven-dried 35 mL screw-cap sealed tube

equipped with a magnetic stir bar was added $Cu(OAc)_2$ (25 mol%), Ag_2CO_3 (200 mol%), substrate **1** (0.1 mmol, 1.0 equiv) and 1,2-dichloroethane (2.0 mL) at air atmosphere. The vessel was then sealed with a Teflon screw-cap and placed into a preheated oil bath at 150 °C for 35 h. After completion, the reaction mixture was allowed to cool to room temperature, and was directly filtered through a short pad of silica gel washed with EtOAc. The filtrate was concentrated under vacuum and purified by chromatography on silica gel to obtain the corresponding product **2**.

Determination of d.r. of 2z-ah.

The d.r. of **2z-ah** were determined by ^{1}H NMR and ^{13}C NMR analysis. The spectrums showed that all these compounds gave only one set of peaks, which means only single diastereoisomer was obtained in all cases. To further confirm this result, we have synthesized the pilot product **2z** (labeled as **2z'**) from racemic cyclic ketone as shown below.

Synthesis of 1,4-dimethyl-*N*-phenyl-1,2,3,4-tetrahydronaphthalen-1-amine (2z').

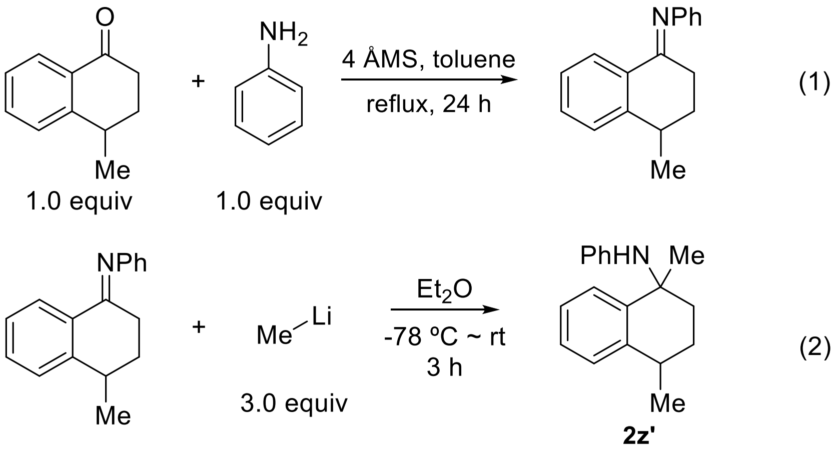

Step 1: Preparation of imine. To an oven-dried round-bottom bottle equipped with a magnetic stir bar was added 4-methyl-3,4-dihydronaphthalen-1(2*H*)-one (1.0 equiv), aniline (1.0 equiv), 4 ÅMS (0.2 g/mmol) and toluene (2.0 M). The mixture was then reflux for 24 h. After completion, it was allowed to cool to room temperature, and was directly filtered through a short pad of Celite®, washed with EtOAc. The filtrate was concentrated under vacuum and was used directly.

Step 2: Preparation of ***2z'***. Methyllithium (1.6 M in Et_2O) was added dropwise to a vigorously stirred solution of imine in Et_2O (1.0 M) at -78 °C under N_2 atmosphere and stirred at room temperature for 3 h. After completion, the reaction was quenched with water. The resulting aqueous layer was extracted with EtOAc for 3 times and the combined organic layer was washed with brine, dried over Na_2SO_4 and concentrated under vacuum. The crude was purified by column chromatography on silica gel to give **2z'** as a white solid (0.45 g, 1.8 mmol, 36% yield; 3:1 dr). ^{1}H NMR (400 MHz, $CDCl_3$) δ 7.54 – 7.52 (m, 4H), 7.31 (d, *J* = 7.7 Hz, 1H), 7.32 – 6.94 (m, 11H), 7.03 – 6.91 (m, 8H), 6.60 – 6.54 (m, 4H), 6.30 (d, *J* = 7.8 Hz, 6H), 6.22 (d, *J* = 7.8 Hz, 2H), 4.01 (m, 4H), 3.04 – 2.97 (m, 3H), 2.97 – 2.91 (m, 1H), 2.69 (td, *J* = 13.7, 3.4 Hz, 3H), 2.58 (td, *J* = 13.5, 3.1 Hz, 1H), 2.17 (tdd, *J* = 13.8, 5.6, 3.3 Hz, 3H), 1.97 (dp, *J* = 14.1, 4.6 Hz, 1H), 1.69 (dq, *J* = 13.6, 3.5 Hz, 3H), 1.64 – 1.54 (m, 2H), 1.54 (s, 3H), 1.49 (s, 9H), 1.41 (dt, *J* = 13.7, 3.7 Hz, 3H), 1.37 (d, *J* = 6.8 Hz, 3H), 1.34 (d, *J* = 7.3 Hz, 9H).; ^{13}C NMR (101

MHz, $CDCl_3$) δ 145.9, 145.7, 142.0, 141.8, 141.2, 141.1, 129.4, 128.8, 127.0, 126.9, 126.8, 126.7, 126.7, 126.6, 126.6, 117.1, 117.0, 115.3, 115.2, 55.5, 34.3, 34.0, 33.2, 33.0, 32.6, 30.7, 28.9, 27.7, 23.6, 21.7. HRMS (ESI) calcd. for $C_{17}H_{23}$ $[M+H]^+$ *m/z* 252.1752, found 252.1752.

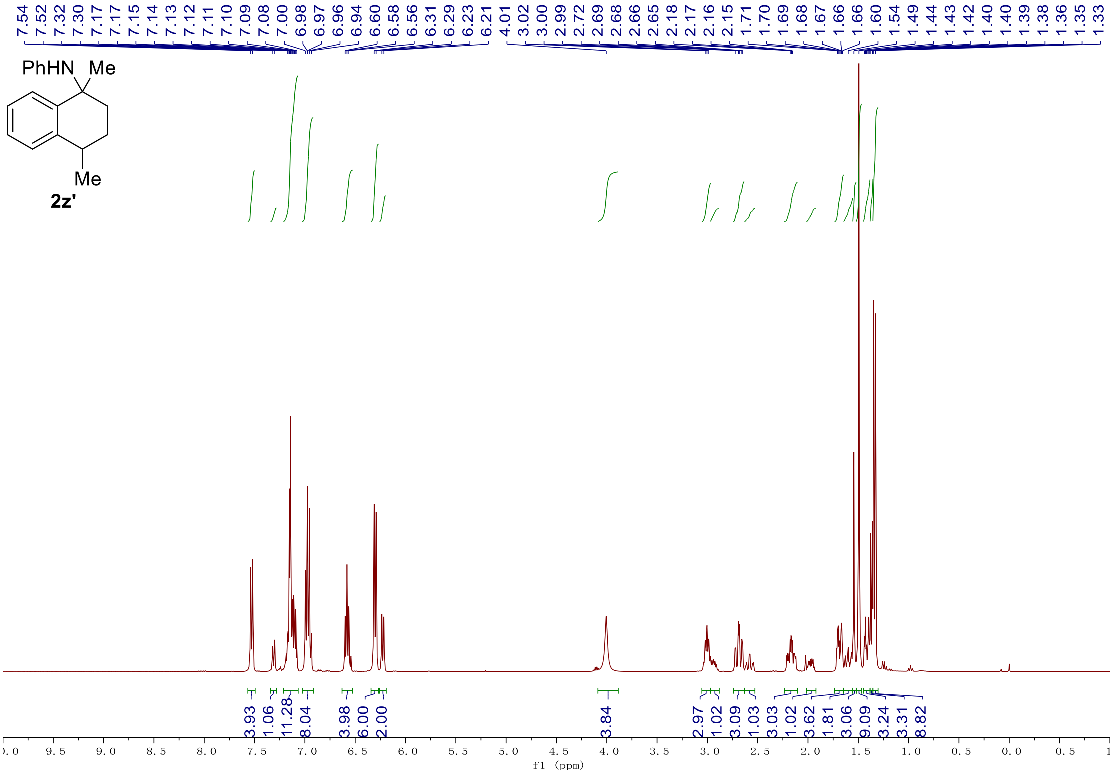

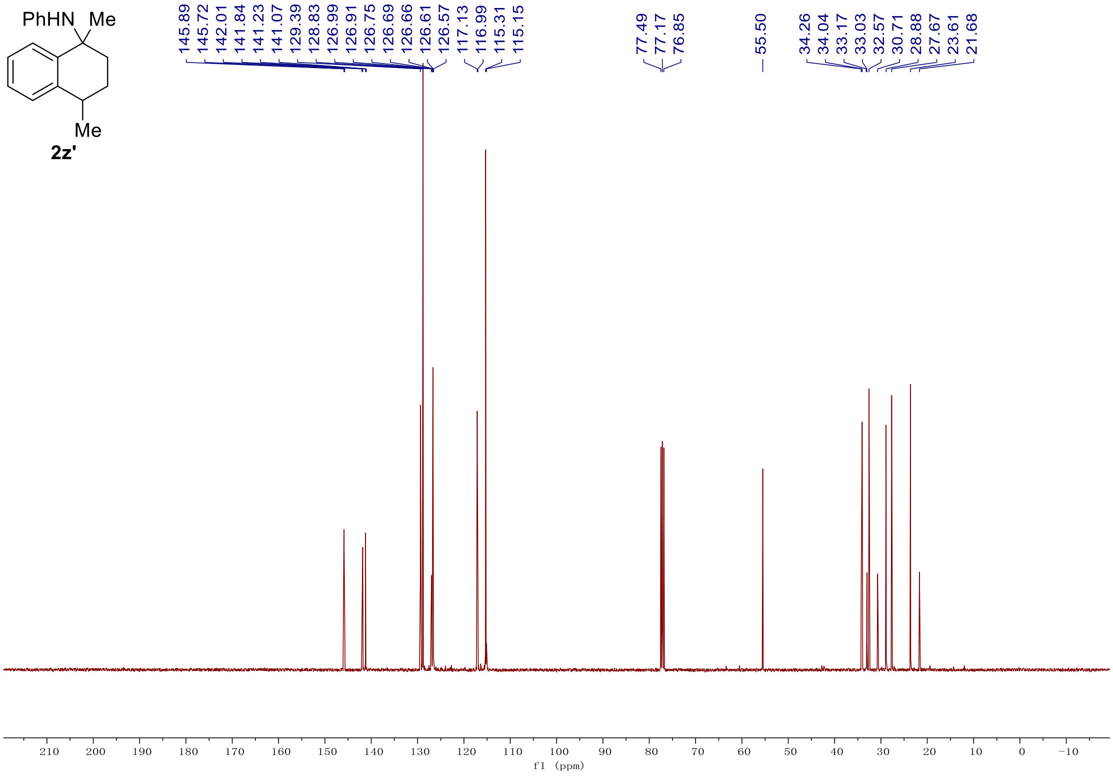

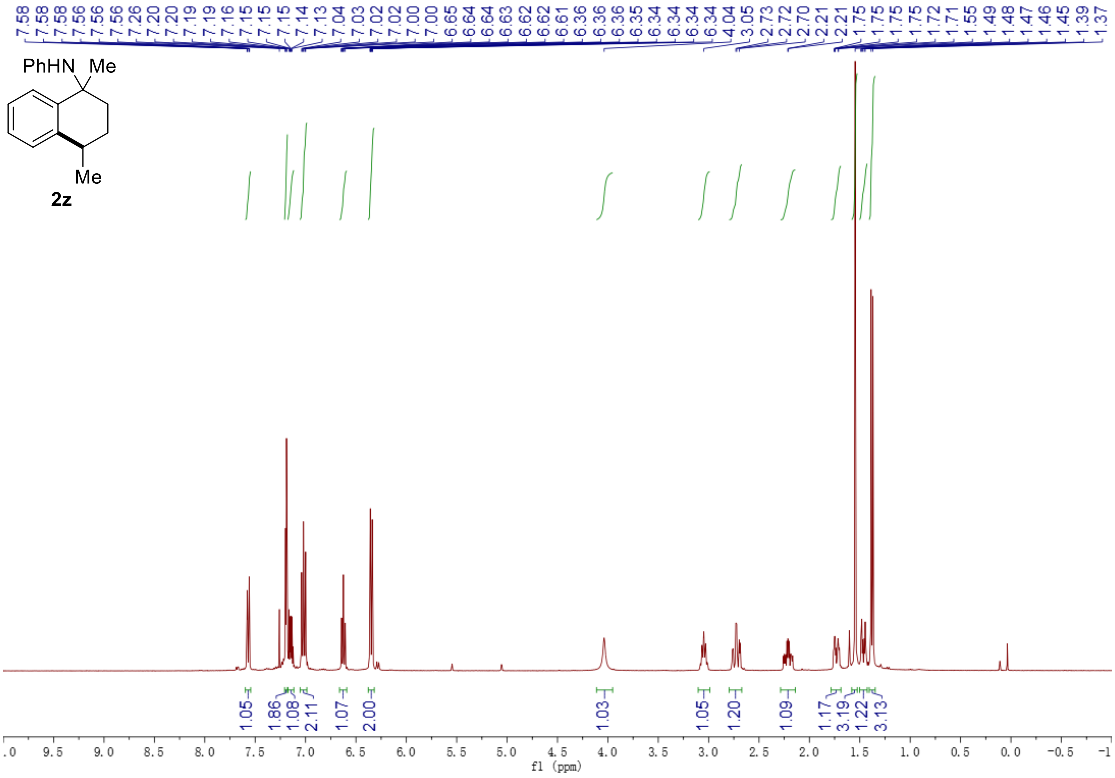

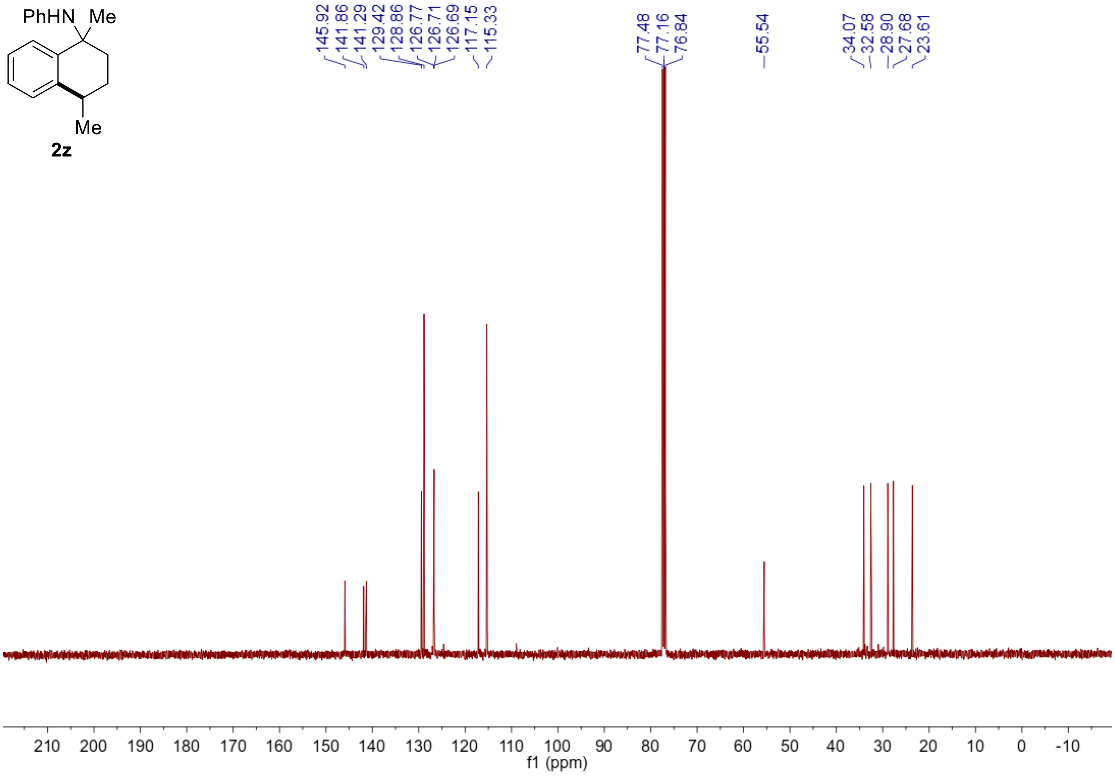

The ^{1}H NMR analysis of **2z’** showed two sets of peaks that belong to both diastereoisomers with 3:1 d.r., and the major one was consistent with **2z** made using our method. Therefore, because all the spectra of **2z-ah** synthesized from our method showed only one set of peaks, it can be inferred that only single diastereoisomers were afforded in all these cases. What’s more, the mixture of diastereoisomers of **2z’** could not be separated through the same

separation method as our general procedure (D).

To further identify the diastereomer, NOESY spectrum was conducted with product **2Z**, which indicated that the relative configuration is *cis*.

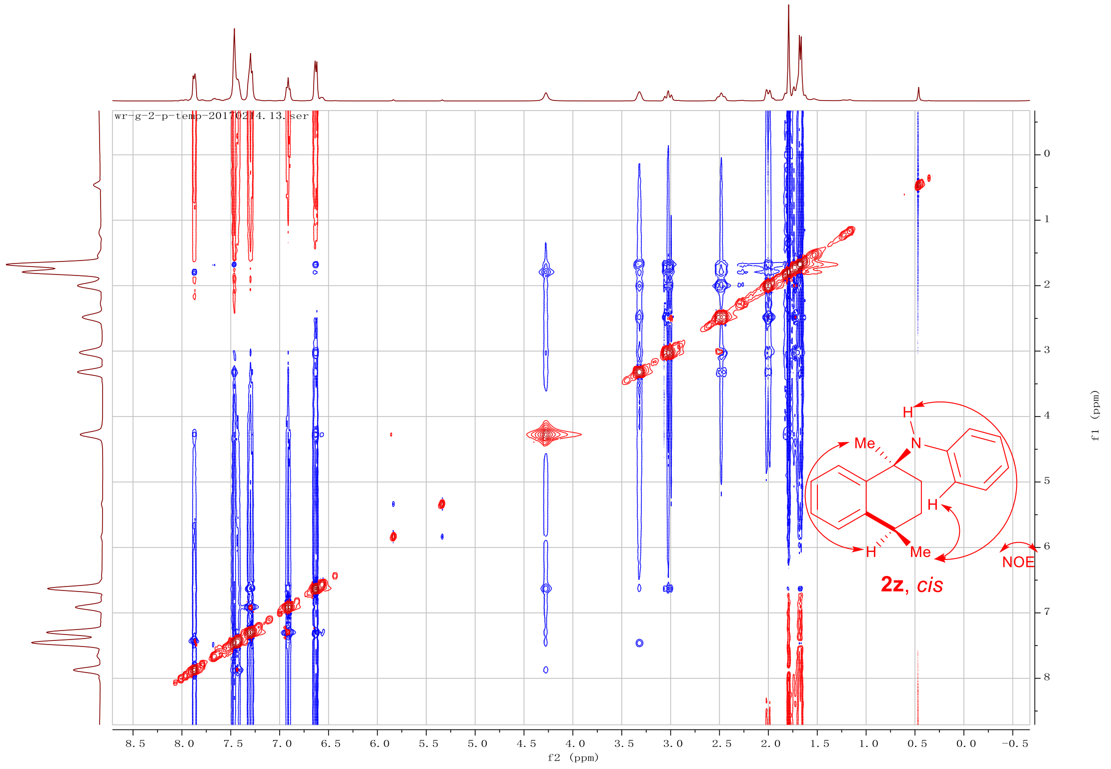

1,1,6,6-tetramethyl-2,3,5,6-tetrahydro-1*H*-phenalene (2r)

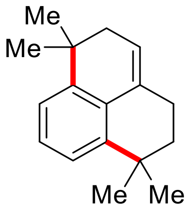

Prepared according to general procedure (D) (but at 135 °C) using **1r** to provide the title compound **2r** as a colorless oil (9.7 mg, 0.43 mmol, 43%). ^{1}H NMR (400 MHz, $CDCl_3$) δ 7.23 (dd, *J* = 6.8, 2.5 Hz, 1H), 7.19 (d, *J* = 7.4 Hz, 1H), 7.17 (d, *J* = 2.2 Hz, 1H), 5.64 (tt, *J* = 4.5, 1.6 Hz, 1H), 2.51 (ddt, *J* = 8.0, 6.1, 1.6 Hz, 2H), 2.18 (dt, *J* = 4.2, 1.8 Hz, 2H), 1.74 – 1.68 (m, 2H), 1.32 (s, 6H), 1.26 (s, 6H).; ^{13}C NMR (101 MHz, $CDCl_3$) δ 144.6, 144.0, 133.5, 129.3, 127.3, 124.0, 121.4, 120.4, 38.6, 38.4, 34.6, 34.1, 31.3, 28.9, 27.7. HRMS (ESI) calcd. for $C_{17}H_{23}$ $[M+H]^+$ *m/z* 227.1800, found 227.1796.

1-butyl-4,4-dimethyl-*N*-phenyl-1,2,3,4-tetrahydronaphthalen-1-amine (2u)

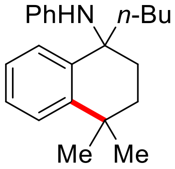

Prepared according to general procedure (D) (but at 135 °C) using **1u** to provide the title compound **2u** as a white solid (9.8 mg, 0.32 mmol, 32%). ^{1}H NMR (400 MHz, $CDCl_3$) δ 7.50 (dd, *J* = 7.9, 1.3 Hz, 1H), 7.37 (d, *J* = 7.6 Hz, 1H), 7.25 – 7.16 (m, 1H), 7.14 – 7.06 (m, 1H), 6.98 (t, *J* = 7.8 Hz, 2H), 6.59 (t, *J* = 7.3 Hz, 1H), 6.26 (d, *J* = 8.0 Hz, 2H), 4.06 (s, 1H), 2.57 (td, *J* = 14.2, 13.2, 3.8 Hz, 1H), 1.96 – 1.79 (m, 2H), 1.77 – 1.57 (m, 3H), 1.53 – 1.40 (m, 2H), 1.40 – 1.31 (m, 8H), 0.94 (t, *J* = 7.1 Hz, 3H).; ^{13}C NMR (101 MHz, $CDCl_3$) δ 145.9, 144.6, 141.5, 128.8, 127.1, 127.1, 126.8, 126.1, 117.1, 115.5, 58.0, 44.2, 35.8, 34.1, 32.2, 32.1, 26.0, 25.9, 23.3, 14.3. HRMS (ESI) calcd. for $C_{22}H_{30}N$ [M+H]$^+$ *m/z* 308.2378, found 308.2373.

1,4-dimethyl-*N*-phenyl-1,2,3,4-tetrahydronaphthalen-1-amine (2z)

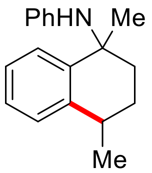

Prepared according to general procedure (D) using **1z** to provide the title compound **2z** as a colorless oil (15.5 mg, 0.62 mmol, 62% yield; single diastereoisomer). ^{1}H NMR (400 MHz, $CDCl_3$) δ 7.57 (dt, *J* = 7.6, 1.1 Hz, 1H), 7.19 (dd, *J* = 4.8, 1.2 Hz, 2H), 7.18 – 7.10 (m, 1H), 7.06 – 6.99 (m, 2H), 6.62 (tt, *J* = 7.3, 1.1 Hz, 1H), 6.38 – 6.31 (m, 2H), 4.04 (s, 1H), 3.10 – 2.99 (m, 1H), 2.73 (td, *J* = 13.8, 3.5 Hz, 1H), 2.21 (tdd, *J* = 13.8, 5.8, 3.3 Hz, 1H), 1.73 (dddd, *J* = 13.7, 4.3, 3.4, 2.2 Hz, 1H), 1.55 (s, 3H), 1.46 (dt, *J* = 13.5, 3.8 Hz, 1H), 1.38 (d, *J* = 7.2 Hz, 3H).; ^{13}C NMR (101 MHz, $CDCl_3$) δ 145.9, 141.9, 141.3, 129.4, 128.9, 126.8, 126.7, 126.7, 117.2, 115.4, 55.6, 34.1, 32.6, 29.0, 27.7, 23.6. HRMS (ESI) calcd. for $C_{18}H_{22}N$ [M+H]$^+$ *m/z* 252.1752, found 252.1751.

1,4,6-trimethyl-*N*-phenyl-1,2,3,4-tetrahydronaphthalen-1-amine (2aa)

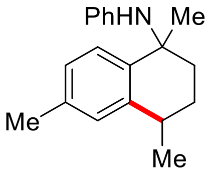

Prepared according to general procedure (D) using **1aa** to provide the title compound **2aa** as a colorless oil (11.1 mg, 0.42 mmol, 42% yield; single diastereoisomer). ^{1}H NMR (400 MHz, $CDCl_3$) δ 7.43 (d, *J* = 7.9 Hz, 1H), 7.04 – 6.93 (m, 4H), 6.61 (tt, *J* = 7.3, 1.1 Hz, 1H), 6.37 – 6.33 (m, 2H), 4.01 (s, 1H), 3.05 – 2.93 (m, 1H), 2.69 (td, *J* = 13.7, 3.5 Hz, 1H), 2.33 (s, 3H), 2.18 (tdd, *J* = 13.9, 5.8, 3.3 Hz, 1H), 1.70 (dddd, *J* = 13.7, 4.3, 3.4, 2.2 Hz, 1H), 1.52 (s, 3H), 1.47 – 1.40 (m, 1H), 1.35 (d, *J* = 7.3 Hz, 3H).; ^{13}C NMR (101 MHz, $CDCl_3$) δ 146.0, 141.1, 138.9, 136.1, 129.9, 128.8, 127.7, 126.7, 117.1, 115.4, 55.4, 34.1, 32.6, 29.0, 27.8, 23.6, 21.2. HRMS (ESI) calcd. for $C_{19}H_{24}N$ [M+H]$^+$ *m/z* 266.1909, found 266.1912.

1,4-dimethyl-*N*,6-diphenyl-1,2,3,4-tetrahydronaphthalen-1-amine (2ab)

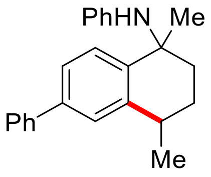

Prepared according to general procedure (D) using **1ab** to provide the title compound **2ab** as a colorless oil (15.0 mg, 0.46 mmol, 46% yield; single diastereoisomer). ^{1}H NMR (400 MHz, $CDCl_3$) δ 7.64 – 7.58 (m, 3H), 7.46 – 7.29 (m, 5H), 7.05 – 6.98 (m, 2H), 6.61 (tt, *J* = 7.3, 1.1 Hz, 1H), 6.40 – 6.34 (m, 2H), 4.05 (s, 1H), 3.17 – 3.01 (m, 1H), 2.72 (td, *J* = 13.8, 3.5 Hz, 1H), 2.22 (tdd, *J* = 13.8, 5.8, 3.3 Hz, 1H), 1.74 (dtd, *J* = 13.7, 3.9, 2.2 Hz, 1H), 1.56 (s, 3H), 1.46 (dt, *J* = 13.6, 3.7 Hz, 1H), 1.39 (d, *J* = 7.3 Hz, 3H).; ^{13}C NMR (101 MHz, $CDCl_3$) δ 145.9, 141.7, 141.1, 141.1, 139.4, 128.9, 128.8, 128.1, 127.3, 127.2, 127.1, 125.6, 117.3, 115.4, 55.5, 34.0, 32.8, 28.9, 27.8, 23.7. HRMS (ESI) calcd. for $C_{24}H_{26}N$ $[M+H]^+$ *m/z* 328.2065, found 328.2062.

6-fluoro-1,4-dimethyl-*N*-phenyl-1,2,3,4-tetrahydronaphthalen-1-amine (2ac)

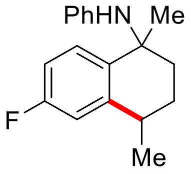

Prepared according to general procedure (D) using **1ac** to provide the title compound **2ac** as a yellow oil (12.1 mg, 0.45 mmol, 45% yield; single diastereoisomer). ^{1}H NMR (400 MHz, $CDCl_3$) δ 7.52 (dd, *J* = 8.7, 6.0 Hz, 1H), 7.05 – 6.98 (m, 2H), 6.90 – 6.79 (m, 2H), 6.63 (tt, *J* = 7.3, 1.1 Hz, 1H), 6.36 – 6.29 (m, 2H), 3.99 (s, 1H), 3.06 – 2.94 (m, 1H), 2.68 (td, *J* = 13.7, 3.5 Hz, 1H), 2.18 (tdd, *J* = 13.8, 5.8, 3.3 Hz, 1H), 1.77 – 1.67 (m, 1H), 1.51 (d, *J* = 0.8 Hz, 3H), 1.44 (dddd, *J* = 13.7, 4.3, 3.4, 0.7 Hz, 1H), 1.36 (d, *J* = 7.3 Hz, 3H).; ^{13}C NMR (101 MHz, $CDCl_3$) δ 161.6 (d, *J* = 245.4 Hz), 145.8, 143.6 (d, *J* = 7.1 Hz), 137.6 (d, *J* = 3.0 Hz), 128.9, 128.8 (d, *J* = 8.1 Hz), 117.4, 115.4, 115.3 (d, *J* = 20.2 Hz), 114.0 (d, *J* = 21.2 Hz), 55.3, 34.1, 32.9, 32.9, 28.9, 27.7, 23.4. HRMS (ESI) calcd. for $C_{18}H_{21}NF$ $[M+H]^+$ *m/z* 270.1658, found 270.1655.

6-chloro-1,4-dimethyl-*N*-phenyl-1,2,3,4-tetrahydronaphthalen-1-amine (2ad)

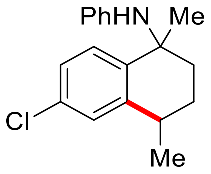

Prepared according to general procedure (D) using **1ad** to provide the title compound **2ad** as a colorless oil (15.1 mg, 0.53 mmol, 53% yield; single diastereoisomer). ^{1}H NMR (400 MHz, $CDCl_3$) δ 7.48 (d, *J* = 8.4 Hz, 1H), 7.15 (d, *J* = 2.3 Hz, 1H), 7.08 (dd, *J* = 8.4, 2.3 Hz, 1H), 7.04 – 6.97 (m, 2H), 6.62 (tt, *J* = 7.2, 1.1 Hz, 1H), 6.34 – 6.26 (m, 2H), 3.98 (s, 1H), 2.99 (p, *J* = 6.7 Hz, 1H), 2.67 (td, *J* = 13.8, 3.5 Hz, 1H), 2.17 (tdd, *J* = 13.9, 5.7, 3.4 Hz, 1H), 1.78 – 1.66 (m, 1H), 1.49 (s, 3H), 1.42 (dt, *J* = 13.6, 3.8 Hz, 1H), 1.35 (d, *J* = 7.3 Hz, 3H).; ^{13}C NMR (101 MHz, $CDCl_3$) δ 145.6, 143.3, 140.5, 132.2, 129.2, 128.9, 128.5, 127.0, 117.5, 115.4, 55.3, 34.1, 32.6, 28.7, 27.6, 23.5. HRMS (ESI) calcd. for $C_{18}H_{21}NCl$ $[M+H]^+$ *m/z* 286.1363, found 286.1360.

1,4-dimethyl-*N*-phenyl-6-(trifluoromethyl)-1,2,3,4-tetrahydronaphthalen-1-amine (2ae)

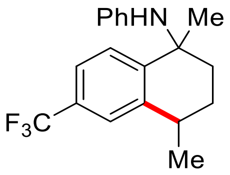

Prepared according to general procedure (D) using **1ae** to provide the title compound **2ae** as a yellow oil (14.0 mg, 0.44 mmol, 44% yield; single diastereoisomer). ^{1}H NMR (400 MHz, $CDCl_3$) δ 7.66 (d, *J* = 8.2 Hz, 1H), 7.42 (d, *J* = 1.9 Hz, 1H), 7.35 (dd, *J* = 8.2, 1.9 Hz, 1H), 7.05 – 6.96 (m, 2H), 6.67 – 6.58 (m, 1H), 6.32 – 6.24 (m, 2H), 4.04 (s, 1H), 3.08 (p, *J* = 6.7 Hz, 1H), 2.72 (td, *J* = 13.9, 3.5 Hz, 1H), 2.21 (tdd, *J* = 13.9, 5.8, 3.4 Hz, 1H), 1.74 (dtd, *J* = 13.9, 3.8, 2.1 Hz, 1H), 1.51 (s, 3H), 1.46 (dt, *J* = 13.6, 3.7 Hz, 1H), 1.37 (d, *J* = 7.3 Hz, 3H).; ^{13}C NMR (101 MHz, $CDCl_3$) δ 146.1, 145.4, 142.0, 129.0, 128.9 (q, *J* = 32.3 Hz), 127.4, 126.4 (q, *J* = 4.0 Hz), 124.5 (q, *J* = 272.7 Hz), 123.5 (q, *J* = 4.0 Hz), 117.6, 115.3, 55.5, 34.1, 32.6, 28.5, 27.5, 23.6. HRMS (ESI) calcd. for $C_{19}H_{21}NF_3$ $[M+H]^+$ *m/z* 320.1626, found 320.1621.

1,4-dimethyl-*N*-phenyl-7-(trifluoromethyl)-1,2,3,4-tetrahydronaphthalen-1-amine (2af)

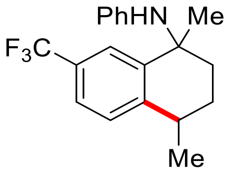

Prepared according to general procedure (D) using **1af** to provide the title compound **2af** as a yellow oil (14.3 mg, 0.45 mmol, 45% yield; single diastereoisomer). ^{1}H NMR (400 MHz, $CDCl_3$) δ 7.84 (d, *J* = 1.9 Hz, 1H), 7.41 (dd, *J* = 8.2, 2.0 Hz, 1H), 7.27 (d, *J* = 8.0 Hz, 1H), 7.04 – 6.98 (m, 2H), 6.64 (tt, *J* = 7.4, 1.1 Hz, 1H), 6.33 – 6.27 (m, 2H), 3.98 (s, 1H), 3.07 (p, *J* = 6.8 Hz, 1H), 2.70 (td, *J* = 13.8, 3.5 Hz, 1H), 2.19 (tdd, *J* = 13.9, 5.7, 3.4 Hz, 1H), 1.78 – 1.69 (m, 1H), 1.52 (d, *J* = 0.7 Hz, 3H), 1.48 – 1.40 (m, 1H), 1.35 (d, *J* = 7.3 Hz, 3H).; ^{13}C NMR (101 MHz, $CDCl_3$) δ 145.6, 145.3, 143.1, 130.1, 129.0 (q, *J* = 32.3 Hz), 128.9, 124.5 (q, *J* = 273.7 Hz), 124.0 (q, *J* = 4.0 Hz), 123.4 (q, *J* = 4.0 Hz), 117.8, 115.7, 55.6, 34.2, 32.7, 28.6, 27.4, 23.4. HRMS (ESI) calcd. for $C_{19}H_{21}NF_3$ $[M+H]^+$ *m/z* 320.1626, found 320.1629.

1-methyl-4-pentyl-*N*-phenyl-1,2,3,4-tetrahydronaphthalen-1-amine (2ag)

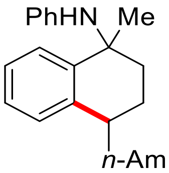

Prepared according to general procedure (D) using **1ag** to provide the title compound **2ag** as a colorless oil (12.3 mg, 0.40 mmol, 40% yield; single diastereoisomer). ^{1}H NMR (400 MHz, $CDCl_3$) δ 7.54 (d, *J* = 7.5 Hz, 1H), 7.22 – 7.09 (m, 3H), 6.99 (t, *J* = 7.7 Hz, 2H), 6.60 (t, *J* = 7.3 Hz, 1H), 6.32 (d, *J* = 8.1 Hz, 2H), 4.03 (s, 1H), 2.78 (dt, *J* = 10.4, 5.1 Hz, 1H), 2.64 (td, *J* = 13.9, 3.3 Hz, 1H), 2.13 – 1.98 (m, 1H), 1.95 – 1.83 (m, 1H), 1.82 – 1.60 (m, 2H), 1.52 (s, 3H), 1.46 –

1.24 (m, 7H), 0.91 (t, J = 6.6 Hz, 3H).; ^{13}C NMR (101 MHz, $CDCl_3$) δ 145.9, 141.9, 141.1, 129.8, 128.8, 126.8, 126.7, 126.5, 117.1, 115.3, 55.6, 38.1, 36.9, 34.3, 32.1, 29.0, 28.0, 23.9, 22.8, 14.3. HRMS (ESI) calcd. for $C_{22}H_{29}NNa$ $[M+Na]^+$ *m/z* 330.2198, found 330.2202.

9-methyl-*N*-phenyl-1,2,3,4,4a,9,10,10a-octahydrophenanthren-9-amine (2ah)

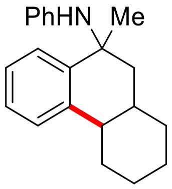

Prepared according to general procedure (D) using **1ah** to provide the title compound **2ah** as a colorless oil (10.5 mg, 0.36 mmol, 36% yield; single diastereoisomer). ^{1}H NMR (400 MHz, $CDCl_3$) δ 7.58 – 7.54 (m, 1H), 7.38 – 7.34 (m, 1H), 7.29 – 7.18 (m, 2H), 7.15 – 7.08 (m, 2H), 6.71 (tt, J = 7.3, 1.1 Hz, 1H), 6.58 – 6.53 (m, 2H), 3.95 (s, 1H), 2.58 – 2.48 (m, 1H), 2.47 – 2.38 (m, 1H), 2.33 – 2.20 (m, 1H), 2.01 – 1.89 (m, 1H), 1.81 – 1.68 (m, 2H), 1.62 (s, 3H), 1.55 – 1.38 (m, 3H), 1.37 – 1.22 (m, 2H), 1.20 – 1.06 (m, 1H).; ^{13}C NMR (101 MHz, $CDCl_3$) δ 145.9, 143.3, 140.4, 128.9, 126.7, 126.5, 124.8, 117.8, 116.6, 54.9, 42.4, 42.4, 37.2, 34.1, 33.3, 30.5, 26.6, 26.0. HRMS (ESI) calcd. for $C_{21}H_{26}N$ $[M+H]^+$ *m/z* 292.2065, found 292.2064.

Substituent Group Effects on Nitrogen.

To test the substituent group effects on nitrogen, several substrates were synthesized and subjected to the standard conditions. The results are showed in Fig. S1 as below:

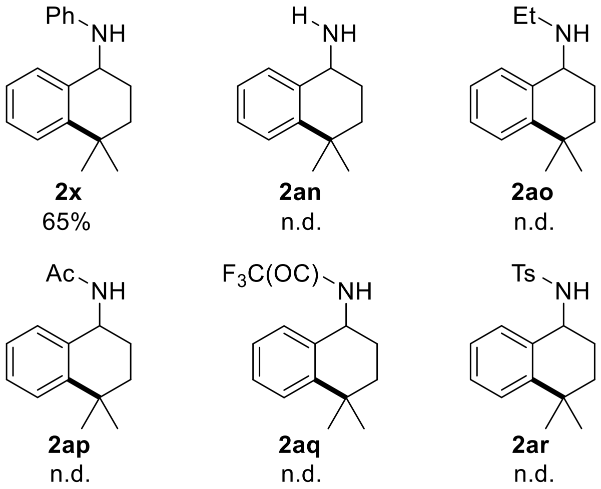

Figure S1 | Effects of Different Substituent Group on Nitrogen.[a]

[a]Reaction conditions: Reactions performed on the substrate (0.1 mmol),$Cu(OAc)_2$ (20 mol%) and Ag_2CO_3 (1.5 equiv) in 1,2-DCE (2.0 mL) at 135 °C for 25 h; n.r., no reaction; n.d., not detected.

IV. Mechanism Details.

Table S5 | Radical Inhibitor Effect

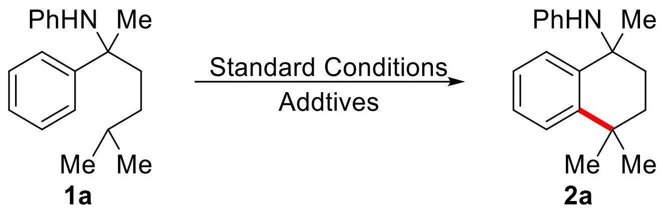

entry	Addtives	yield[b]
1	TEMPO (1.0 eq.)	trace
2	TEMPO (0.5 eq.)	trace
3	TEMPO (0.3 eq.)	10%
4	Galvinoxyl (1.0 eq.)	0
5	Galvinoxyl (0.5 eq.)	0
6	Galvinoxyl (0.3 eq.)	10%
7	BHT (1.0 eq.)	0
8	BHT (0.5 eq.)	0
9	BHT (0.3 eq.)	20%

[a]Determined by TLC and isolating.

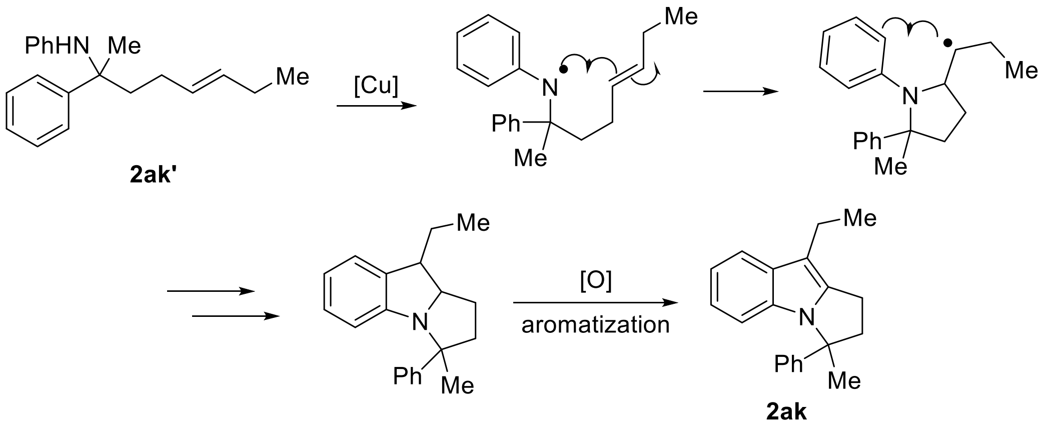

Figure S2 | Mechanism supplementary. A pathway from **2ak' to 2ak**.

(*E*)-2-(but-1-en-1-yl)-1,5-diphenylpyrrolidine (2aj-1)

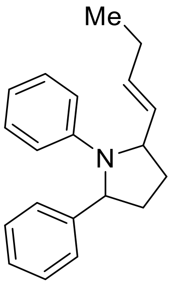

Prepared according to general procedure (C) (but at 150 °C for 9 h) using **1aj** to provide the title compound **2aj-1** as a colorless oil (7.8 mg, 0.28 mmol, 14%). ^{1}H NMR (400 MHz, $CDCl_3$) δ

7.30 – 7.22 (m, 2H), 7.22 – 7.15 (m, 1H), 7.15 – 7.11 (m, 2H), 7.10 – 7.02 (m, 2H), 6.55 (t, *J* = 7.3 Hz, 1H), 6.50 – 6.42 (m, 2H), 5.59 (ddd, *J* = 15.5, 6.9, 5.9 Hz, 1H), 5.42 (ddt, *J* = 15.3, 6.0, 1.5 Hz, 1H), 4.92 (d, *J* = 8.3 Hz, 1H), 4.56 (t, *J* = 7.0 Hz, 1H), 2.54 (dddd, *J* = 14.4, 12.1, 8.4, 6.3 Hz, 1H), 2.34 – 2.17 (m, 1H), 2.11 – 1.96 (m, 2H), 1.79 (dd, *J* = 12.0, 6.2 Hz, 1H), 1.69 (dd, *J* = 12.1, 6.2 Hz, 1H), 0.96 (t, *J* = 7.4 Hz, 3H).; ^{13}C NMR (101 MHz, $CDCl_3$) δ 145.5, 144.3, 133.3, 129.3, 128.7, 128.5, 126.6, 126.2, 115.2, 113.9, 62.4, 60.6, 33.1, 30.0, 25.4, 13.9. HRMS (ESI) calcd. for $C_{20}H_{24}N$ $[M+H]^+$ *m/z* 278.1909, found 278.1912.

(*E*)-2-(buta-1,3-dien-1-yl)-1,5-diphenylpyrrolidine (2aj-2)

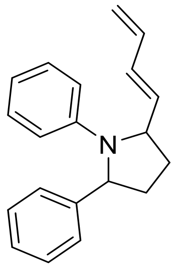

Prepared according to general procedure (C) (but at 150 °C for 9 h) using **1aj** to provide the title compound **2aj-2** as a colorless oil (9.9 mg, 0.36 mmol, 18%). ^{1}H NMR (400 MHz, $CDCl_3$) δ 7.31 – 7.24 (m, 2H), 7.23 – 7.17 (m, 1H), 7.16 – 7.12 (m, 2H), 7.12 – 7.05 (m, 2H), 6.58 (tt, *J* = 7.3, 1.1 Hz, 1H), 6.52 – 6.42 (m, 2H), 6.42 – 6.26 (m, 1H), 6.14 (ddq, *J* = 15.3, 10.4, 0.8 Hz, 1H), 5.76 (ddd, *J* = 15.2, 5.9, 0.7 Hz, 1H), 5.14 (dd, *J* = 16.9, 1.7 Hz, 1H), 5.04 (dd, *J* = 10.0, 1.7 Hz, 1H), 4.98 (d, *J* = 8.2 Hz, 1H), 4.73 – 4.60 (m, 1H), 2.54 (dddd, *J* = 13.6, 12.0, 8.3, 6.3 Hz, 1H), 2.31 (dddd, *J* = 14.0, 12.2, 8.1, 6.2 Hz, 1H), 1.83 (ddt, *J* = 11.9, 6.1, 1.0 Hz, 1H), 1.76 (ddt, *J* = 12.2, 6.3, 1.0 Hz, 1H).; ^{13}C NMR (101 MHz, $CDCl_3$) δ 145.2, 144.0, 136.6, 135.4, 131.3, 128.8, 128.6, 126.7, 126.2, 116.7, 115.6, 113.8, 62.5, 60.3, 33.2, 29.8. HRMS (ESI) calcd. for $C_{20}H_{22}N$ $[M+H]^+$ *m/z* 276.1752, found 276.1748.

9-ethyl-3-methyl-3-phenyl-2,3-dihydro-1*H*-pyrrolo[1,2-a]indole (2ak)

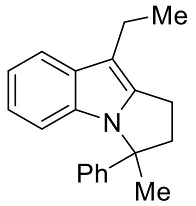

Prepared according to general procedure (C) (but at 150 °C for 9 h) using **1ak** to provide the title compound **2ak** as a colorless oil (9.9 mg, 0.36 mmol, 18%). ^{1}H NMR (400 MHz, $CDCl_3$) δ 7.56 (dt, *J* = 7.9, 1.0 Hz, 1H), 7.31 – 7.19 (m, 3H), 7.08 – 6.99 (m, 3H), 6.98 – 6.90 (m, 2H), 3.10 – 2.86 (m, 2H), 2.78 (q, *J* = 7.6 Hz, 2H), 2.67 (t, *J* = 7.2 Hz, 2H), 2.00 (s, 3H), 1.32 (t, *J* = 7.6 Hz, 3H).; ^{13}C NMR (101 MHz, $CDCl_3$) δ 145.5, 140.7, 132.9, 131.8, 128.6, 127.1, 125.4, 119.9, 118.7, 118.5, 110.5, 107.8, 65.7, 47.2, 25.8, 22.5, 18.2, 15.1. HRMS (ESI) calcd. for $C_{20}H_{22}N$ $[M+H]^+$ *m/z* 276.1752, found 276.1750.

***N*-(5-methyl-2-phenylhex-5-en-2-yl)aniline (3a)**

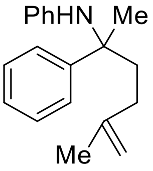

Colorless oil (13.0 mg, 0.49 mmol, 49%). ^{1}H NMR (400 MHz, $CDCl_3$) δ 7.53 – 7.45 (m, 2H), 7.39 – 7.29 (m, 2H), 7.28 – 7.20 (m, 1H), 7.07 – 6.95 (m, 2H), 6.62 (tt, *J* = 7.3, 1.1 Hz, 1H), 6.39 – 6.31 (m, 2H), 4.84 – 4.59 (m, 2H), 4.05 (s, 1H), 2.17 – 1.88 (m, 4H), 1.67 (s, 3H), 1.65 (t, J = 1.1 Hz, 3H).; ^{13}C NMR (101 MHz, $CDCl_3$) δ 146.5, 146.1, 145.8, 128.8, 128.6, 126.5, 126.3, 117.3, 115.5, 110.0, 58.5, 42.3, 32.1, 26.1, 22.8. HRMS (ESI) calcd. for $C_{19}H_{24}N$ $[M+H]^+$ *m/z* 266.1909, found 266.1911.

V. References.

[1] WO2009/42694.AI, **2009**.

[2] Guzen, K. P.; Guarezemini, A. S.; Órfão, A. T. G.; Cella, R.; Pereiraa, C. M. P.; Stefani, H. A. *Tetrahedron Lett.*, **2007**, *48*, 1845.

[3] Patel, J. P.; Li, A.-H.; Dong, H.; Korlipara, V. L.; Mulvihill, M. J. *Tetrahedron Lett.*, **2009**, *50*, 5975.

[4] Fifera, N. L.; White, J. M. *Org. Biomol. Chem.*, **2005**, *3*, 1776.

[5] Kataoka, F.; Nishida, S.; Tsuji, T.; Murakami, M. *J. Am. Chem. Soc.*, **1981**, *103*, 6878.

[6] Wagner, P. J.; Liu, K. C.; Noguchi, Y. *J. Am. Chem. Soc.*, **1981**, *103*, 3837.

[7] WO2004/69256.A1, **2004**.

VI. Spectroscopic Data (NMR Spectrum).

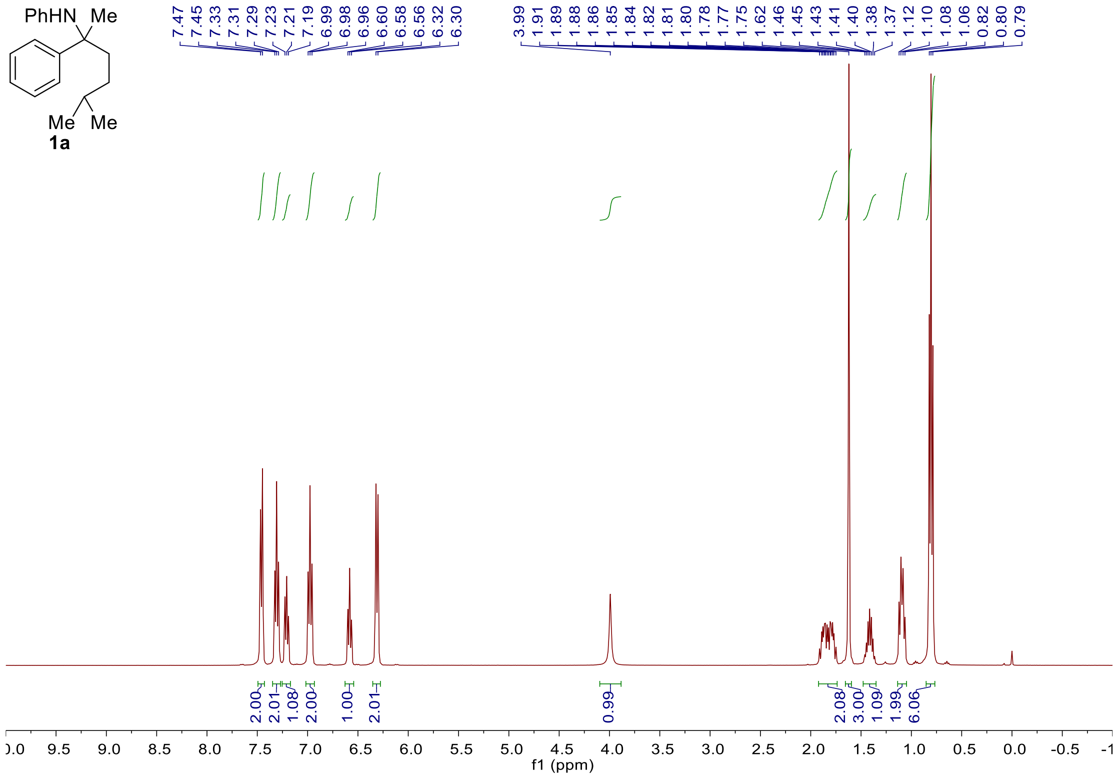

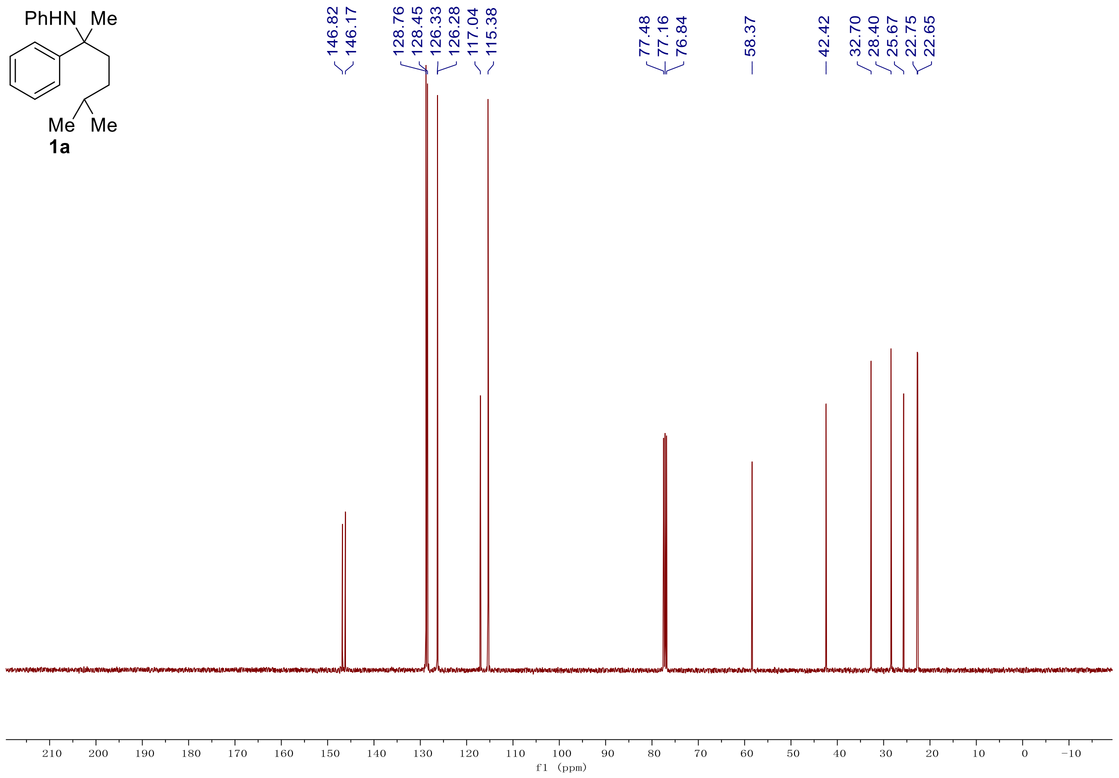

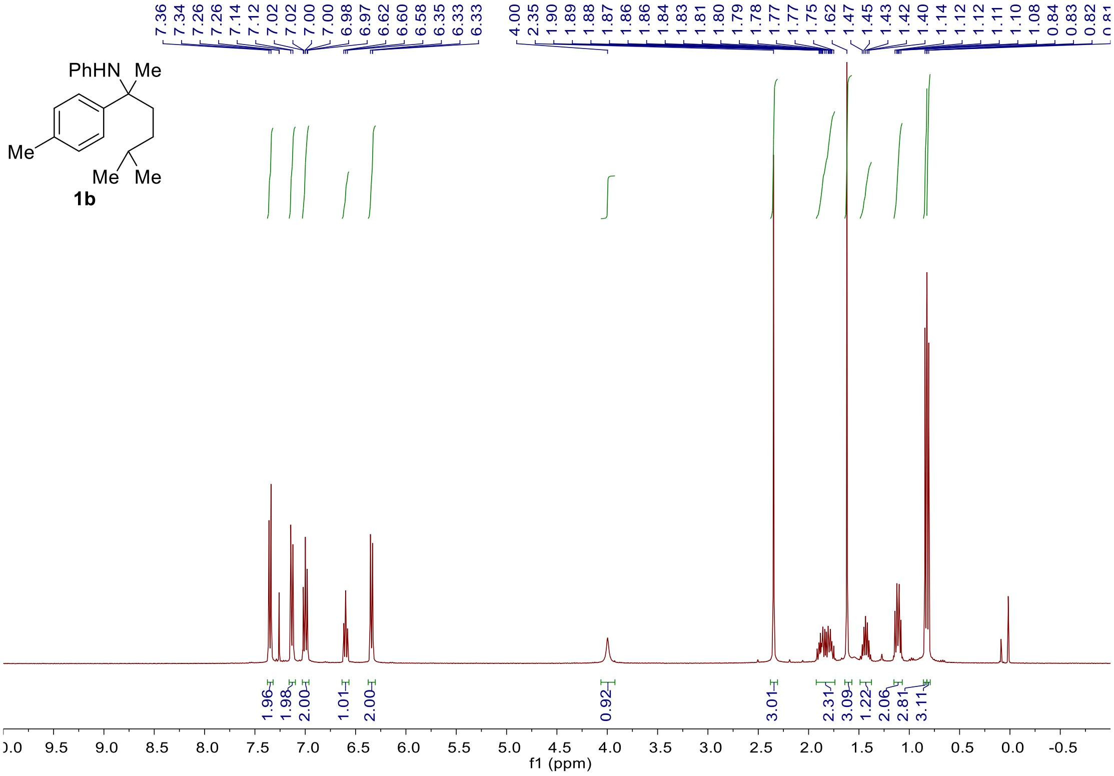
PhHN Me
Me
Me Me
1b
7.36 7.34 7.26 7.26 7.14 7.12 7.02 7.02 7.00 7.00 6.98 6.97 6.62 6.60 6.58 6.35 6.33 6.33
4.00 2.35 1.90 1.89 1.88 1.87 1.86 1.86 1.84 1.83 1.81 1.80 1.79 1.78 1.77 1.77 1.75 1.62 1.47 1.45 1.43 1.42 1.40 1.14 1.12 1.12 1.11 1.10 1.08 0.84 0.83 0.82
1.96 1.98 2.00 1.01 2.00 0.92 3.01 2.31 3.09 1.22 2.06 2.81 3.11
f1 (ppm)

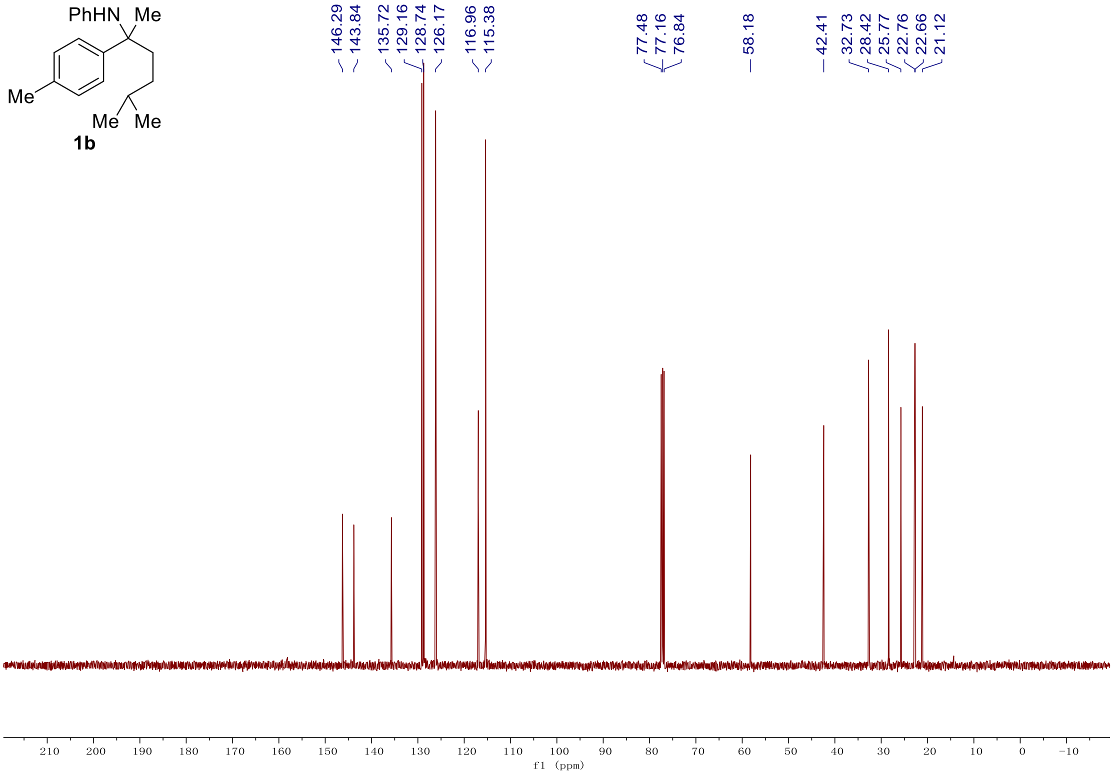
PhHN Me
Me
Me Me
1b
146.29 143.84 135.72 129.16 128.74 126.17 116.96 115.38
77.48 77.16 76.84
58.18
42.41 32.73 28.42 25.77 22.76 22.66 21.12
f1 (ppm)

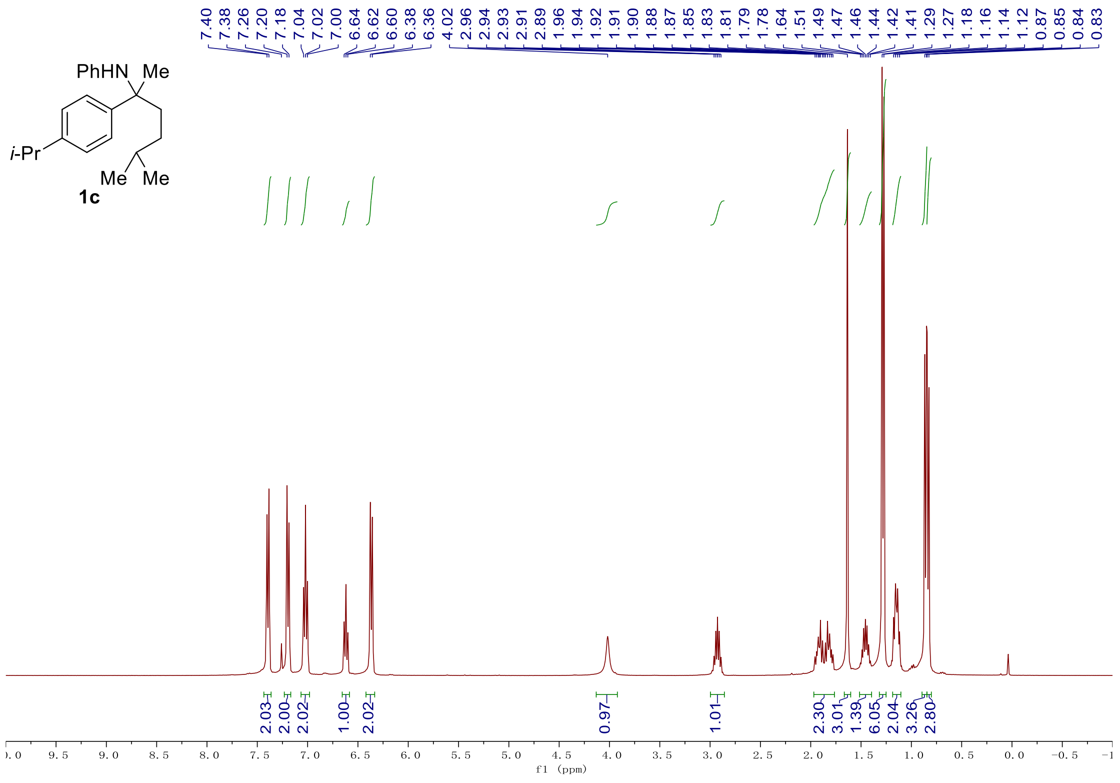

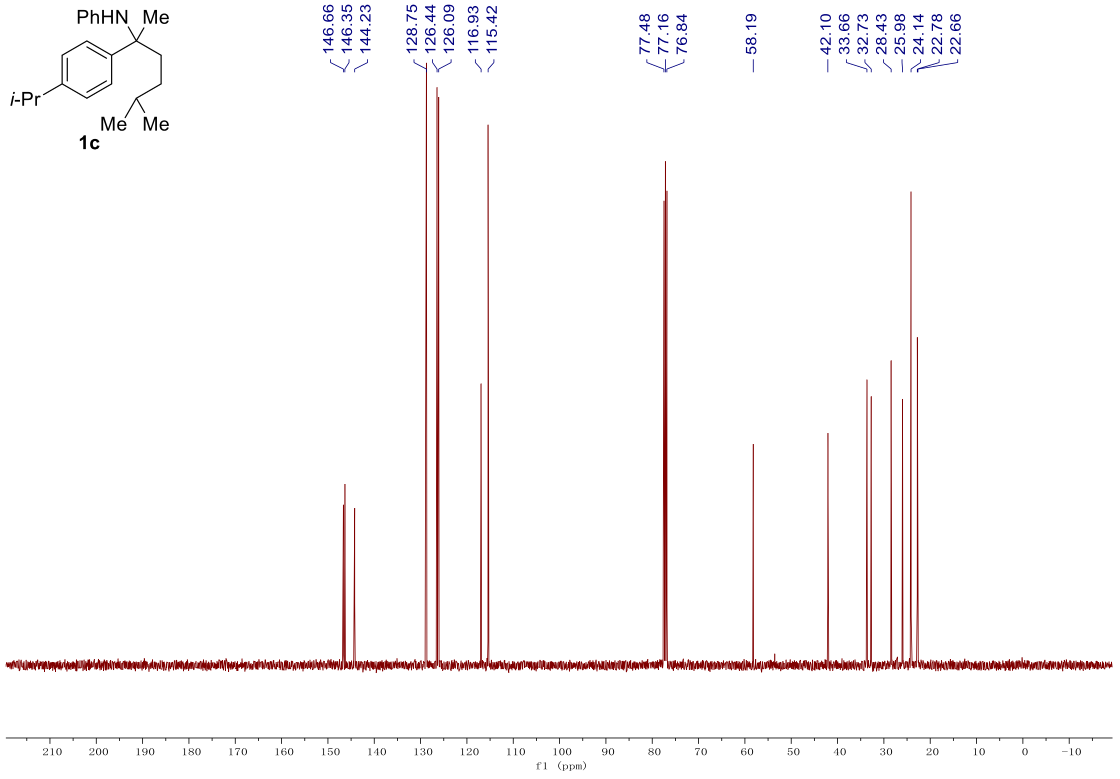

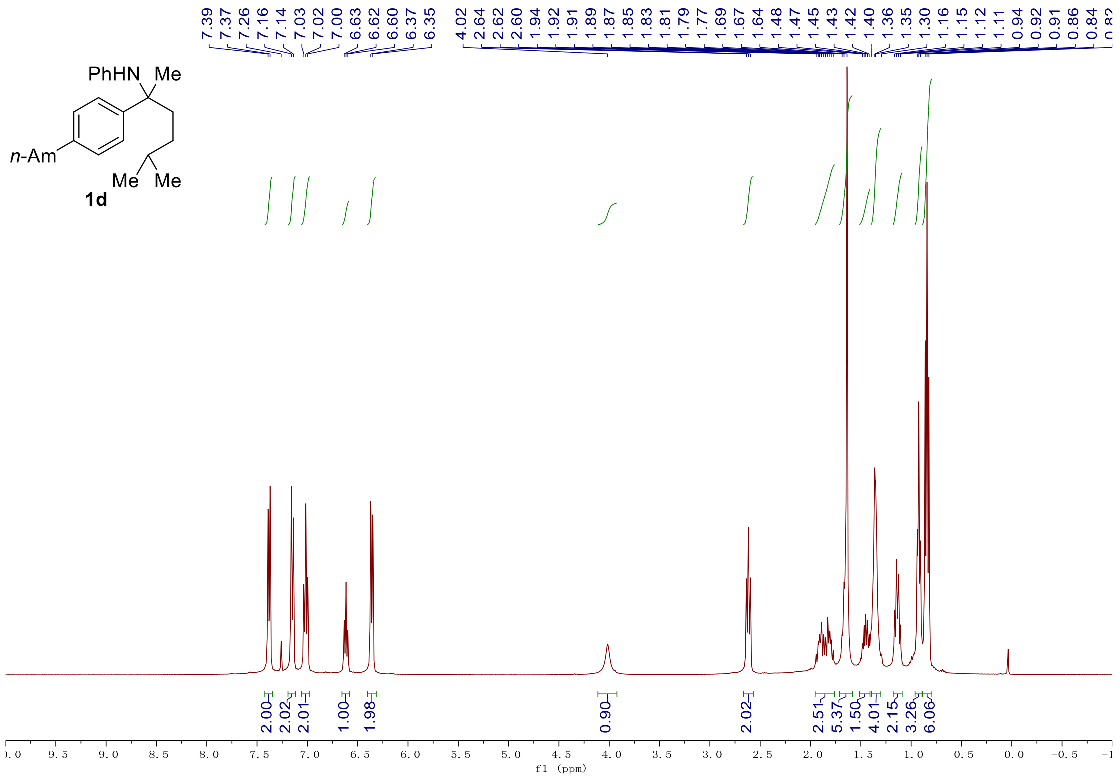
PhHN Me
n-Am
Me Me
1d
f1 (ppm)

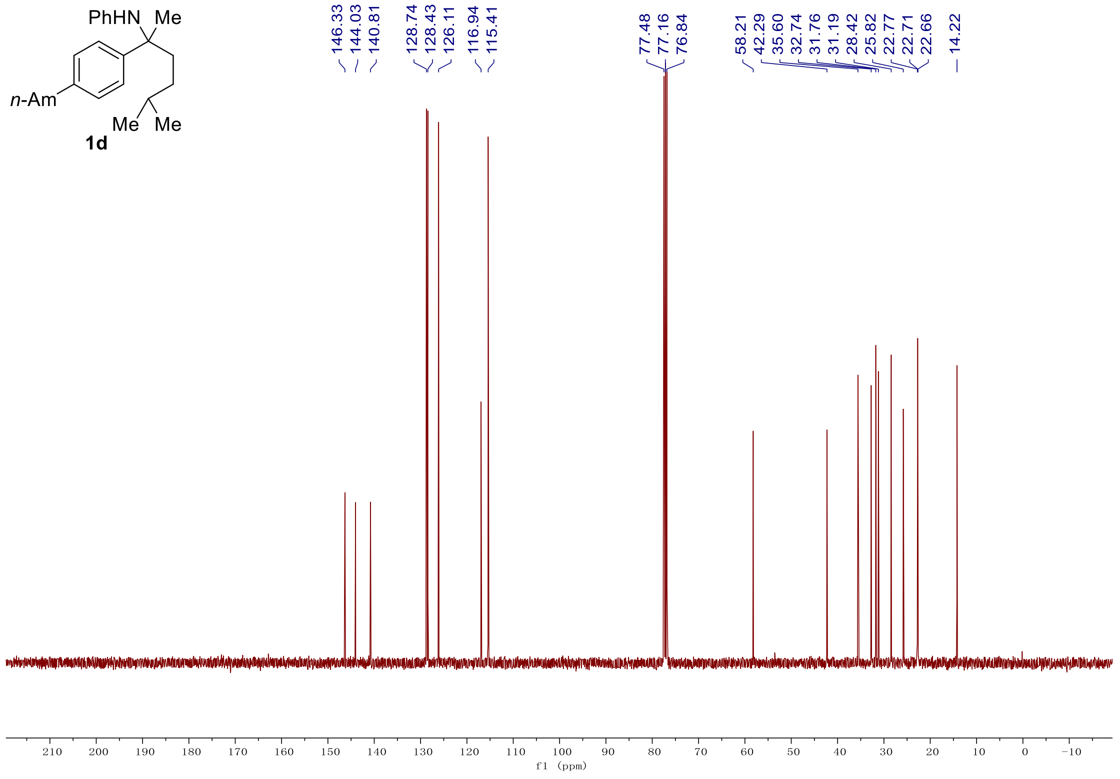
PhHN Me
n-Am
Me Me
1d
146.33
144.03
140.81
128.74
128.43
126.11
116.94
115.41
77.48
77.16
76.84
58.21
42.29
35.60
32.74
31.76
31.19
28.42
25.82
22.77
22.71
22.66
14.22
f1 (ppm)

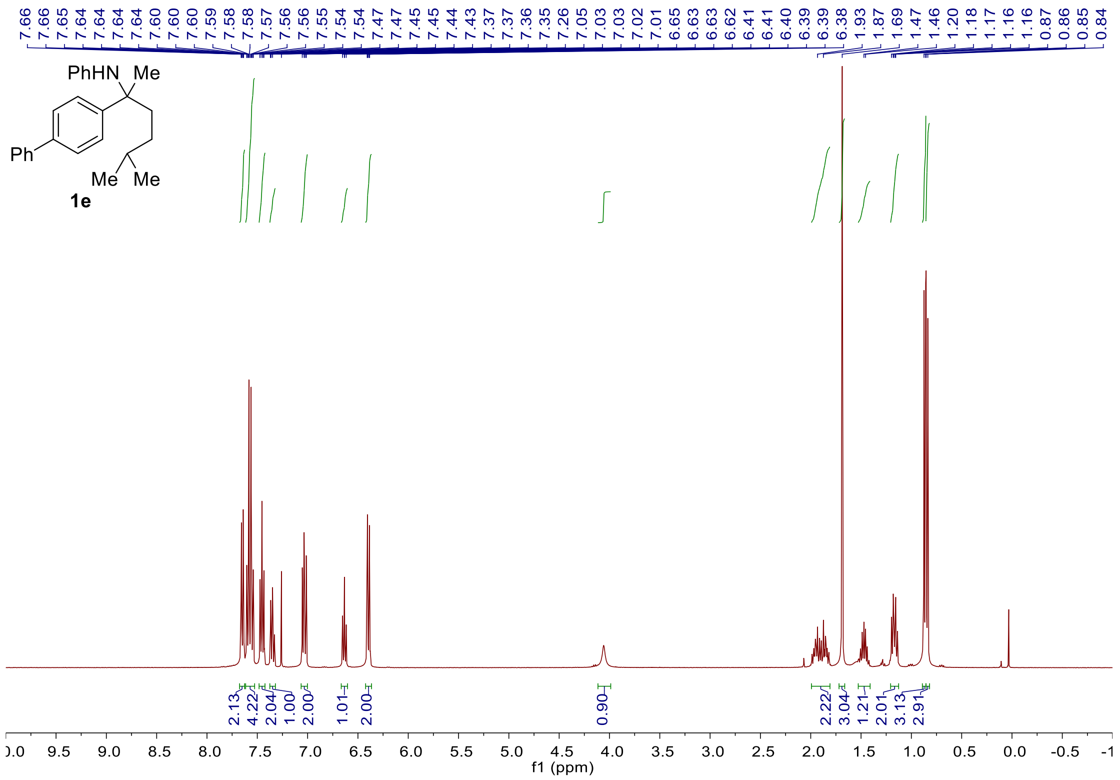
7.66 7.66 7.65 7.64 7.64 7.64 7.60 7.60 7.59 7.58 7.58 7.57 7.56 7.56 7.55 7.54 7.54 7.47 7.47 7.45 7.45 7.44 7.43 7.37 7.37 7.36 7.35 7.26 7.05 7.03 7.03 7.02 7.01 6.65 6.63 6.63 6.62 6.41 6.41 6.40 6.39 6.39 6.38 1.93 1.87 1.69 1.47 1.46 1.20 1.18 1.17 1.16 1.16 0.87 0.86 0.85 0.84
PhHN Me
Ph
Me Me
1e
2.13 4.22 2.04 1.00 2.00 1.01 2.00 0.90 2.22 3.04 1.21 2.01 3.13 2.91
f1 (ppm)

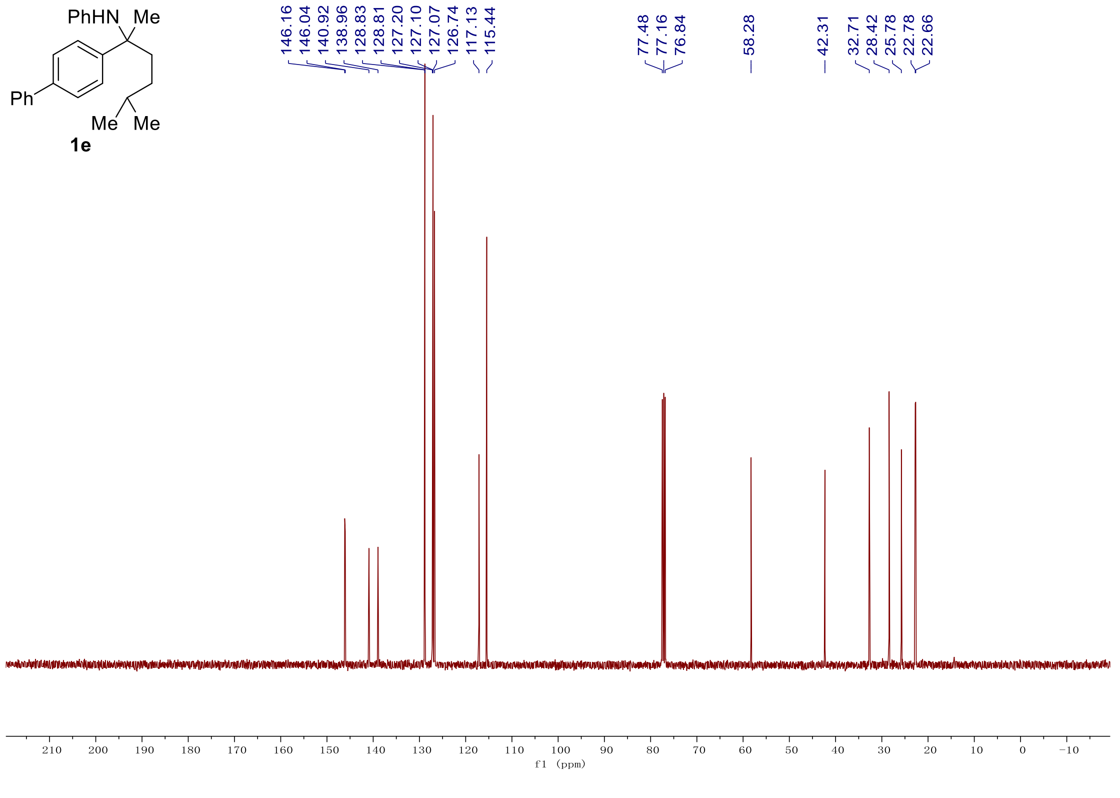
146.16 146.04 140.92 138.96 128.83 128.81 127.20 127.10 127.07 126.74 117.13 115.44
77.48 77.16 76.84
58.28
42.31
32.71 28.42 25.78 22.78 22.66
PhHN Me
Ph
Me Me
1e
f1 (ppm)

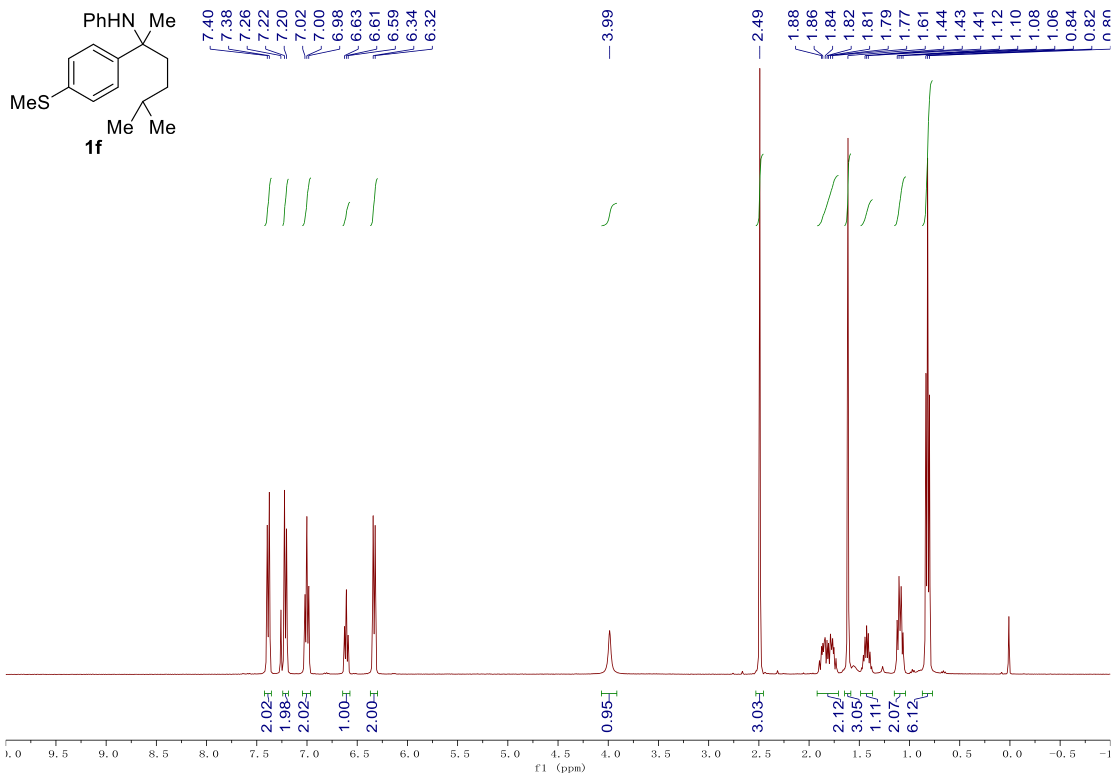

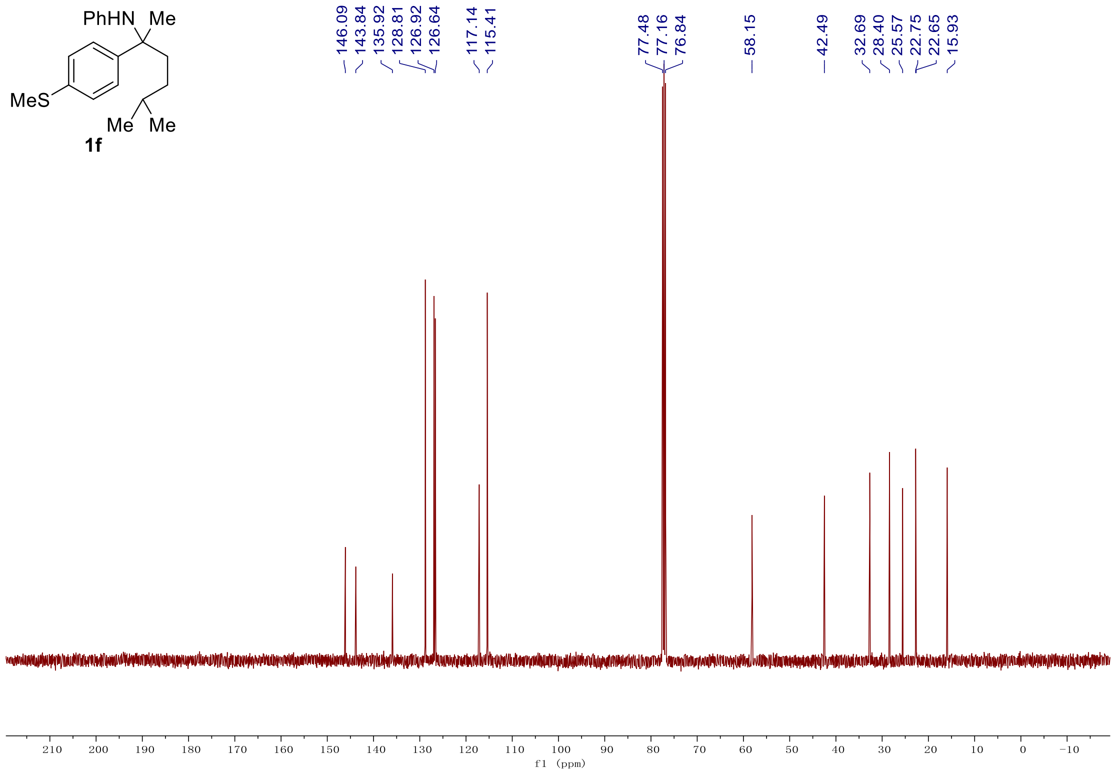

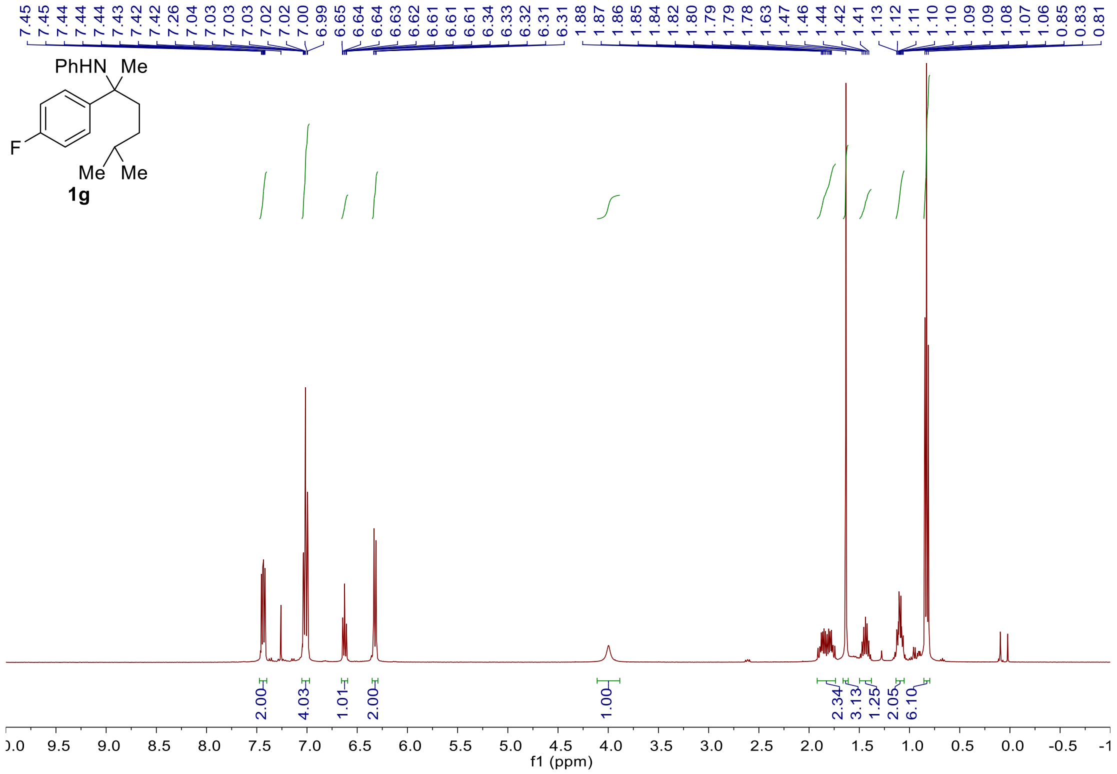
7.45 7.45 7.44 7.44 7.43 7.42 7.42 7.26 7.04 7.03 7.03 7.02 7.02 7.00 6.99 6.65 6.64 6.64 6.63 6.62 6.61 6.61 6.61 6.34 6.33 6.32 6.31 6.31 1.88 1.87 1.86 1.85 1.84 1.82 1.80 1.79 1.79 1.78 1.63 1.47 1.46 1.44 1.42 1.41 1.13 1.12 1.11 1.10 1.09 1.09 1.08 1.07 1.06 0.85 0.83 0.81
PhHN Me
F
Me Me
1g
2.00 4.03 1.01 2.00 1.00 2.34 3.13 1.25 2.05 6.10
f1 (ppm)

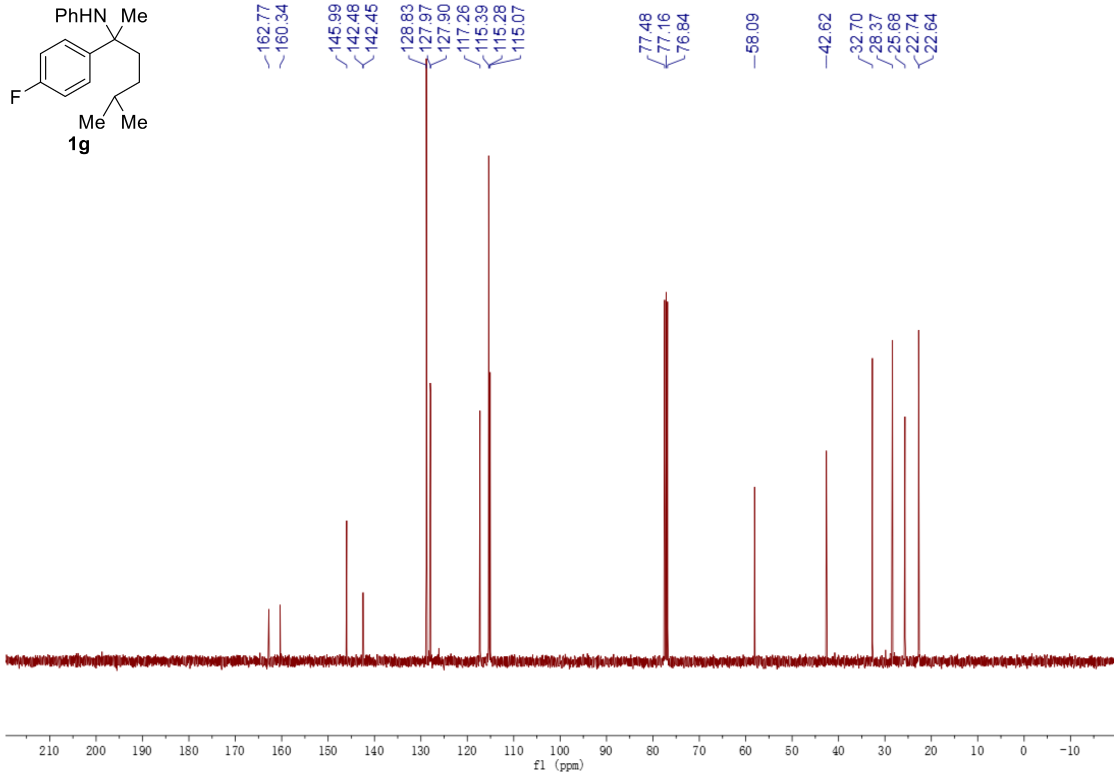
162.77 160.34 145.99 142.48 142.45 128.83 127.97 127.90 117.26 115.39 115.28 115.07 77.48 77.16 76.84 58.09 42.62 32.70 28.37 25.68 22.74 22.64
PhHN Me
F
Me Me
1g
f1 (ppm)

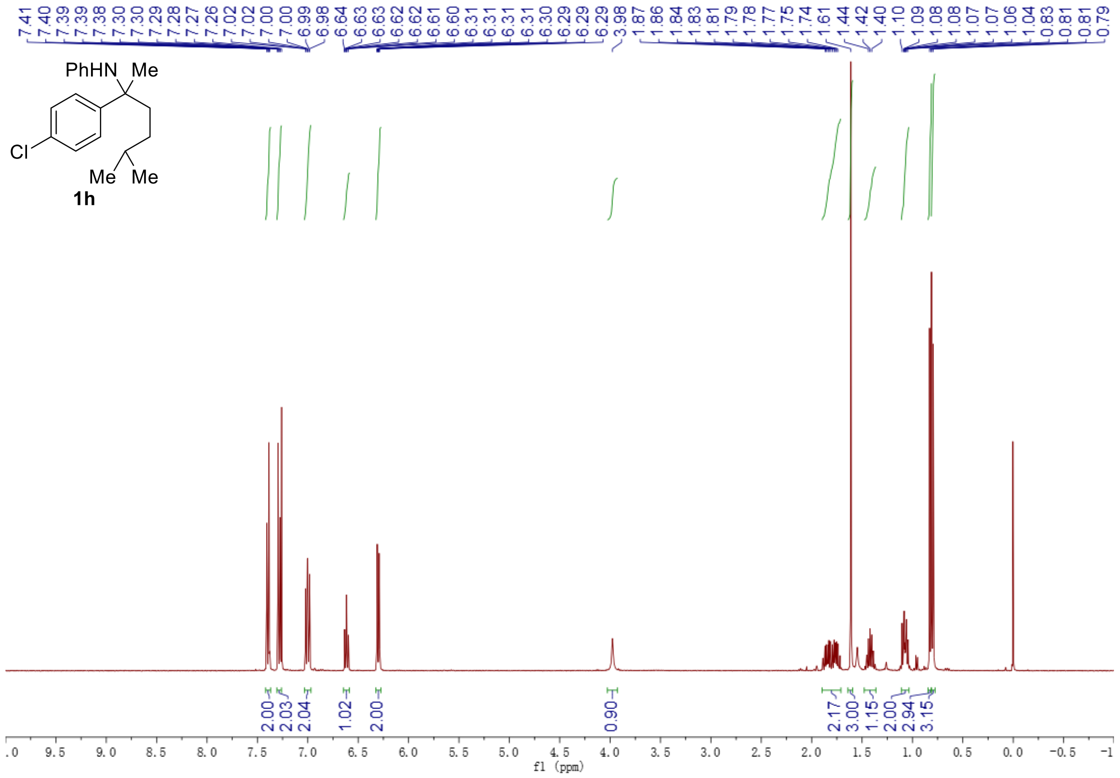

7.41
7.40
7.39
7.39
7.38
7.30
7.30
7.29
7.28
7.27
7.26
7.02
7.02
7.00
7.00
6.99
6.98
6.64
6.63
6.63
6.62
6.62
6.61
6.60
6.31
6.31
6.31
6.31
6.30
6.29
6.29
6.29
3.98
1.87
1.86
1.84
1.83
1.81
1.79
1.78
1.77
1.75
1.74
1.61
1.44
1.42
1.40
1.10
1.09
1.08
1.08
1.07
1.07
1.06
1.04
0.83
0.81
0.81
0.79
PhHN
Me
Cl
Me
Me
1h
2.00
2.03
2.04
1.02
2.00
0.90
2.17
3.00
1.15
2.00
2.94
3.15
f1 (ppm)

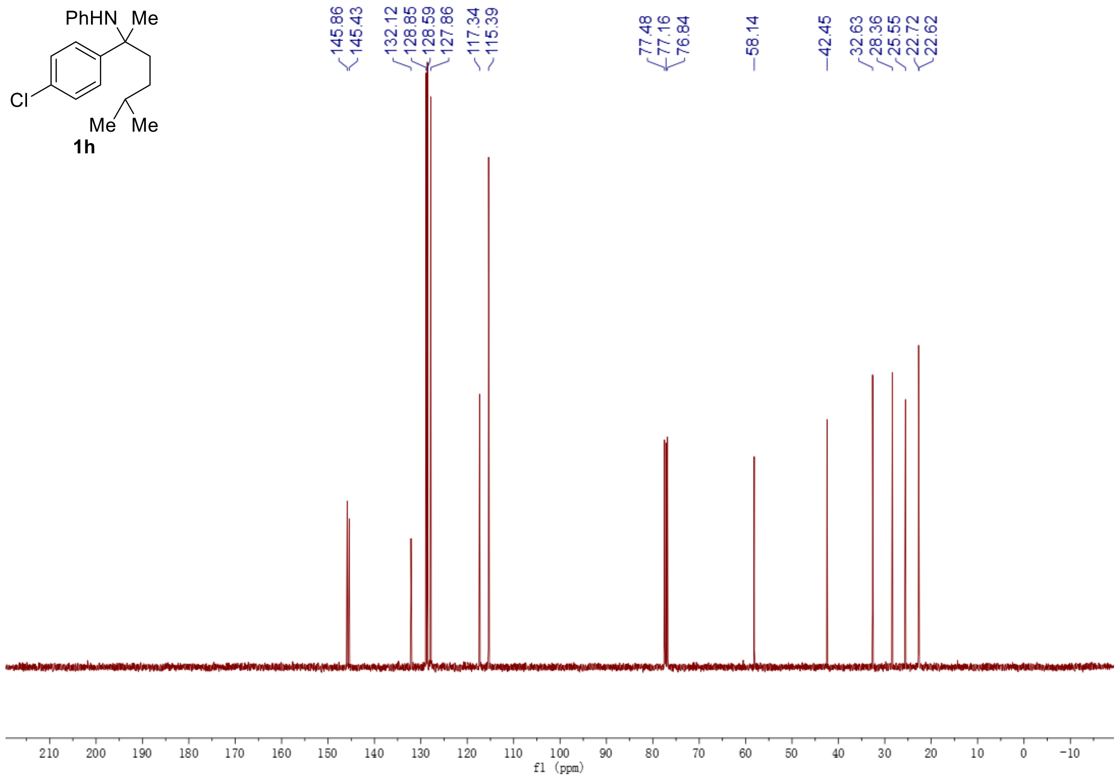

145.86
145.43
132.12
128.85
128.59
127.86
117.34
115.39
77.48
77.16
76.84
58.14
42.45
32.63
28.36
25.55
22.72
22.62
PhHN
Me
Cl
Me
Me
1h
f1 (ppm)

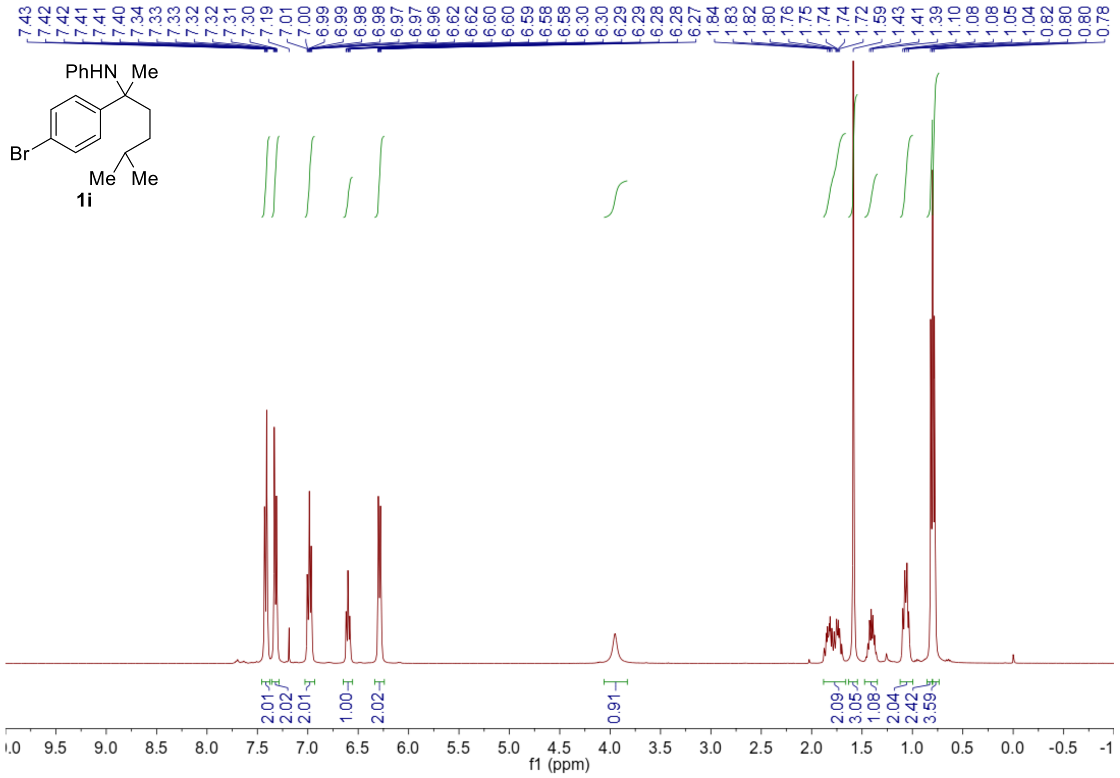
PhHN Me
Br
Me Me
1i
2.01
2.02
2.01
1.00
2.02
0.91
2.09
3.05
1.08
2.04
2.42
3.59
f1 (ppm)

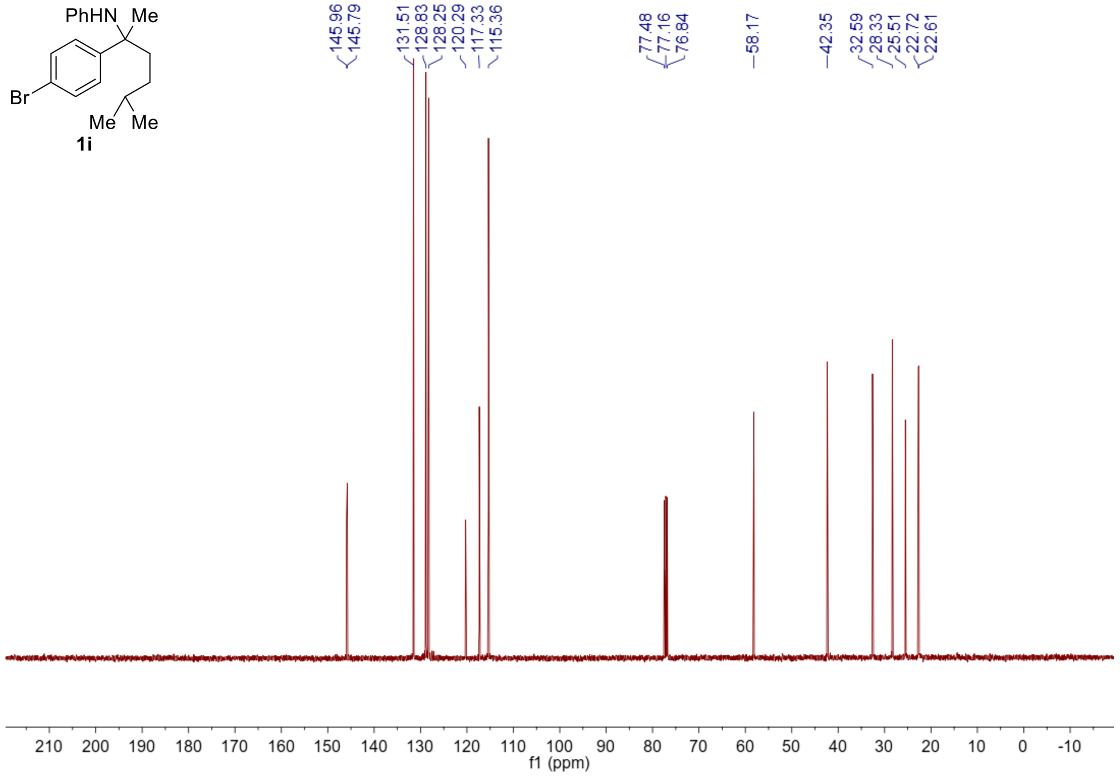
PhHN Me
Br
Me Me
1i
145.96
145.79
131.51
128.83
128.25
120.29
117.33
115.36
77.48
77.16
76.84
58.17
42.35
32.59
28.33
25.51
22.72
22.61
f1 (ppm)

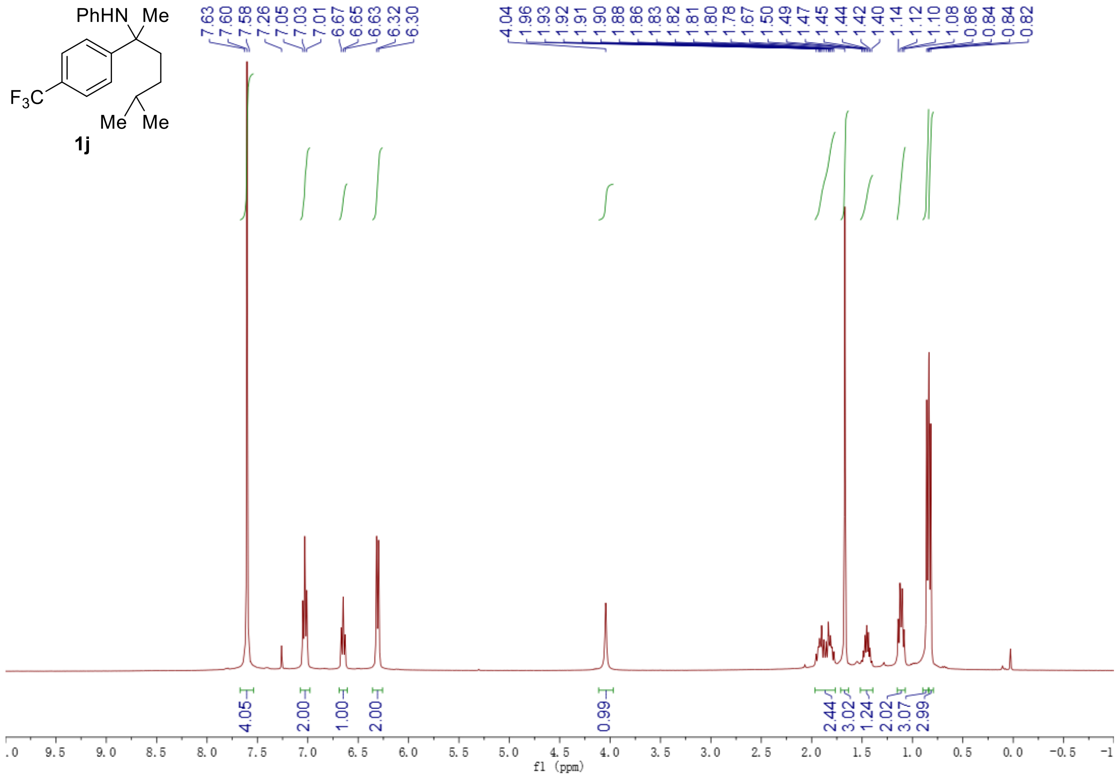
PhHN Me
F3C
Me Me
1j
7.63 7.60 7.58 7.26 7.05 7.03 7.01 6.67 6.65 6.63 6.32 6.30
4.04 1.96 1.93 1.92 1.91 1.90 1.88 1.86 1.83 1.82 1.81 1.80 1.78 1.67 1.50 1.49 1.47 1.45 1.44 1.42 1.40 1.14 1.12 1.10 1.08 0.86 0.84 0.84 0.82
4.05 2.00 1.00 2.00 0.99 2.44 3.02 1.24 2.02 3.07 2.99
f1 (ppm)

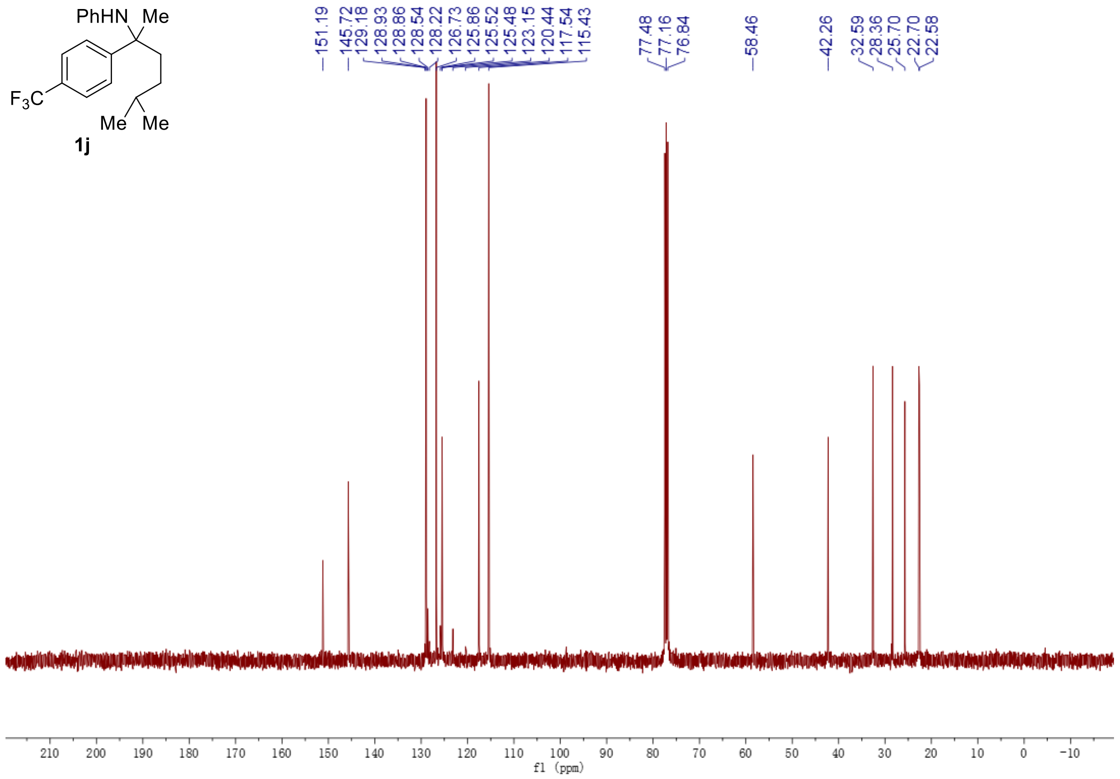
PhHN Me
F3C
Me Me
1j
151.19 145.72 129.18 128.93 128.86 128.54 128.22 126.73 125.86 125.52 125.48 123.15 120.44 117.54 115.43
77.48 77.16 76.84
58.46
42.26
32.59 28.36 25.70 22.70 22.58
f1 (ppm)

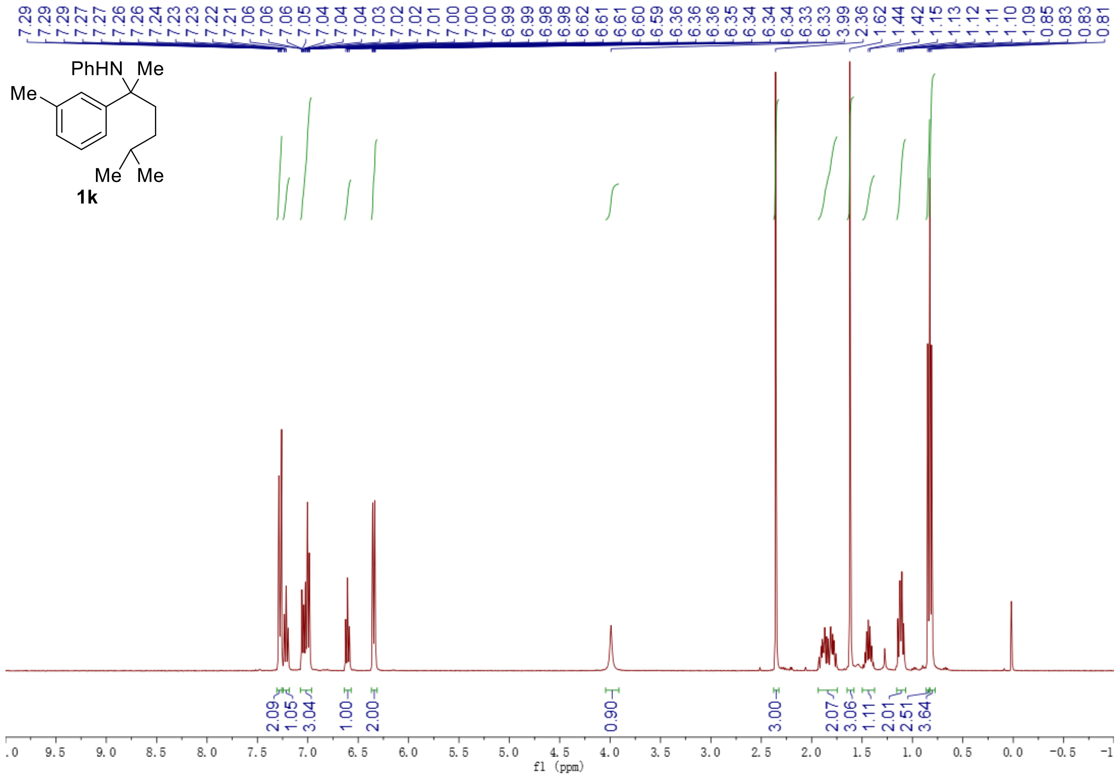

1k

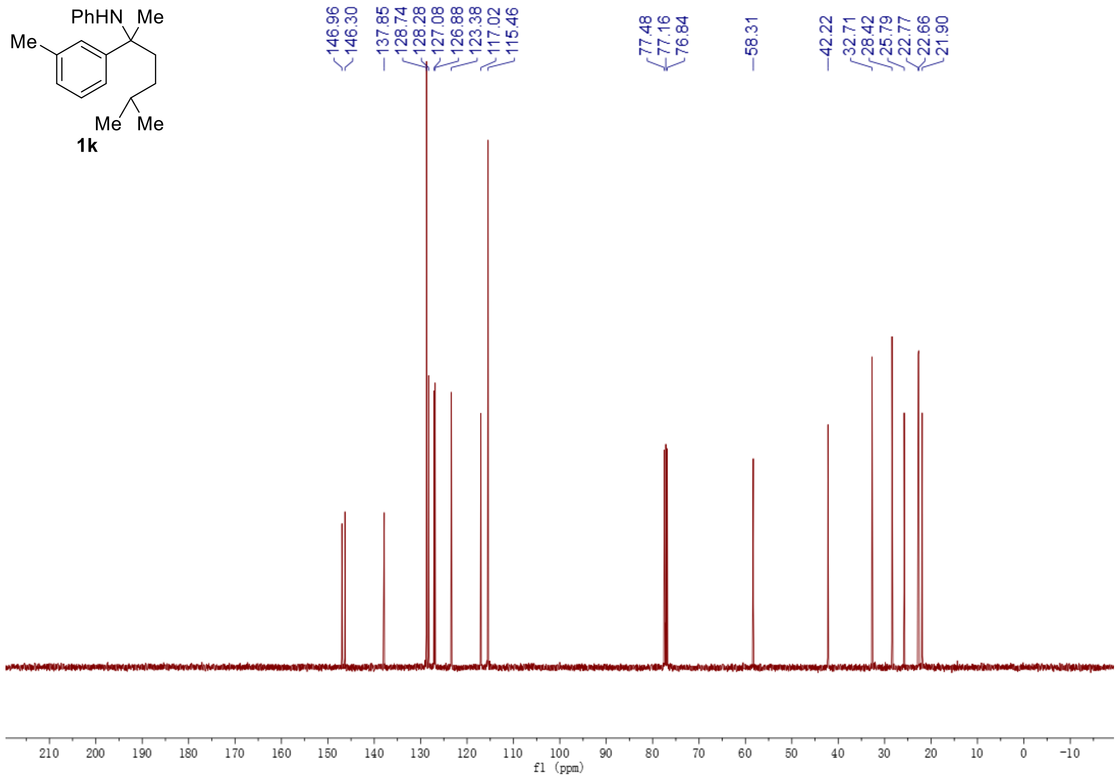

1k

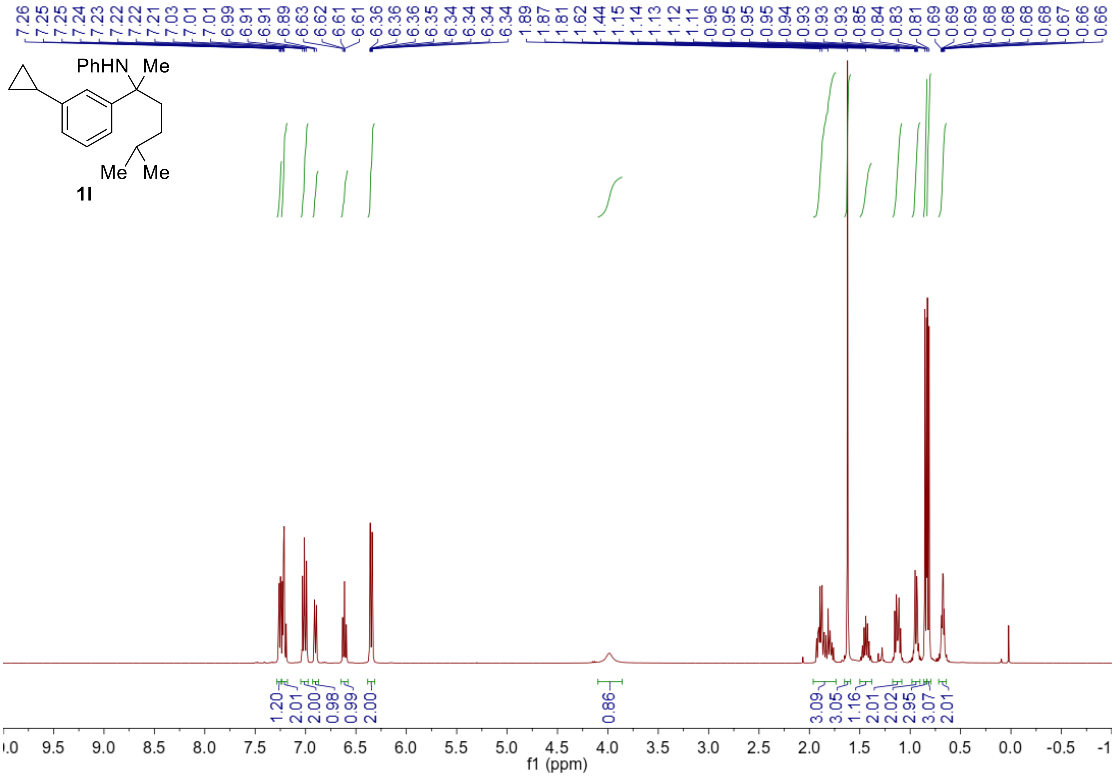
7.26 7.25 7.25 7.24 7.23 7.22 7.22 7.21 7.03 7.01 7.01 6.99 6.91 6.91 6.89 6.63 6.62 6.61 6.61 6.36 6.36 6.36 6.35 6.34 6.34 6.34 6.34 1.89 1.87 1.81 1.62 1.44 1.15 1.14 1.13 1.12 1.11 0.96 0.95 0.95 0.95 0.94 0.93 0.93 0.93 0.85 0.84 0.83 0.81 0.69 0.69 0.69 0.68 0.68 0.68 0.67 0.66 0.66
PhHN Me
Me Me
1l
1.20 2.01 2.00 0.98 0.99 2.00 0.86 3.09 3.05 1.16 2.01 2.02 2.95 3.07 2.01
f1 (ppm)

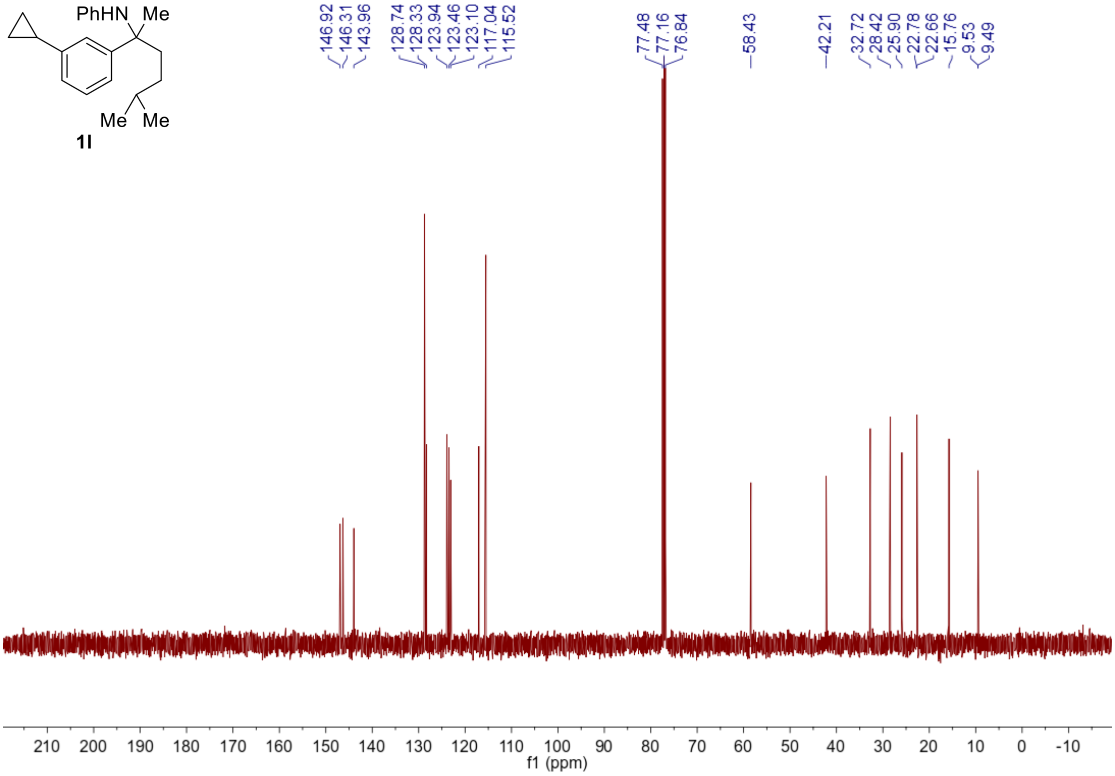
PhHN Me
Me Me
1l
146.92 146.31 143.96 128.74 128.33 123.94 123.46 123.10 117.04 115.52 77.48 77.16 76.84 58.43 42.21 32.72 28.42 25.90 22.78 22.66 15.76 9.53 9.49
f1 (ppm)

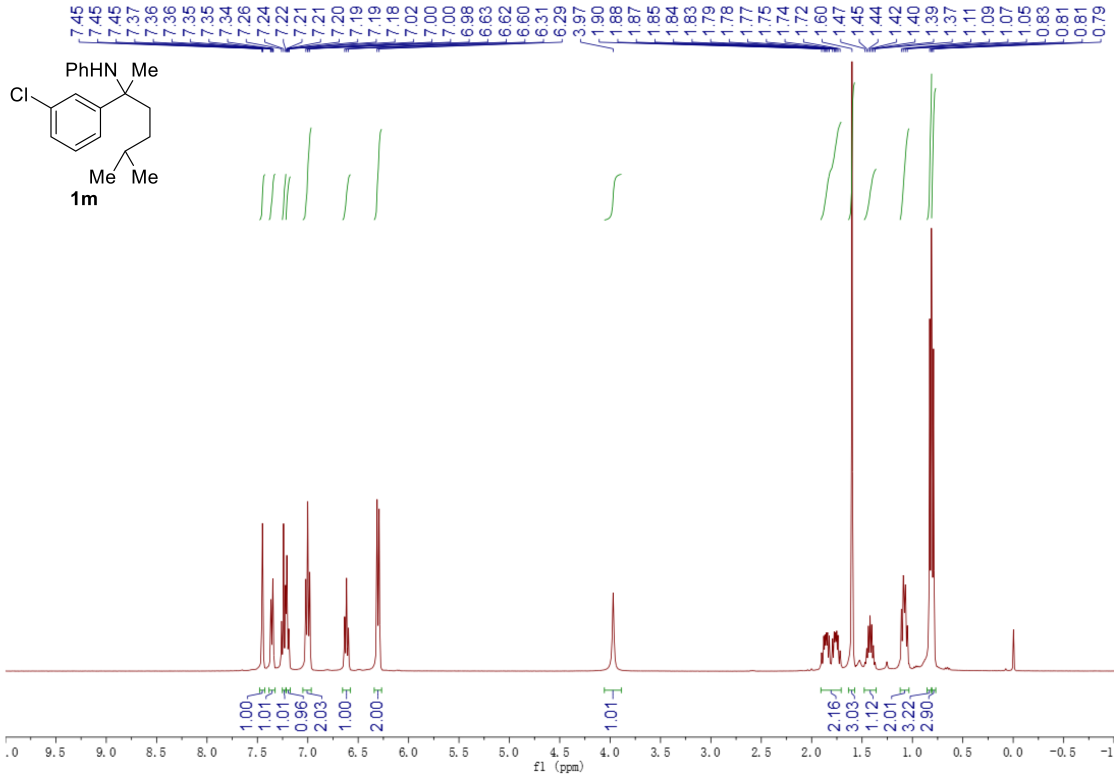
PhHN Me
Cl
Me Me
1m
7.45 7.45 7.37 7.36 7.36 7.35 7.35 7.34 7.26 7.24 7.22 7.21 7.21 7.20 7.19 7.18 7.02 7.00 7.00 6.98 6.63 6.62 6.60 6.31 6.29 3.97 1.90 1.88 1.87 1.85 1.84 1.83 1.79 1.78 1.77 1.75 1.74 1.72 1.60 1.47 1.45 1.44 1.42 1.40 1.39 1.37 1.11 1.09 1.07 1.05 0.83 0.81 0.81 0.79
1.00 1.01 1.01 0.96 2.03 1.00 2.00 1.01 2.16 3.03 1.12 2.01 3.22 2.90
f1 (ppm)

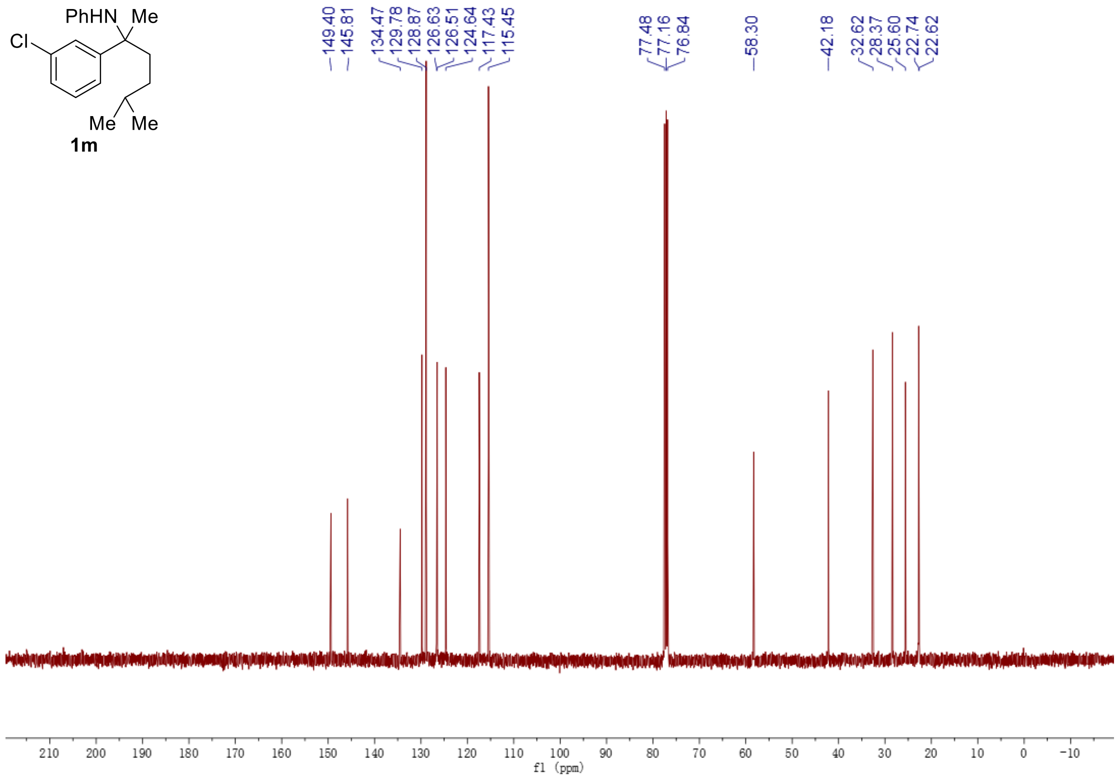
PhHN Me
Cl
Me Me
1m
149.40 145.81 134.47 129.78 128.87 126.63 126.51 124.64 117.43 115.45 77.48 77.16 76.84 58.30 42.18 32.62 28.37 25.60 22.74 22.62
f1 (ppm)

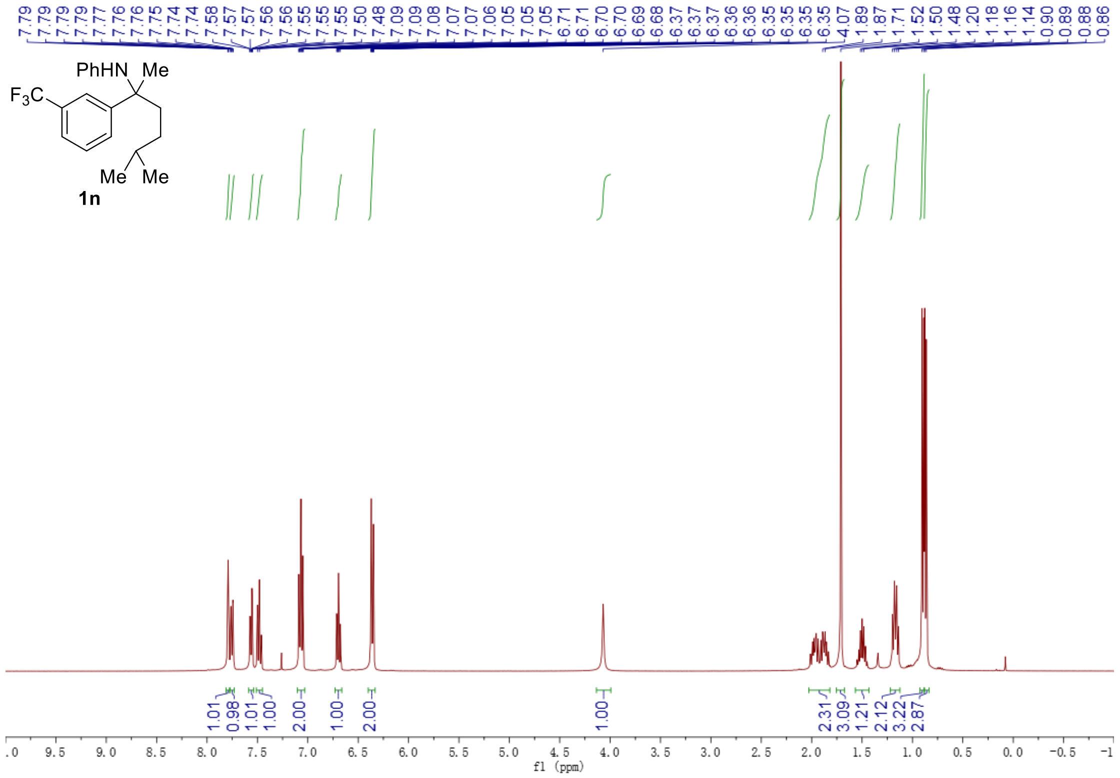

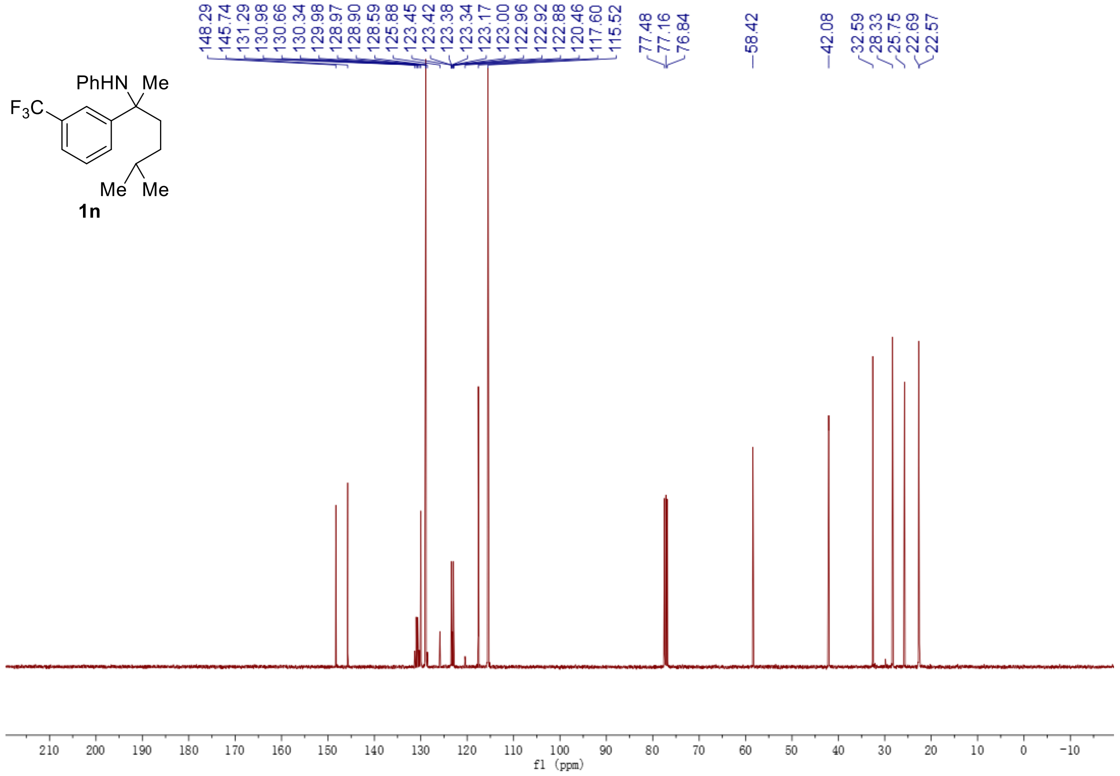

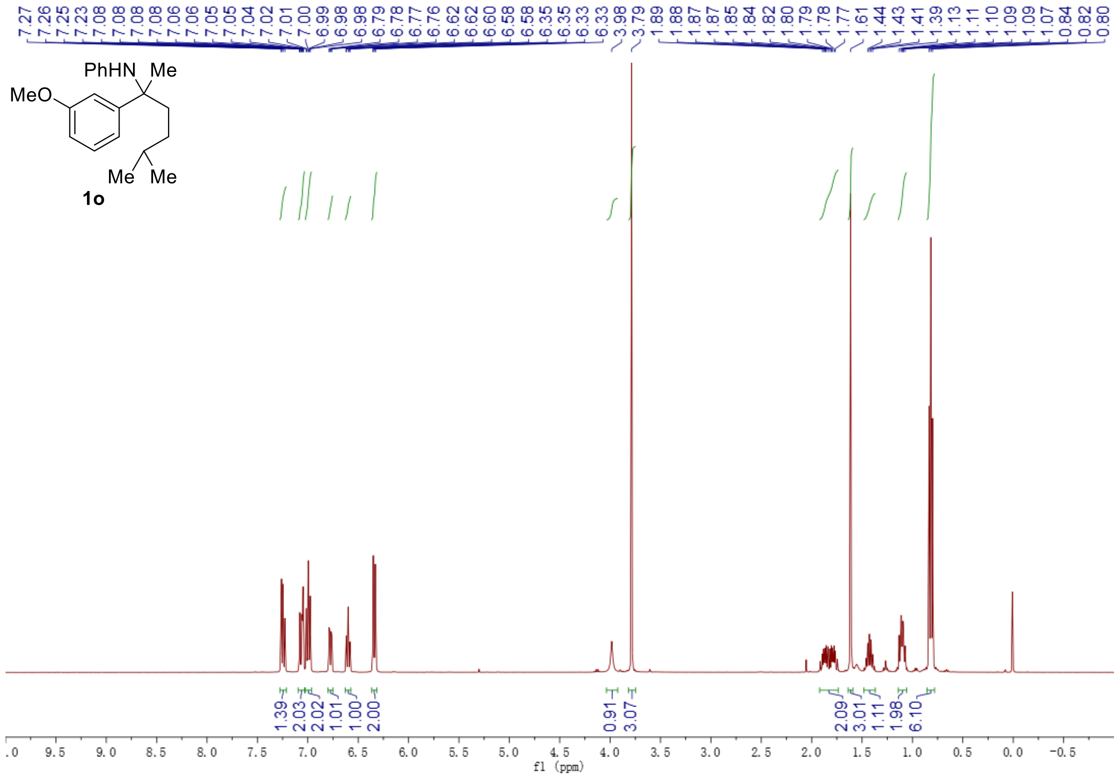
7.27 7.26 7.25 7.23 7.08 7.08 7.08 7.08 7.06 7.06 7.05 7.05 7.04 7.02 7.01 7.00 6.99 6.98 6.98 6.79 6.78 6.77 6.76 6.62 6.62 6.60 6.58 6.58 6.35 6.35 6.33 6.33 3.98 3.79 1.89 1.88 1.87 1.87 1.85 1.84 1.82 1.80 1.79 1.78 1.77 1.61 1.44 1.43 1.41 1.39 1.13 1.11 1.10 1.09 1.07 0.84 0.82 0.80
PhHN Me
MeO
Me Me
1o
1.39 2.03 2.02 1.01 1.00 2.00 0.91 3.07 2.09 3.01 1.11 1.98 6.10
f1 (ppm)

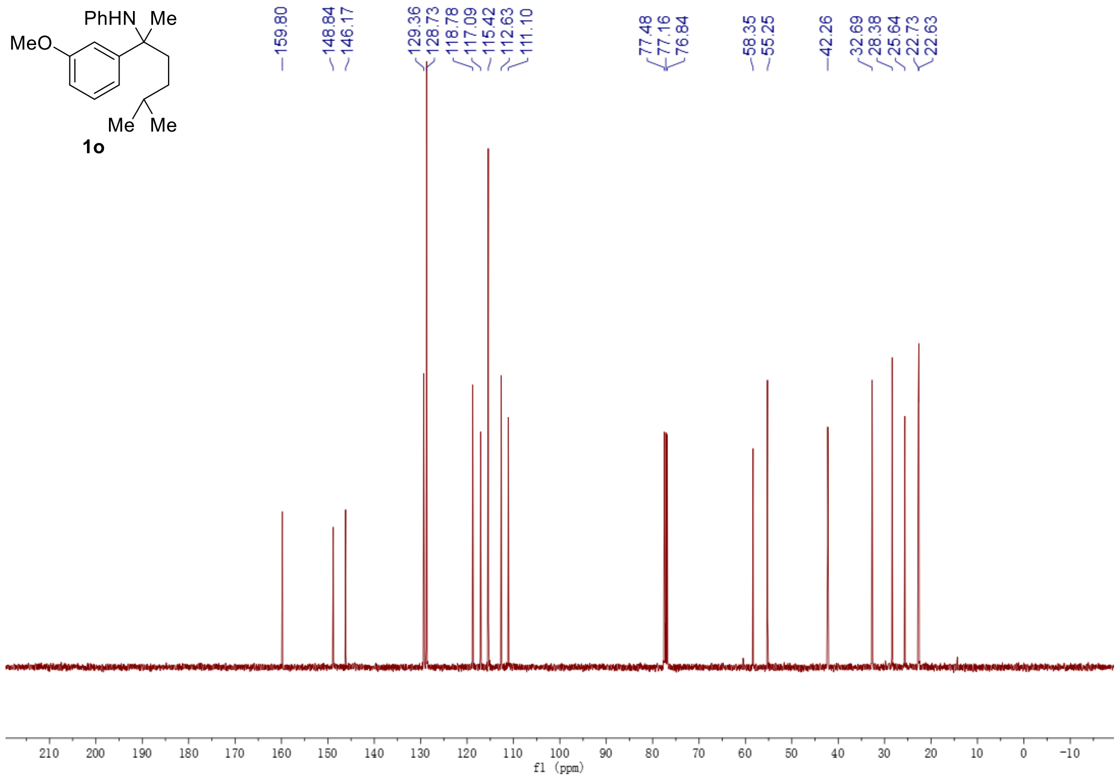
PhHN Me
MeO
Me Me
1o
159.80 148.84 146.17 129.36 128.73 118.78 117.09 115.42 112.63 111.10 77.48 77.16 76.84 58.35 55.25 42.26 32.69 28.38 25.64 22.73 22.63
f1 (ppm)

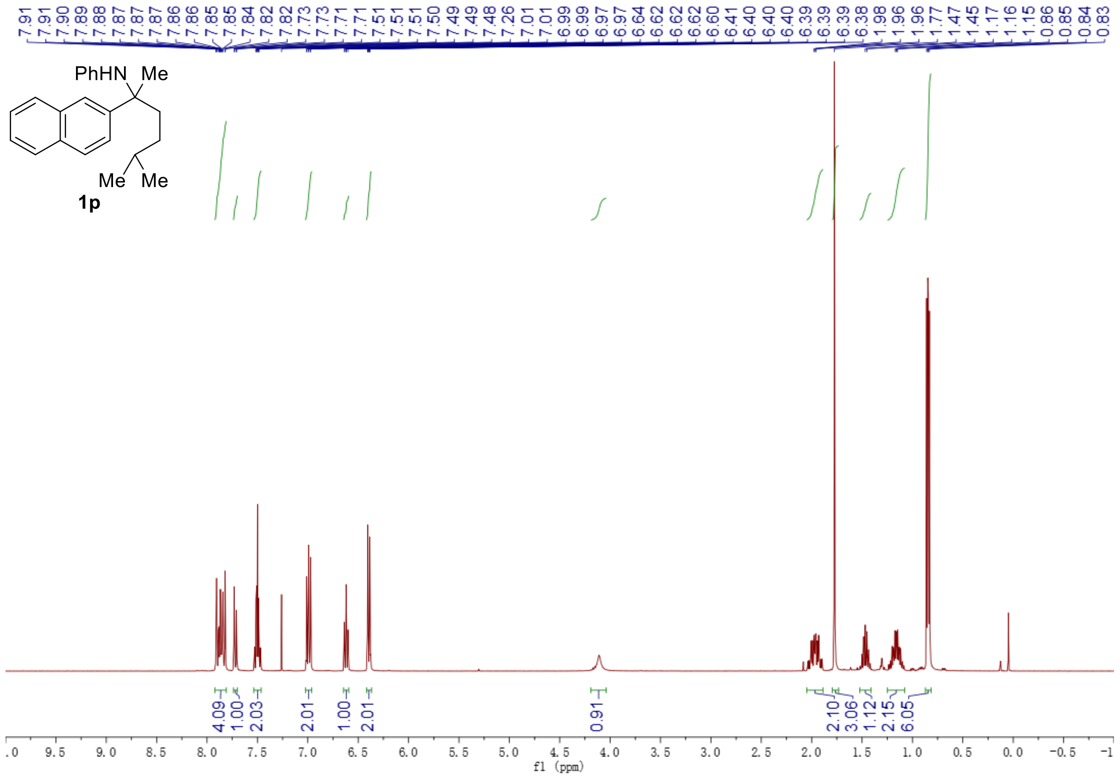
PhHN
Me
Me
Me
1p
f1 (ppm)

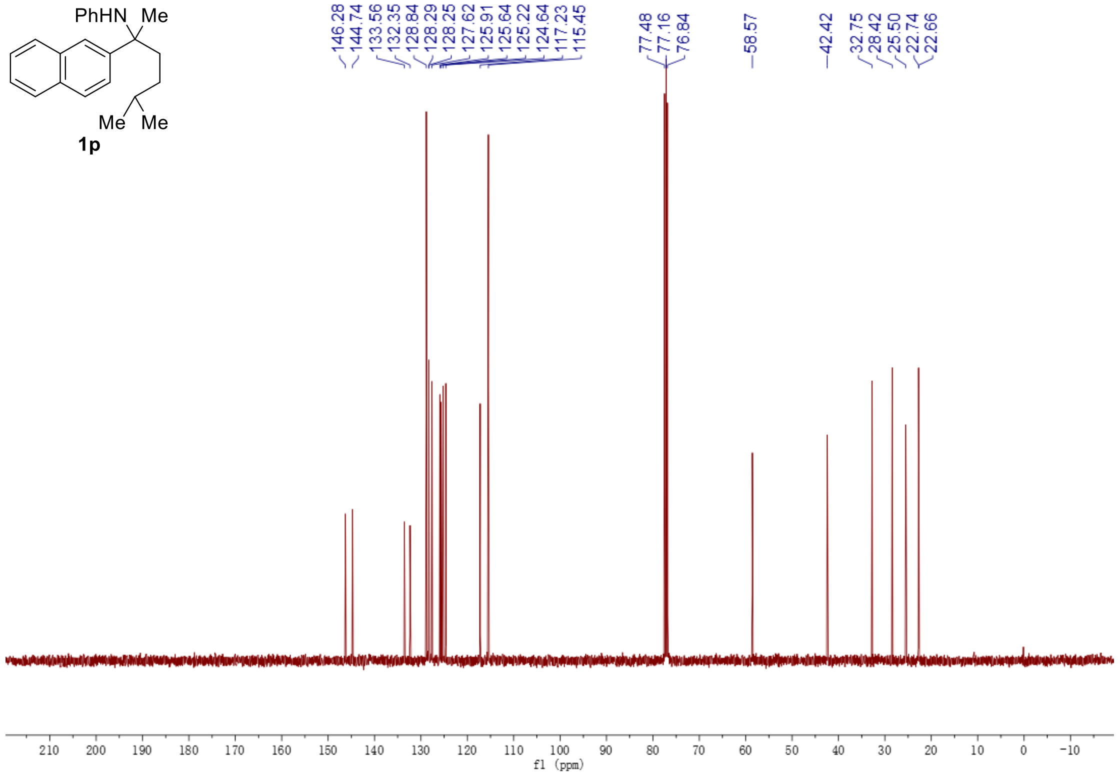
PhHN
Me
Me
Me
1p
f1 (ppm)

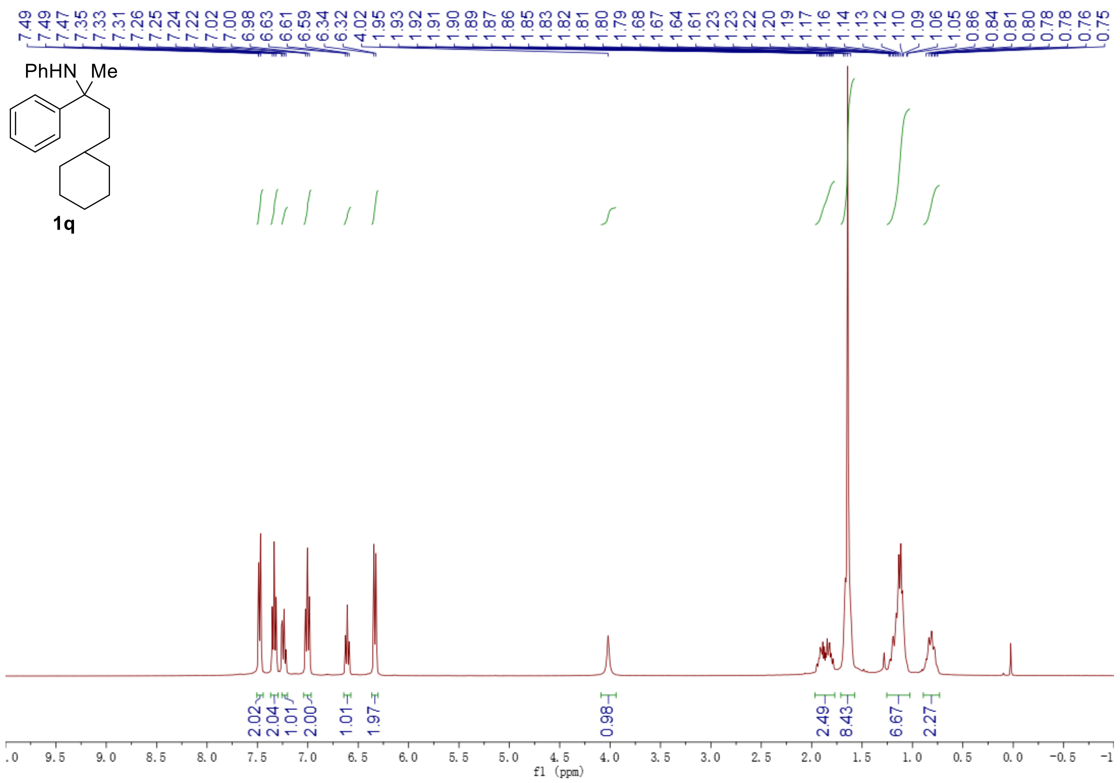
PhHN Me
1q

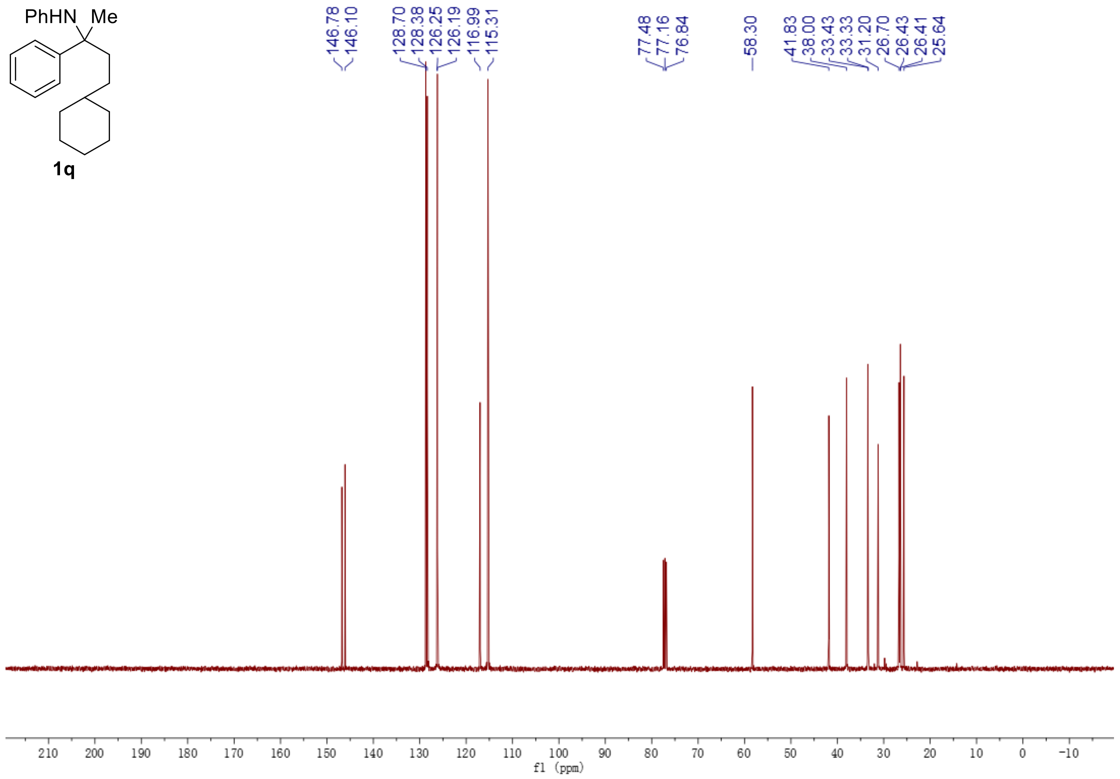
PhHN Me
1q

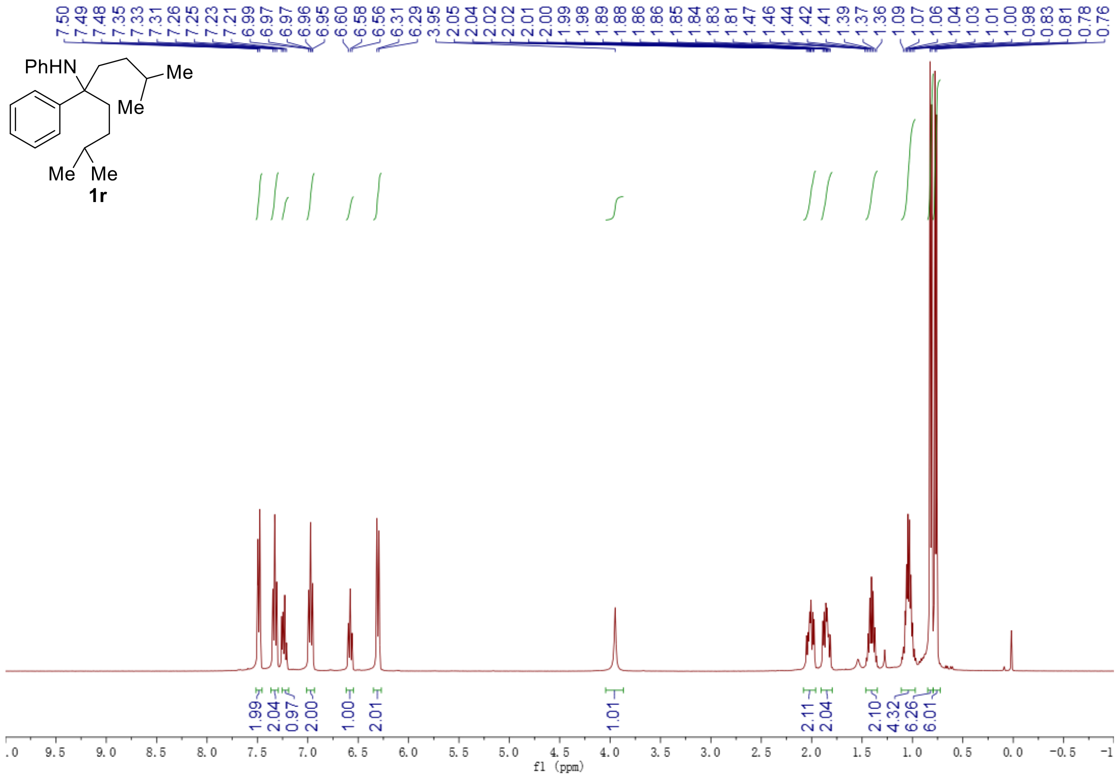
7.50 7.49 7.48 7.35 7.33 7.31 7.26 7.25 7.23 7.21 6.99 6.97 6.97 6.96 6.95 6.60 6.58 6.56 6.31 6.29 3.95 2.05 2.04 2.02 2.02 2.01 2.00 1.99 1.98 1.89 1.88 1.86 1.85 1.84 1.83 1.81 1.47 1.46 1.44 1.42 1.41 1.39 1.37 1.36 1.09 1.07 1.06 1.04 1.03 1.01 1.00 0.98 0.83 0.81 0.78 0.76
PhHN
Me
Me
Me
Me
1r
1.99 2.04 0.97 2.00 1.00 2.01 1.01 2.11 2.04 2.10 4.32 6.26 6.01
f1 (ppm)

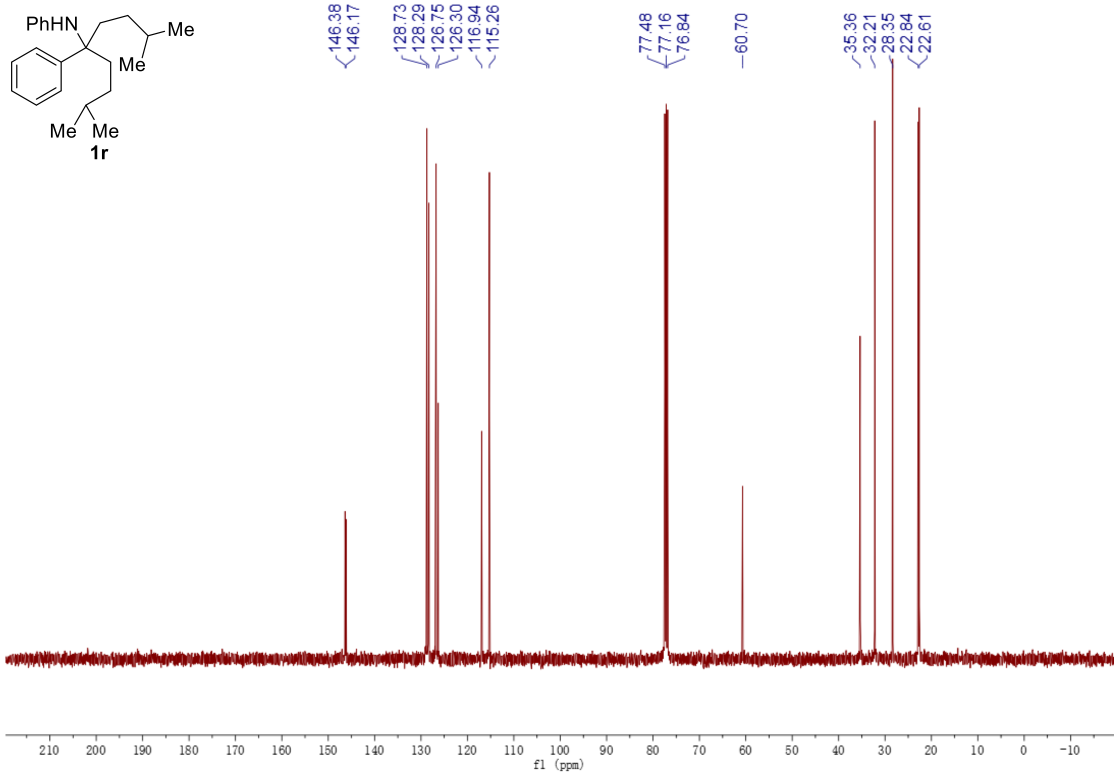
PhHN
Me
Me
Me
Me
1r
146.38 146.17
128.73 128.29 126.75 126.30 116.94 115.26
77.48 77.16 76.84
60.70
35.36 32.21 28.35 22.84 22.61
f1 (ppm)

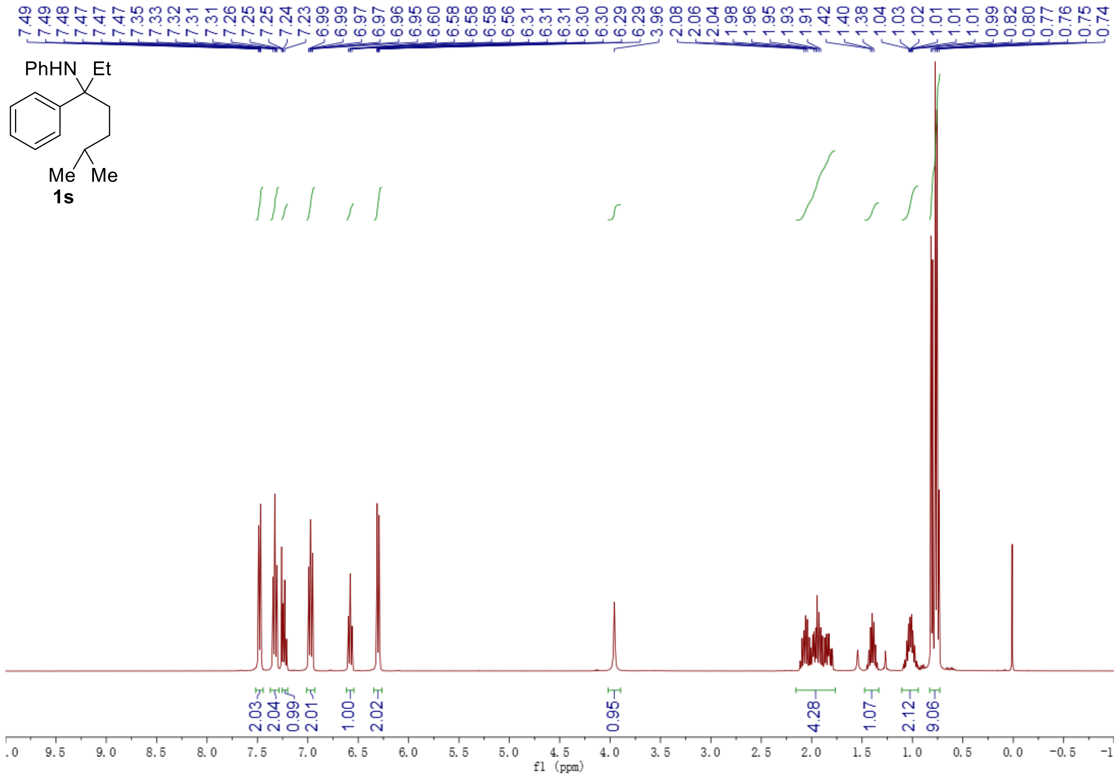

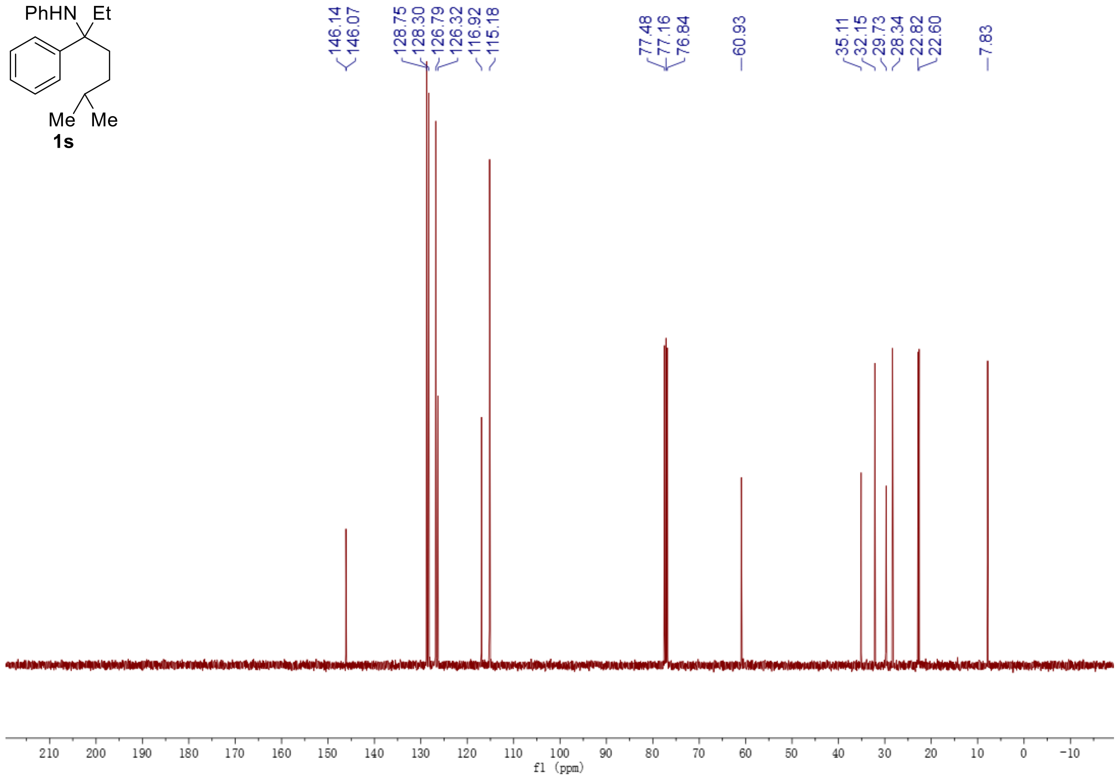

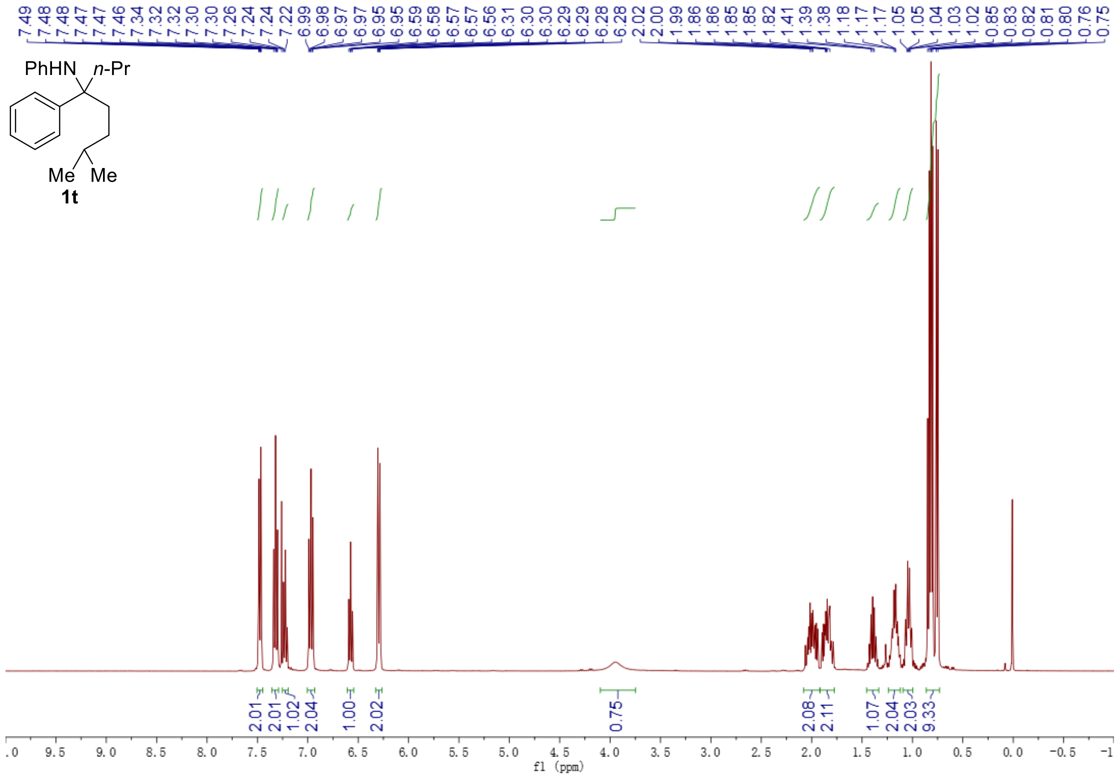
PhHN n-Pr
Me Me
1t
7.49 7.48 7.48 7.47 7.47 7.46 7.34 7.32 7.32 7.30 7.26 7.24 7.24 7.22 6.99 6.98 6.97 6.97 6.95 6.95 6.59 6.58 6.57 6.57 6.56 6.31 6.30 6.29 6.29 6.28 6.28 2.02 2.00 1.99 1.86 1.85 1.85 1.82 1.41 1.39 1.38 1.18 1.17 1.17 1.05 1.05 1.04 1.03 1.02 0.85 0.83 0.82 0.81 0.80 0.76 0.75
2.01 2.01 1.02 2.04 1.00 2.02 0.75 2.08 2.11 1.07 2.04 2.03 9.33
f1 (ppm)

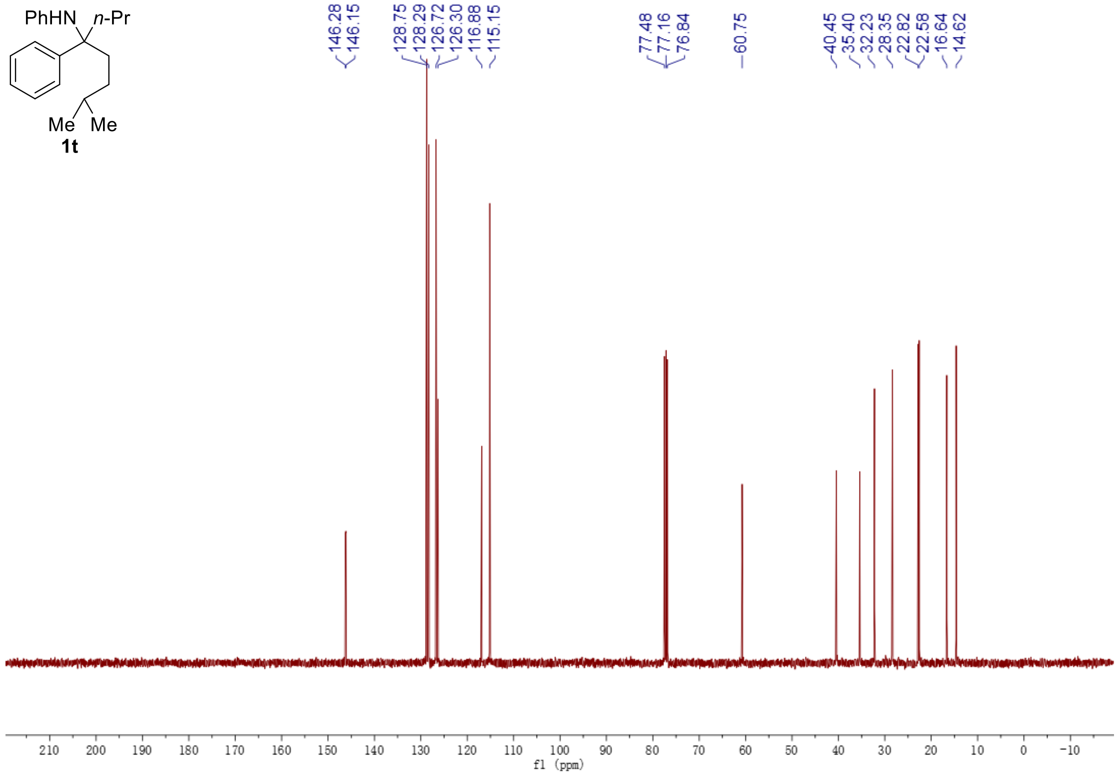
PhHN n-Pr
Me Me
1t
146.28 146.15 128.75 128.29 126.72 126.30 116.88 115.15 77.48 77.16 76.84 60.75 40.45 35.40 32.23 28.35 22.82 22.58 16.64 14.62
f1 (ppm)

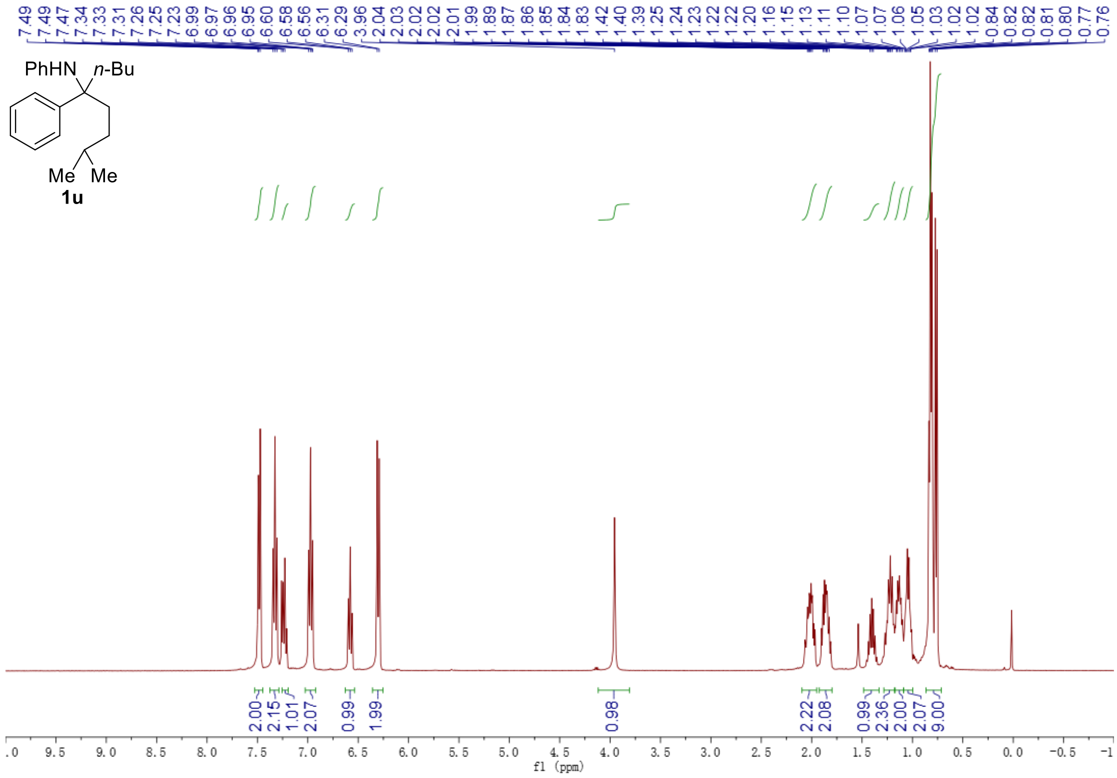
PhHN n-Bu
Me Me
1u
7.49 7.49 7.47 7.34 7.33 7.31 7.26 7.25 7.23 6.99 6.97 6.96 6.95 6.60 6.58 6.56 6.31 6.29 3.96 2.04 2.03 2.02 2.02 2.01 1.99 1.89 1.87 1.86 1.85 1.84 1.83 1.42 1.40 1.39 1.25 1.24 1.23 1.22 1.22 1.20 1.16 1.15 1.13 1.11 1.10 1.07 1.07 1.06 1.05 1.03 1.02 1.02 0.84 0.82 0.82 0.81 0.80 0.77 0.76
2.00 2.15 1.01 2.07 0.99 1.99 0.98 2.22 2.08 0.99 2.36 2.00 2.07 9.00
f1 (ppm)

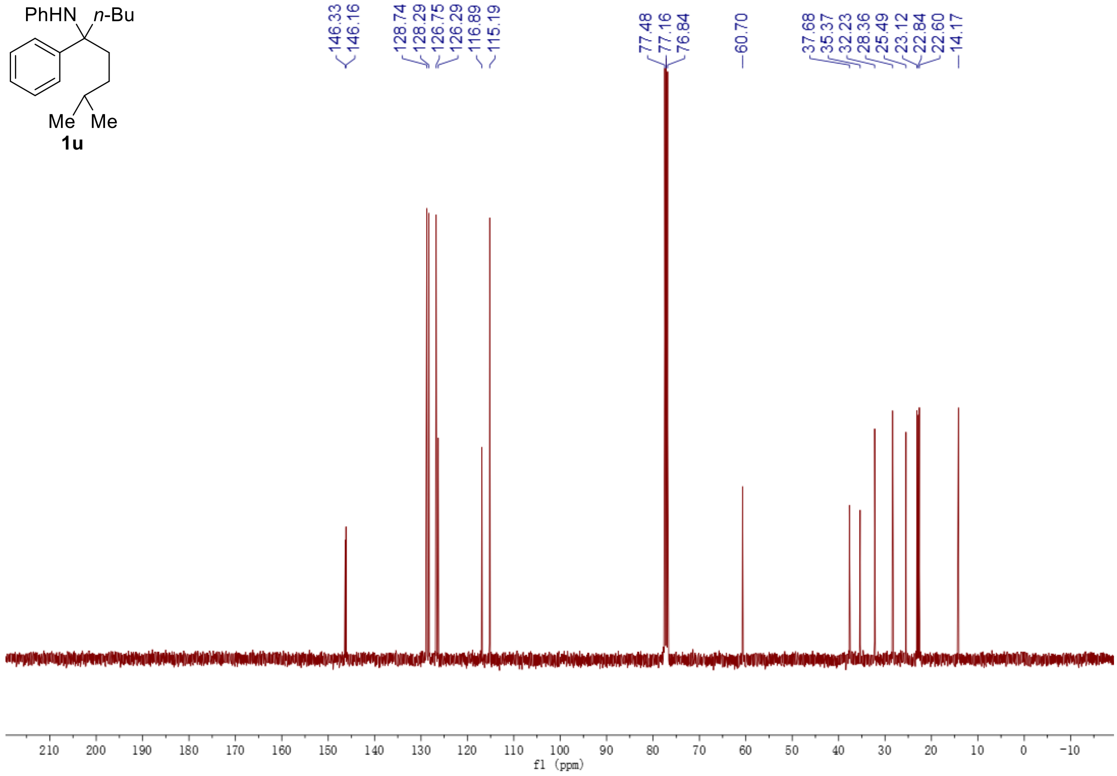
PhHN n-Bu
Me Me
1u
146.33 146.16 128.74 128.29 126.75 126.29 116.89 115.19 77.48 77.16 76.84 60.70 37.68 35.37 32.23 28.36 25.49 23.12 22.84 22.60 14.17
f1 (ppm)

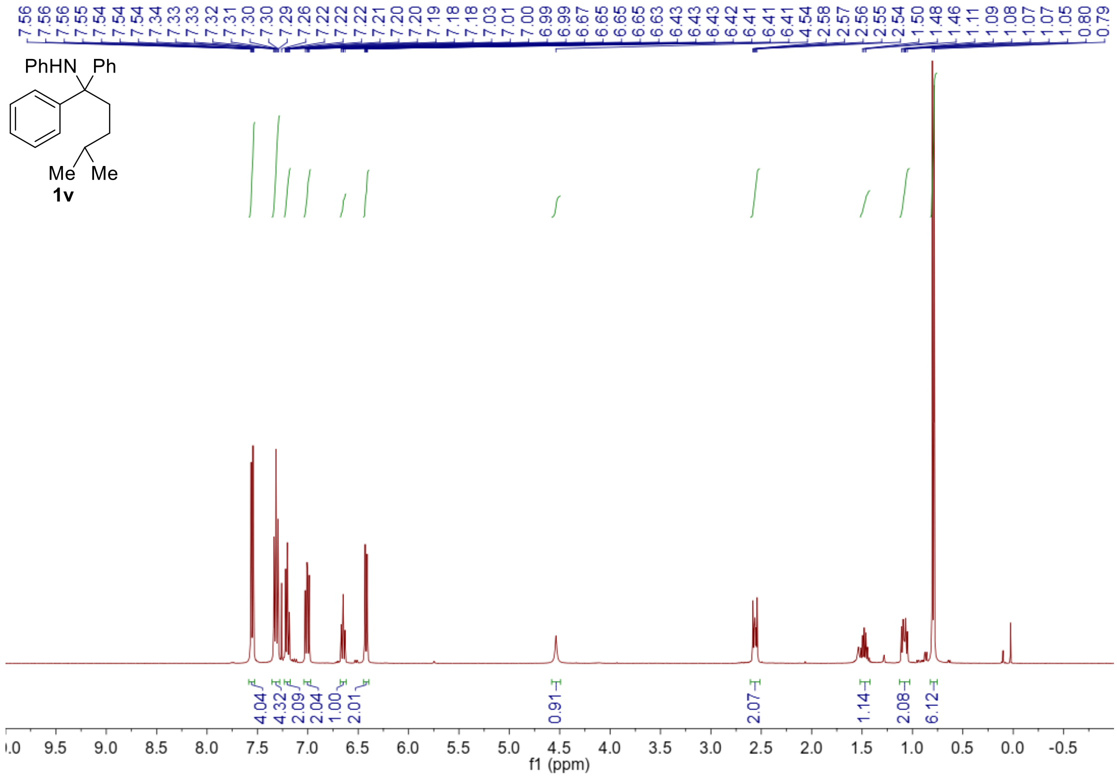
7.56 7.56 7.55 7.54 7.54 7.54 7.34 7.33 7.32 7.31 7.30 7.30 7.29 7.26 7.22 7.22 7.22 7.21 7.20 7.20 7.19 7.18 7.18 7.03 7.01 7.00 6.99 6.99 6.67 6.65 6.65 6.63 6.43 6.43 6.43 6.42 6.41 6.41 6.41 4.54 2.58 2.57 2.56 2.55 2.54 1.50 1.48 1.46 1.11 1.09 1.08 1.07 1.07 1.05 0.80 0.79
PhHN Ph
Me Me
1v
4.04 4.32 2.09 2.04 1.00 2.01 0.91 2.07 1.14 2.08 6.12
f1 (ppm)

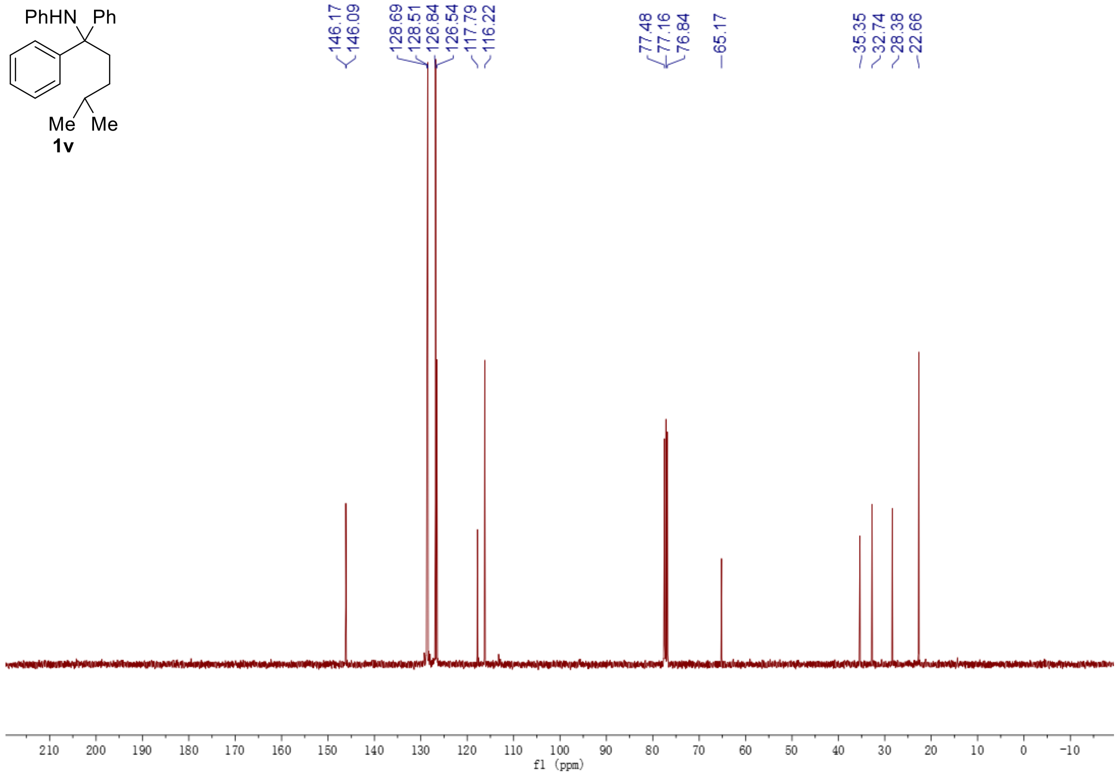
146.17 146.09 128.69 128.51 126.84 126.54 117.79 116.22 77.48 77.16 76.84 65.17 35.35 32.74 28.38 22.66
PhHN Ph
Me Me
1v
f1 (ppm)

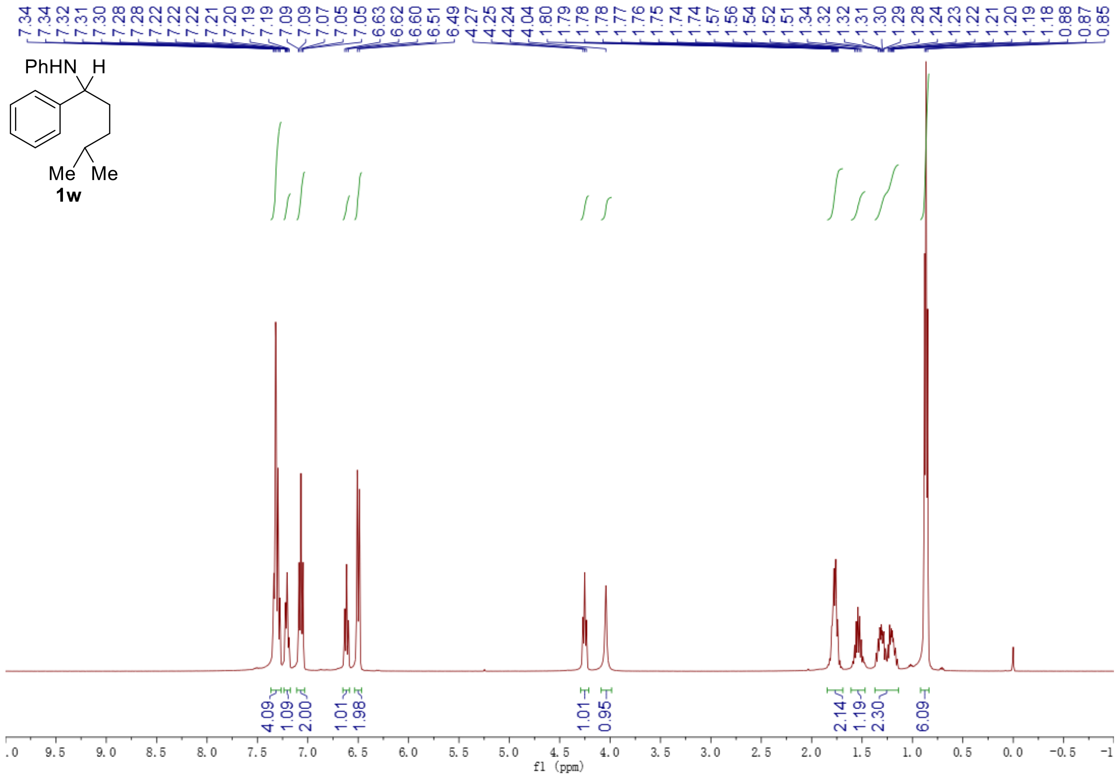
7.34 7.34 7.32 7.31 7.30 7.28 7.22 7.22 7.22 7.21 7.20 7.19 7.09 7.09 7.07 7.05 7.05 6.63 6.62 6.60 6.51 6.49 4.27 4.25 4.24 4.04 1.80 1.79 1.78 1.78 1.77 1.76 1.75 1.74 1.74 1.57 1.56 1.54 1.52 1.51 1.34 1.32 1.31 1.30 1.29 1.28 1.24 1.23 1.22 1.21 1.20 1.19 1.18 0.88 0.87 0.85
PhHN H
Me Me
1w
4.09 1.09 2.00 1.01 1.98 1.01 0.95 2.14 1.19 2.30 6.09
f1 (ppm)

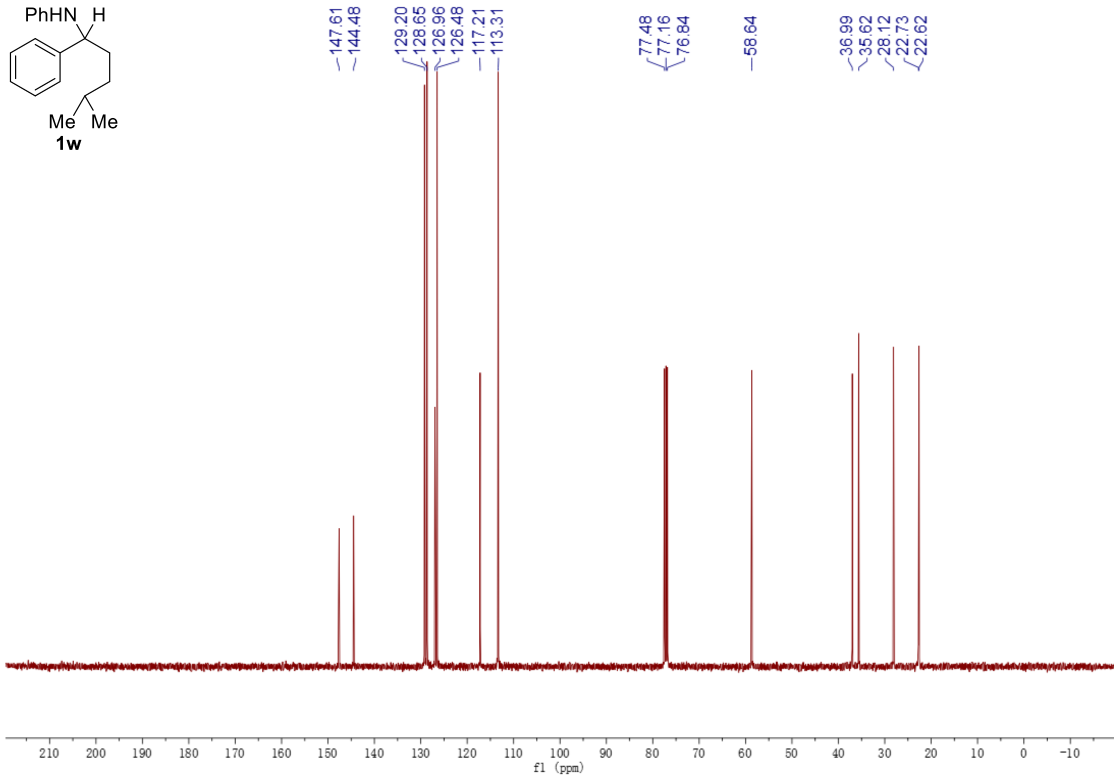
147.61 144.48 129.20 128.65 126.96 126.48 117.21 113.31 77.48 77.16 76.84 58.64 36.99 35.62 28.12 22.73 22.62
PhHN H
Me Me
1w
f1 (ppm)

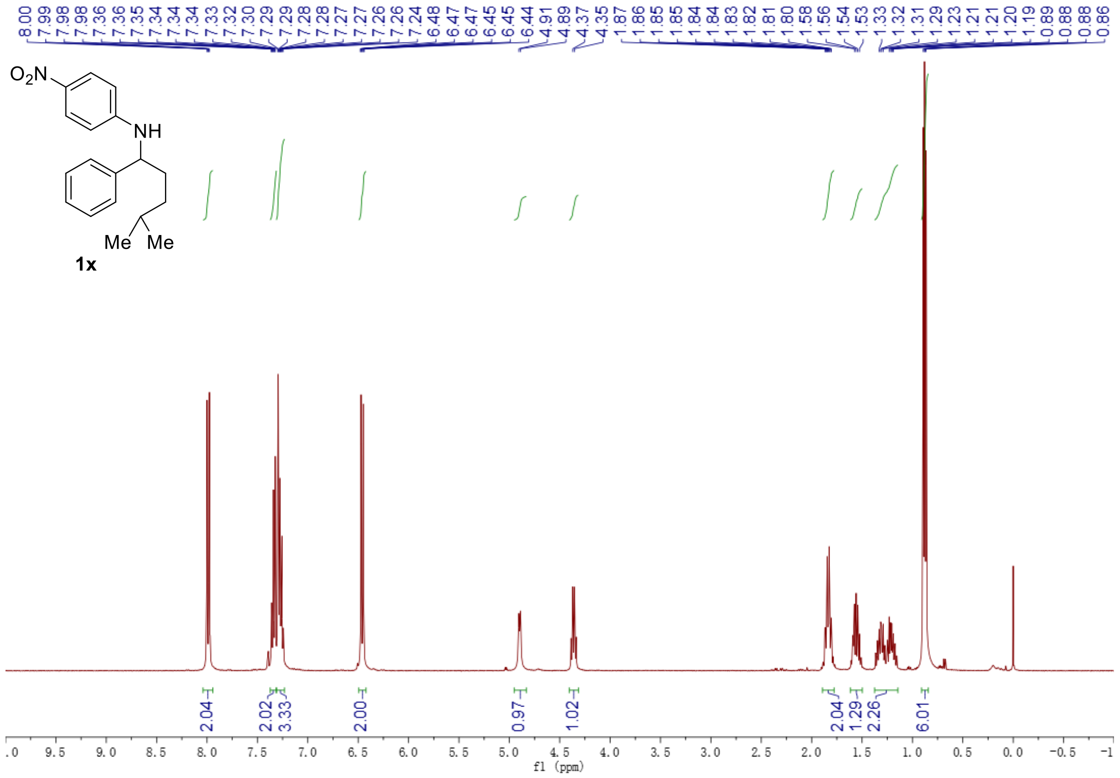

O2N
NH
Me
Me
1x
8.00
7.99
7.98
7.98
7.36
7.36
7.35
7.34
7.34
7.34
7.33
7.32
7.30
7.29
7.29
7.28
7.28
7.27
7.27
7.26
7.26
7.24
6.48
6.47
6.47
6.45
6.45
6.44
4.91
4.89
4.37
4.35
1.87
1.86
1.85
1.85
1.84
1.84
1.83
1.82
1.81
1.80
1.58
1.56
1.54
1.53
1.33
1.32
1.31
1.29
1.23
1.21
1.21
1.20
1.19
0.89
0.88
0.88
0.86
2.04
2.02
3.33
2.00
0.97
1.02
2.04
1.29
2.26
6.01
f1 (ppm)

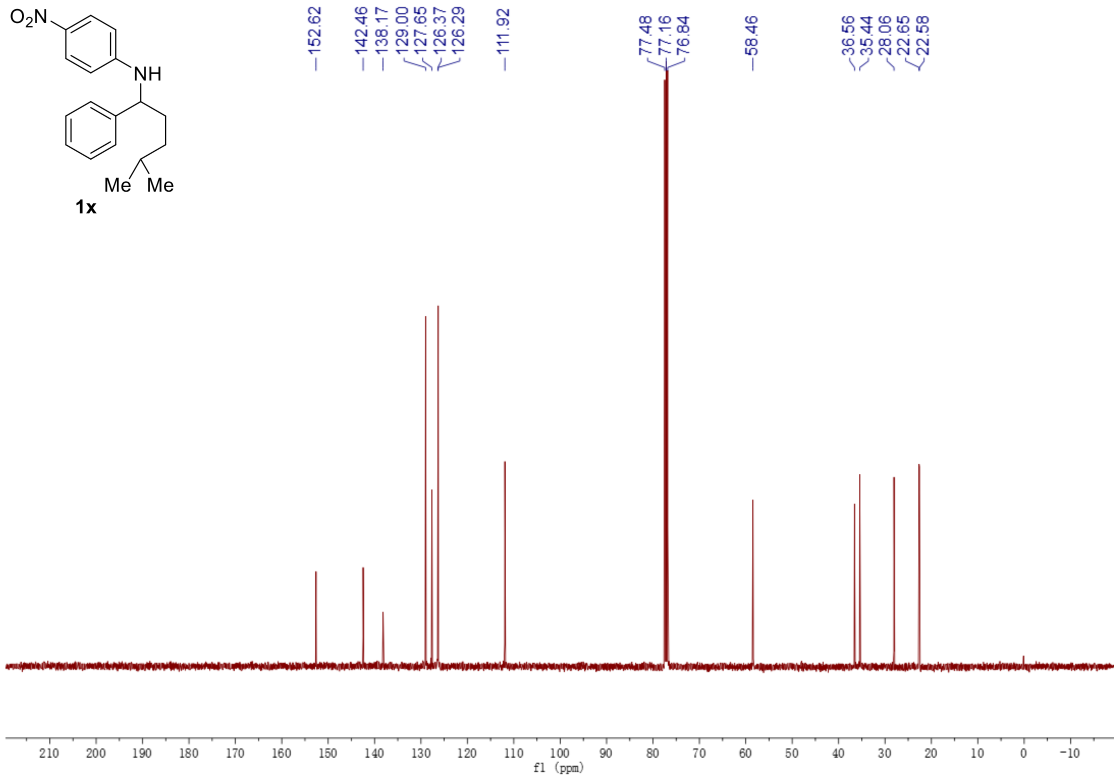

O2N
NH
Me
Me
1x
152.62
142.46
138.17
129.00
127.65
126.37
126.29
111.92
77.48
77.16
76.84
58.46
36.56
35.44
28.06
22.65
22.58
f1 (ppm)

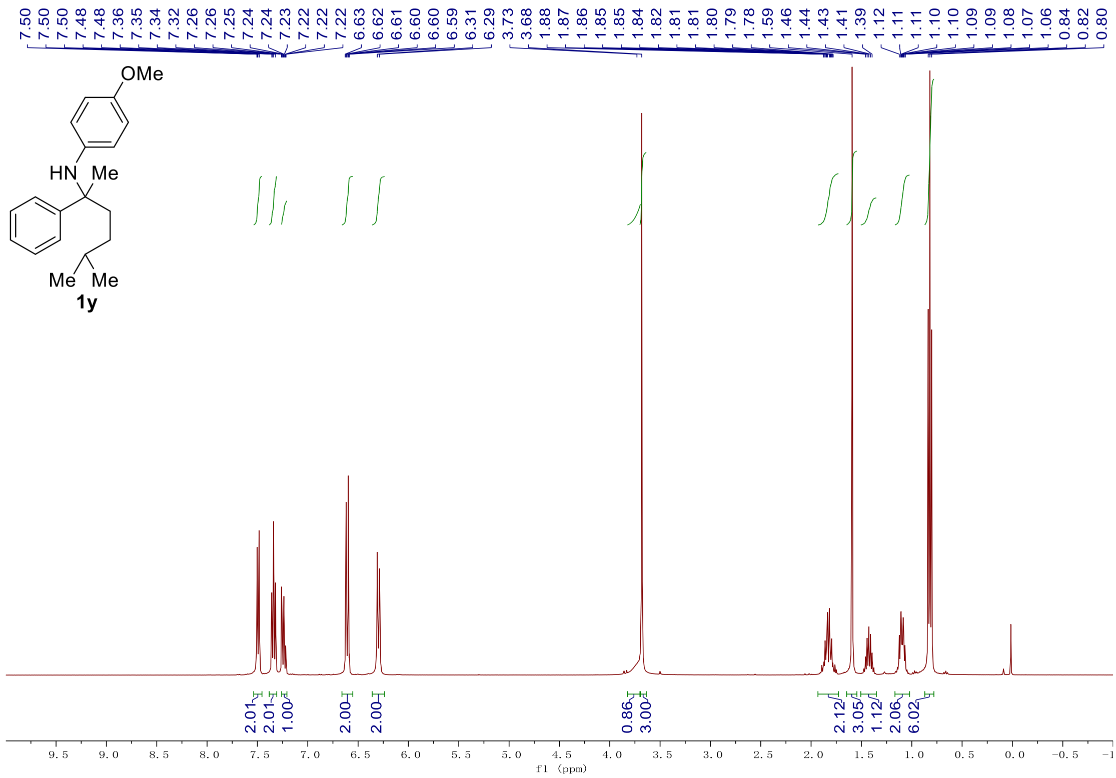
7.50 7.50 7.48 7.48 7.36 7.35 7.34 7.32 7.26 7.26 7.25 7.24 7.24 7.23 7.22 7.22 7.22 6.63 6.62 6.61 6.60 6.60 6.59 6.31 6.29 3.73 3.68 1.88 1.87 1.86 1.85 1.85 1.84 1.82 1.81 1.81 1.80 1.79 1.78 1.59 1.46 1.44 1.43 1.41 1.39 1.12 1.11 1.11 1.10 1.10 1.09 1.08 1.07 1.06 0.84 0.82 0.80
OMe
HN Me
Me Me
1y
2.01 2.01 1.00 2.00 2.00 0.86 3.00 2.12 3.05 1.12 2.06 6.02
f1 (ppm)

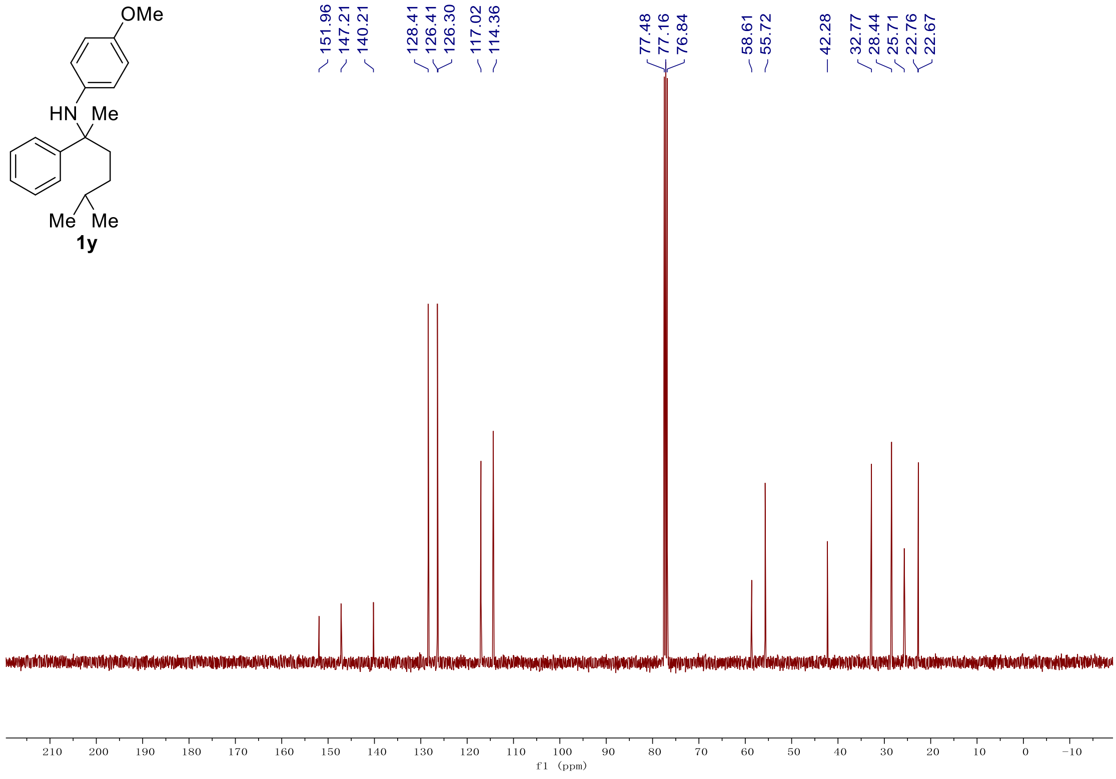
151.96 147.21 140.21 128.41 126.41 126.30 117.02 114.36 77.48 77.16 76.84 58.61 55.72 42.28 32.77 28.44 25.71 22.76 22.67
OMe
HN Me
Me Me
1y
f1 (ppm)

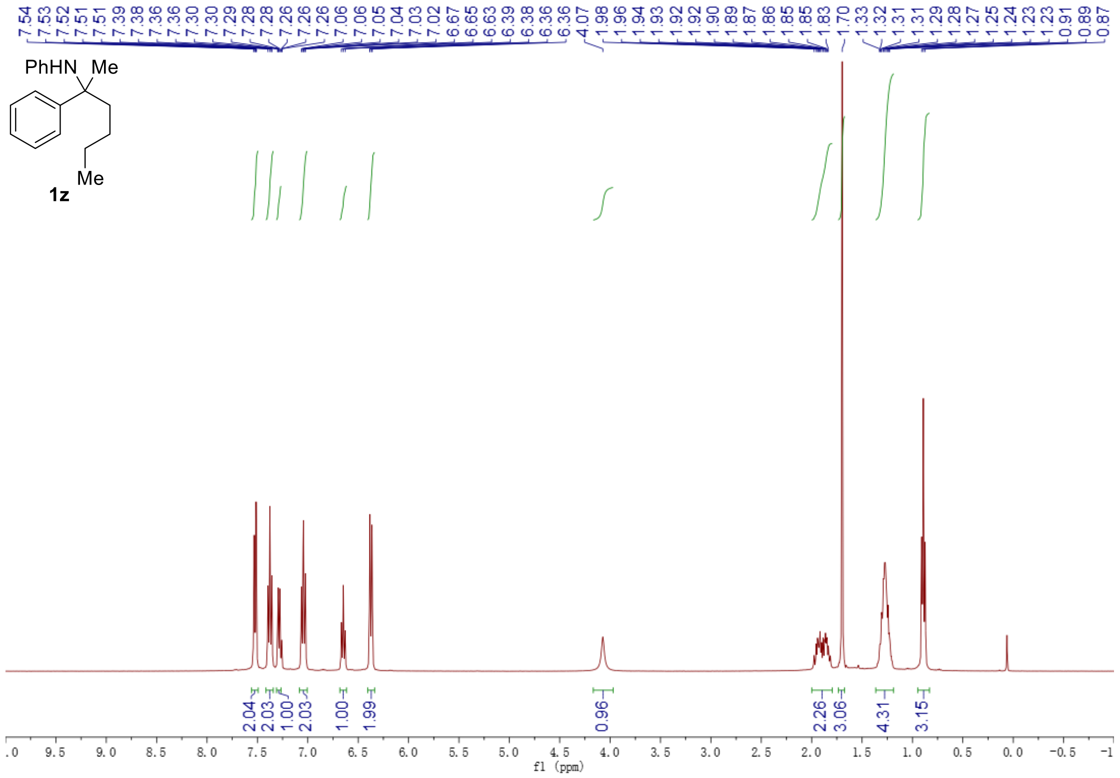
PhHN Me
Me
1z
7.54 7.53 7.52 7.51 7.51 7.39 7.38 7.36 7.36 7.36 7.30 7.29 7.28 7.28 7.26 7.26 7.06 7.06 7.05 7.04 7.03 7.02 6.67 6.65 6.63 6.39 6.38 6.36 6.36 4.07 1.98 1.96 1.94 1.93 1.92 1.92 1.90 1.89 1.87 1.86 1.85 1.85 1.83 1.70 1.33 1.32 1.31 1.31 1.29 1.28 1.27 1.25 1.24 1.23 1.23 0.91 0.89 0.87
2.04 2.03 1.00 2.03 1.00 1.99 0.96 2.26 3.06 4.31 3.15
f1 (ppm)

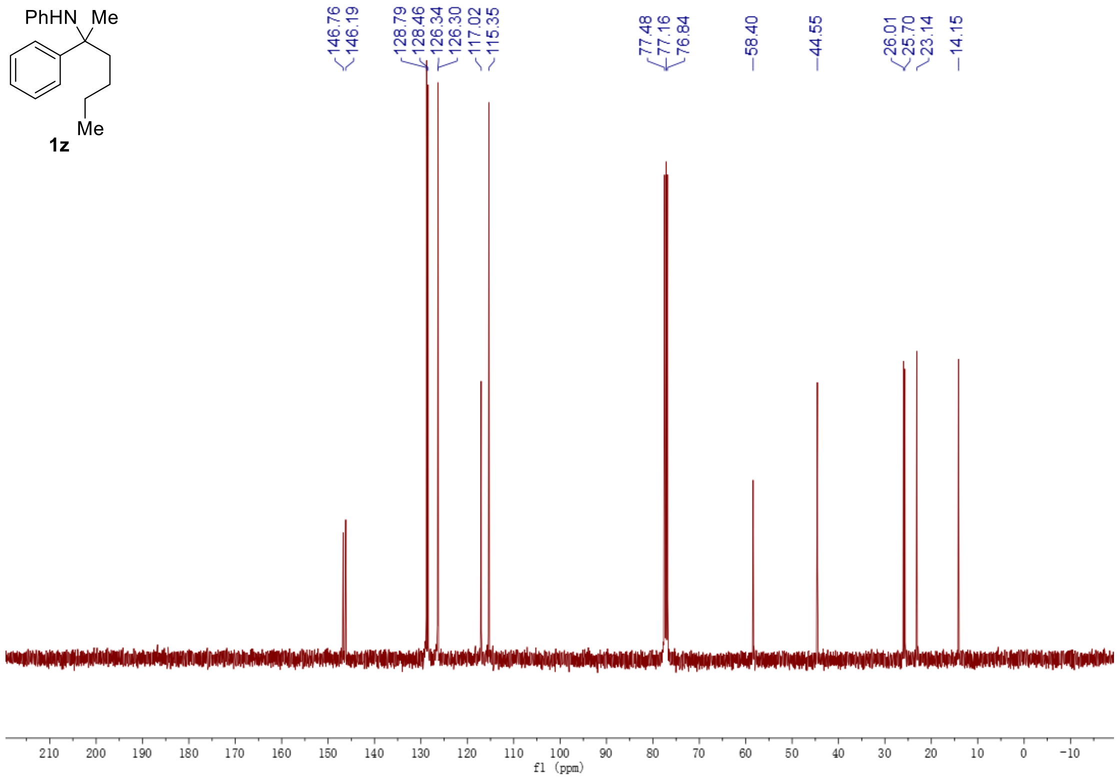
PhHN Me
Me
1z
146.76 146.19 128.79 128.46 126.34 126.30 117.02 115.35 77.48 77.16 76.84 58.40 44.55 26.01 25.70 23.14 14.15
f1 (ppm)

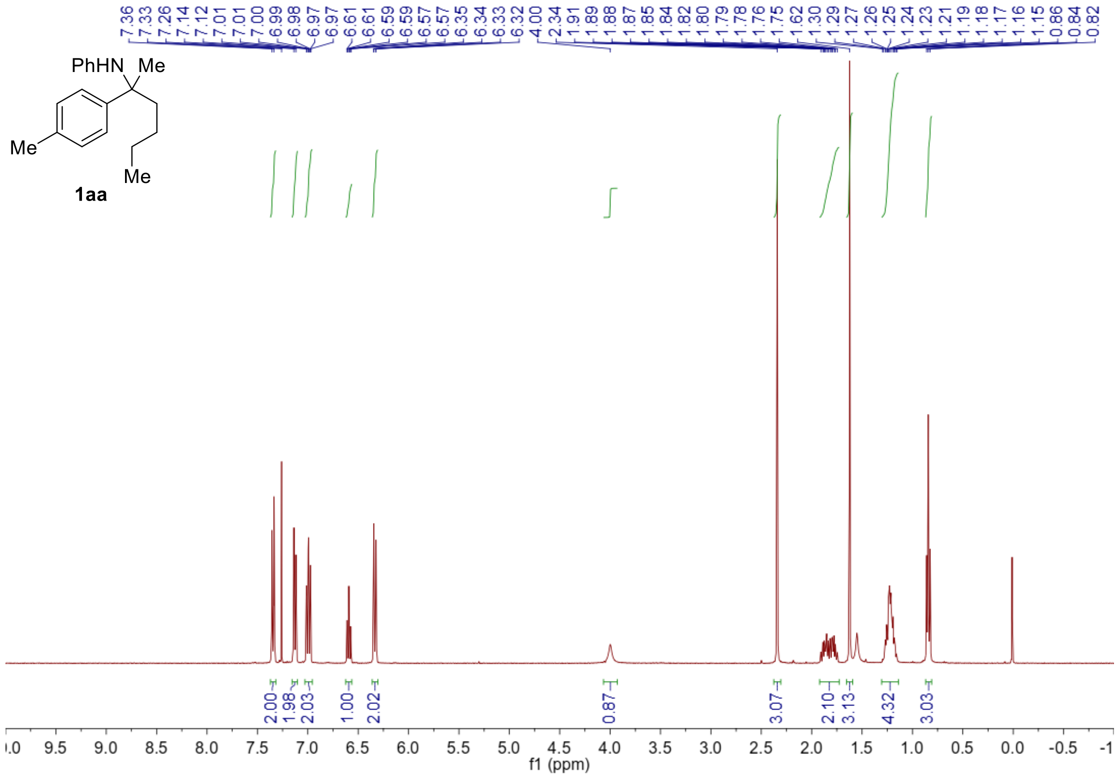
PhHN Me
Me
Me
1aa
7.36
7.33
7.26
7.14
7.12
7.01
7.00
6.99
6.98
6.97
6.61
6.61
6.59
6.59
6.57
6.57
6.35
6.34
6.33
6.32
4.00
2.34
1.91
1.89
1.88
1.87
1.85
1.84
1.82
1.80
1.79
1.78
1.76
1.75
1.62
1.30
1.29
1.27
1.26
1.25
1.24
1.23
1.21
1.19
1.18
1.17
1.16
1.15
0.86
0.84
0.82
2.00
1.98
2.03
1.00
2.02
0.87
3.07
2.10
3.13
4.32
3.03
f1 (ppm)

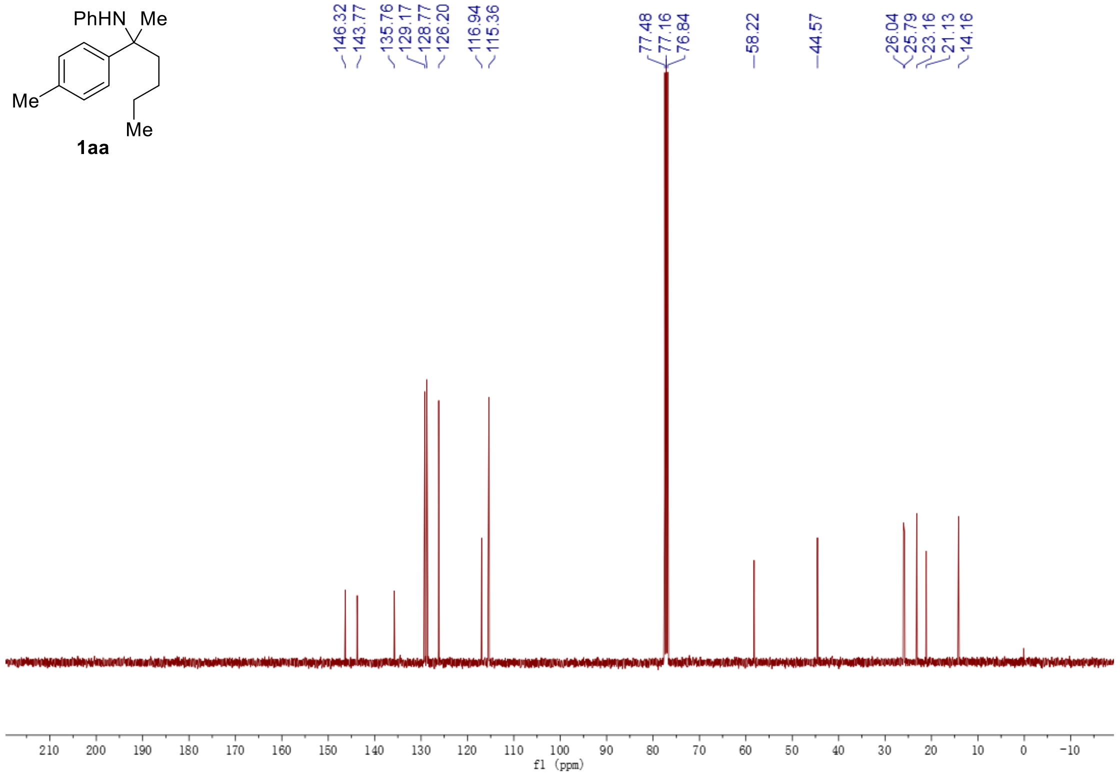
PhHN Me
Me
Me
1aa
146.32
143.77
135.76
129.17
128.77
126.20
116.94
115.36
77.48
77.16
76.84
58.22
44.57
26.04
25.79
23.16
21.13
14.16
f1 (ppm)

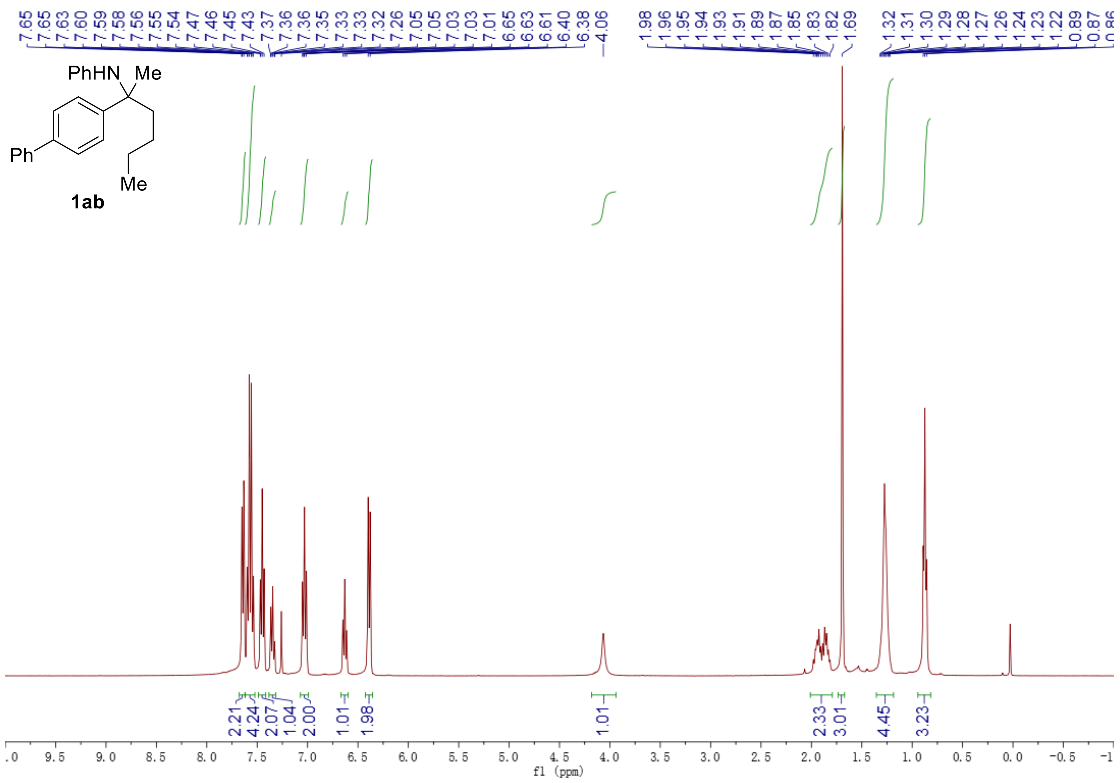

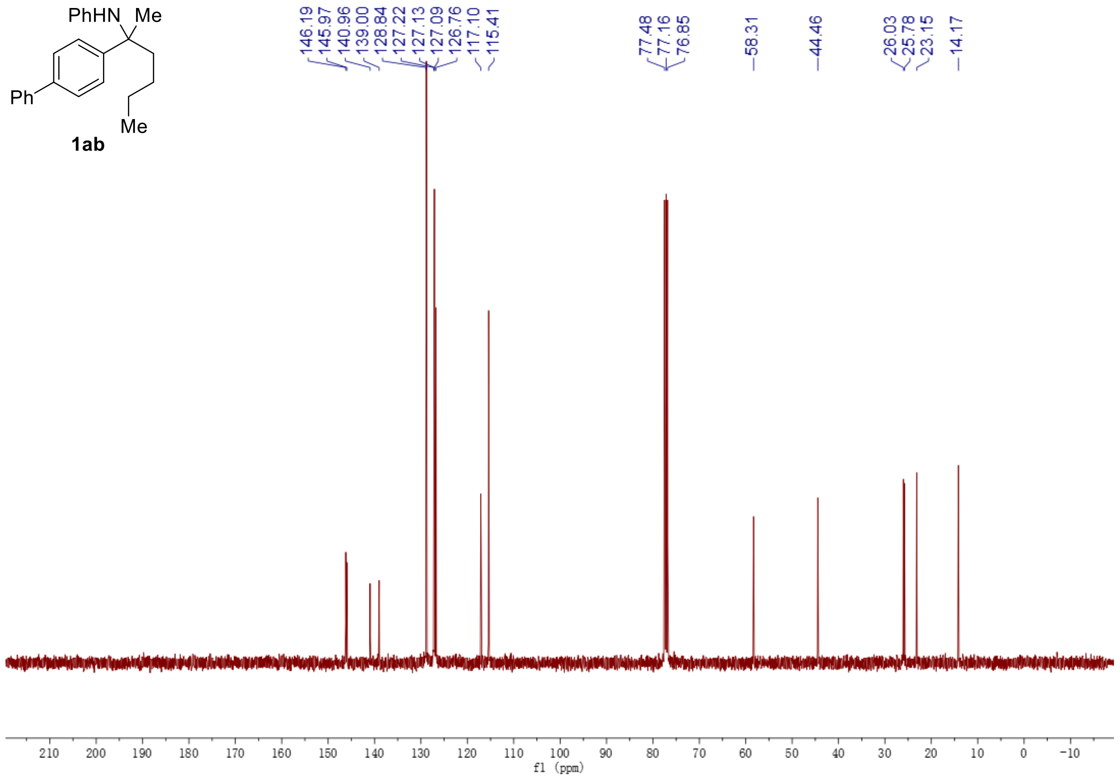

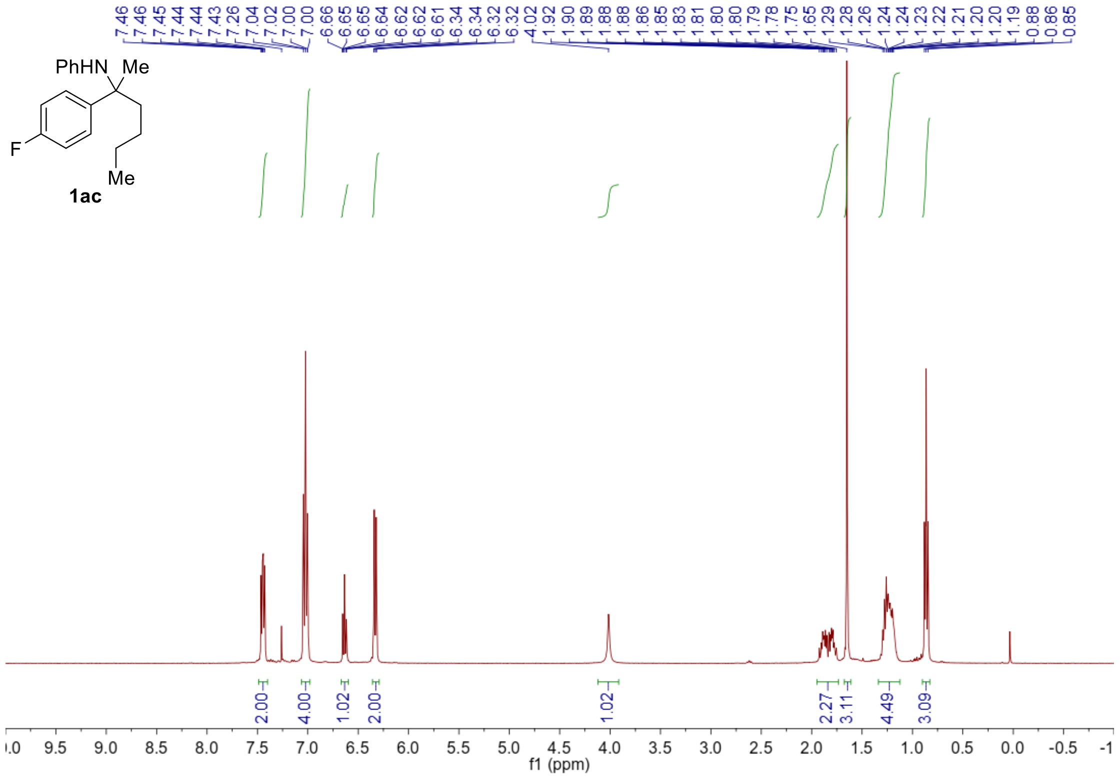
PhHN Me
F
Me
1ac
7.46 7.46 7.45 7.44 7.44 7.43 7.26 7.04 7.02 7.00 7.00 6.66 6.65 6.65 6.64 6.62 6.62 6.61 6.34 6.34 6.32 6.32 4.02 1.92 1.90 1.89 1.88 1.88 1.86 1.85 1.83 1.81 1.80 1.80 1.79 1.78 1.75 1.65 1.29 1.28 1.26 1.24 1.24 1.23 1.22 1.21 1.20 1.20 1.19 0.88 0.86 0.85
2.00 4.00 1.02 2.00 1.02 2.27 3.11 4.49 3.09
f1 (ppm)

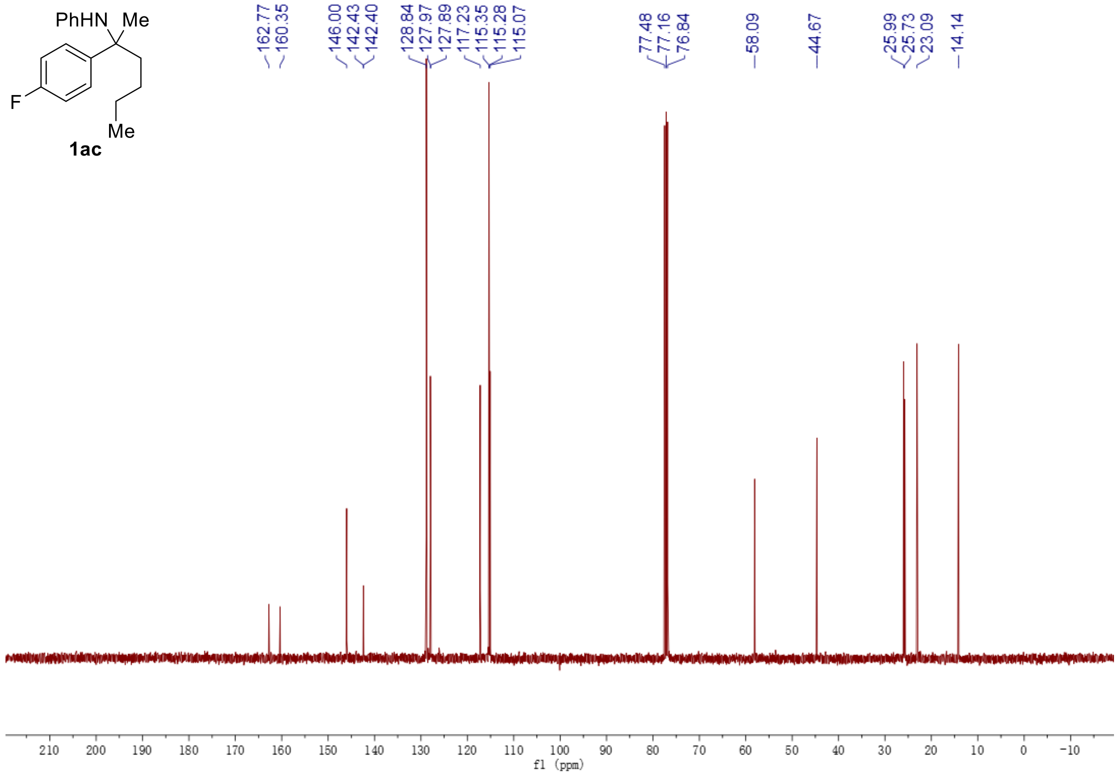
PhHN Me
F
Me
1ac
162.77 160.35 146.00 142.43 142.40 128.84 127.97 127.89 117.23 115.35 115.28 115.07 77.48 77.16 76.84 58.09 44.67 25.99 25.73 23.09 14.14
f1 (ppm)

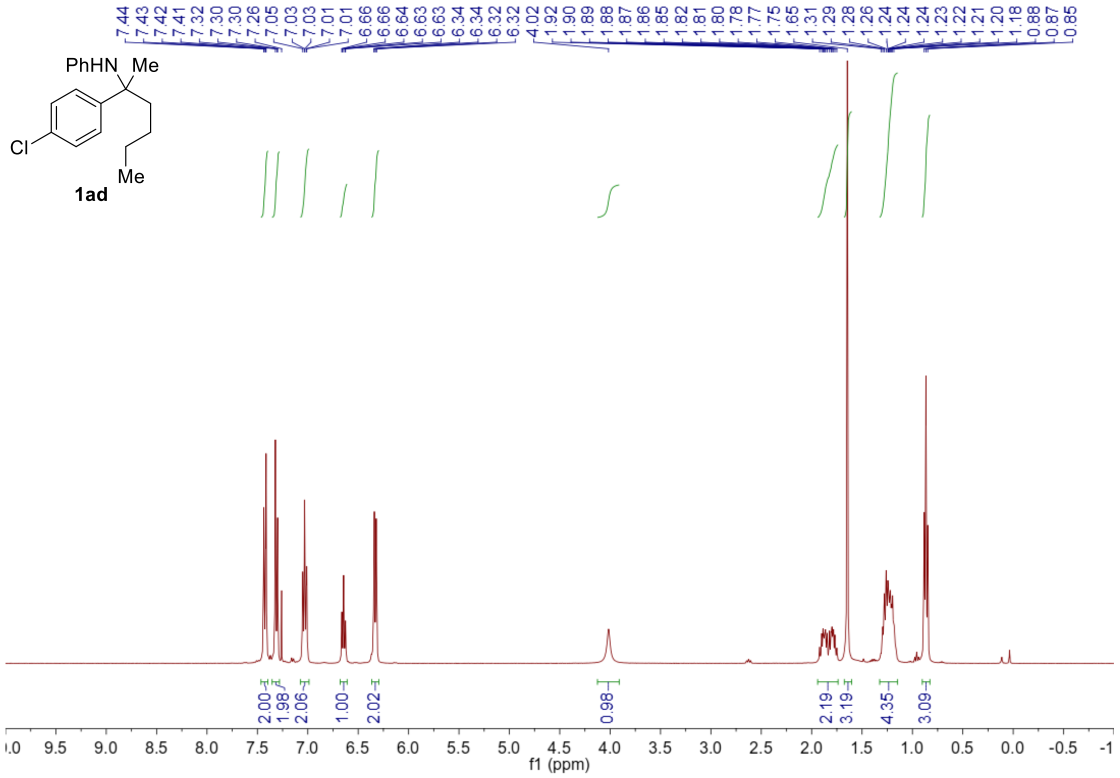
7.44
7.43
7.42
7.41
7.32
7.30
7.30
7.26
7.05
7.03
7.03
7.01
6.66
6.66
6.64
6.63
6.63
6.34
6.34
6.32
6.32
4.02
1.92
1.90
1.89
1.88
1.87
1.86
1.85
1.82
1.81
1.80
1.78
1.77
1.75
1.65
1.31
1.29
1.28
1.26
1.24
1.24
1.24
1.23
1.22
1.21
1.20
1.18
0.88
0.87
0.85
PhHN
Me
Cl
Me
1ad
2.00
1.98
2.06
1.00
2.02
0.98
2.19
3.19
4.35
3.09
f1 (ppm)

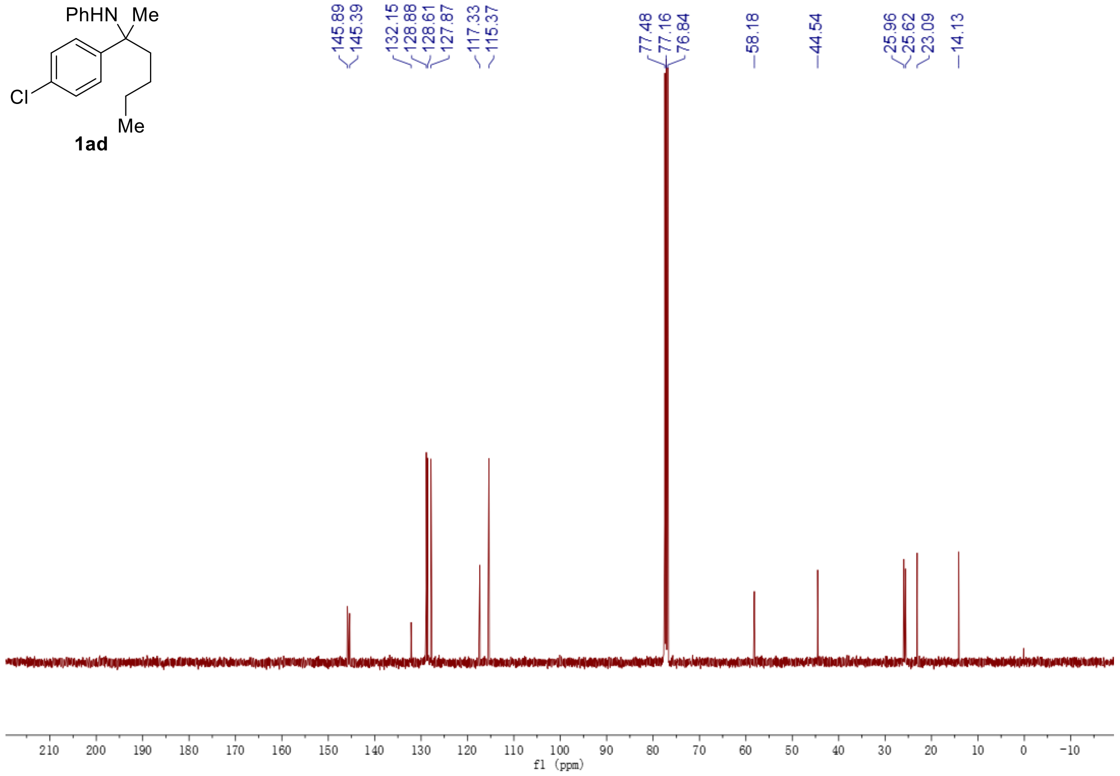
145.89
145.39
132.15
128.88
128.61
127.87
117.33
115.37
77.48
77.16
76.84
58.18
44.54
25.96
25.62
23.09
14.13
PhHN
Me
Cl
Me
1ad
f1 (ppm)

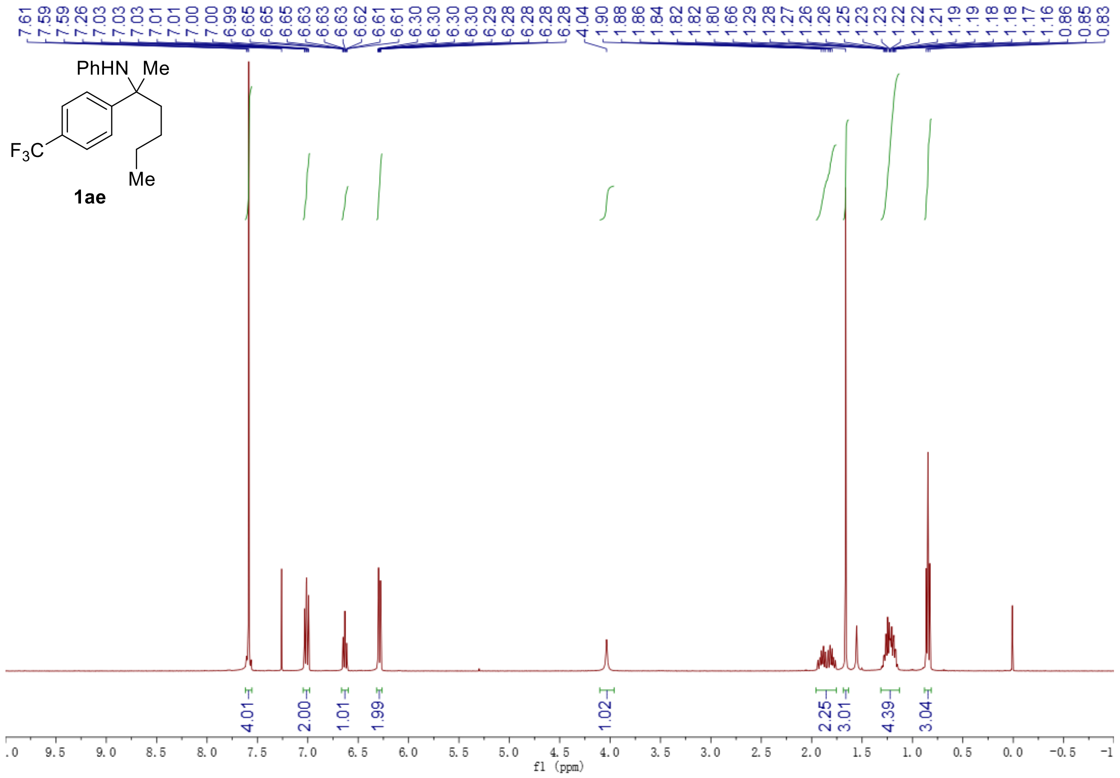
PhHN
Me
F3C
Me
1ae
7.61
7.59
7.59
7.26
7.03
7.03
7.03
7.01
7.01
7.00
7.00
6.99
6.65
6.65
6.65
6.63
6.63
6.63
6.62
6.61
6.61
6.30
6.30
6.30
6.30
6.29
6.28
6.28
6.28
6.28
4.04
1.90
1.88
1.86
1.84
1.82
1.82
1.80
1.66
1.29
1.28
1.27
1.26
1.26
1.25
1.23
1.23
1.22
1.22
1.21
1.19
1.19
1.18
1.18
1.17
1.16
0.86
0.85
0.83
4.01
2.00
1.01
1.99
1.02
2.25
3.01
4.39
3.04
f1 (ppm)
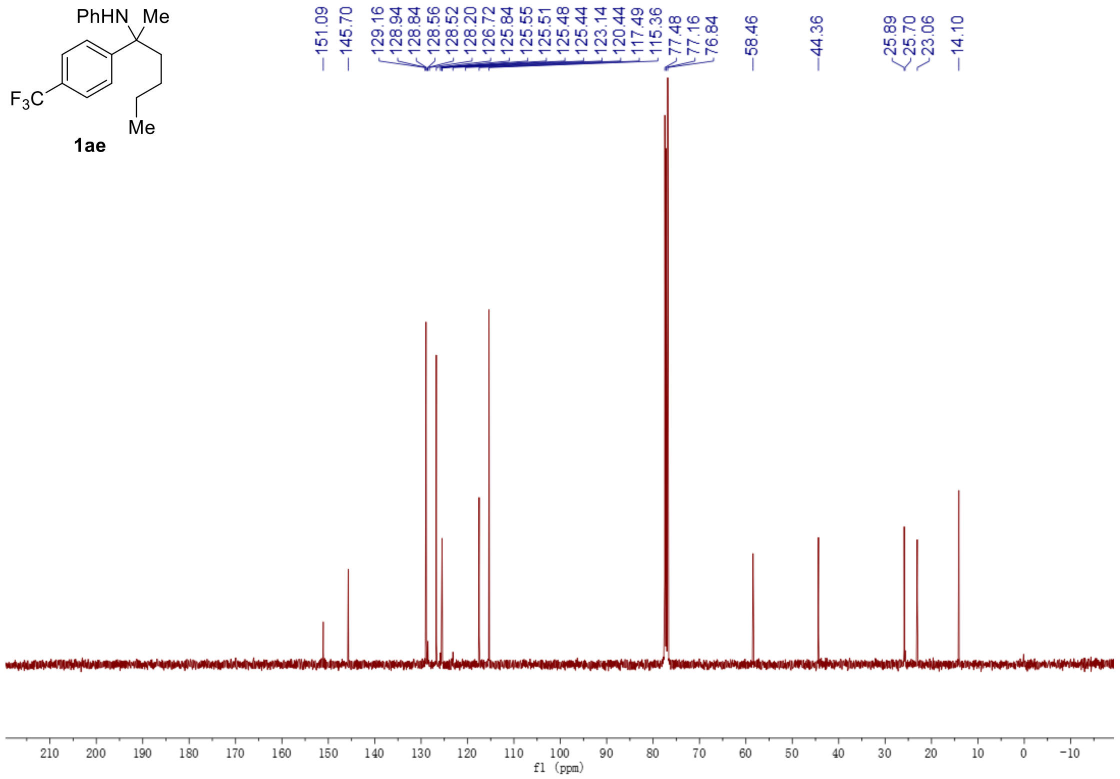
PhHN
Me
F3C
Me
1ae
151.09
145.70
129.16
128.94
128.84
128.56
128.52
128.20
126.72
125.84
125.55
125.51
125.48
125.44
123.14
120.44
117.49
115.36
77.48
77.16
76.84
58.46
44.36
25.89
25.70
23.06
14.10
f1 (ppm)

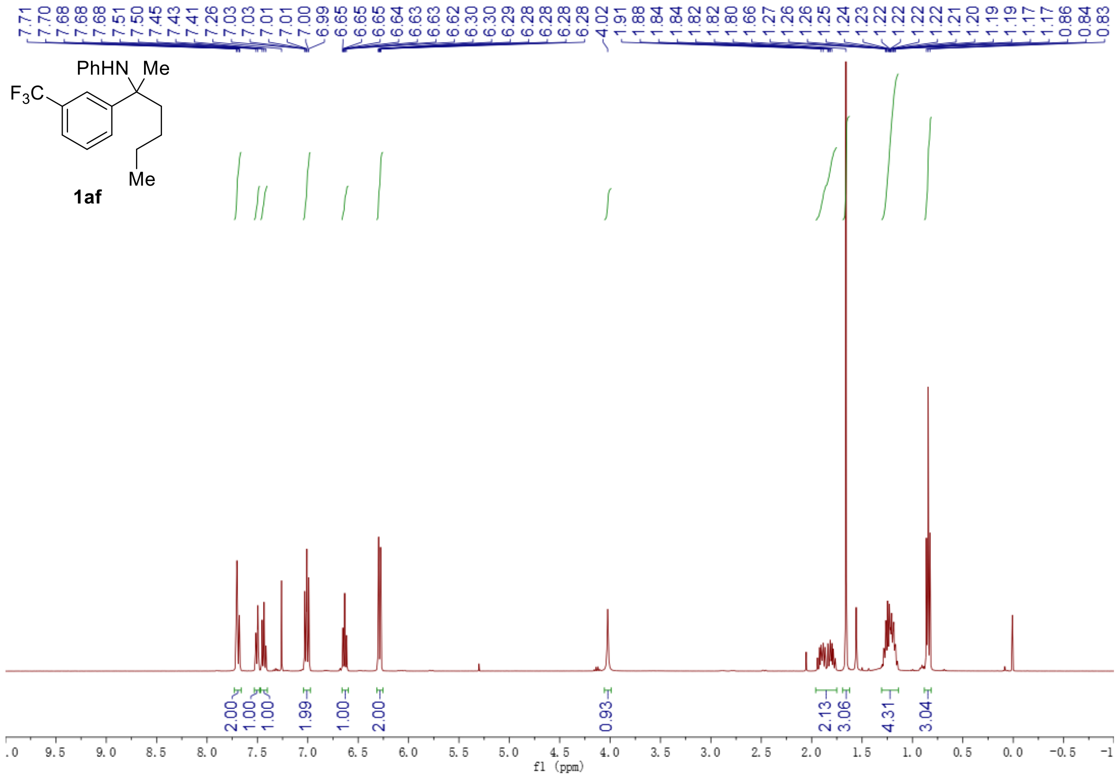
PhHN Me
F3C
Me
1af
f1 (ppm)

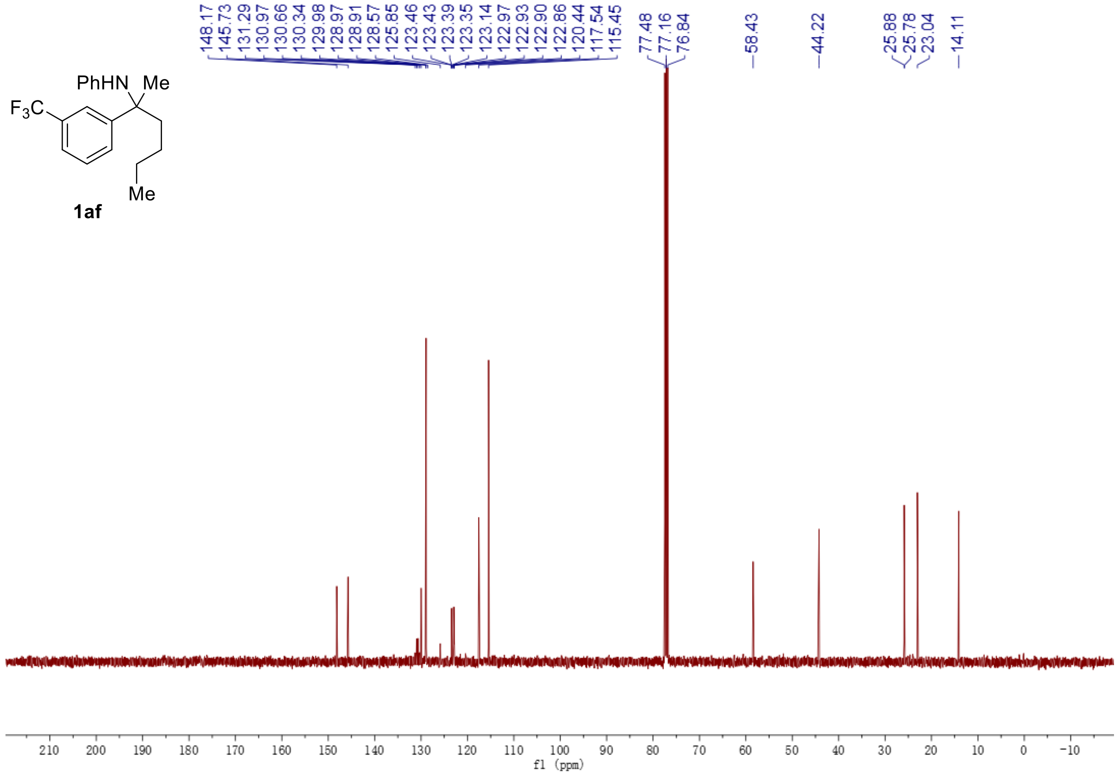
PhHN Me
F3C
Me
1af
f1 (ppm)

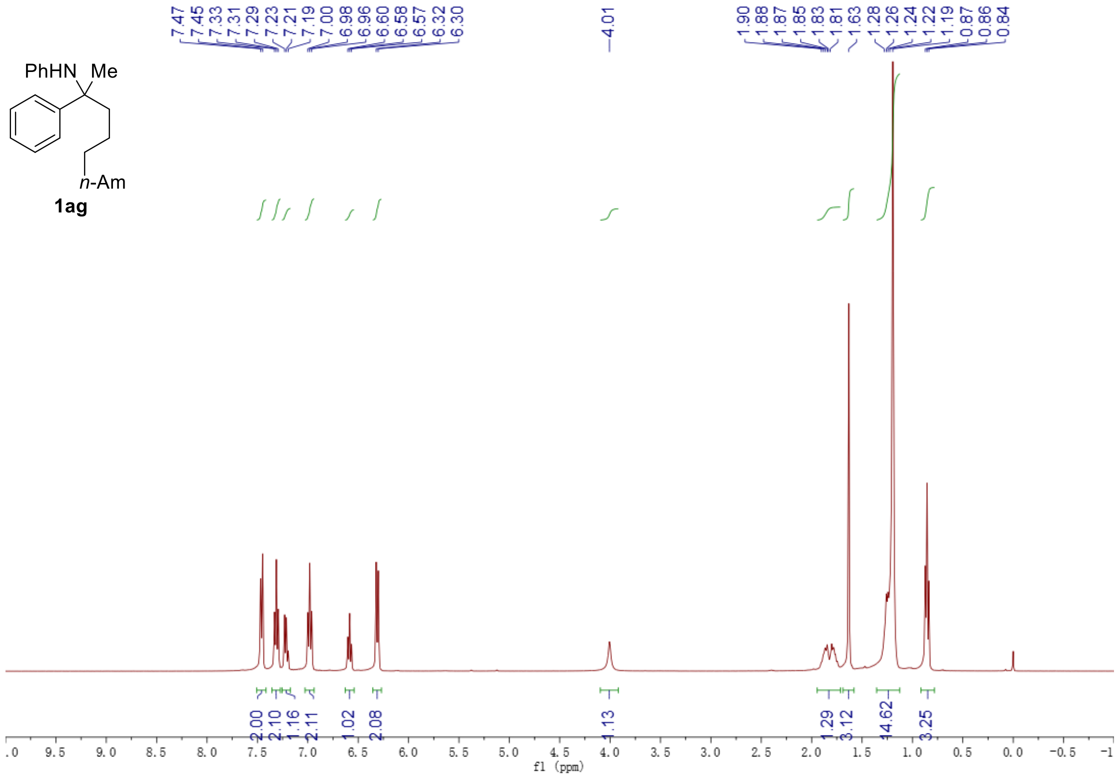
PhHN Me
n-Am
1ag
7.47 7.45 7.33 7.31 7.29 7.23 7.21 7.19 7.00 6.98 6.96 6.60 6.58 6.57 6.32 6.30
4.01
1.90 1.88 1.87 1.85 1.83 1.81 1.63 1.28 1.26 1.24 1.22 1.19 0.87 0.86 0.84
2.00 2.10 1.16 2.11 1.02 2.08 1.13 1.29 3.12 14.62 3.25
f1 (ppm)

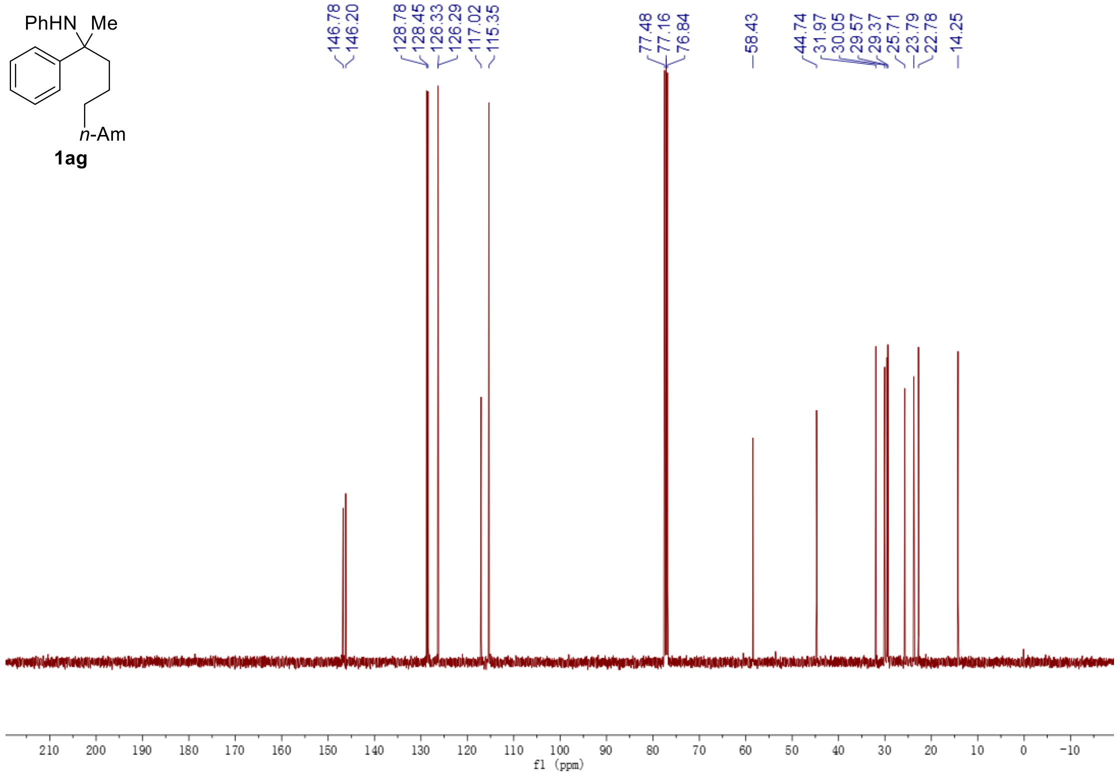
PhHN Me
n-Am
1ag
146.78 146.20 128.78 128.45 126.33 126.29 117.02 115.35
77.48 77.16 76.84
58.43
44.74 31.97 30.05 29.57 29.37 25.71 23.79 22.78 14.25
f1 (ppm)

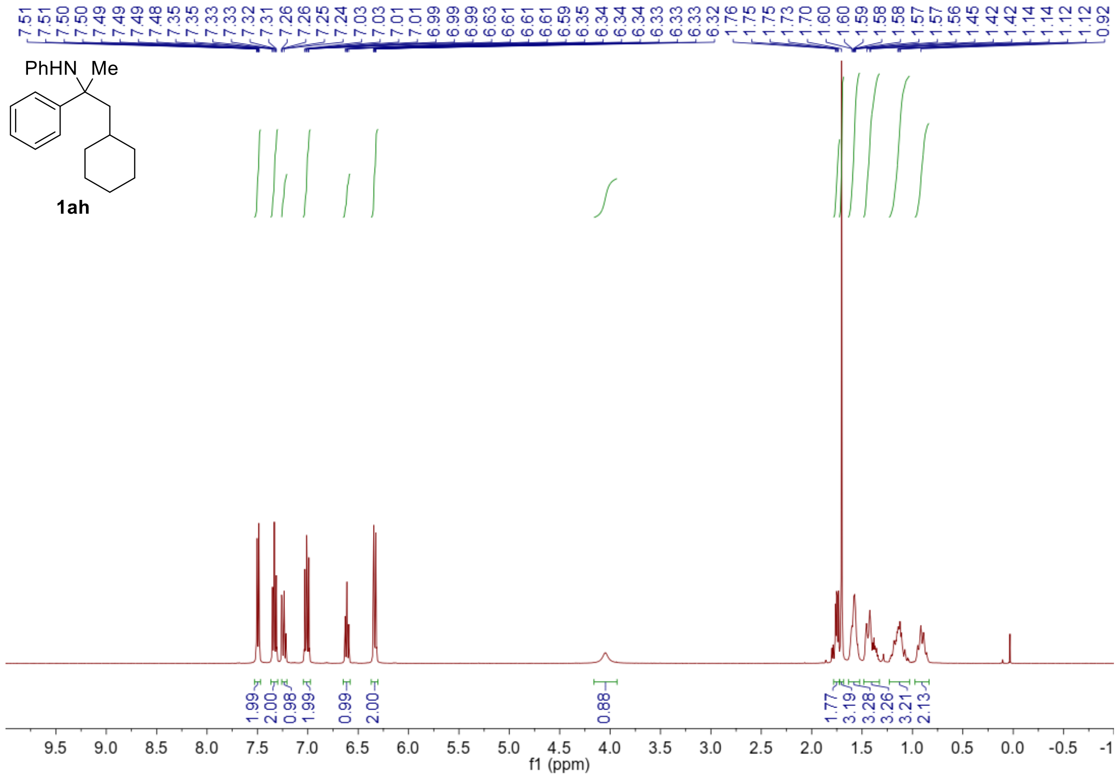
PhHN Me
1ah
f1 (ppm)

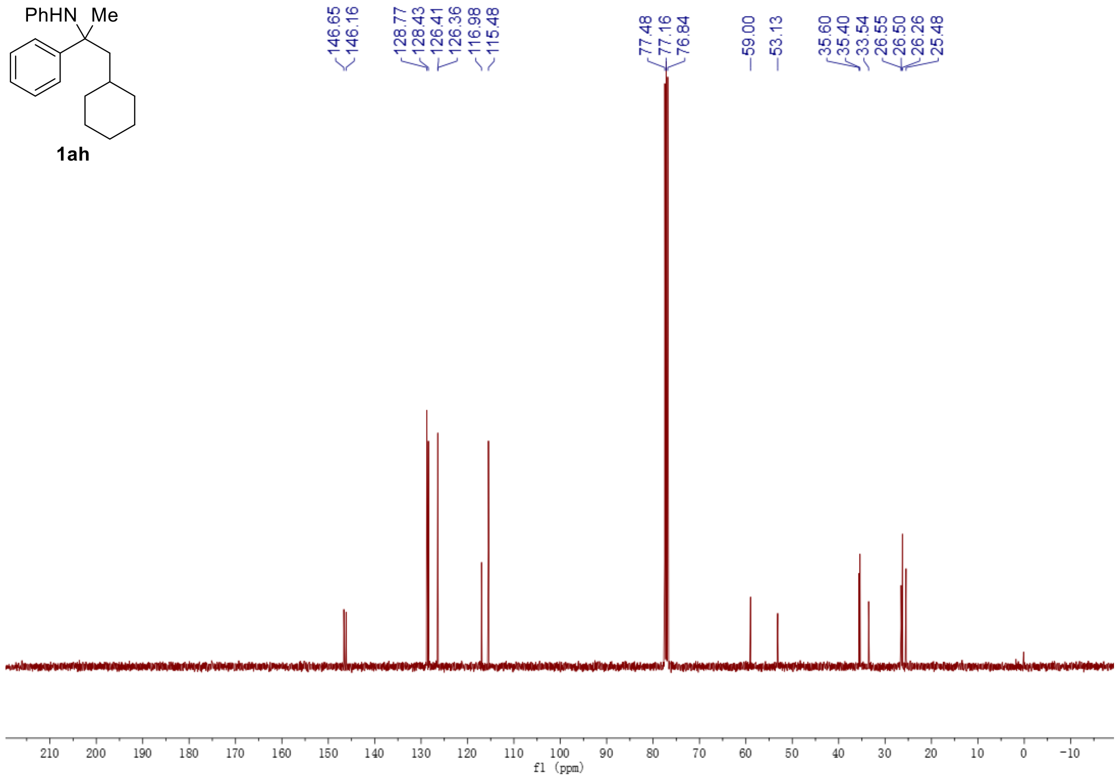
PhHN Me
1ah
146.65
146.16
128.77
128.43
126.41
126.36
116.98
115.48
77.48
77.16
76.84
59.00
53.13
35.60
35.40
33.54
26.55
26.50
26.26
25.48
f1 (ppm)

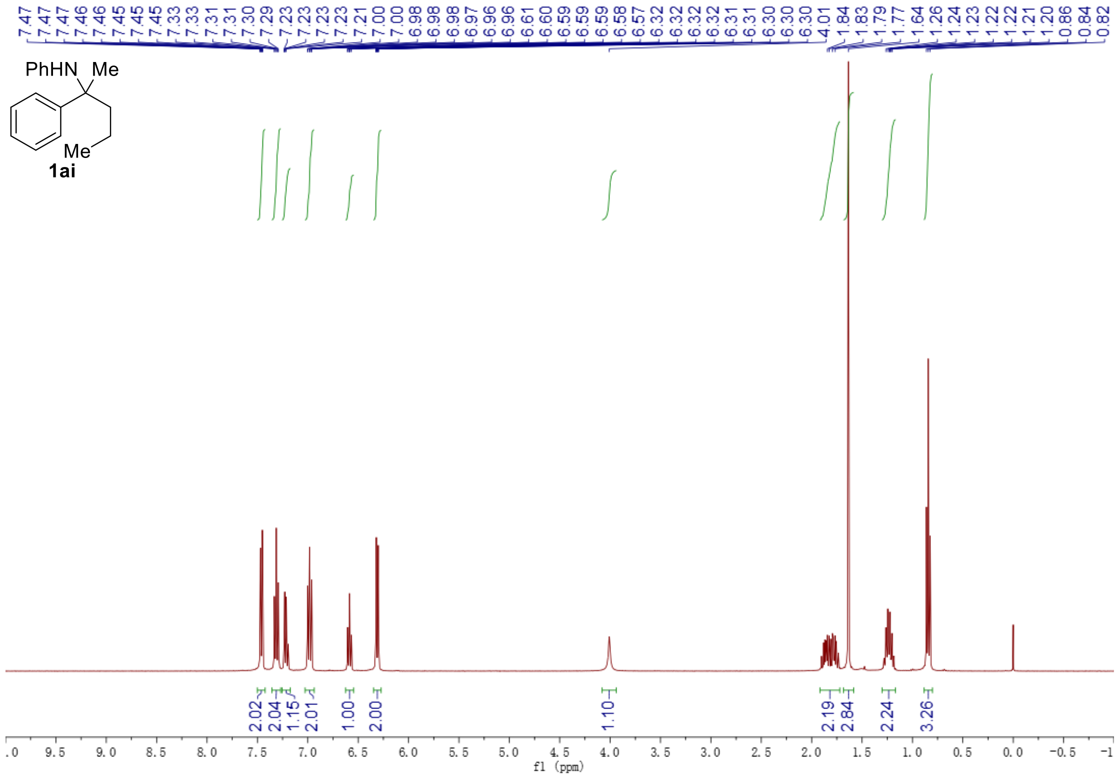
PhHN Me
Me
1ai
7.47
7.47
7.46
7.46
7.45
7.45
7.33
7.33
7.31
7.31
7.30
7.29
7.23
7.23
7.23
7.23
7.21
7.00
7.00
6.98
6.98
6.97
6.96
6.96
6.61
6.60
6.59
6.59
6.59
6.58
6.57
6.32
6.32
6.32
6.32
6.31
6.31
6.30
6.30
6.30
4.01
1.84
1.83
1.79
1.77
1.64
1.26
1.24
1.23
1.22
1.22
1.21
1.20
0.86
0.84
0.82
2.02
2.04
1.15
2.01
1.00
2.00
1.10
2.19
2.84
2.24
3.26
f1 (ppm)

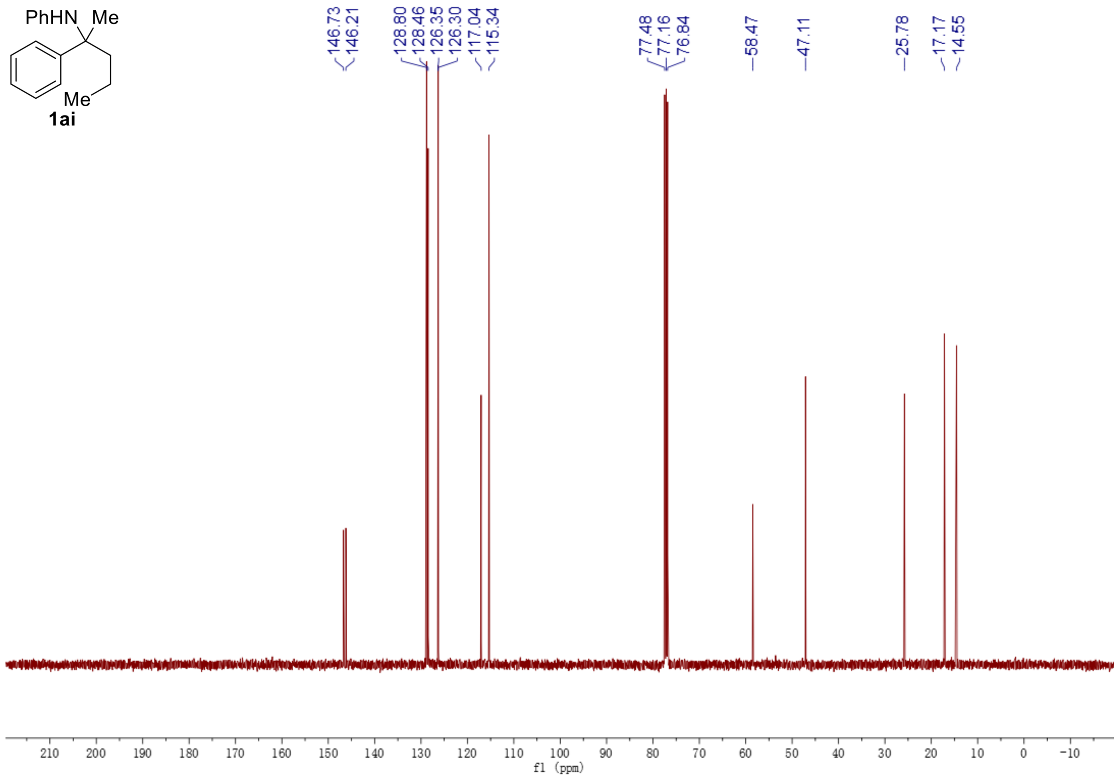
PhHN Me
Me
1ai
146.73
146.21
128.80
128.46
126.35
126.30
117.04
115.34
77.48
77.16
76.84
58.47
47.11
25.78
17.17
14.55
f1 (ppm)

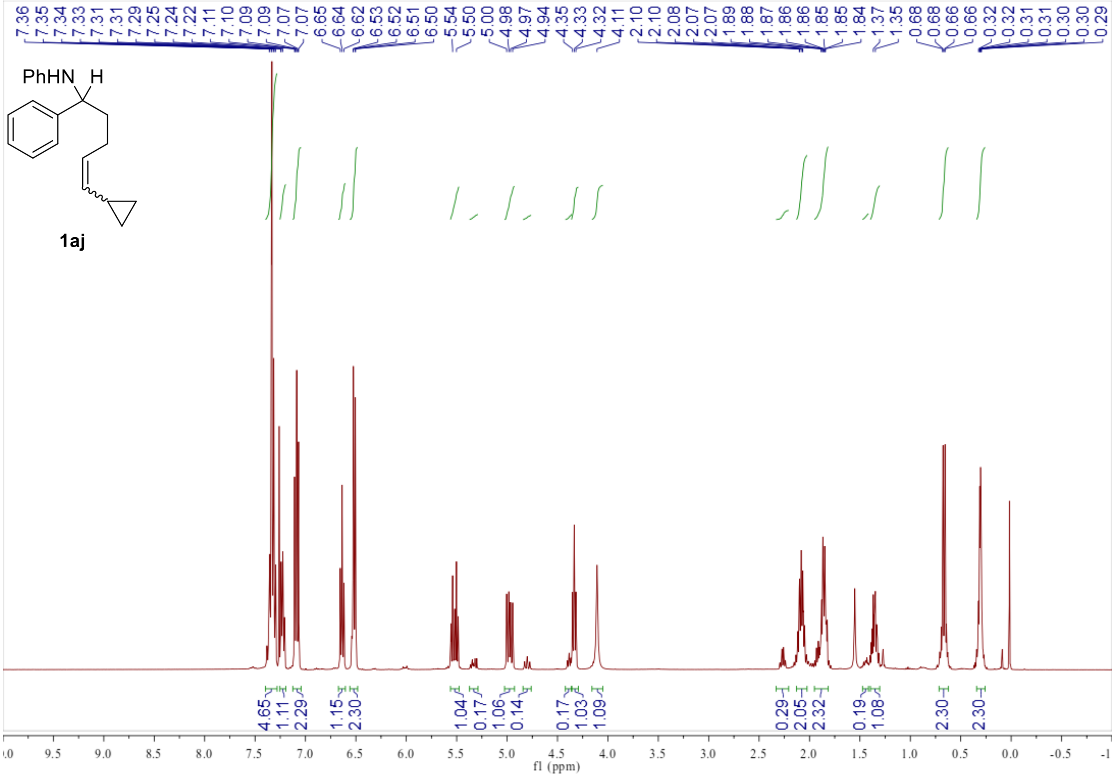
7.36 7.35 7.34 7.33 7.31 7.29 7.25 7.24 7.22 7.11 7.10 7.09 7.09 7.07 7.07 6.65 6.64 6.62 6.53 6.52 6.51 6.50 5.54 5.50 5.00 4.98 4.97 4.94 4.35 4.33 4.32 4.11 2.10 2.08 2.07 2.07 1.89 1.88 1.87 1.86 1.86 1.85 1.85 1.84 1.37 1.35 0.68 0.68 0.66 0.66 0.32 0.32 0.31 0.30 0.30 0.29
PhHN H
1aj
4.65 1.11 2.29 1.15 2.30 1.04 0.17 1.06 0.14 0.17 1.03 1.09 0.29 2.05 2.32 0.19 1.08 2.30 2.30
f1 (ppm)

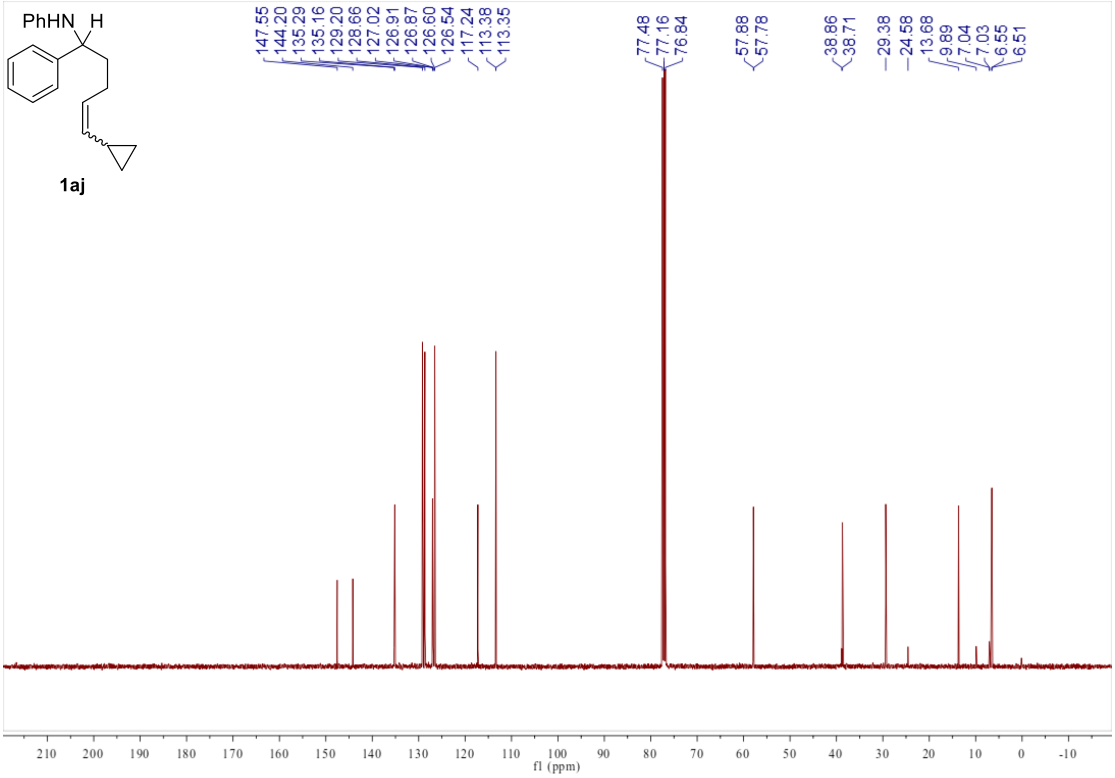
147.55 144.20 135.29 135.16 129.20 128.66 127.02 126.91 126.87 126.60 126.54 117.24 113.38 113.35
77.48 77.16 76.84
57.88 57.78
38.86 38.71
29.38 24.58 13.68 9.89 7.04 7.03 6.55 6.51
PhHN H
1aj
f1 (ppm)

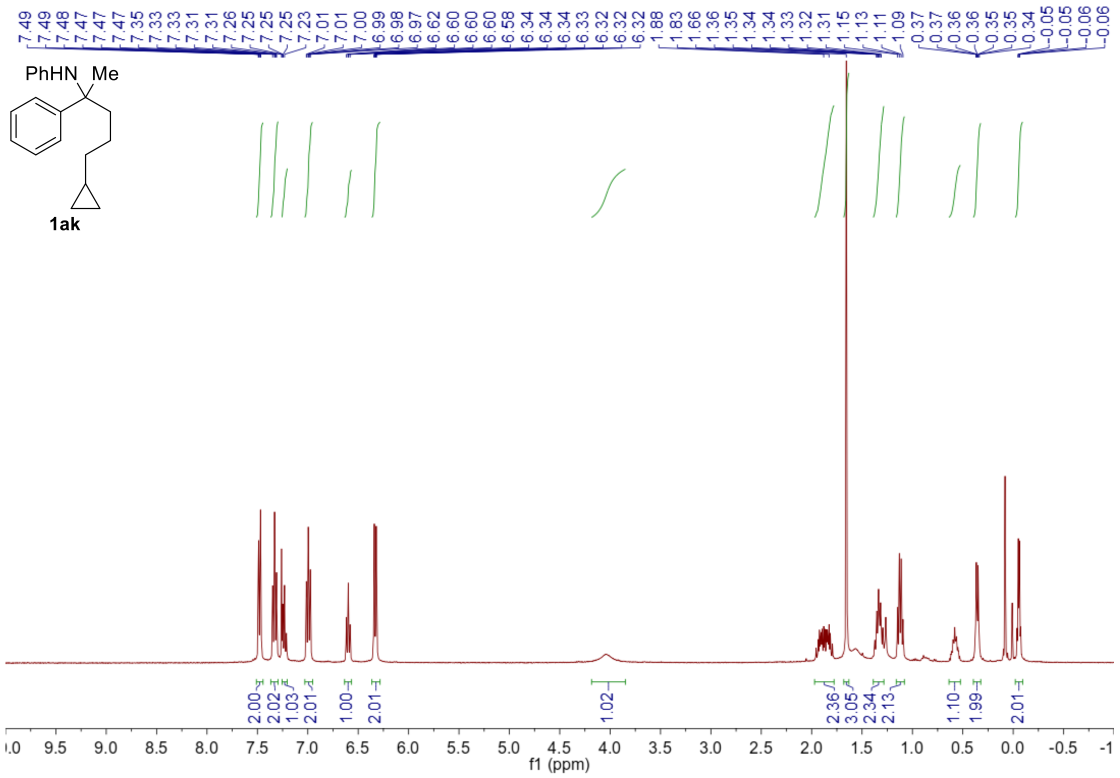
PhHN Me
1ak
f1 (ppm)

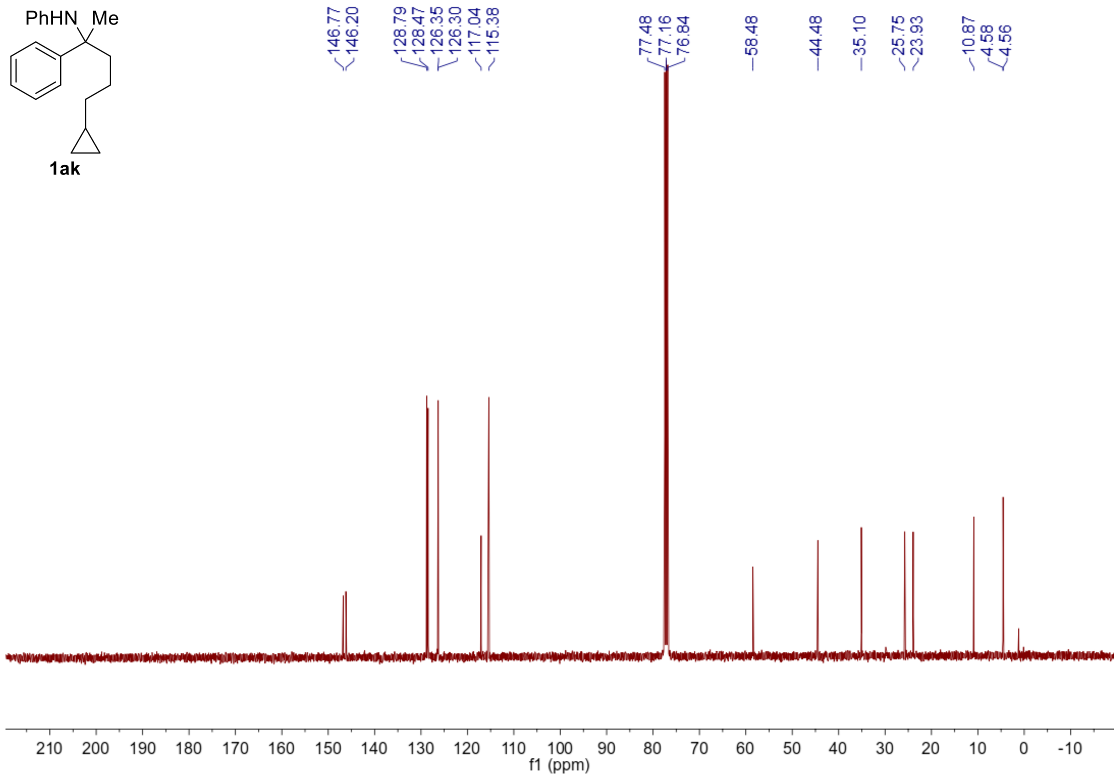
PhHN Me
1ak
f1 (ppm)

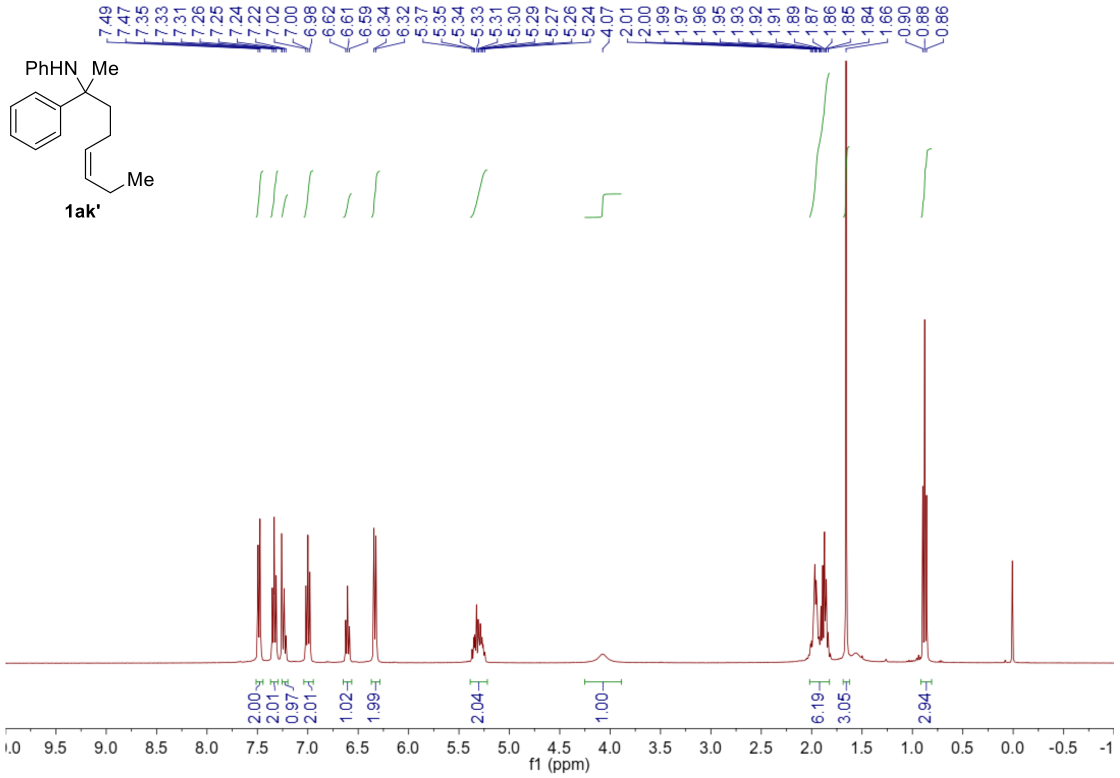
PhHN Me
Me
1ak'
7.49
7.47
7.35
7.33
7.31
7.26
7.25
7.24
7.22
7.02
7.00
6.98
6.62
6.61
6.59
6.34
6.32
5.37
5.35
5.34
5.33
5.31
5.30
5.29
5.27
5.26
5.24
4.07
2.01
2.00
1.99
1.97
1.96
1.95
1.93
1.92
1.91
1.89
1.87
1.86
1.85
1.84
1.66
0.90
0.88
0.86
2.00
2.01
0.97
2.01
1.02
1.99
2.04
1.00
6.19
3.05
2.94
f1 (ppm)

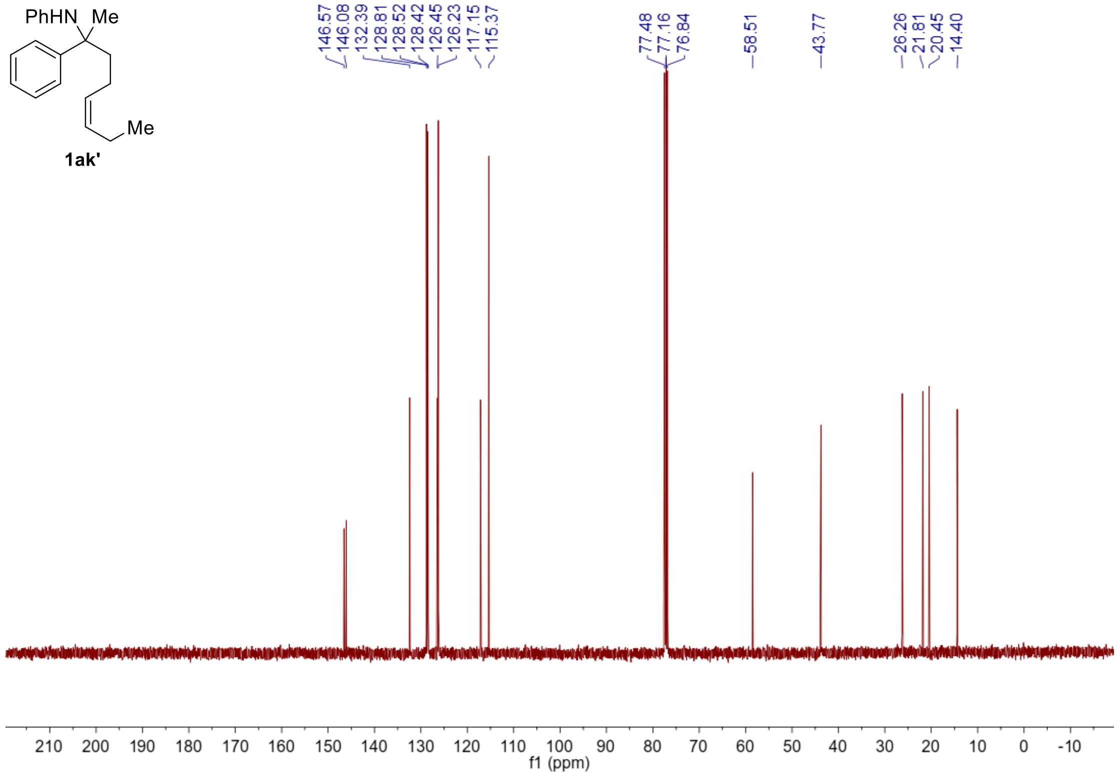
PhHN Me
Me
1ak'
146.57
146.08
132.39
128.81
128.52
128.42
126.45
126.23
117.15
115.37
77.48
77.16
76.84
58.51
43.77
26.26
21.81
20.45
14.40
f1 (ppm)

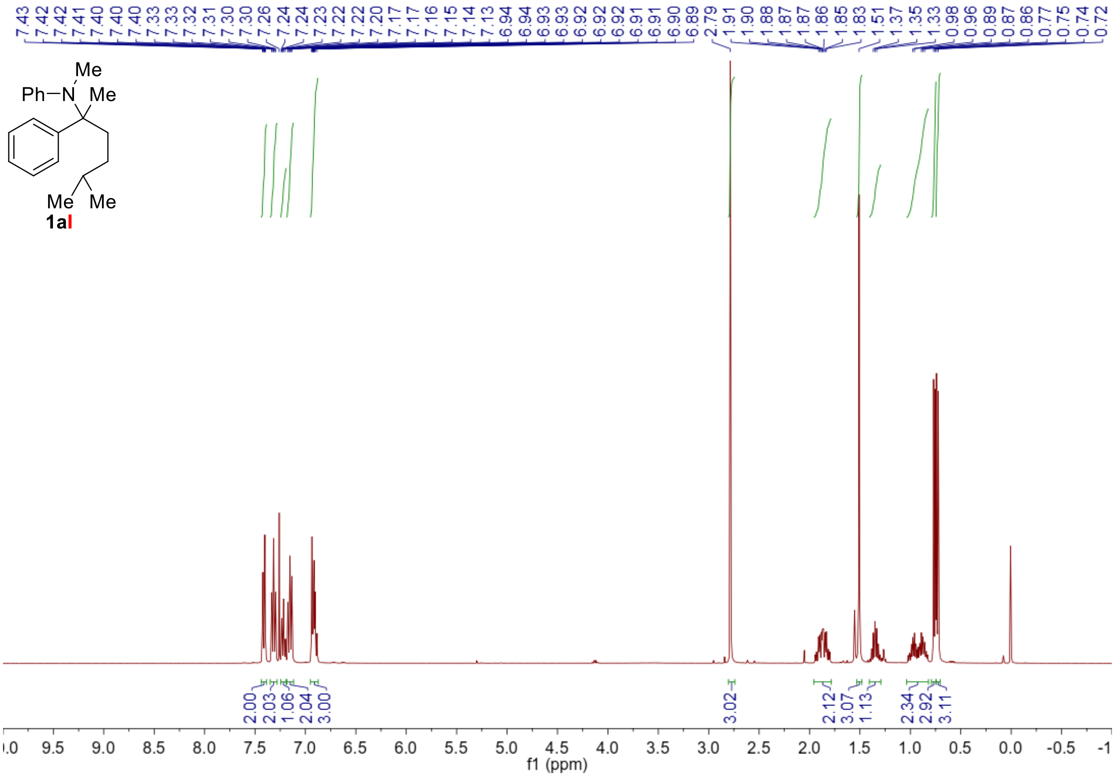
7.43
7.42
7.42
7.41
7.40
7.40
7.40
7.33
7.33
7.32
7.31
7.30
7.30
7.26
7.24
7.24
7.23
7.22
7.22
7.20
7.17
7.17
7.16
7.15
7.14
7.13
6.94
6.93
6.93
6.92
6.92
6.91
6.91
6.90
6.89
2.79
1.91
1.90
1.88
1.87
1.87
1.86
1.85
1.83
1.51
1.37
1.35
1.33
0.98
0.96
0.89
0.87
0.86
0.77
0.75
0.74
0.72
Me
Ph–N Me
Me Me
1al
2.00
2.03
1.06
2.04
3.00
3.02
2.12
3.07
1.13
2.34
2.92
3.11
f1 (ppm)

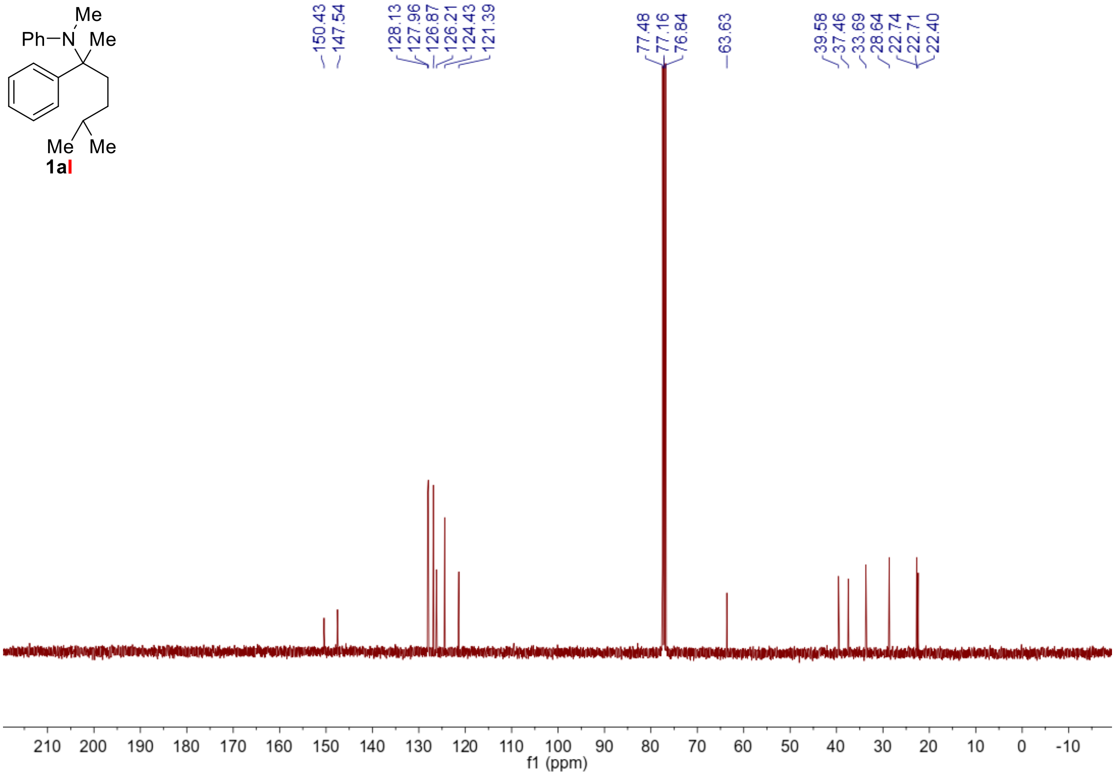
150.43
147.54
128.13
127.96
126.87
126.21
124.43
121.39
77.48
77.16
76.84
63.63
39.58
37.46
33.69
28.64
22.74
22.71
22.40
Me
Ph–N Me
Me Me
1al
f1 (ppm)

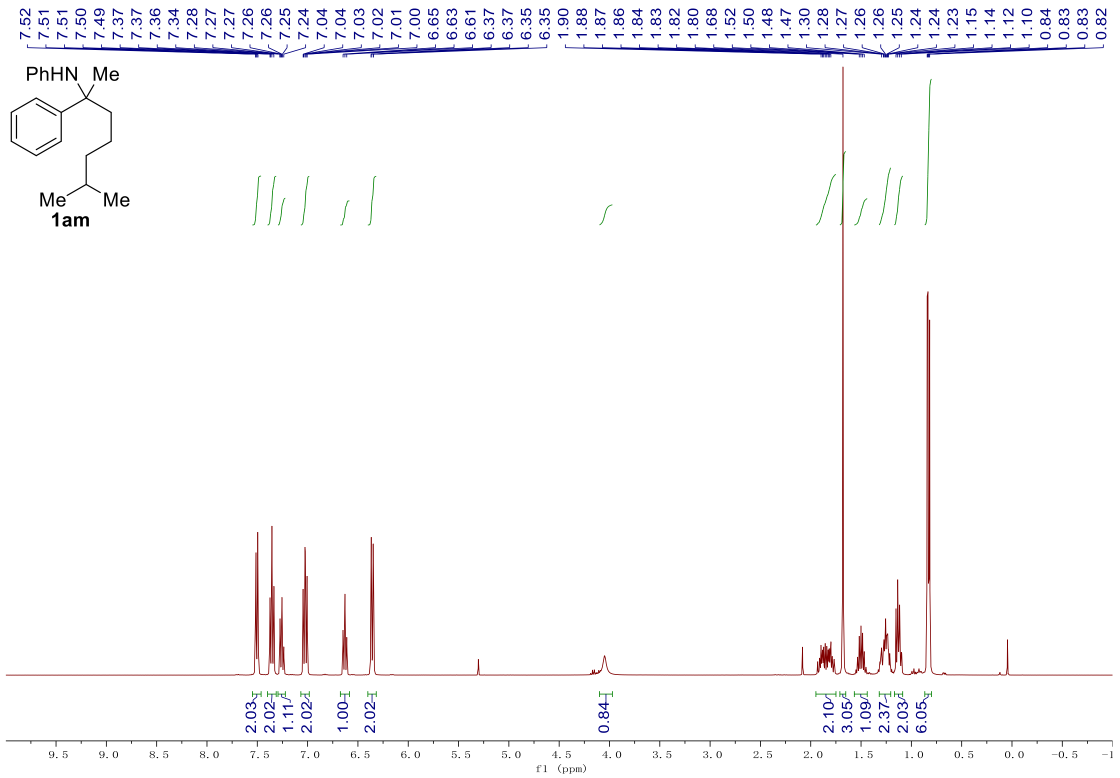
7.52 7.51 7.51 7.50 7.49 7.37 7.37 7.36 7.34 7.28 7.27 7.27 7.26 7.26 7.25 7.24 7.04 7.04 7.03 7.02 7.01 7.00 6.65 6.63 6.61 6.37 6.37 6.35 6.35 1.90 1.88 1.87 1.86 1.84 1.83 1.82 1.80 1.68 1.52 1.50 1.48 1.47 1.30 1.28 1.27 1.26 1.26 1.25 1.24 1.24 1.23 1.15 1.14 1.12 1.10 0.84 0.83 0.83 0.82
PhHN Me
Me Me
1am
2.03 2.02 1.11 2.02 1.00 2.02 0.84 2.10 3.05 1.09 2.37 2.03 6.05
f1 (ppm)

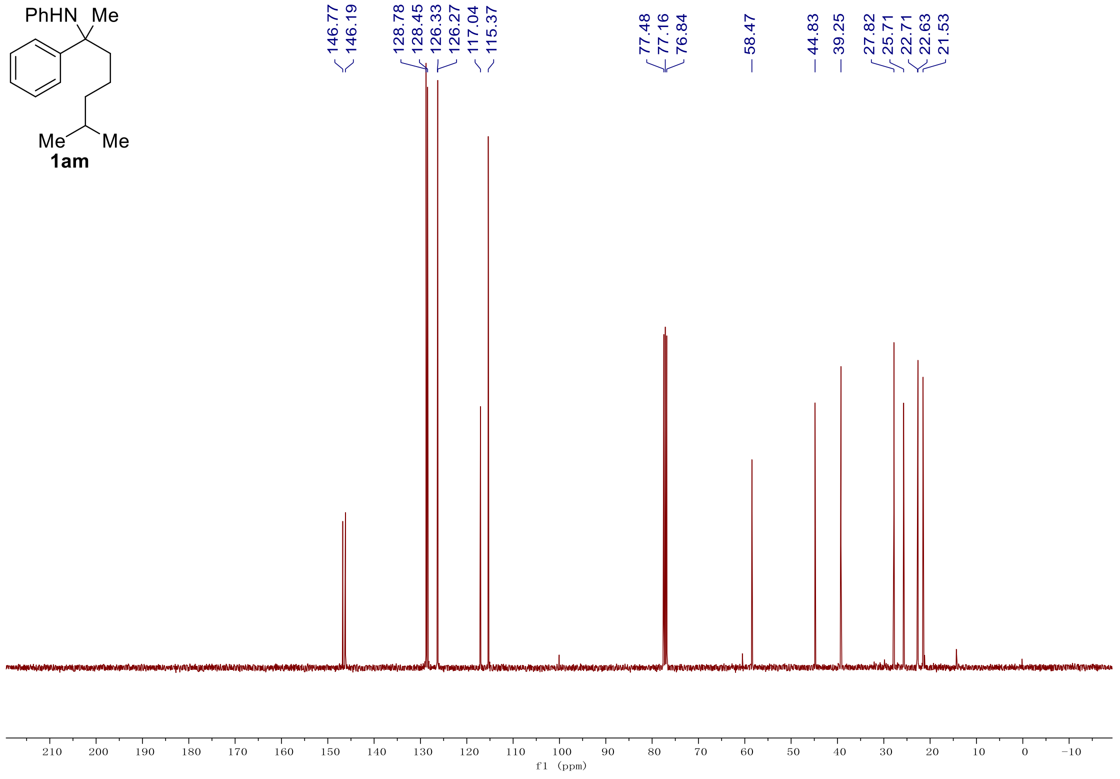
146.77 146.19 128.78 128.45 126.33 126.27 117.04 115.37 77.48 77.16 76.84 58.47 44.83 39.25 27.82 25.71 22.71 22.63 21.53
PhHN Me
Me Me
1am
f1 (ppm)

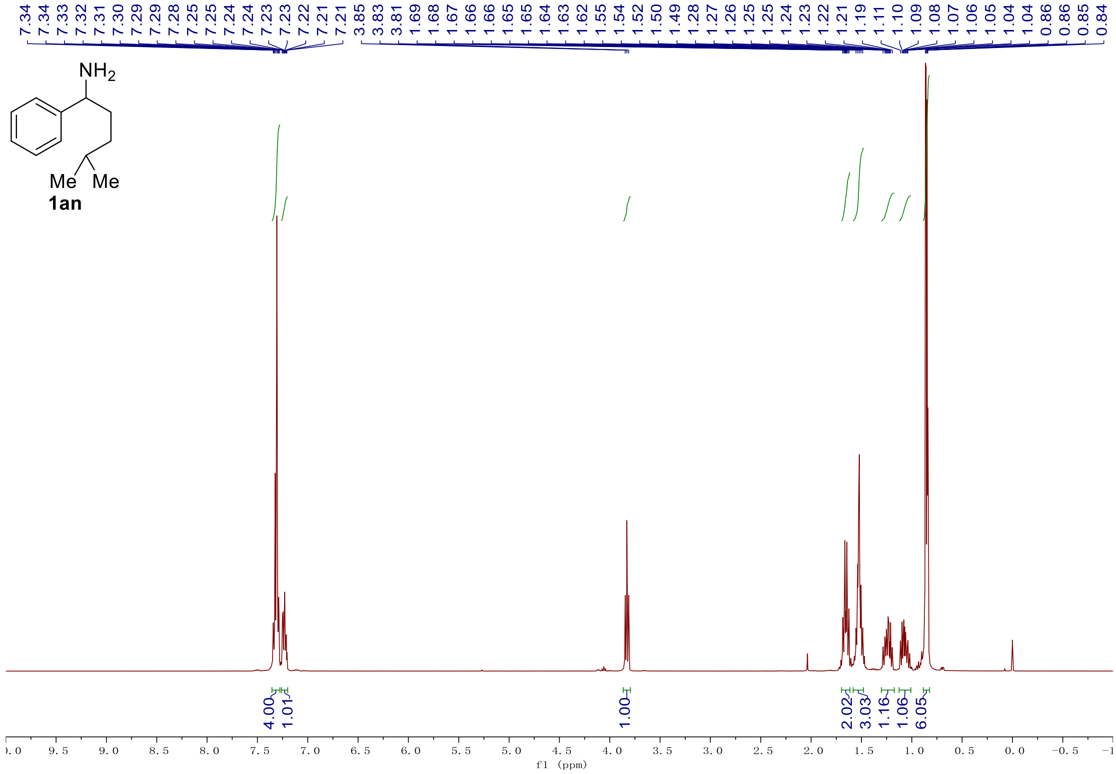
7.34 7.34 7.33 7.32 7.31 7.30 7.29 7.29 7.28 7.25 7.25 7.24 7.24 7.23 7.23 7.22 7.21 7.21
3.85 3.83 3.81 1.69 1.68 1.67 1.66 1.66 1.65 1.65 1.64 1.63 1.62 1.55 1.54 1.52 1.50 1.49 1.28 1.27 1.26 1.25 1.25 1.24 1.23 1.22 1.21 1.19 1.11 1.10 1.09 1.08 1.07 1.06 1.05 1.04 1.04 0.86 0.86 0.85 0.84
NH2
Me Me
1an
4.00 1.01 1.00 2.02 3.03 1.16 1.06 6.05
f1 (ppm)

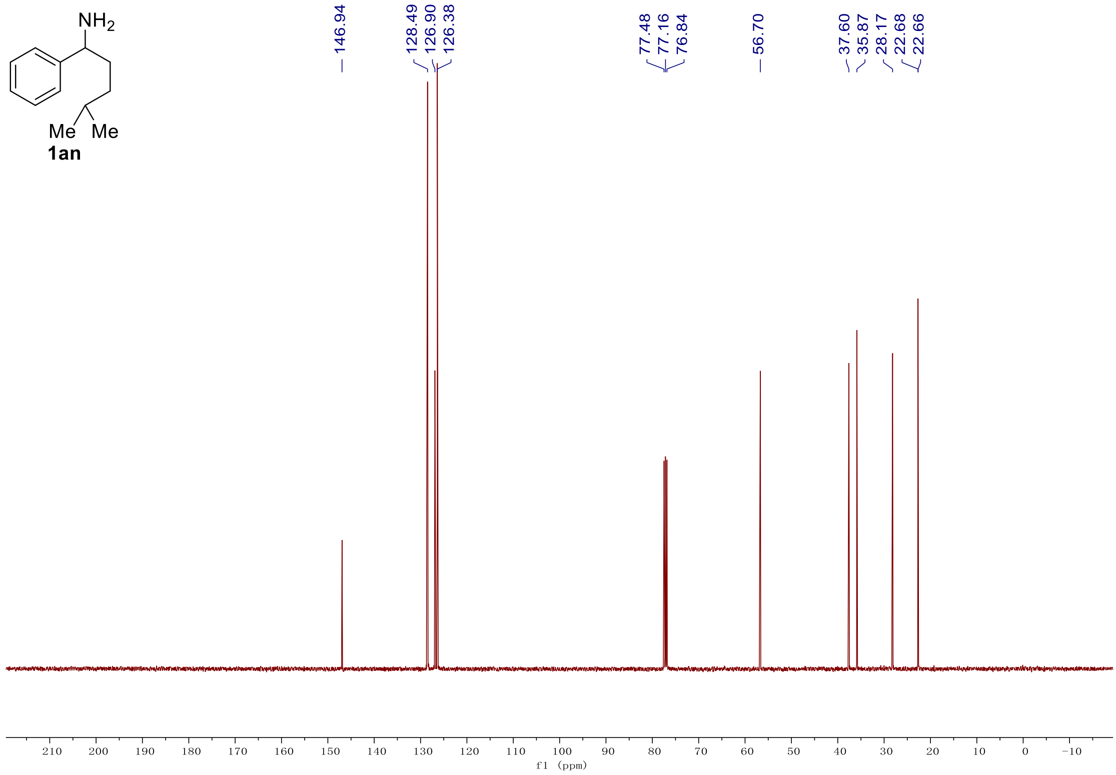
146.94
128.49 126.90 126.38
77.48 77.16 76.84
56.70
37.60 35.87 28.17 22.68 22.66
NH2
Me Me
1an
f1 (ppm)

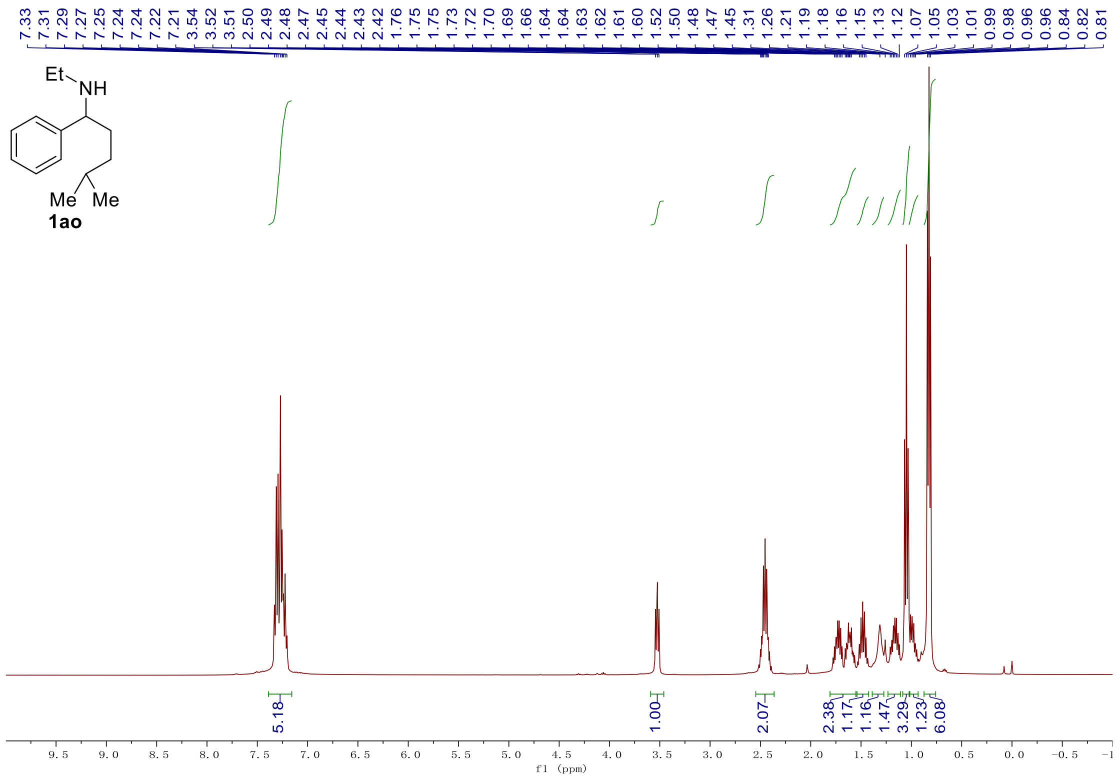
Et
NH
Me
Me
1ao
f1 (ppm)

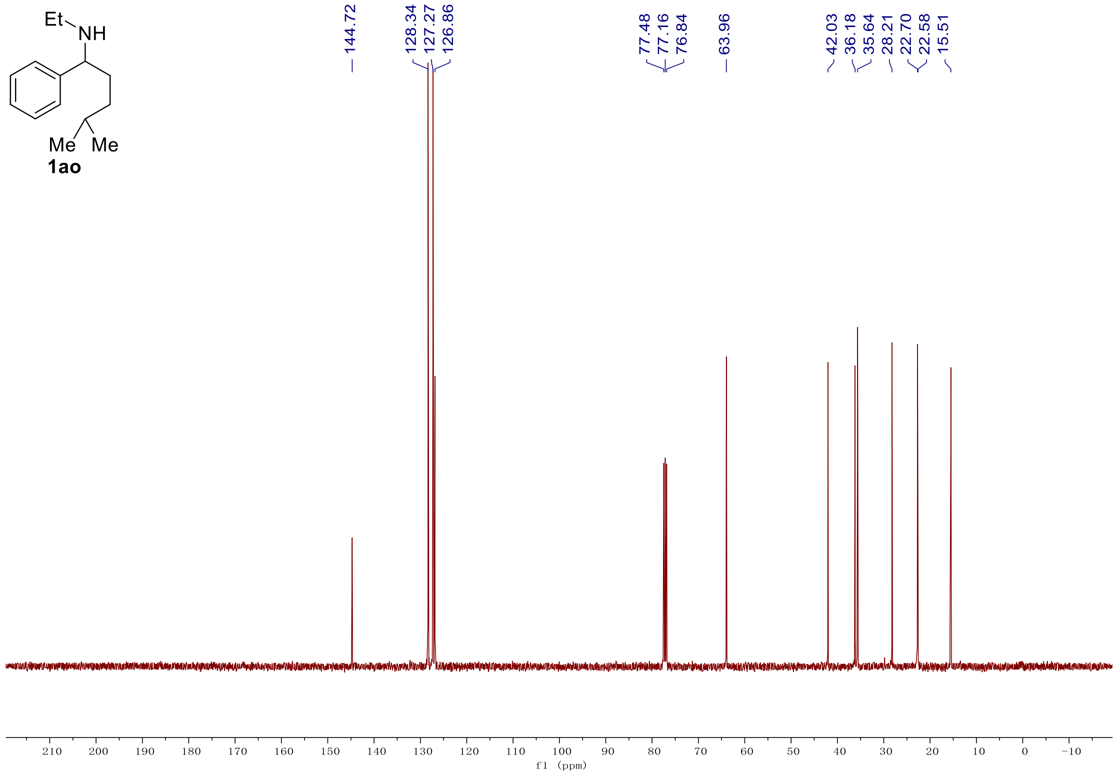
Et
NH
Me
Me
1ao
f1 (ppm)

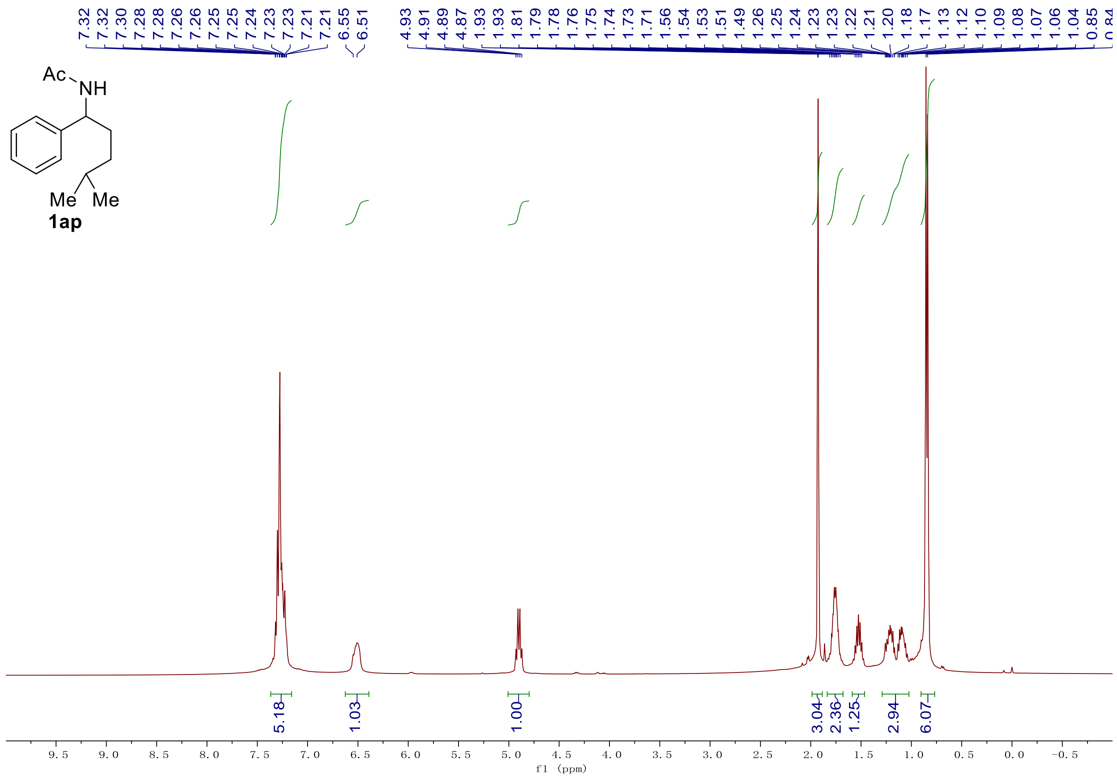

7.32
7.32
7.30
7.28
7.28
7.26
7.26
7.25
7.25
7.24
7.23
7.23
7.21
7.21
6.55
6.51
4.93
4.91
4.89
4.87
1.93
1.93
1.81
1.79
1.78
1.76
1.75
1.74
1.73
1.71
1.56
1.54
1.53
1.51
1.49
1.26
1.25
1.24
1.23
1.23
1.22
1.21
1.20
1.18
1.17
1.13
1.12
1.10
1.09
1.08
1.07
1.06
1.04
0.85
Ac
NH
Me
Me
1ap
5.18
1.03
1.00
3.04
2.36
1.25
2.94
6.07
f1 (ppm)

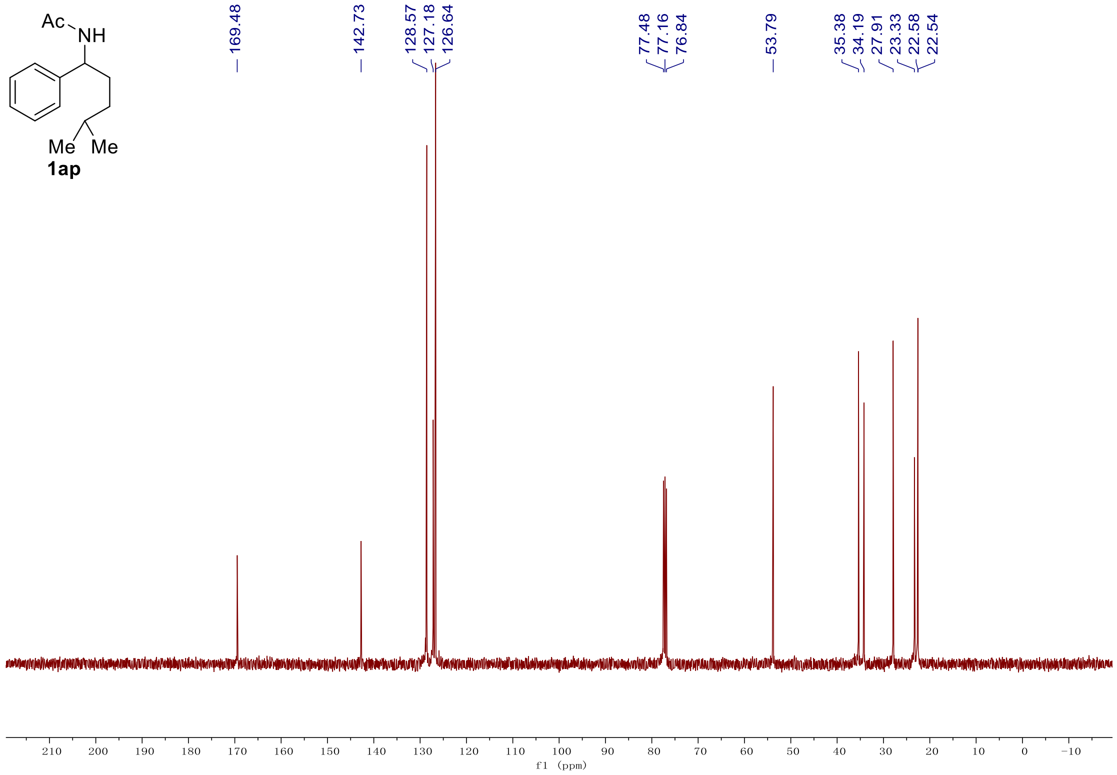

Ac
NH
Me
Me
1ap
169.48
142.73
128.57
127.18
126.64
77.48
77.16
76.84
53.79
35.38
34.19
27.91
23.33
22.58
22.54
f1 (ppm)

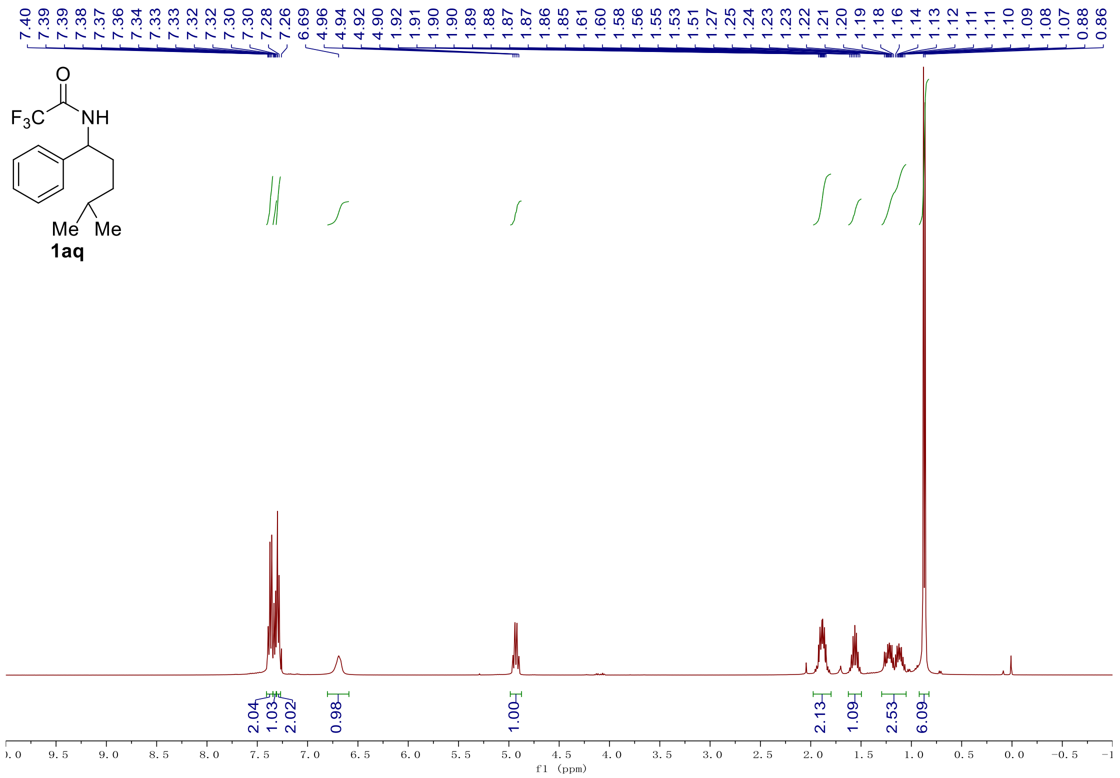
7.40 7.39 7.39 7.38 7.37 7.36 7.34 7.33 7.33 7.32 7.30 7.30 7.28 7.26 6.69 4.96 4.94 4.92 4.90 1.92 1.91 1.90 1.90 1.89 1.88 1.87 1.86 1.85 1.61 1.60 1.58 1.56 1.55 1.53 1.51 1.27 1.25 1.24 1.23 1.23 1.22 1.21 1.20 1.19 1.18 1.16 1.14 1.13 1.12 1.11 1.10 1.09 1.08 1.07 0.88 0.86
O
F_3C
NH
Me
Me
1aq
2.04
1.03
2.02
0.98
1.00
2.13
1.09
2.53
6.09
f1 (ppm)

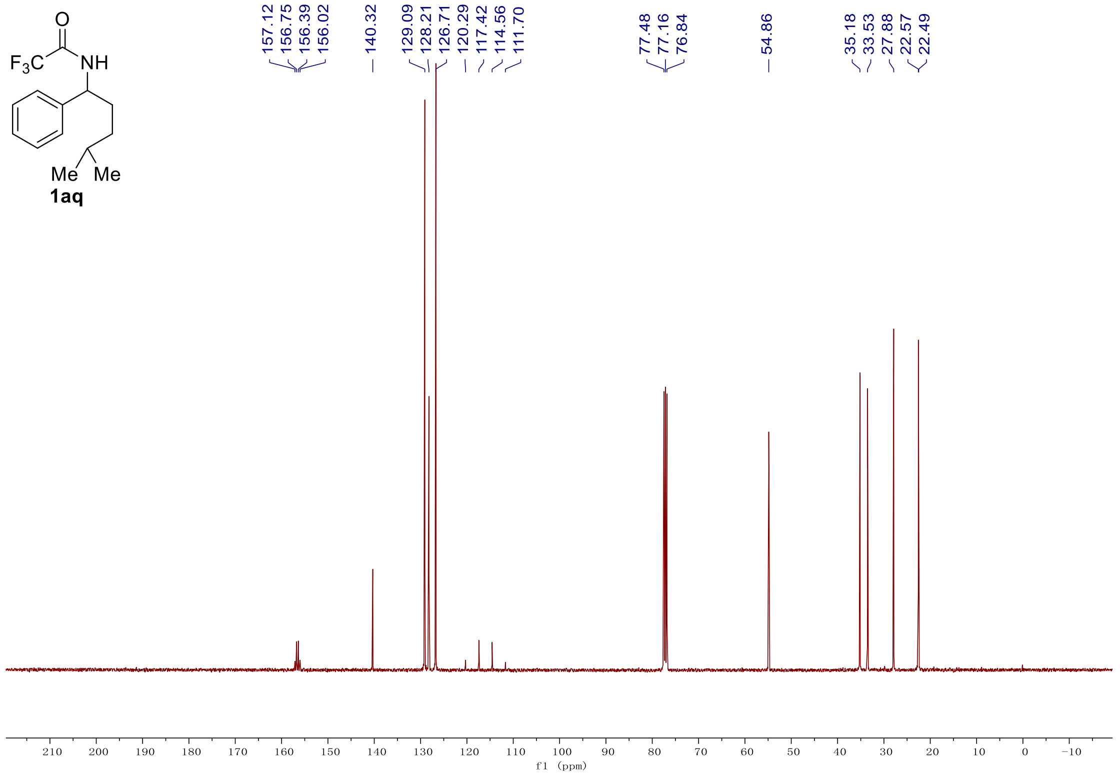
157.12 156.75 156.39 156.02
140.32
129.09 128.21 126.71 120.29 117.42 114.56 111.70
77.48 77.16 76.84
54.86
35.18 33.53 27.88 22.57 22.49
O
F_3C
NH
Me
Me
1aq
f1 (ppm)

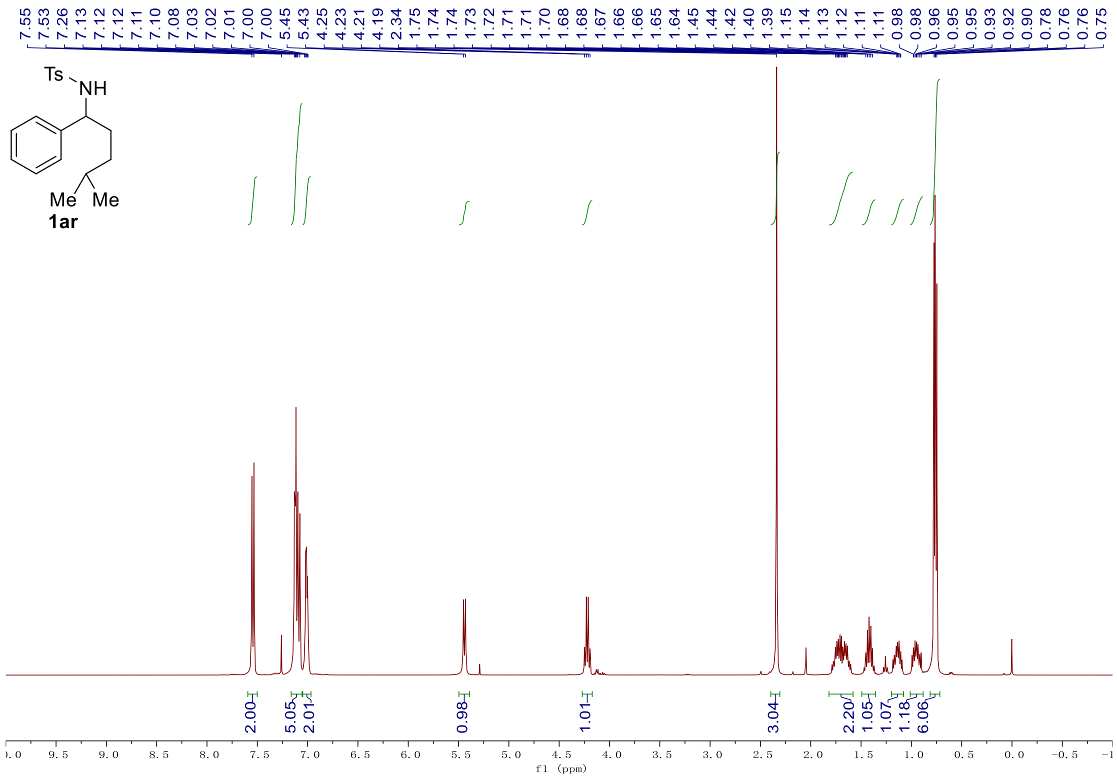
7.55 7.53 7.26 7.13 7.12 7.11 7.10 7.08 7.03 7.02 7.01 7.00 7.00 5.45 5.43 4.25 4.23 4.21 4.19 2.34 1.75 1.74 1.74 1.73 1.72 1.71 1.71 1.70 1.68 1.68 1.67 1.66 1.65 1.64 1.45 1.44 1.42 1.40 1.39 1.15 1.14 1.13 1.12 1.11 1.11 0.98 0.98 0.96 0.95 0.95 0.93 0.92 0.90 0.78 0.76 0.76 0.75
Ts
NH
Me
Me
1ar
2.00
5.05
2.01
0.98
1.01
3.04
2.20
1.05
1.07
1.18
6.06
f1 (ppm)

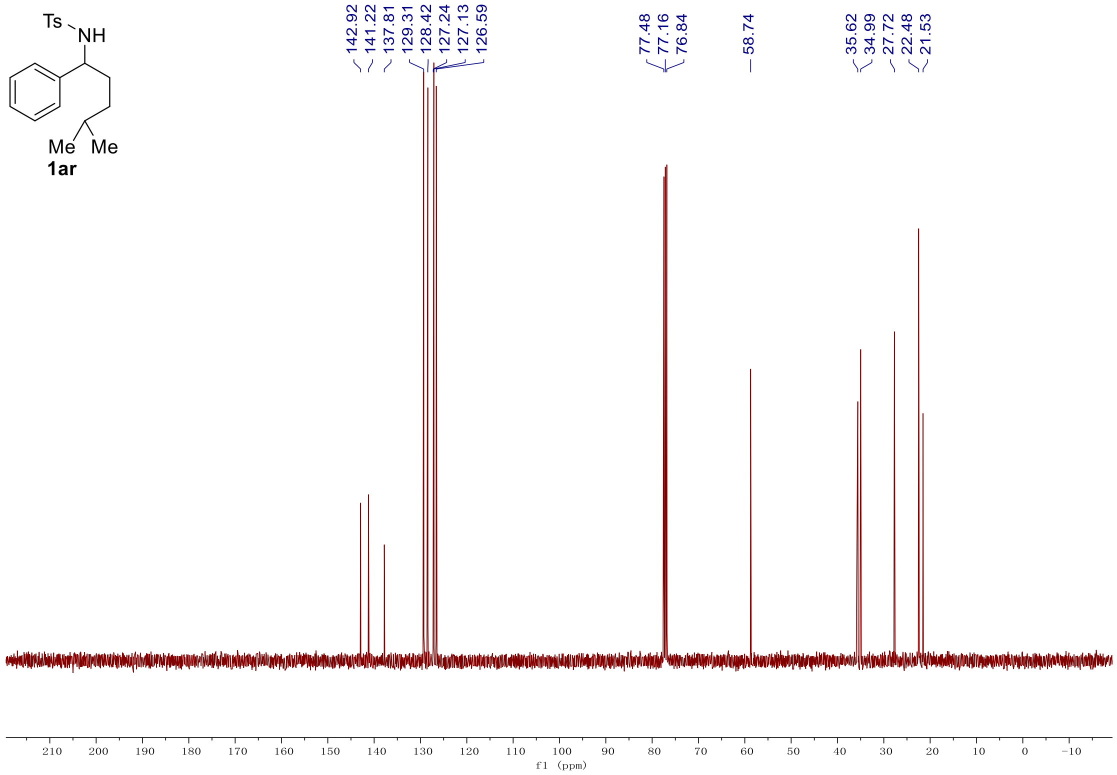
142.92 141.22 137.81 129.31 128.42 127.24 127.13 126.59
77.48 77.16 76.84
58.74
35.62 34.99 27.72 22.48 21.53
Ts
NH
Me
Me
1ar
f1 (ppm)

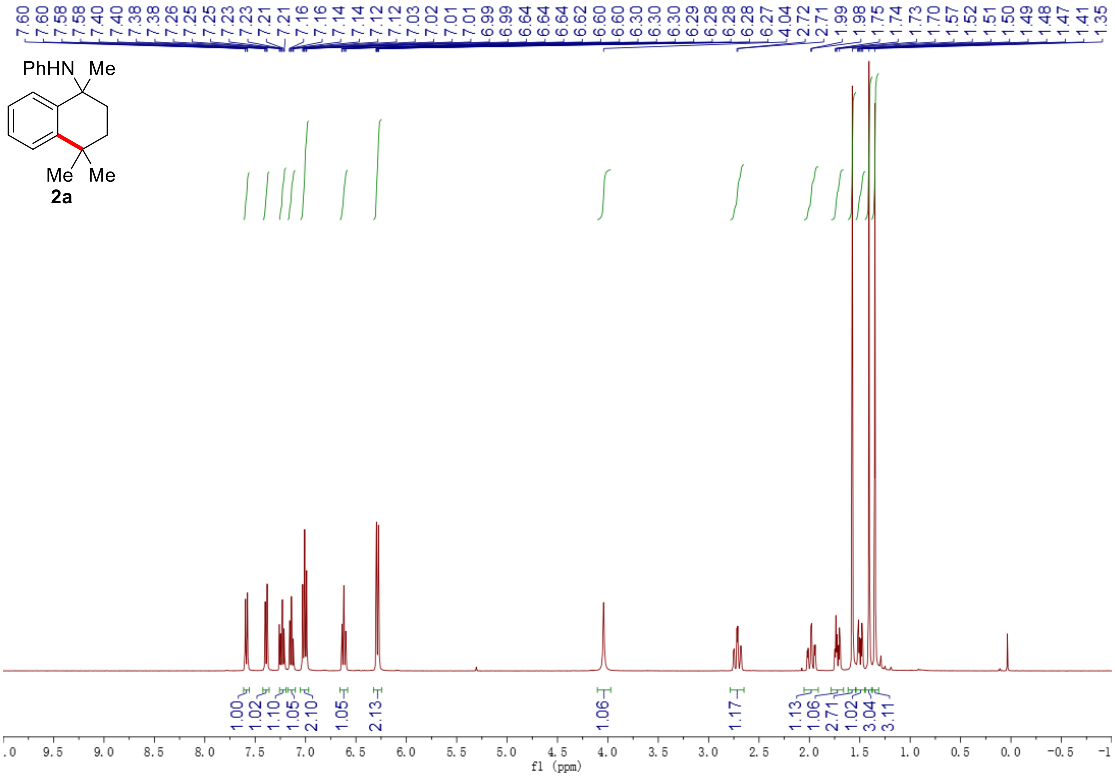
7.60 7.60 7.58 7.58 7.40 7.40 7.38 7.38 7.26 7.25 7.25 7.23 7.23 7.21 7.21 7.16 7.16 7.14 7.14 7.12 7.12 7.03 7.02 7.01 7.01 6.99 6.99 6.64 6.64 6.62 6.62 6.60 6.60 6.30 6.30 6.30 6.29 6.28 6.28 6.28 6.27 4.04 2.72 2.71 1.99 1.98 1.75 1.74 1.73 1.70 1.57 1.52 1.51 1.50 1.49 1.48 1.47 1.41 1.35
PhHN Me
Me Me
2a
1.00 1.02 1.10 1.05 2.10 1.05 2.13 1.06 1.17 1.13 1.06 2.71 1.02 3.04 3.11
f1 (ppm)

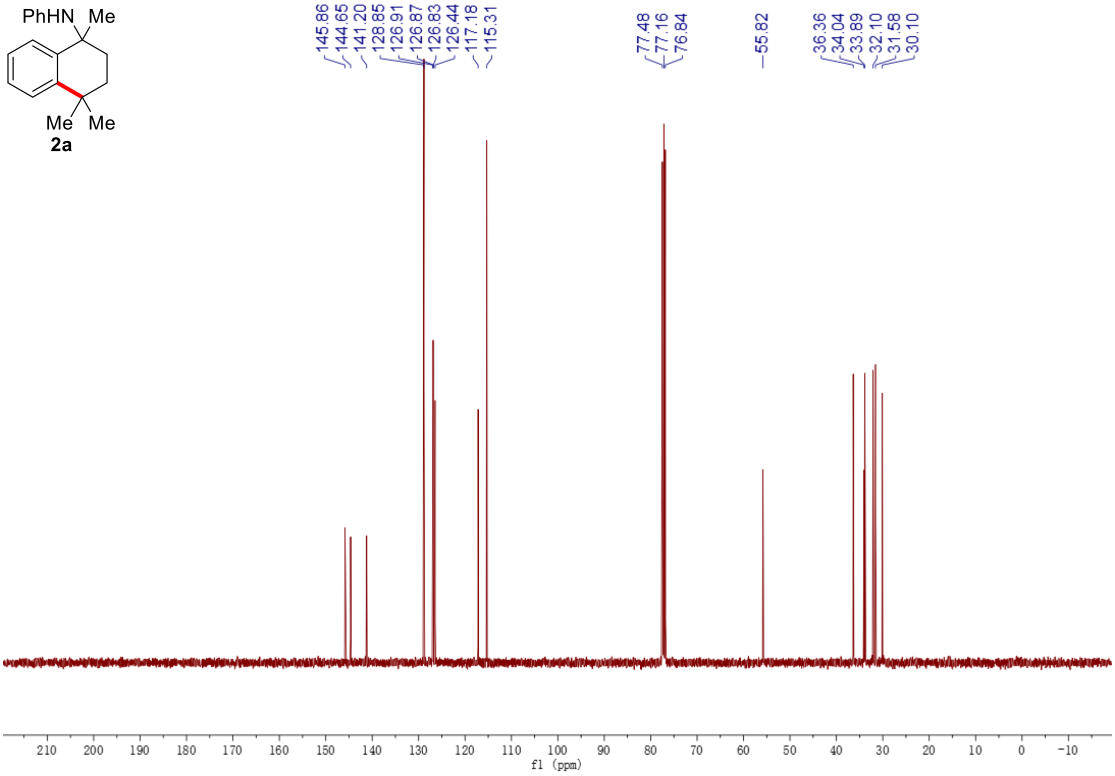
145.86 144.65 141.20 128.85 126.91 126.87 126.83 126.44 117.18 115.31
77.48 77.16 76.84
55.82
36.36 34.04 33.89 32.10 31.58 30.10
PhHN Me
Me Me
2a
f1 (ppm)

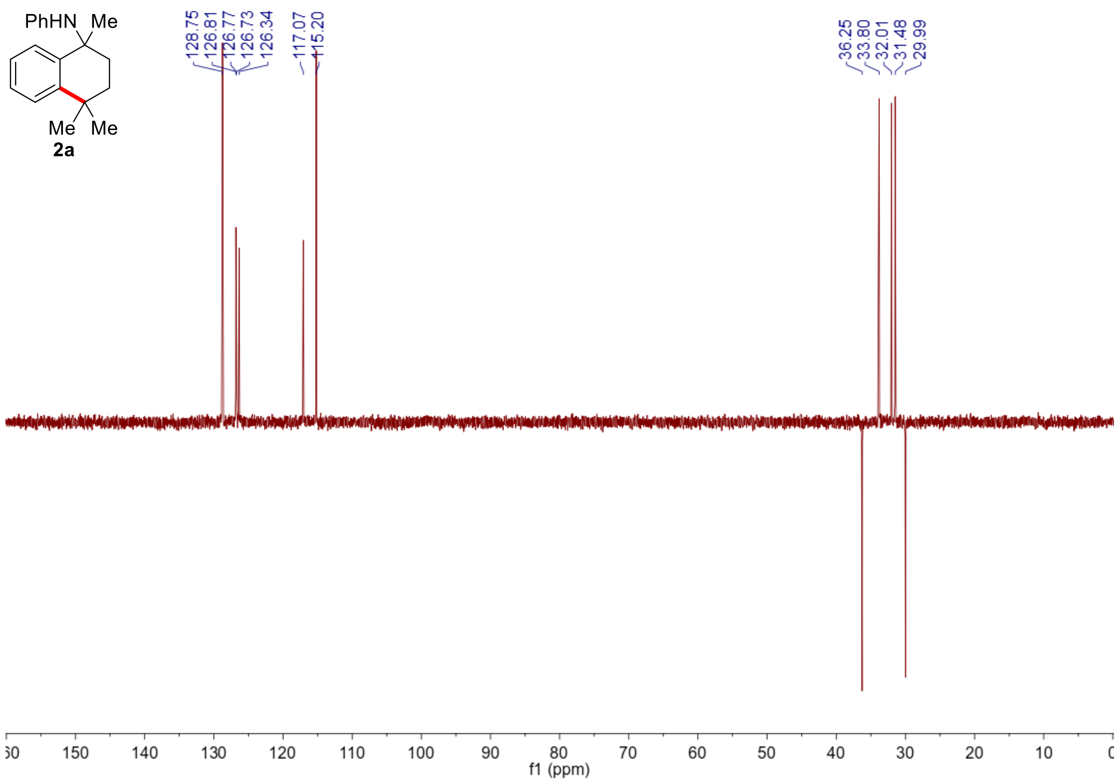
PhHN Me
Me Me
2a
128.75
126.81
126.77
126.73
126.34
117.07
115.20
36.25
33.80
32.01
31.48
29.99
f1 (ppm)

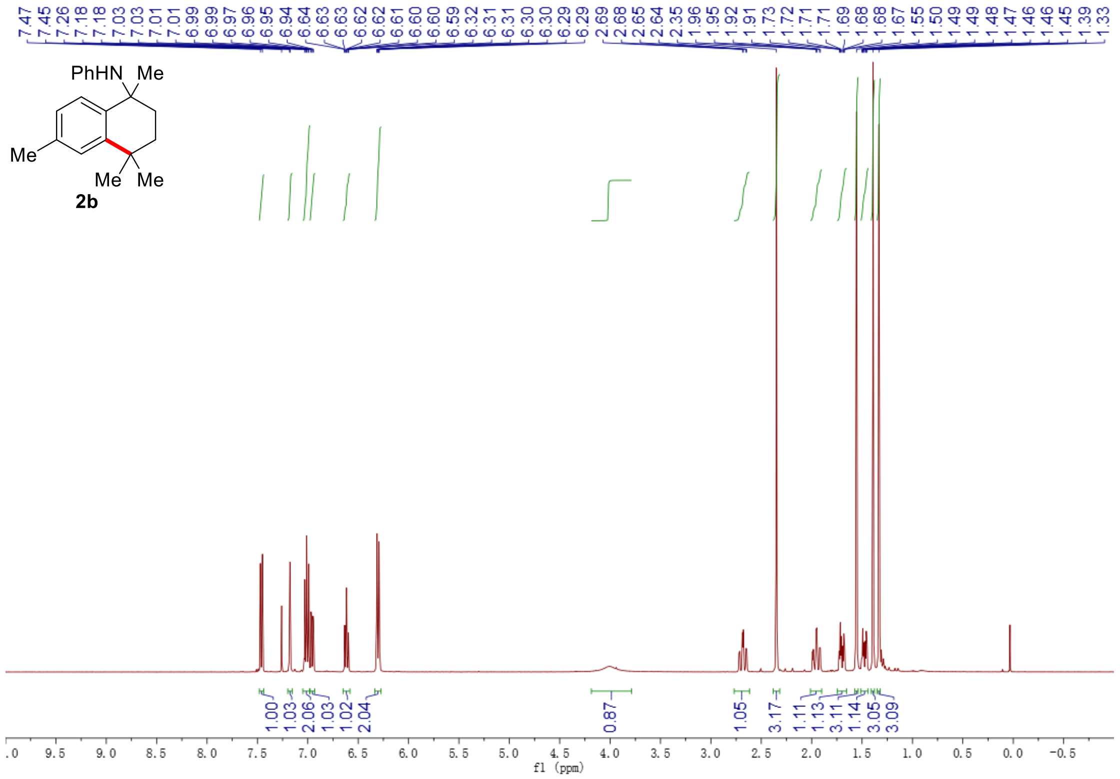
7.47
7.45
7.26
7.18
7.18
7.03
7.01
7.01
6.99
6.99
6.97
6.96
6.95
6.94
6.64
6.63
6.63
6.62
6.62
6.61
6.60
6.60
6.59
6.32
6.31
6.31
6.30
6.29
6.29
2.69
2.68
2.65
2.64
2.35
1.96
1.95
1.92
1.91
1.73
1.72
1.71
1.71
1.69
1.68
1.68
1.67
1.55
1.50
1.49
1.48
1.47
1.46
1.45
1.39
1.33
PhHN Me
Me
Me Me
2b
1.00
1.03
2.06
1.03
1.02
2.04
0.87
1.05
3.17
1.11
1.13
3.11
1.14
3.05
3.09
f1 (ppm)

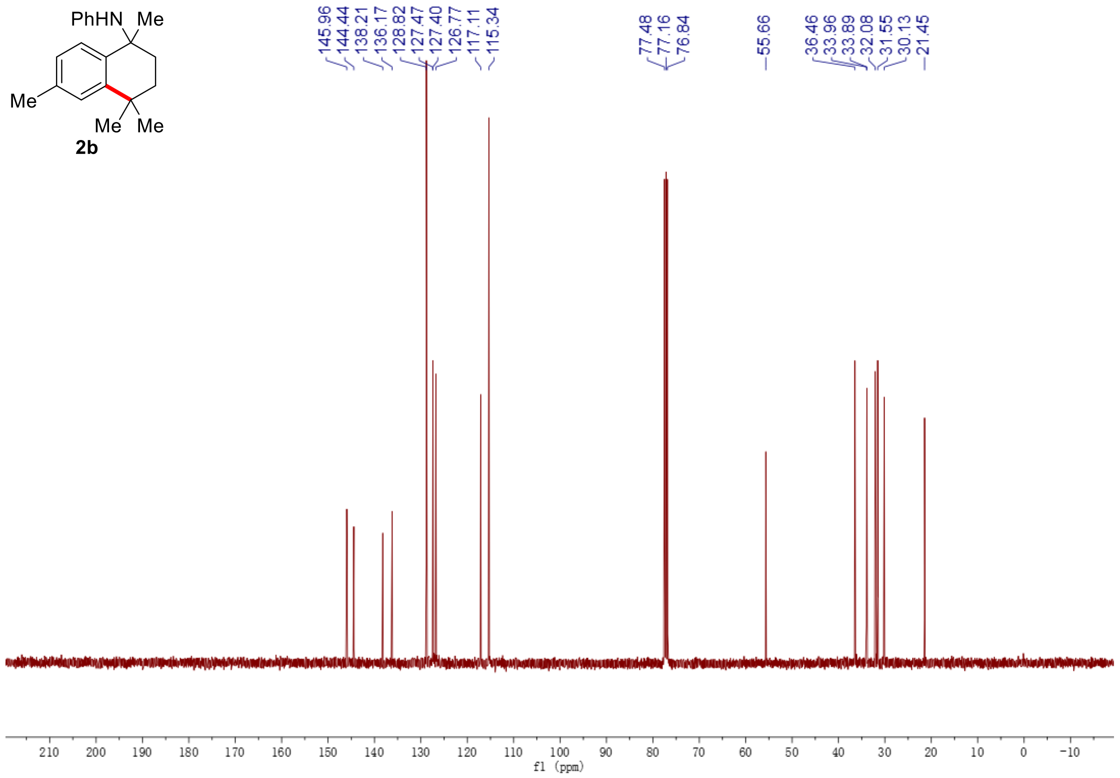
PhHN Me
Me
Me Me
145.96
144.44
138.21
136.17
128.82
127.47
127.40
126.77
117.11
115.34
77.48
77.16
76.84
55.66
36.46
33.96
33.89
32.08
31.55
30.13
21.45
210 200 190 180 170 160 150 140 130 120 110 100 90 80 70 60 50 40 30 20 10 0 -10
f1 (ppm)

2b

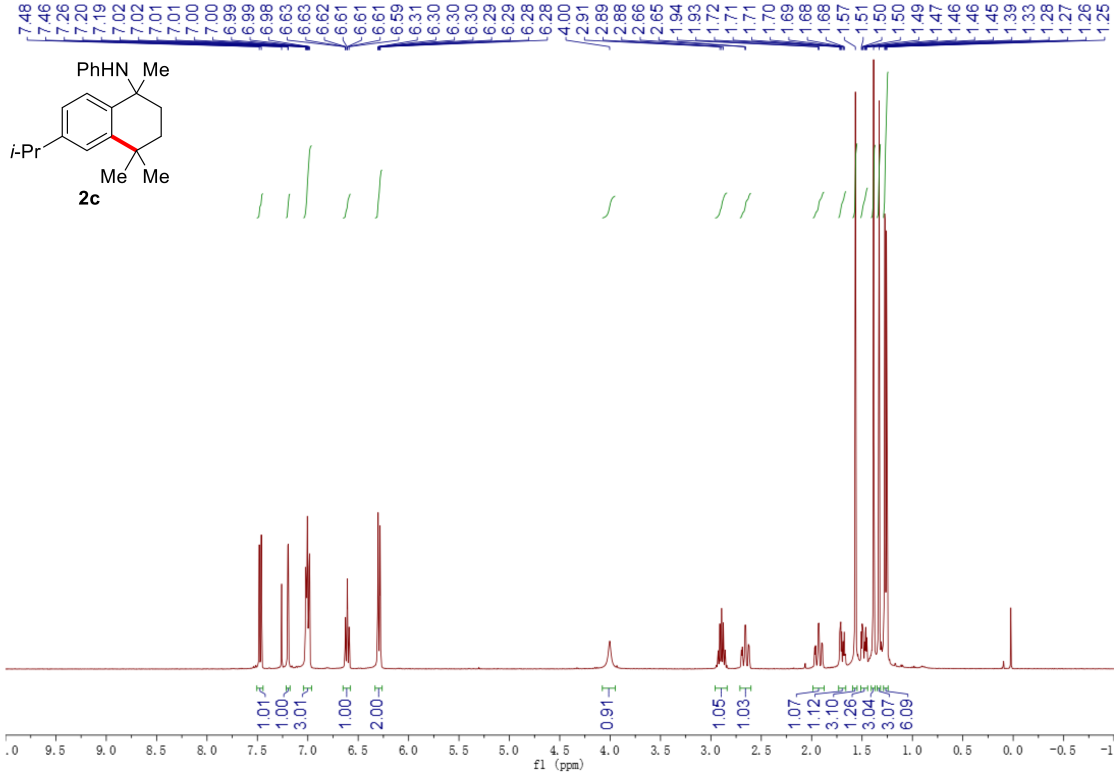
PhHN Me
i-Pr
Me Me
7.48
7.46
7.26
7.20
7.19
7.02
7.01
7.01
7.00
6.99
6.99
6.98
6.63
6.62
6.61
6.61
6.59
6.31
6.30
6.30
6.29
6.29
6.28
4.00
2.91
2.89
2.88
2.66
2.65
1.94
1.93
1.72
1.71
1.71
1.70
1.69
1.68
1.68
1.57
1.51
1.50
1.50
1.49
1.47
1.46
1.46
1.45
1.39
1.33
1.28
1.27
1.26
1.25
1.01
1.00
3.01
1.00
2.00
0.91
1.05
1.03
1.07
1.12
3.10
1.26
3.04
3.07
6.09
.0 9.5 9.0 8.5 8.0 7.5 7.0 6.5 6.0 5.5 5.0 4.5 4.0 3.5 3.0 2.5 2.0 1.5 1.0 0.5 0.0 -0.5 -1
f1 (ppm)

2c

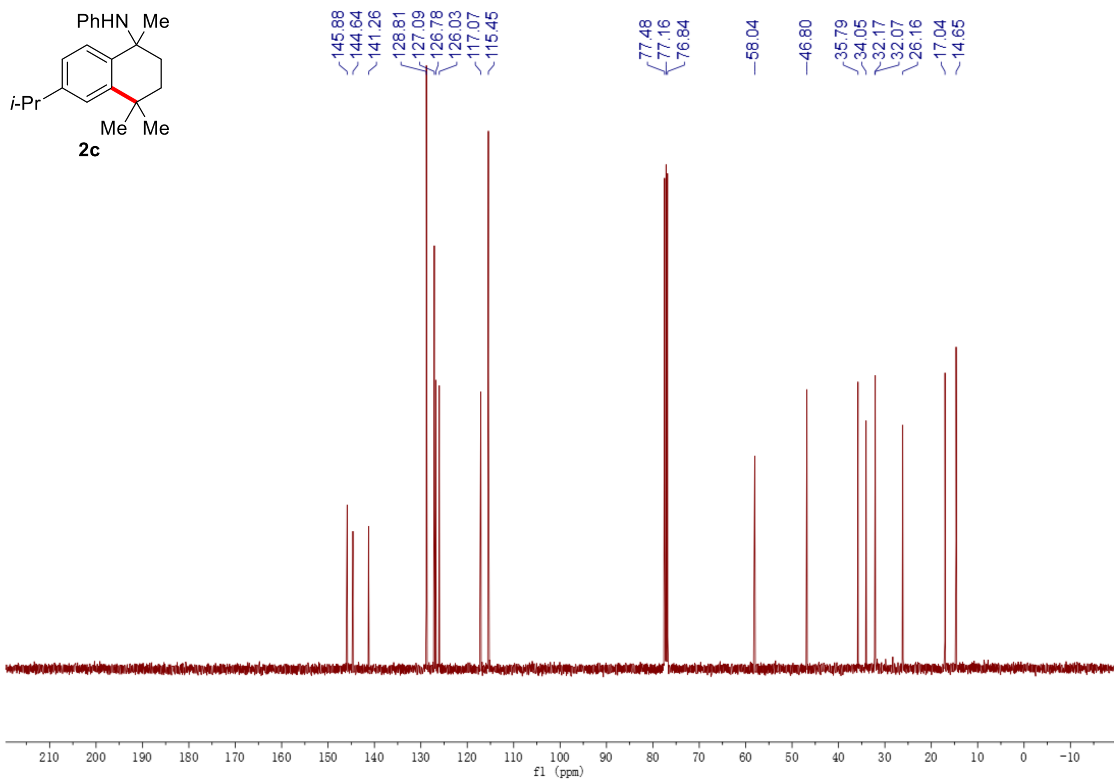
PhHN Me
i-Pr
Me Me
2c
145.88
144.64
141.26
128.81
127.09
126.78
126.03
117.07
115.45
77.48
77.16
76.84
58.04
46.80
35.79
34.05
32.17
32.07
26.16
17.04
14.65
f1 (ppm)

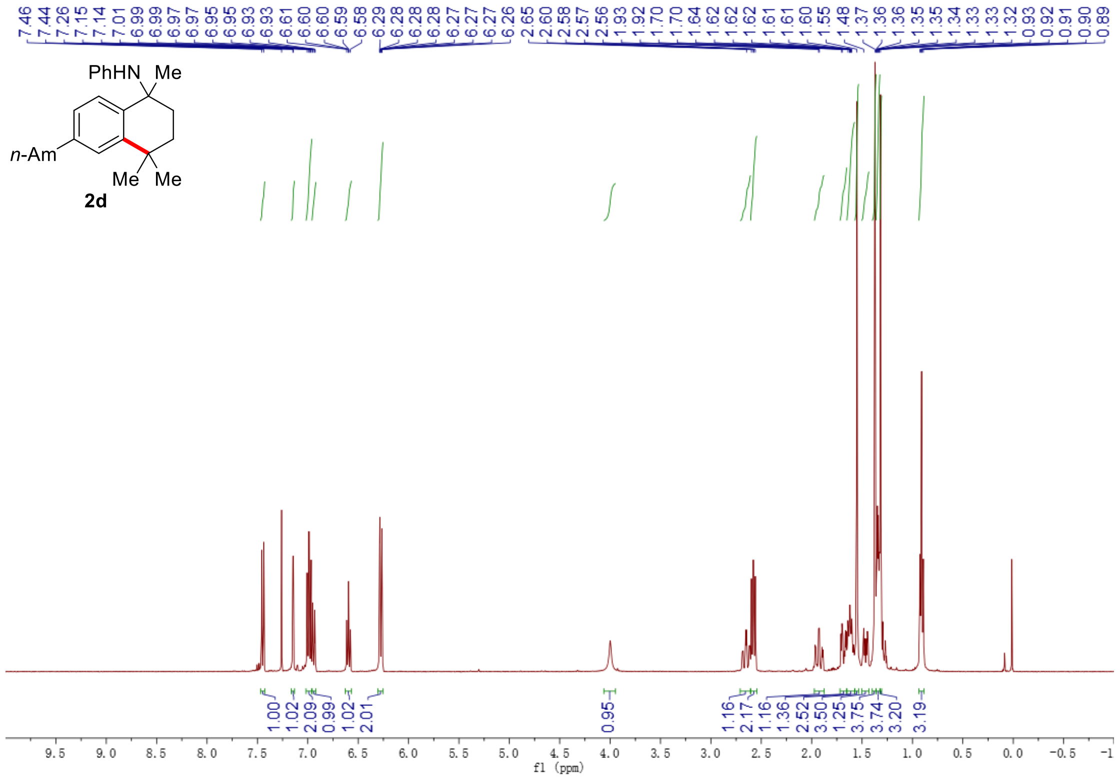
PhHN Me
n-Am
Me Me
2d
f1 (ppm)

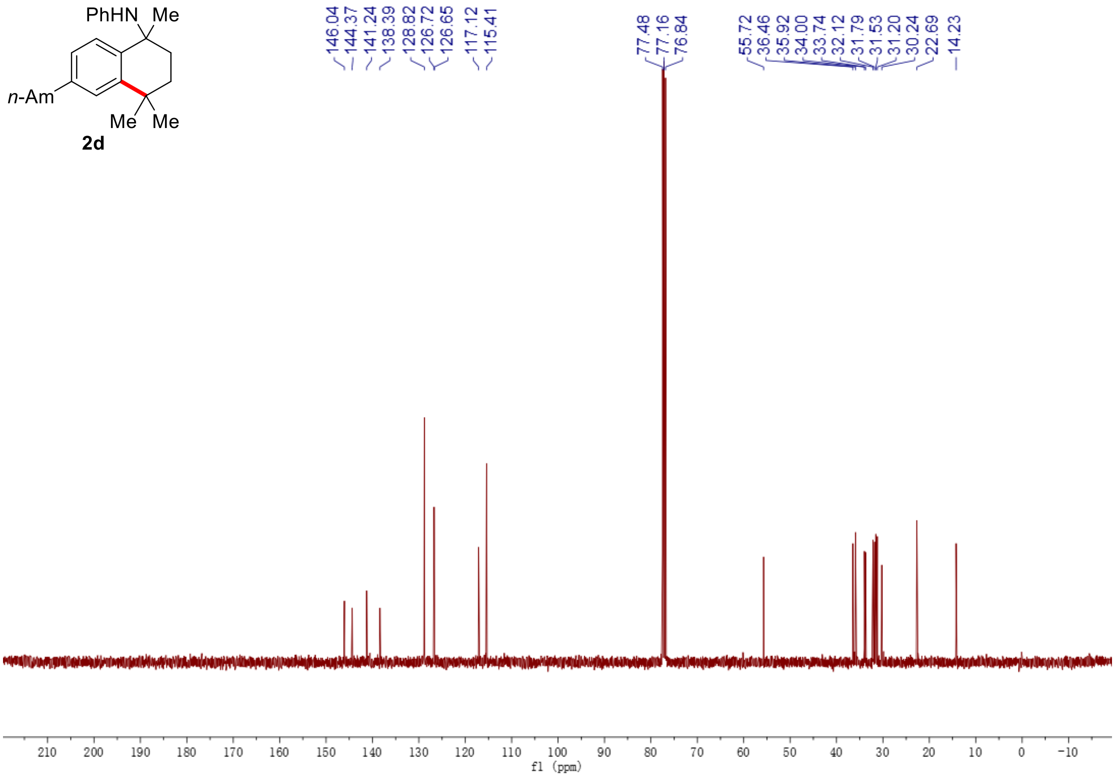

2d

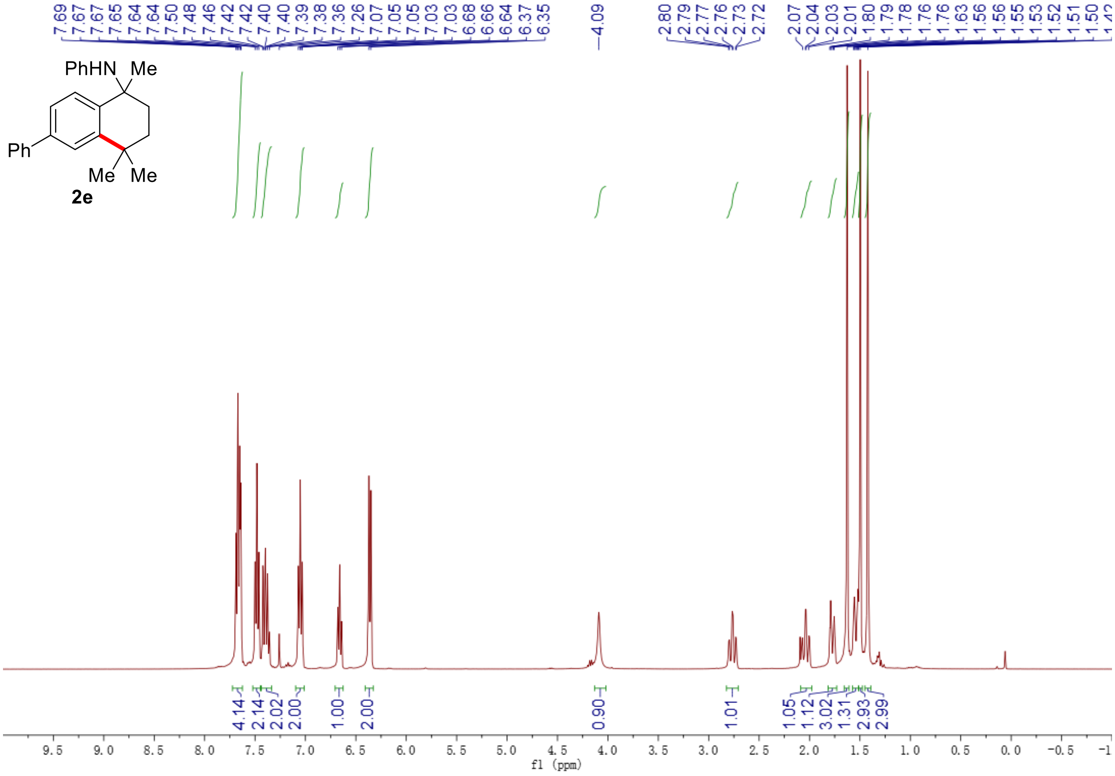

2e

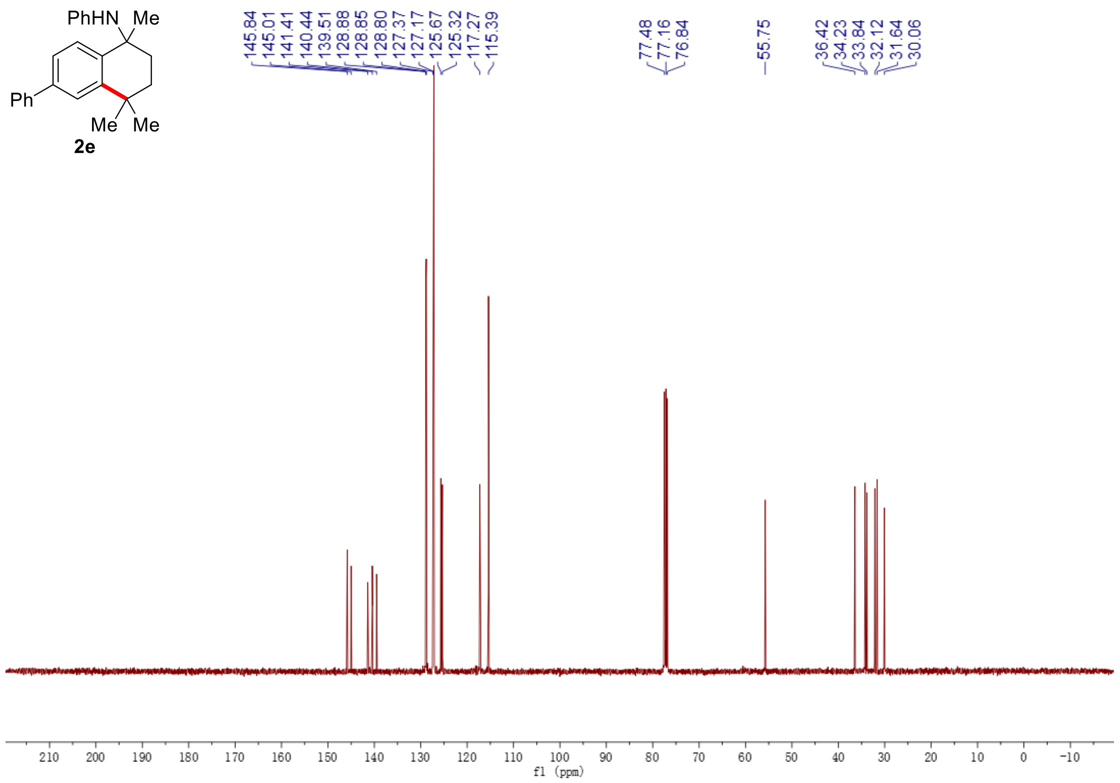
PhHN
Me
Ph
Me
Me
2e
145.84
145.01
141.41
140.44
139.51
128.88
128.85
128.80
127.37
127.17
125.67
125.32
117.27
115.39
77.48
77.16
76.84
55.75
36.42
34.23
33.84
32.12
31.64
30.06
f1 (ppm)

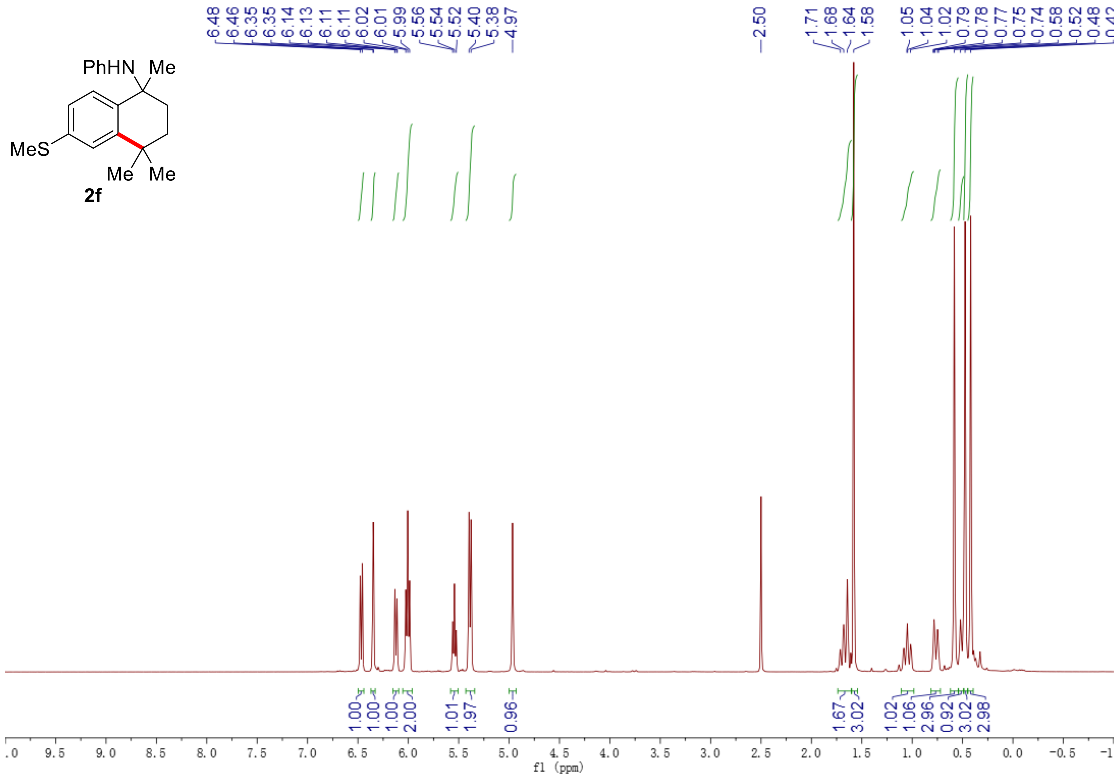
PhHN
Me
MeS
Me
Me
2f
6.48
6.46
6.35
6.35
6.14
6.13
6.11
6.11
6.02
6.01
5.99
5.56
5.54
5.52
5.40
5.38
4.97
2.50
1.71
1.68
1.64
1.58
1.05
1.04
1.02
0.79
0.78
0.77
0.75
0.74
0.58
0.52
0.48
1.00
1.00
1.00
2.00
1.01
1.97
0.96
1.67
3.02
1.02
1.06
2.96
0.92
3.02
2.98
f1 (ppm)

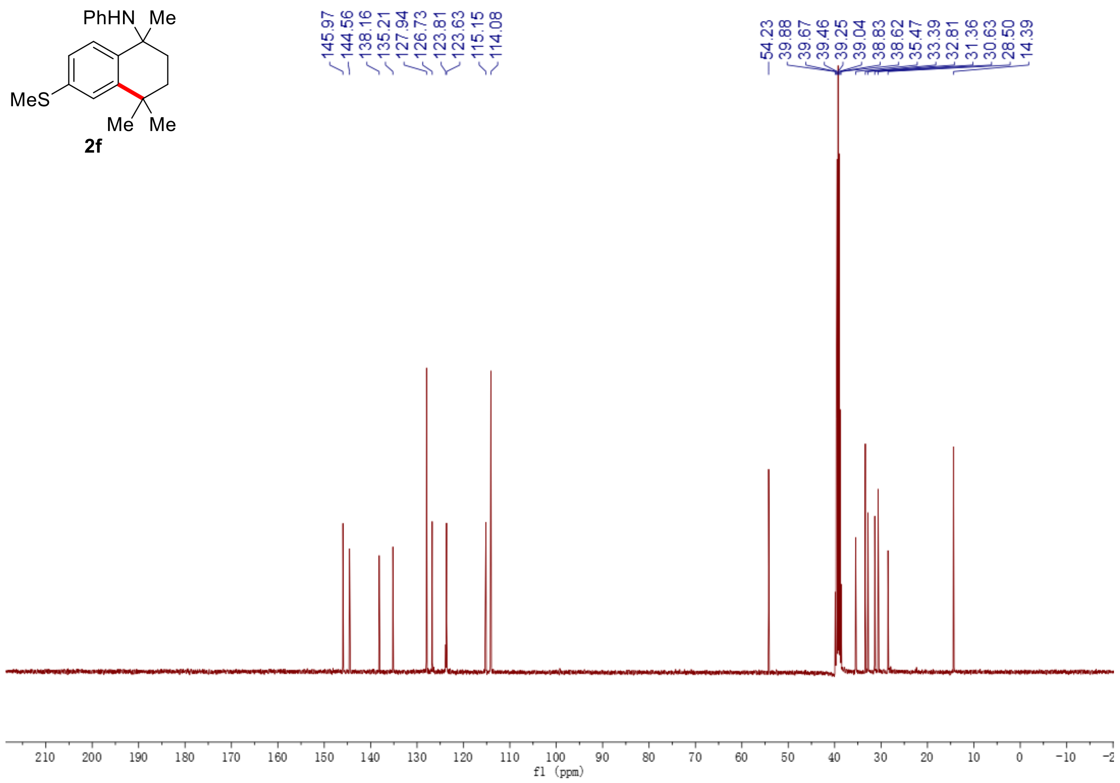
PhHN
Me
MeS
Me
Me
2f
145.97
144.56
138.16
135.21
127.94
126.73
123.81
123.63
115.15
114.08
54.23
39.88
39.67
39.46
39.25
39.04
38.83
38.62
35.47
33.39
32.81
31.36
30.63
28.50
14.39
f1 (ppm)

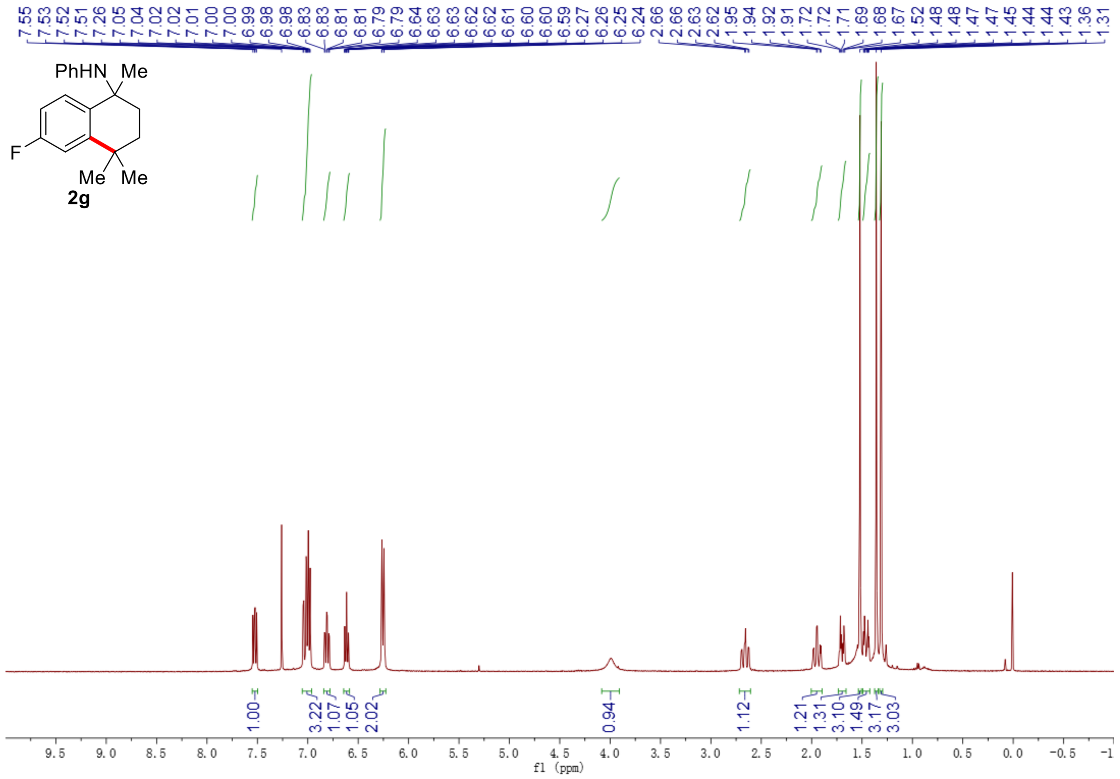
PhHN
Me
F
Me
Me
2g
1.00
3.22
1.07
1.05
2.02
0.94
1.12
1.21
1.31
3.10
1.49
3.17
3.03
f1 (ppm)

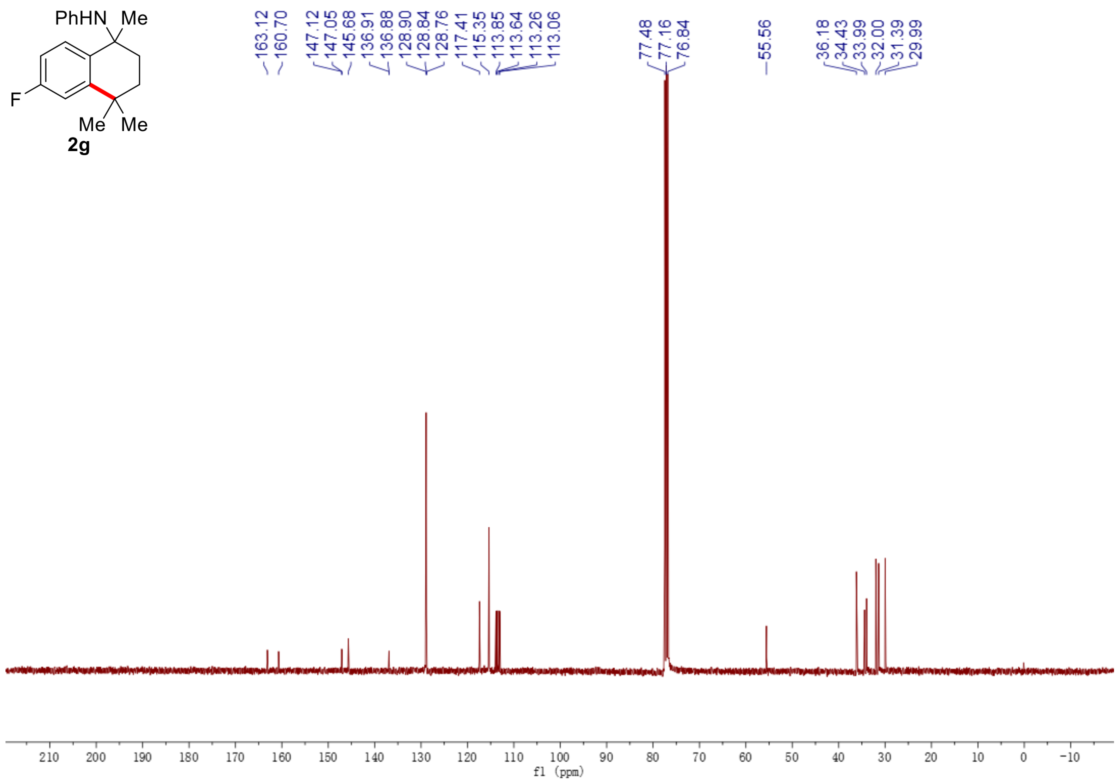
PhHN Me
F
Me Me
2g
163.12
160.70
147.12
147.05
145.68
136.91
136.88
128.90
128.84
128.76
117.41
115.35
113.85
113.64
113.26
113.06
77.48
77.16
76.84
55.56
36.18
34.43
33.99
32.00
31.39
29.99
210 200 190 180 170 160 150 140 130 120 110 100 90 80 70 60 50 40 30 20 10 0
f1 (ppm)

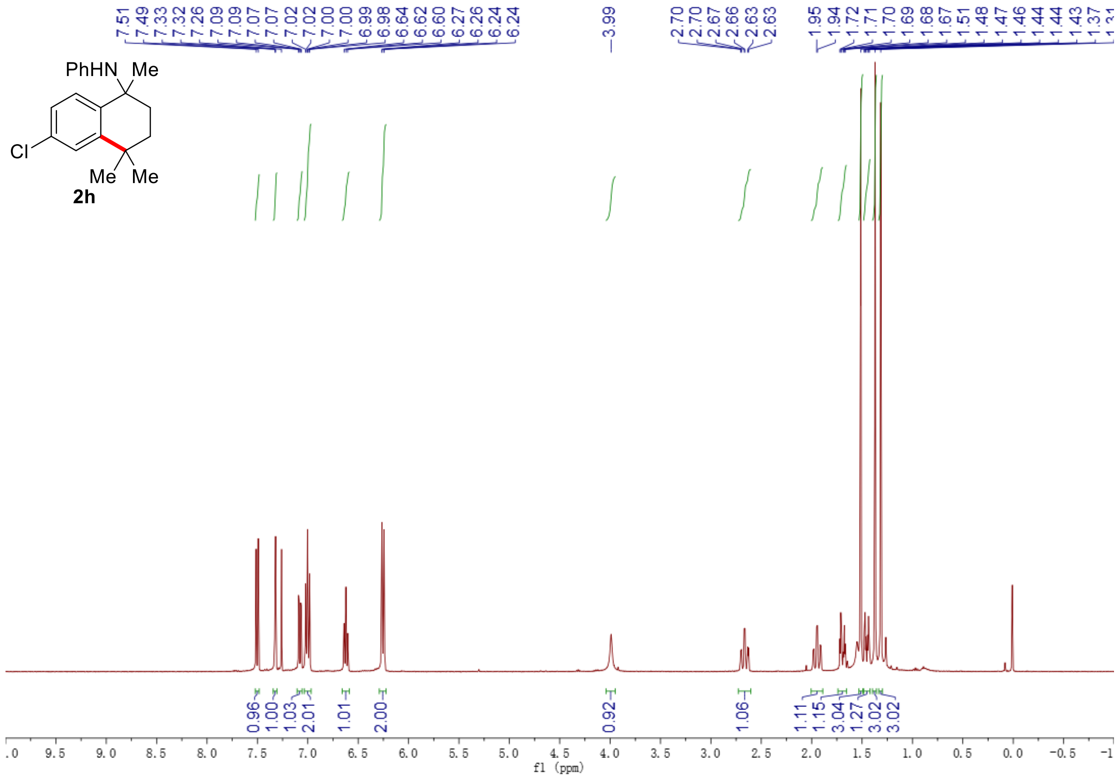
PhHN Me
Cl
Me Me
2h
7.51
7.49
7.33
7.32
7.26
7.09
7.09
7.07
7.07
7.02
7.02
7.00
7.00
6.99
6.98
6.64
6.62
6.60
6.27
6.26
6.24
6.24
3.99
2.70
2.70
2.67
2.66
2.63
2.63
1.95
1.94
1.72
1.71
1.70
1.69
1.68
1.67
1.51
1.48
1.47
1.46
1.44
1.44
1.43
1.37
0.96
1.00
1.03
2.01
1.01
2.00
0.92
1.06
1.11
1.15
3.04
1.27
3.02
3.02
9.5 9.0 8.5 8.0 7.5 7.0 6.5 6.0 5.5 5.0 4.5 4.0 3.5 3.0 2.5 2.0 1.5 1.0 0.5 0.0 -0.5
f1 (ppm)

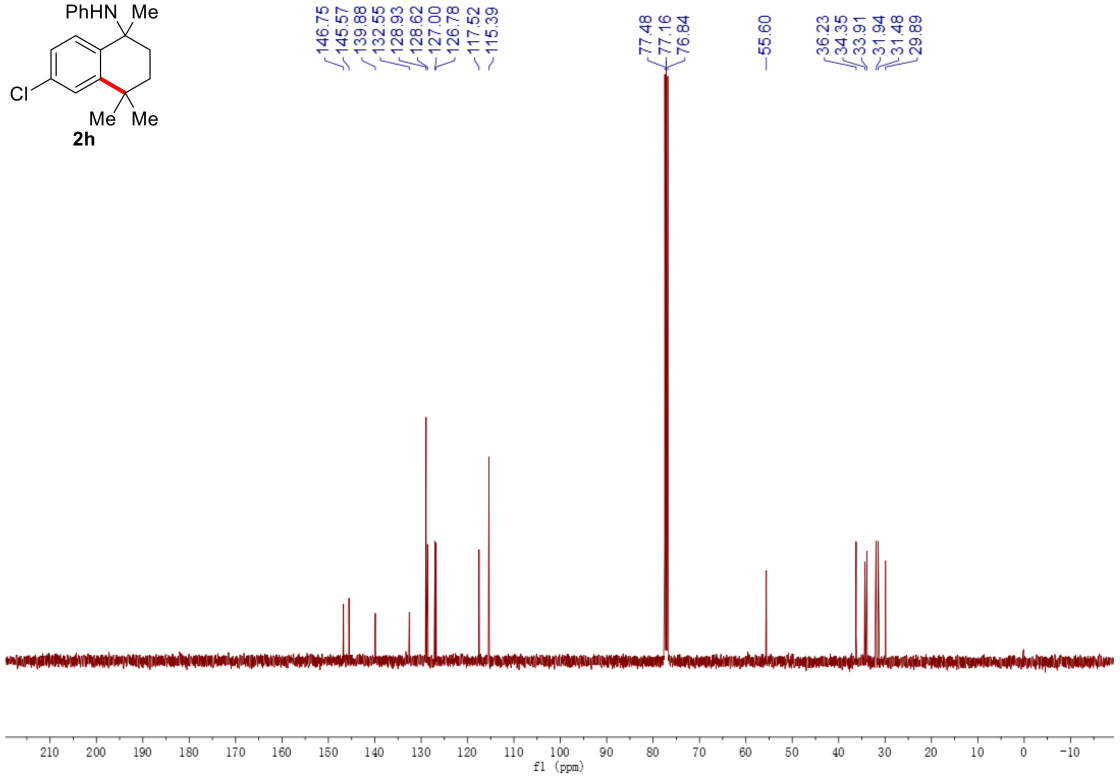
PhHN Me
Cl
Me Me
2h
146.75
145.57
139.88
132.55
128.93
128.62
127.00
126.78
117.52
115.39
77.48
77.16
76.84
55.60
36.23
34.35
33.91
31.94
31.48
29.89
f1 (ppm)

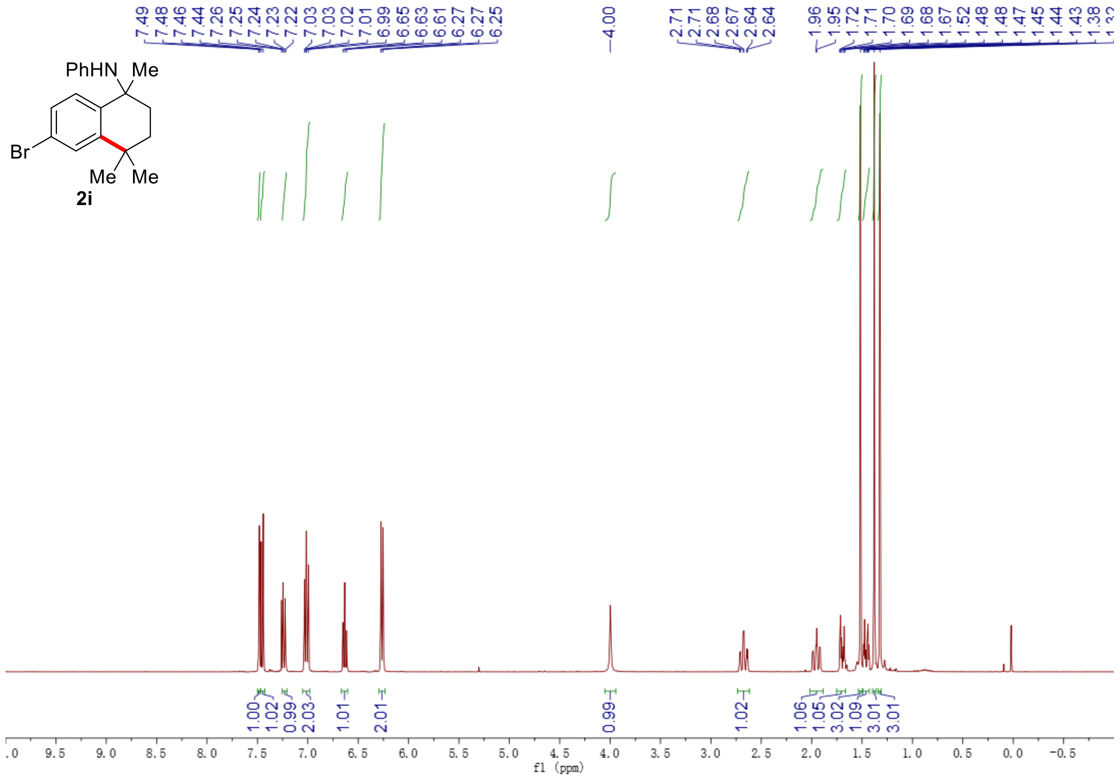
PhHN Me
Br
Me Me
2i
7.49
7.48
7.46
7.44
7.26
7.25
7.24
7.23
7.22
7.03
7.03
7.02
7.01
6.99
6.65
6.63
6.61
6.27
6.27
6.25
4.00
2.71
2.71
2.68
2.67
2.64
2.64
1.96
1.95
1.72
1.71
1.70
1.69
1.68
1.67
1.52
1.48
1.48
1.47
1.45
1.44
1.43
1.38
1.00
1.02
0.99
2.03
1.01
2.01
0.99
1.02
1.06
1.05
3.02
1.09
3.01
3.01
f1 (ppm)

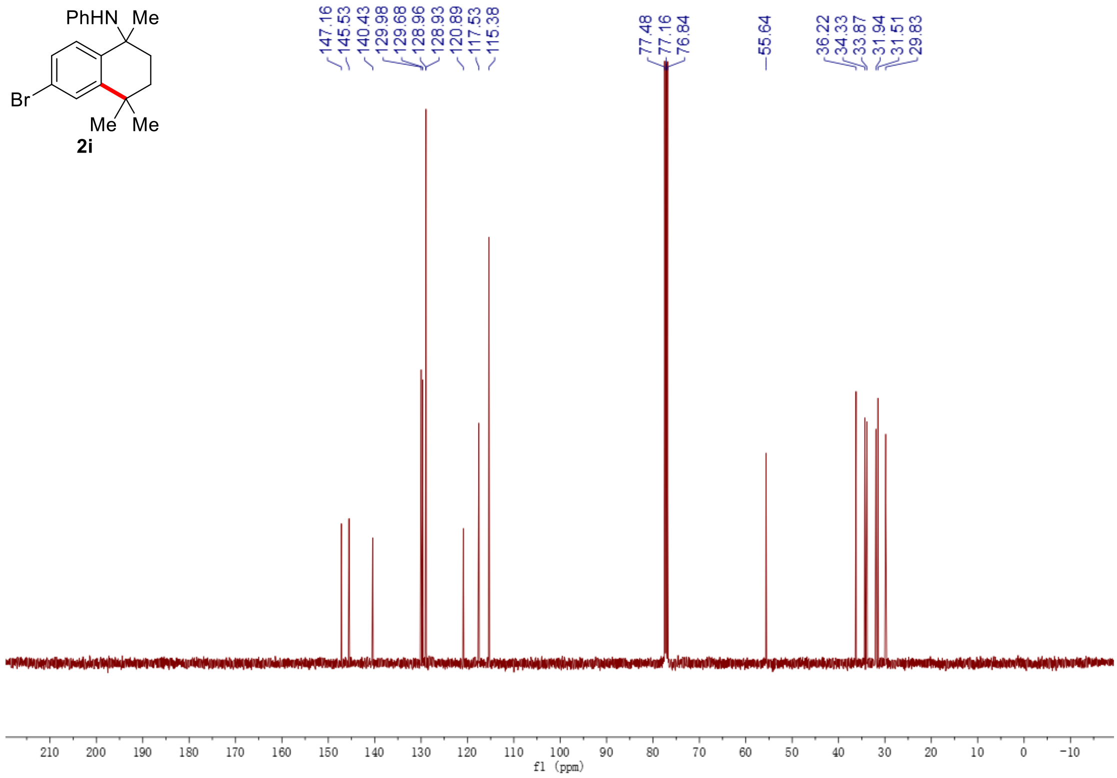
PhHN Me
Br
Me Me
2i
147.16
145.53
140.43
129.98
129.68
128.96
128.93
120.89
117.53
115.38
77.48
77.16
76.84
55.64
36.22
34.33
33.87
31.94
31.51
29.83
f1 (ppm)

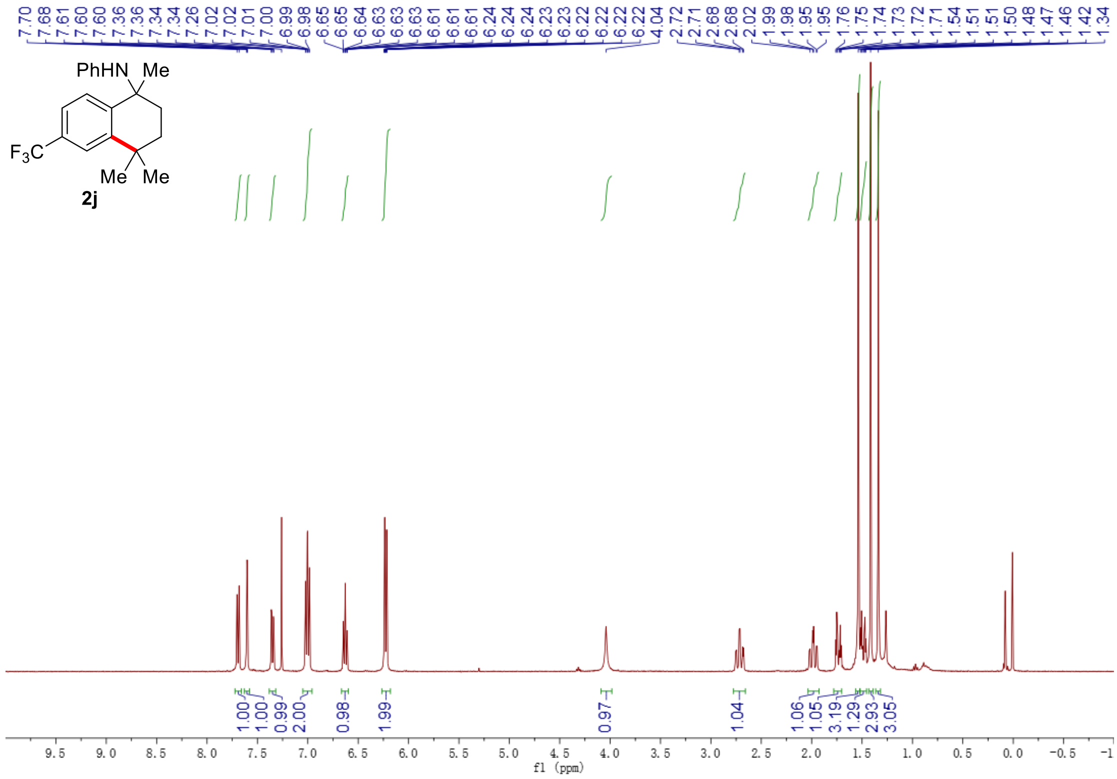
PhHN Me
F3C
Me Me
2j
7.70
7.68
7.61
7.60
7.60
7.36
7.36
7.34
7.34
7.26
7.02
7.02
7.01
7.00
6.99
6.98
6.65
6.65
6.64
6.63
6.63
6.61
6.61
6.61
6.24
6.24
6.24
6.23
6.23
6.22
6.22
6.22
6.22
4.04
2.72
2.71
2.68
2.68
2.02
1.99
1.98
1.95
1.95
1.76
1.75
1.74
1.73
1.72
1.71
1.54
1.51
1.50
1.48
1.47
1.46
1.42
1.34
1.00
1.00
0.99
2.00
0.98
1.99
0.97
1.04
1.06
1.05
3.19
1.29
2.93
3.05
f1 (ppm)

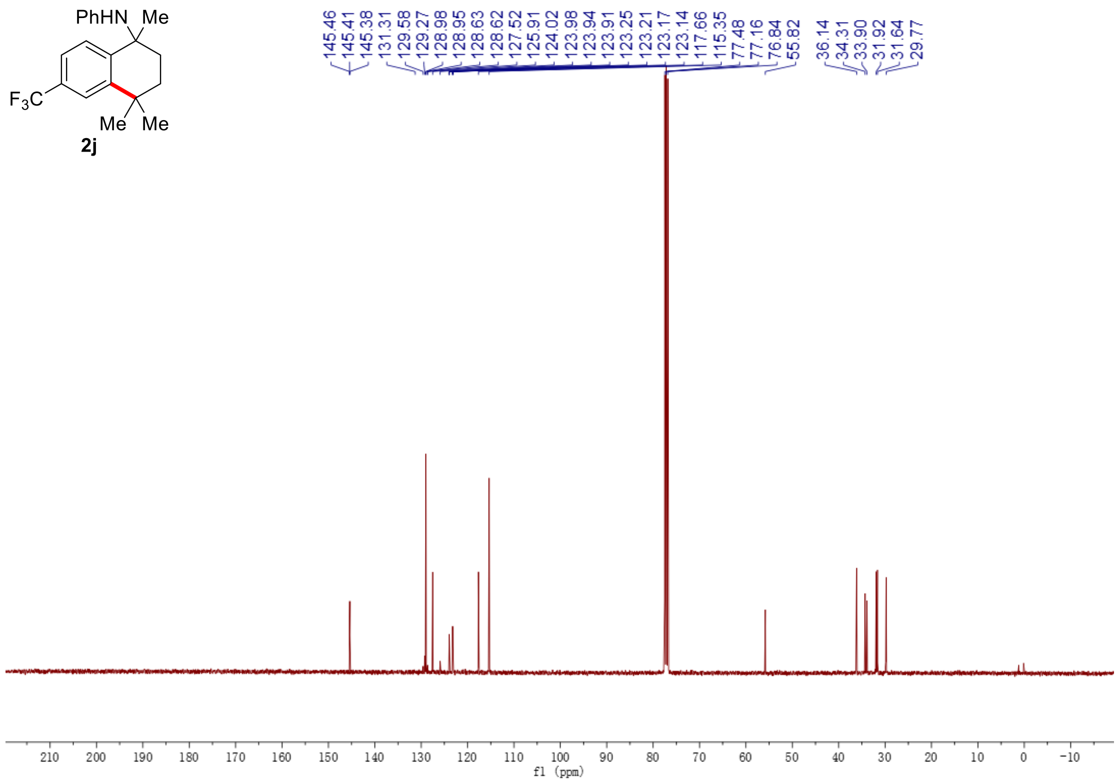
PhHN Me
F3C
Me Me
145.46
145.41
145.38
131.31
129.58
129.27
128.98
128.95
128.63
128.62
127.52
125.91
124.02
123.98
123.94
123.91
123.25
123.21
123.17
123.14
117.66
115.35
77.48
77.16
76.84
55.82
36.14
34.31
33.90
31.92
31.64
29.77
210 200 190 180 170 160 150 140 130 120 110 100 90 80 70 60 50 40 30 20 10 0 -10
f1 (ppm)

2j

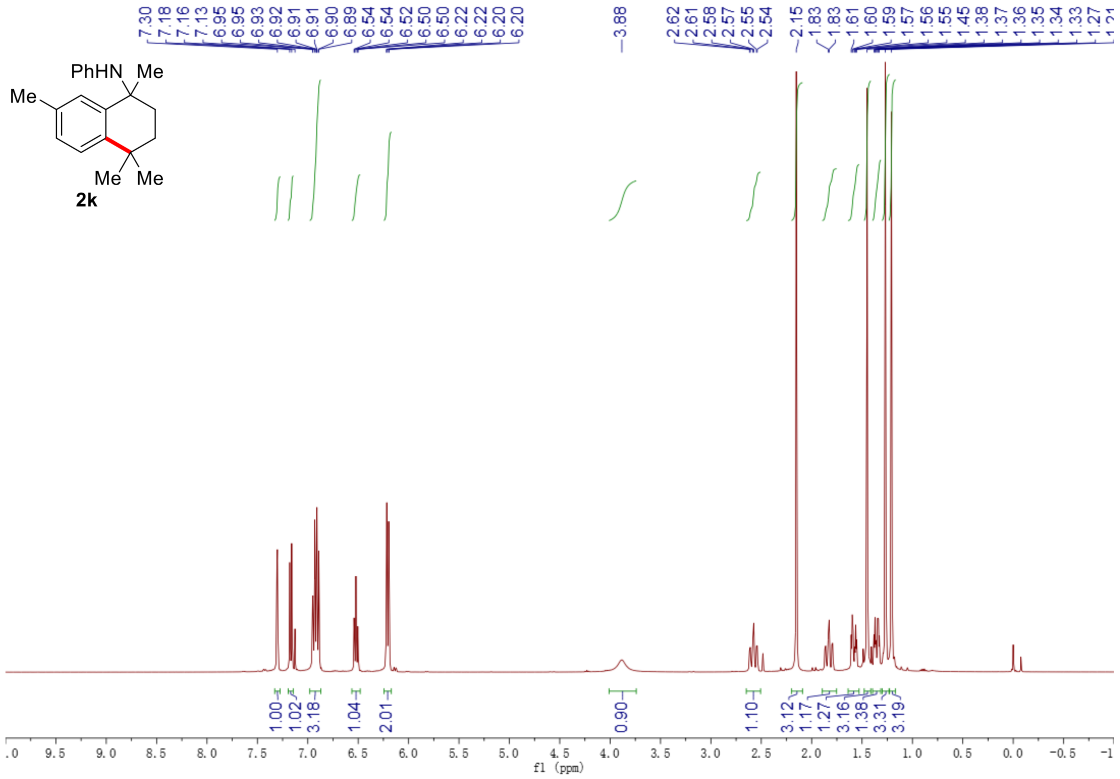
PhHN Me
Me
Me Me
7.30
7.18
7.16
7.13
6.95
6.93
6.92
6.91
6.90
6.89
6.54
6.54
6.52
6.50
6.50
6.22
6.22
6.20
6.20
3.88
2.62
2.61
2.58
2.57
2.55
2.54
2.15
1.83
1.83
1.61
1.60
1.59
1.57
1.56
1.55
1.45
1.38
1.37
1.36
1.35
1.34
1.33
1.27
1.00
1.02
3.18
1.04
2.01
0.90
1.10
3.12
1.17
1.27
3.16
1.38
3.31
3.19
9.5 9.0 8.5 8.0 7.5 7.0 6.5 6.0 5.5 5.0 4.5 4.0 3.5 3.0 2.5 2.0 1.5 1.0 0.5 0.0 -0.5
f1 (ppm)

2k

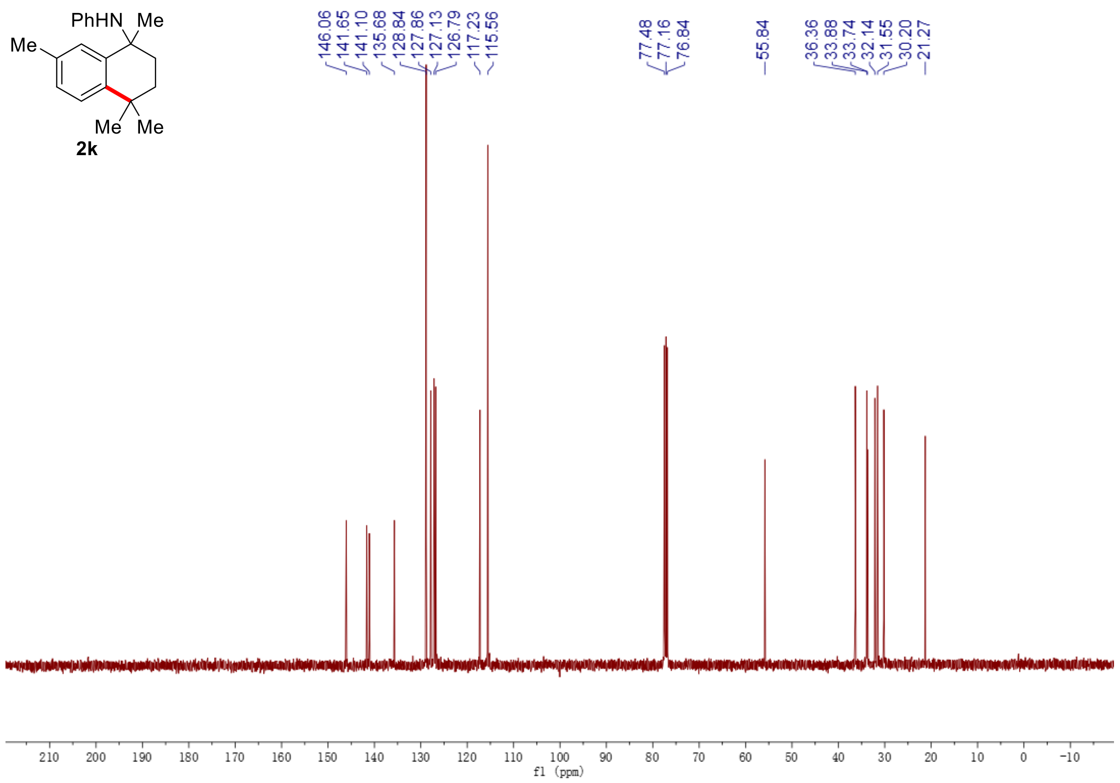
PhHN Me
Me
Me Me
2k
146.06
141.65
141.10
135.68
128.84
127.86
127.13
126.79
117.23
115.56
77.48
77.16
76.84
55.84
36.36
33.88
33.74
32.14
31.55
30.20
21.27
210 200 190 180 170 160 150 140 130 120 110 100 90 80 70 60 50 40 30 20 10 0 -10
f1 (ppm)

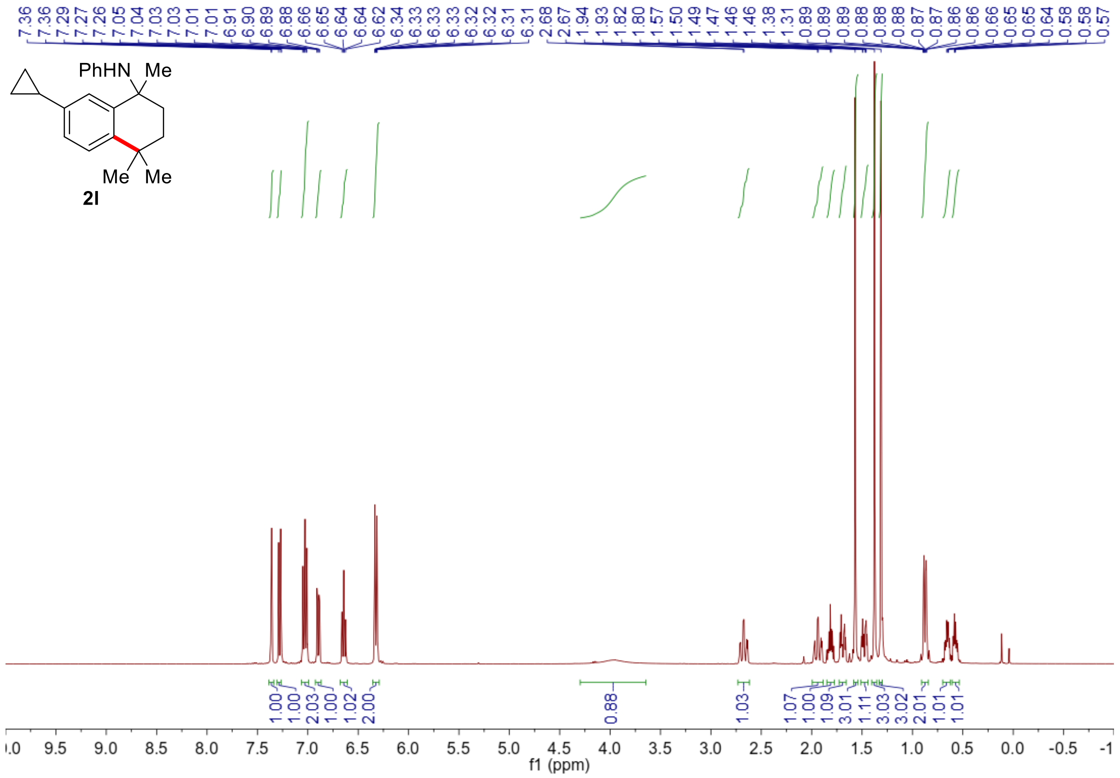
7.36
7.36
7.29
7.27
7.26
7.05
7.04
7.03
7.03
7.01
7.01
6.91
6.90
6.89
6.88
6.66
6.65
6.64
6.64
6.62
6.34
6.33
6.33
6.33
6.32
6.32
6.31
6.31
2.68
2.67
1.94
1.93
1.82
1.80
1.57
1.50
1.49
1.47
1.46
1.46
1.38
1.31
0.89
0.89
0.89
0.88
0.88
0.88
0.87
0.87
0.86
0.86
0.66
0.65
0.65
0.64
0.58
0.58
0.57
PhHN Me
Me Me
2l
1.00
1.00
2.03
1.00
1.02
2.00
0.88
1.03
1.07
1.00
1.09
3.01
1.11
3.03
3.02
2.01
1.01
1.01
10.0 9.5 9.0 8.5 8.0 7.5 7.0 6.5 6.0 5.5 5.0 4.5 4.0 3.5 3.0 2.5 2.0 1.5 1.0 0.5 0.0 -0.5 -1
f1 (ppm)

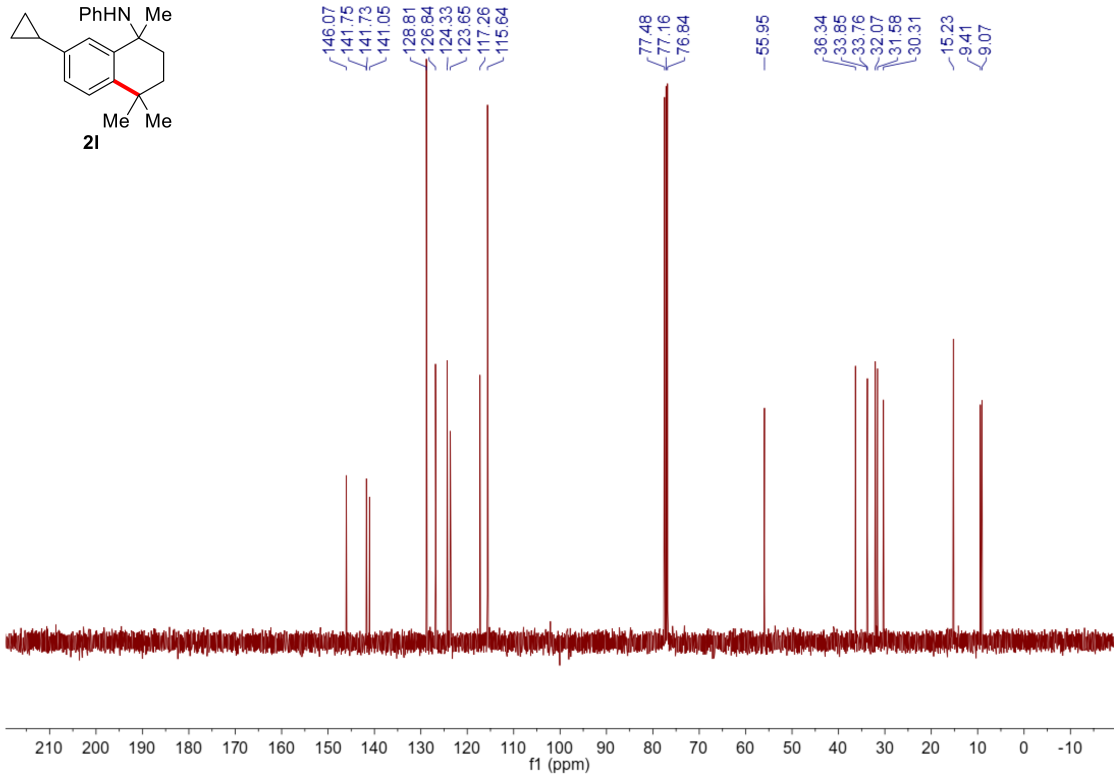
PhHN Me
Me Me
2l
146.07
141.75
141.73
141.05
128.81
126.84
124.33
123.65
117.26
115.64
77.48
77.16
76.84
55.95
36.34
33.85
33.76
32.07
31.58
30.31
15.23
9.41
9.07
f1 (ppm)

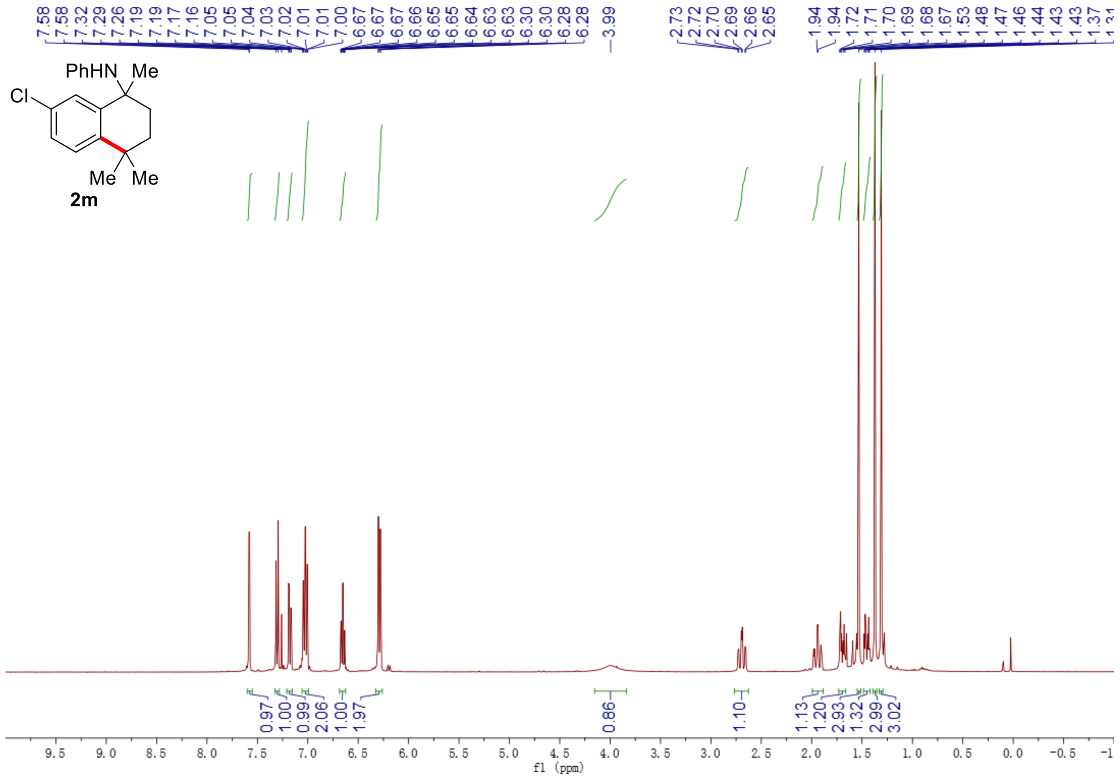
PhHN Me
Cl
Me Me
2m
3.99
0.97
1.00
0.99
2.06
1.00
1.97
0.86
1.10
1.13
1.20
2.93
1.32
2.99
3.02
f1 (ppm)

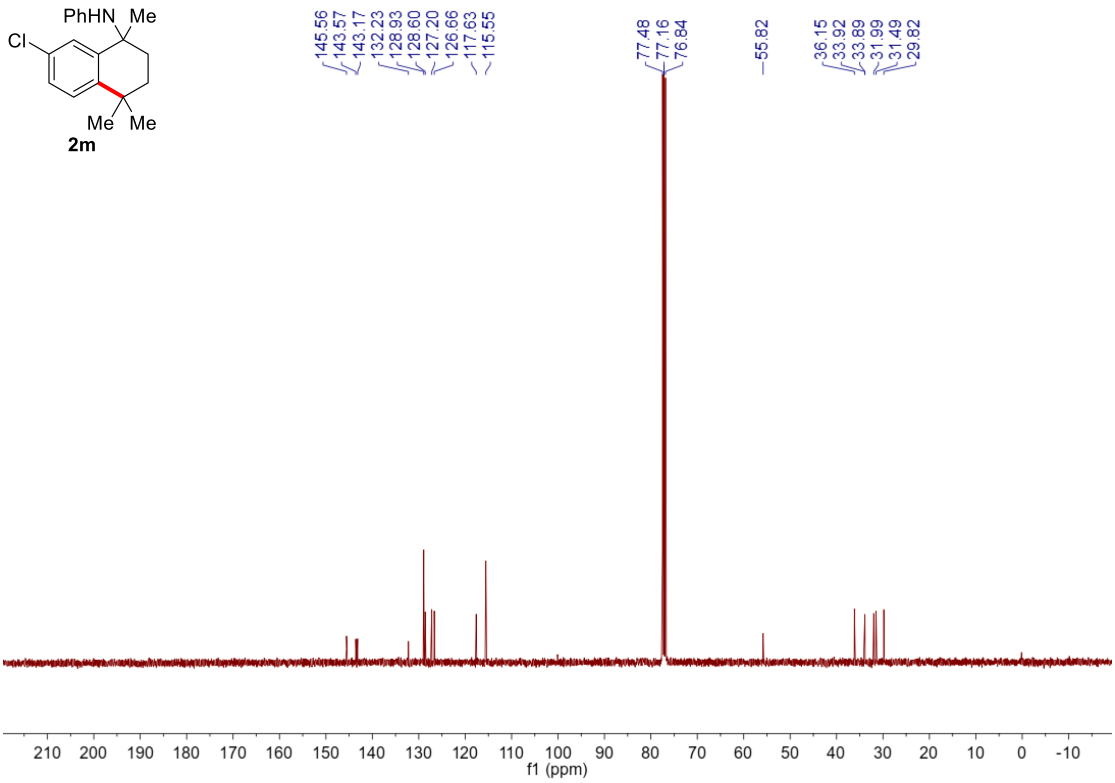
PhHN Me
Cl
Me Me
145.56
143.57
143.17
132.23
128.93
128.60
127.20
126.66
117.63
115.55
77.48
77.16
76.84
55.82
36.15
33.92
33.89
31.99
31.49
29.82
210 200 190 180 170 160 150 140 130 120 110 100 90 80 70 60 50 40 30 20 10 0 -10
f1 (ppm)

2m

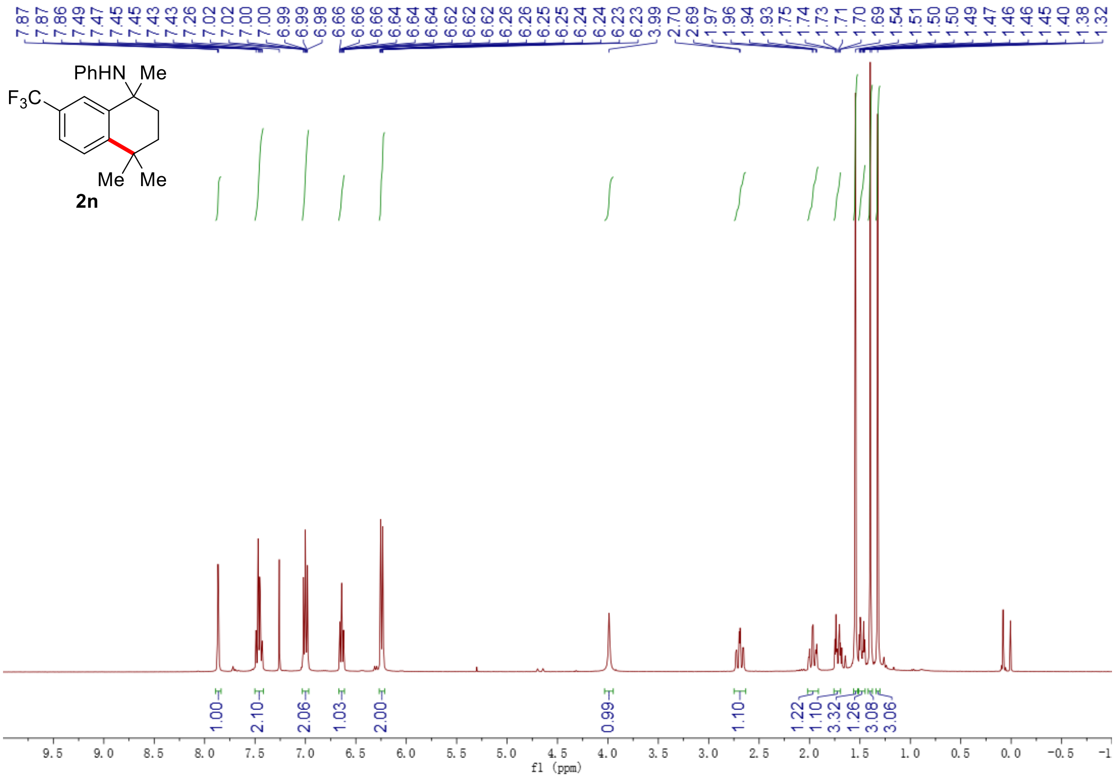
PhHN Me
F_3C
Me Me
7.87
7.87
7.86
7.49
7.47
7.45
7.45
7.43
7.43
7.26
7.02
7.02
7.00
7.00
6.99
6.99
6.98
6.66
6.66
6.64
6.64
6.62
6.62
6.26
6.26
6.25
6.25
6.24
6.24
6.23
6.23
3.99
2.70
2.69
1.97
1.96
1.94
1.93
1.75
1.74
1.73
1.71
1.70
1.69
1.54
1.51
1.50
1.50
1.49
1.47
1.46
1.46
1.45
1.40
1.38
1.32
1.00
2.10
2.06
1.03
2.00
0.99
1.10
1.22
1.10
3.32
1.26
3.08
3.06
9.5 9.0 8.5 8.0 7.5 7.0 6.5 6.0 5.5 5.0 4.5 4.0 3.5 3.0 2.5 2.0 1.5 1.0 0.5 0.0 -0.5 -1
f1 (ppm)

2n

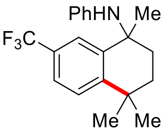
PhHN
Me
F_3C
Me
Me

2n

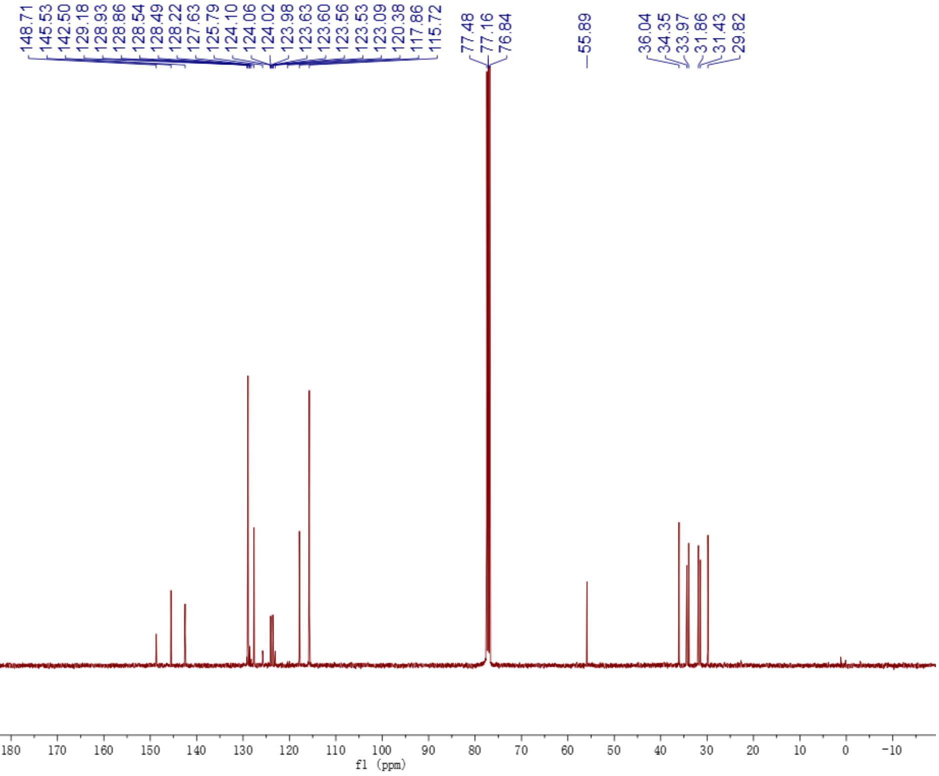
148.71
145.53
142.50
129.18
128.93
128.86
128.54
128.49
128.22
127.63
125.79
124.10
124.06
124.02
123.98
123.63
123.60
123.56
123.53
123.09
120.38
117.86
115.72
77.48
77.16
76.84
55.89
36.04
34.35
33.97
31.86
31.43
29.82
210 200 190 180 170 160 150 140 130 120 110 100 90 80 70 60 50 40 30 20 10 0 -10
f1 (ppm)

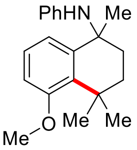
PhHN
Me
Me
O
Me
Me

2o

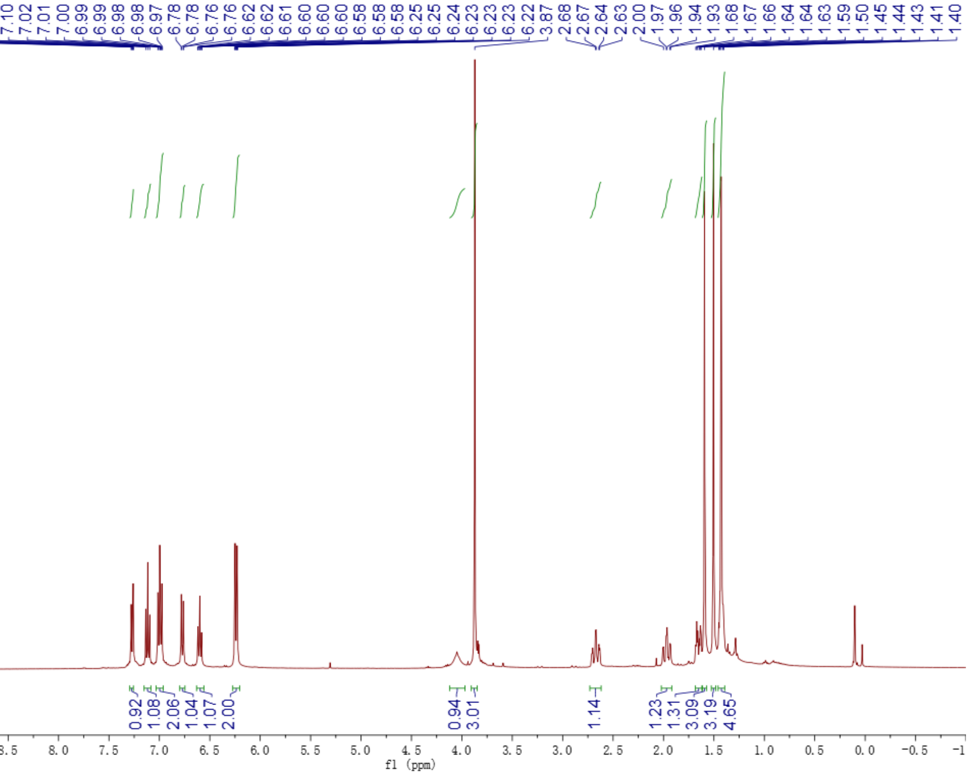
7.28
7.28
7.26
7.26
7.26
7.14
7.12
7.10
7.02
7.01
7.00
6.99
6.99
6.98
6.98
6.97
6.78
6.78
6.76
6.62
6.61
6.60
6.60
6.60
6.58
6.58
6.25
6.25
6.24
6.23
6.23
6.23
6.22
3.87
2.68
2.67
2.64
2.63
2.00
1.97
1.96
1.94
1.93
1.68
1.67
1.66
1.64
1.63
1.59
1.50
1.45
1.44
1.43
1.41
1.40
0.92
1.08
2.06
1.04
1.07
2.00
0.94
3.01
1.14
1.23
1.31
3.09
3.19
4.65
.0 9.5 9.0 8.5 8.0 7.5 7.0 6.5 6.0 5.5 5.0 4.5 4.0 3.5 3.0 2.5 2.0 1.5 1.0 0.5 0.0 -0.5 -1
f1 (ppm)

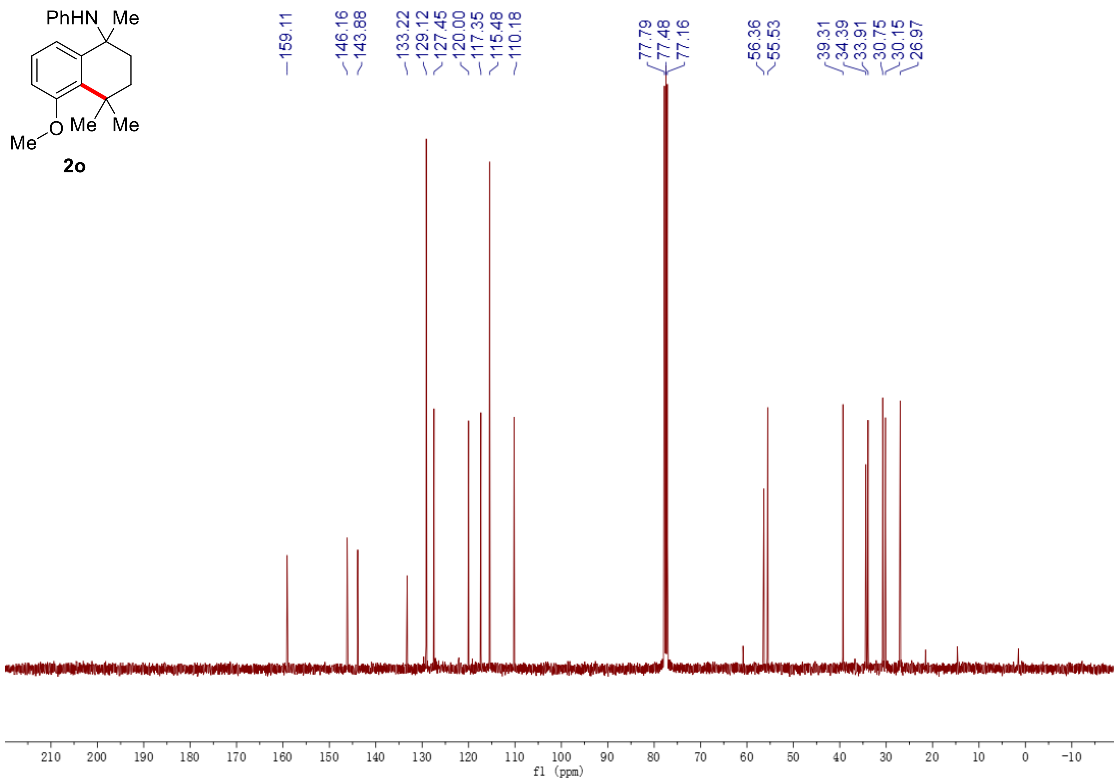
PhHN
Me
O
Me
Me
Me
2o
159.11
146.16
143.88
133.22
129.12
127.45
120.00
117.35
115.48
110.18
77.79
77.48
77.16
56.36
55.53
39.31
34.39
33.91
30.75
30.15
26.97
210 200 190 180 170 160 150 140 130 120 110 100 90 80 70 60 50 40 30 20 10 0 -10
f1 (ppm)

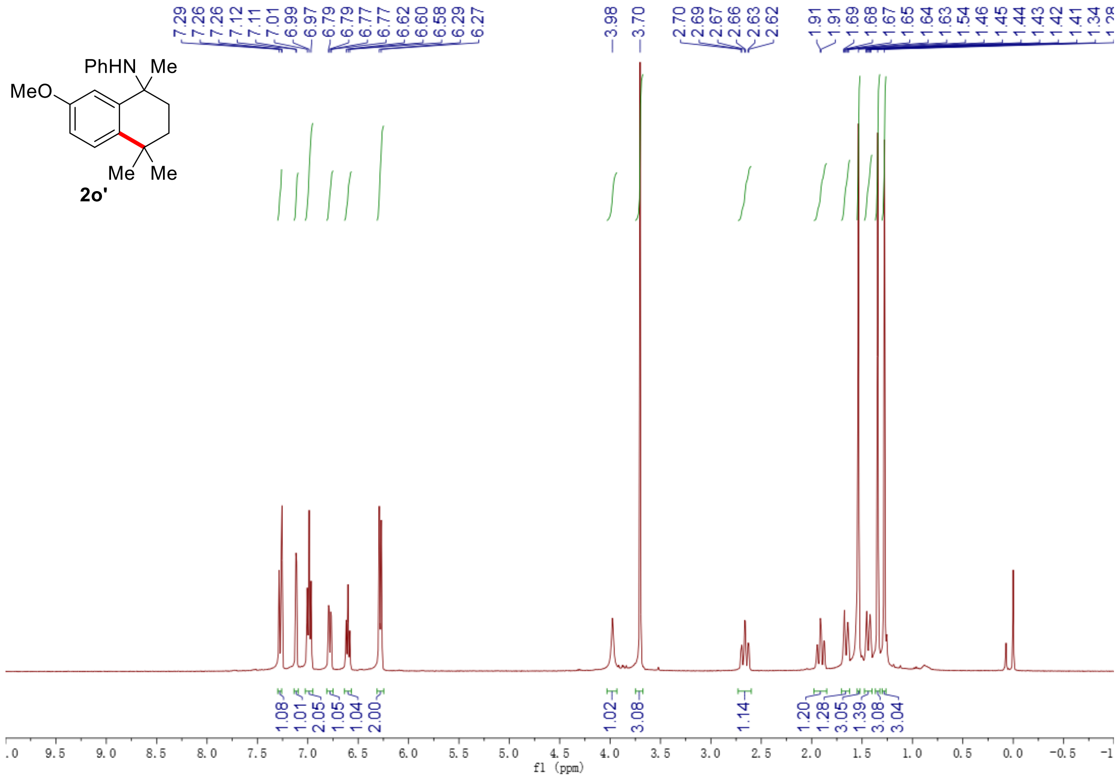
PhHN
Me
MeO
Me
Me
2o'
7.29
7.26
7.26
7.12
7.11
7.01
6.99
6.97
6.79
6.79
6.77
6.77
6.62
6.60
6.58
6.29
6.27
3.98
3.70
2.70
2.69
2.67
2.66
2.63
2.62
1.91
1.91
1.69
1.68
1.67
1.65
1.64
1.63
1.54
1.46
1.45
1.44
1.43
1.42
1.41
1.34
1.08
1.01
2.05
1.05
1.04
2.00
1.02
3.08
1.14
1.20
1.28
3.05
1.39
3.08
3.04
9.5 9.0 8.5 8.0 7.5 7.0 6.5 6.0 5.5 5.0 4.5 4.0 3.5 3.0 2.5 2.0 1.5 1.0 0.5 0.0 -0.5
f1 (ppm)

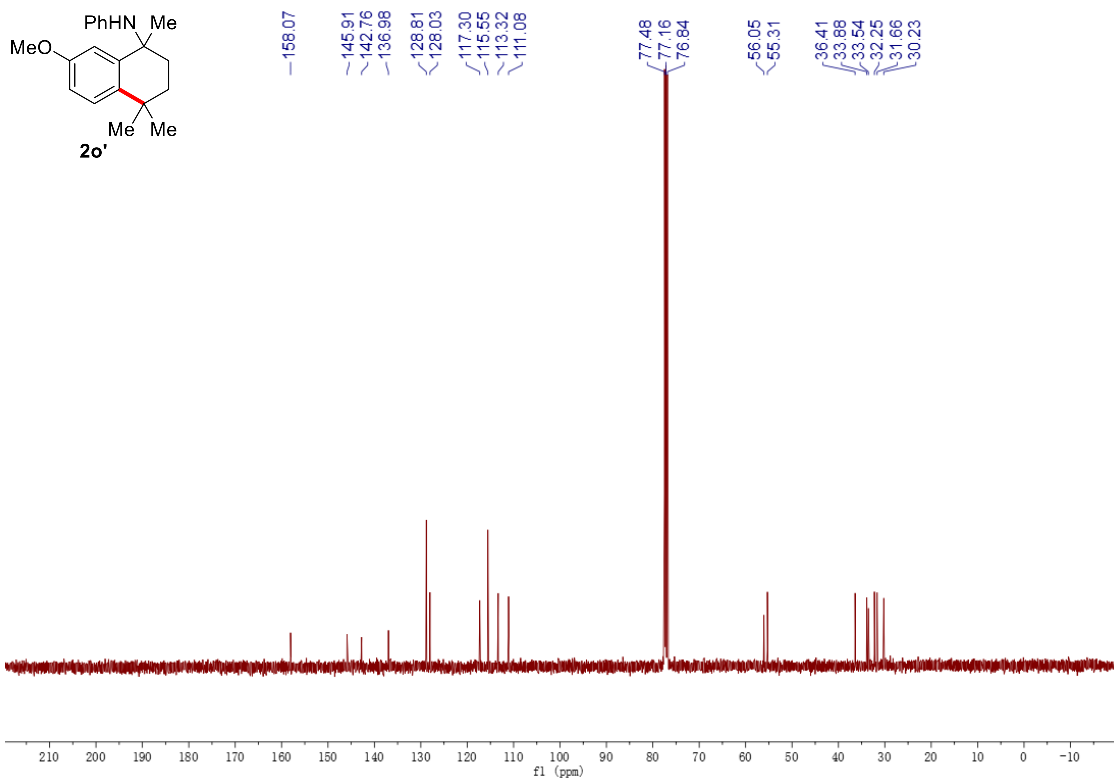

MeO
PhHN Me
Me Me
2o'
158.07
145.91
142.76
136.98
128.81
128.03
117.30
115.55
113.32
111.08
77.48
77.16
76.84
56.05
55.31
36.41
33.88
33.54
32.25
31.66
30.23
f1 (ppm)

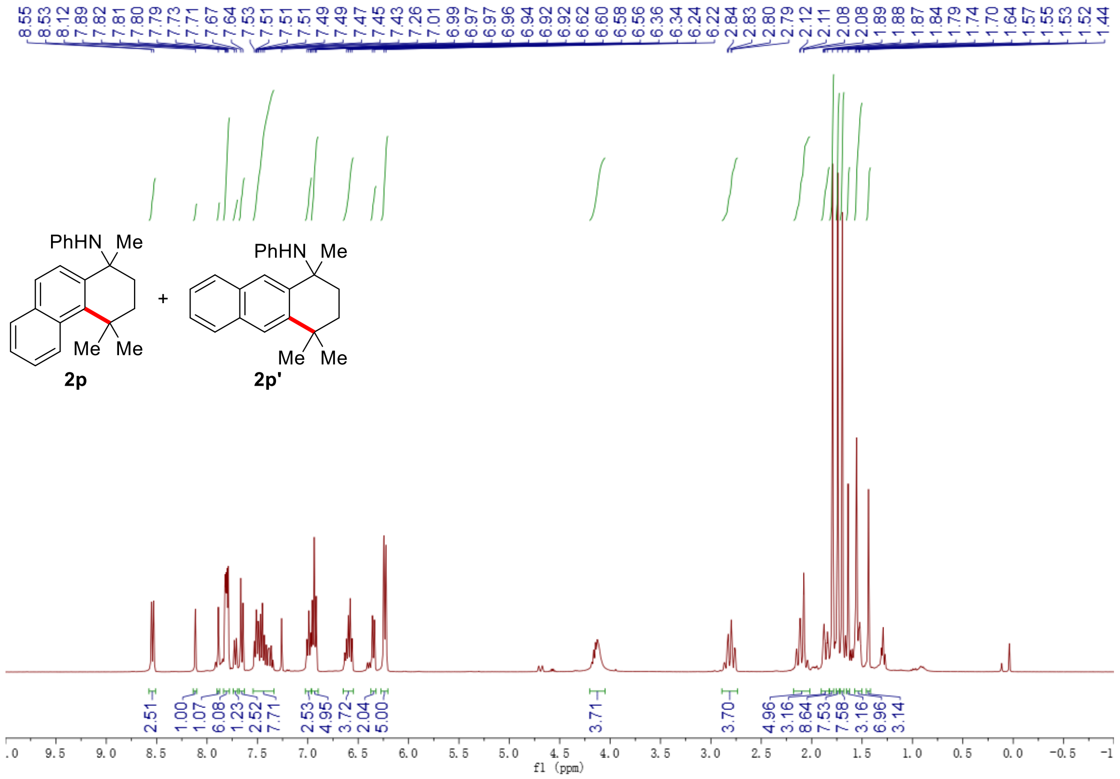

8.55 8.53 8.12 7.89 7.82 7.81 7.80 7.79 7.73 7.71 7.67 7.64 7.53 7.51 7.51 7.49 7.49 7.47 7.45 7.43 7.26 7.01 6.99 6.97 6.97 6.96 6.94 6.92 6.92 6.62 6.60 6.58 6.56 6.36 6.34 6.24 6.22 2.84 2.83 2.80 2.79 2.12 2.11 2.08 2.08 1.89 1.88 1.87 1.84 1.79 1.74 1.70 1.64 1.57 1.55 1.53 1.52 1.44
PhHN Me
Me Me
2p
+
PhHN Me
Me Me
2p'
2.51 1.00 1.07 6.08 1.23 2.52 7.71 2.53 4.95 3.72 2.04 5.00 3.71 3.70 4.96 3.16 8.64 7.53 7.58 3.16 6.96 3.14
f1 (ppm)

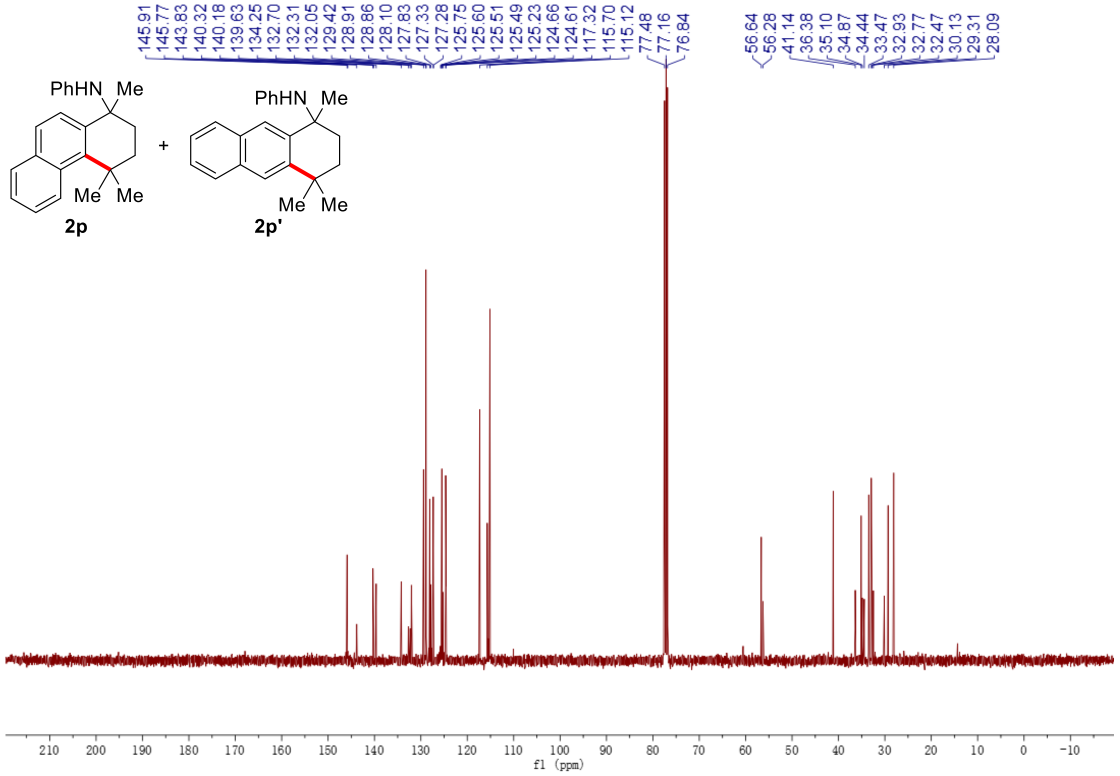
145.91
145.77
143.83
140.32
140.18
139.63
134.25
132.70
132.31
132.05
129.42
128.91
128.86
128.10
127.83
127.33
127.28
125.75
125.60
125.51
125.49
125.23
124.66
124.61
117.32
115.70
115.12
77.48
77.16
76.84
56.64
56.28
41.14
36.38
35.10
34.87
34.44
33.47
32.93
32.77
32.47
30.13
29.31
28.09
PhHN Me
Me Me
2p
+
PhHN Me
Me Me
2p'
f1 (ppm)

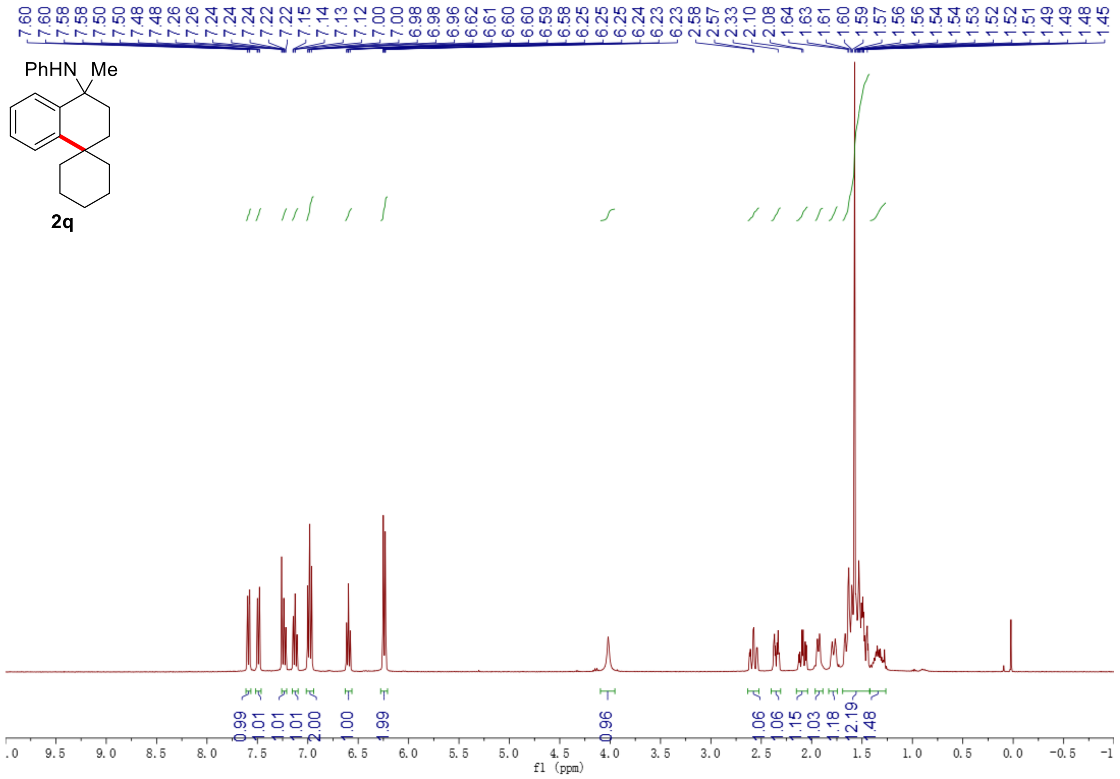
7.60
7.60
7.58
7.58
7.50
7.50
7.48
7.48
7.26
7.26
7.24
7.24
7.22
7.22
7.15
7.14
7.13
7.12
7.00
7.00
6.98
6.98
6.96
6.62
6.61
6.60
6.60
6.59
6.58
6.25
6.25
6.24
6.23
6.23
2.58
2.57
2.33
2.10
2.08
1.64
1.63
1.61
1.60
1.59
1.57
1.56
1.56
1.54
1.54
1.53
1.52
1.52
1.51
1.49
1.49
1.48
1.45
PhHN Me
2q
0.99
1.01
1.01
1.01
2.00
1.00
1.99
0.96
1.06
1.06
1.15
1.03
1.18
12.19
1.48
f1 (ppm)

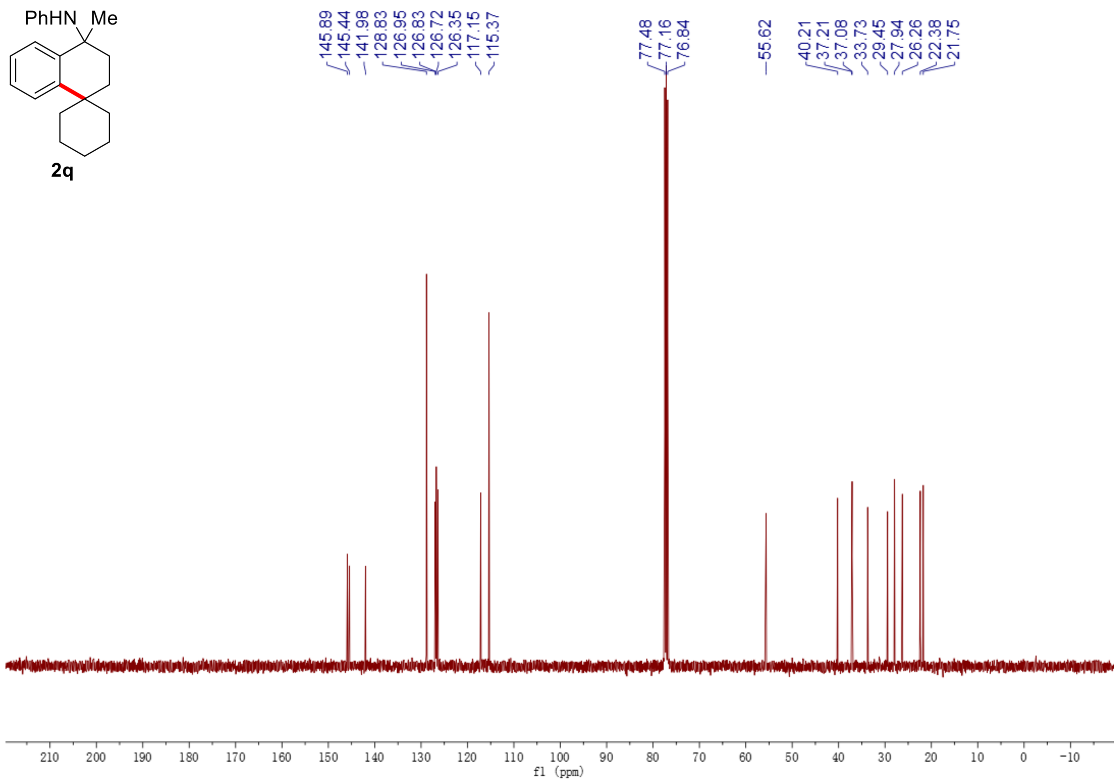
PhHN Me
2q
145.89
145.44
141.98
128.83
126.95
126.83
126.72
126.35
117.15
115.37
77.48
77.16
76.84
55.62
40.21
37.21
37.08
33.73
29.45
27.94
26.26
22.38
21.75
f1 (ppm)

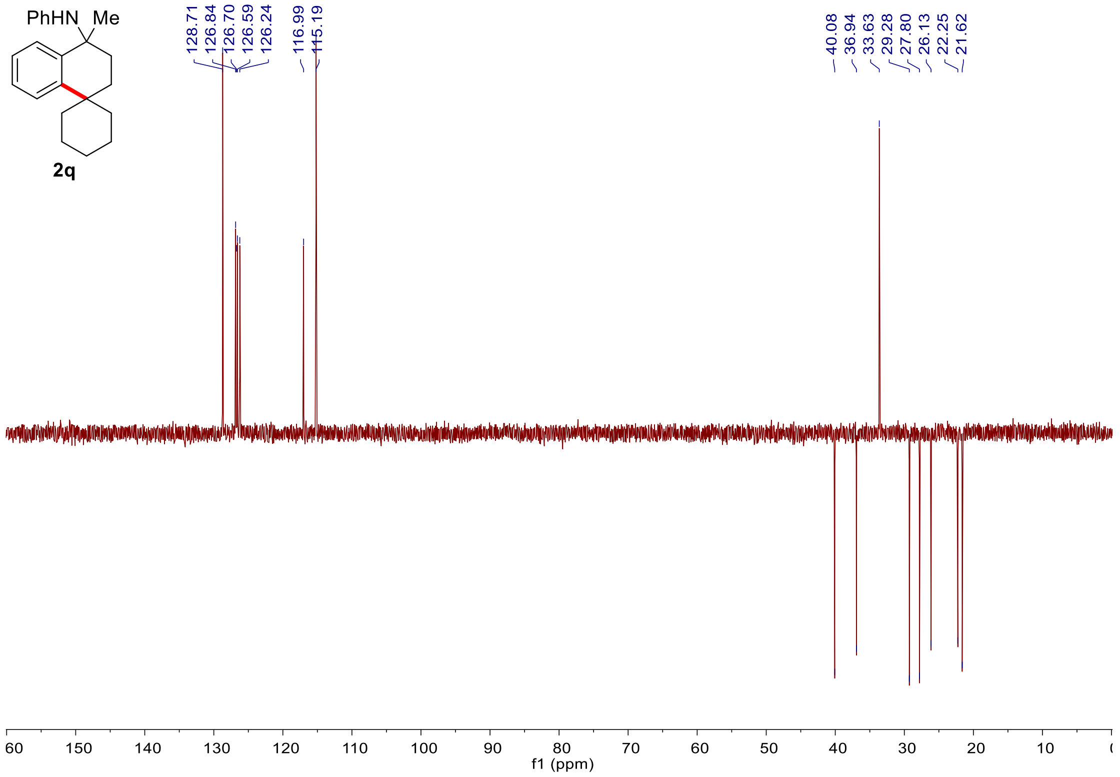
PhHN Me
2q
128.71
126.84
126.70
126.59
126.24
116.99
115.19
40.08
36.94
33.63
29.28
27.80
26.13
22.25
21.62
f1 (ppm)

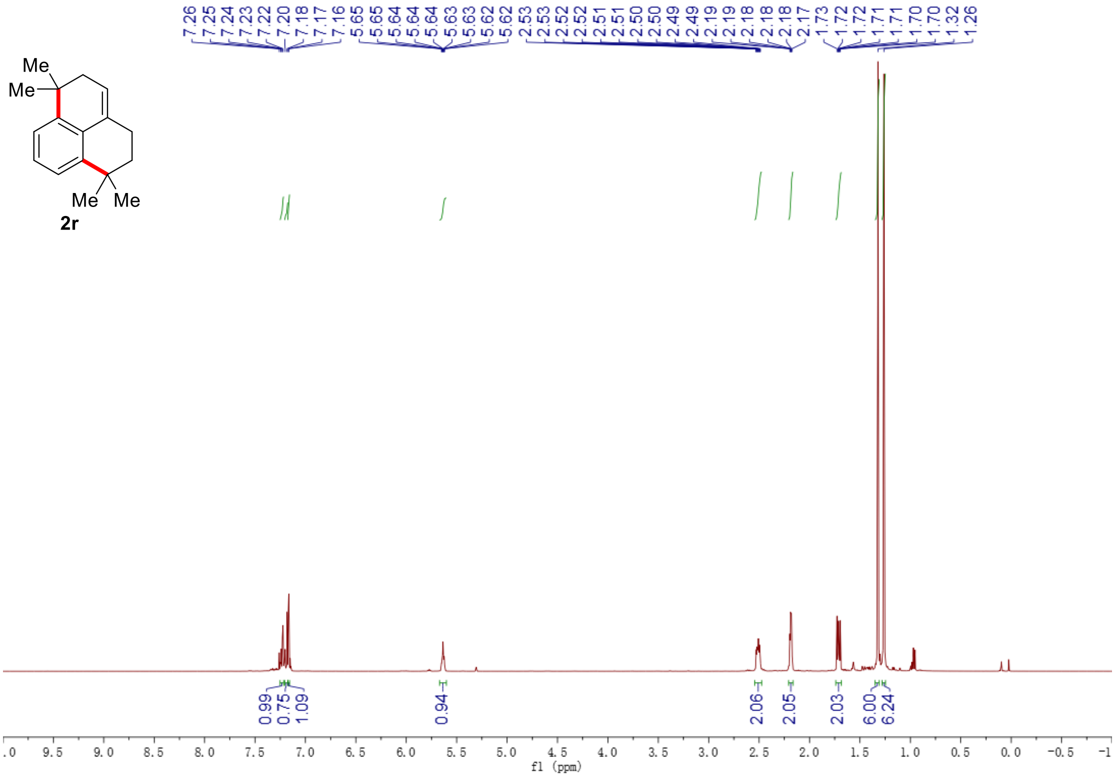
Me
Me
Me
Me
2r
0.99
0.75
1.09
0.94
2.06
2.05
2.03
6.00
6.24
f1 (ppm)

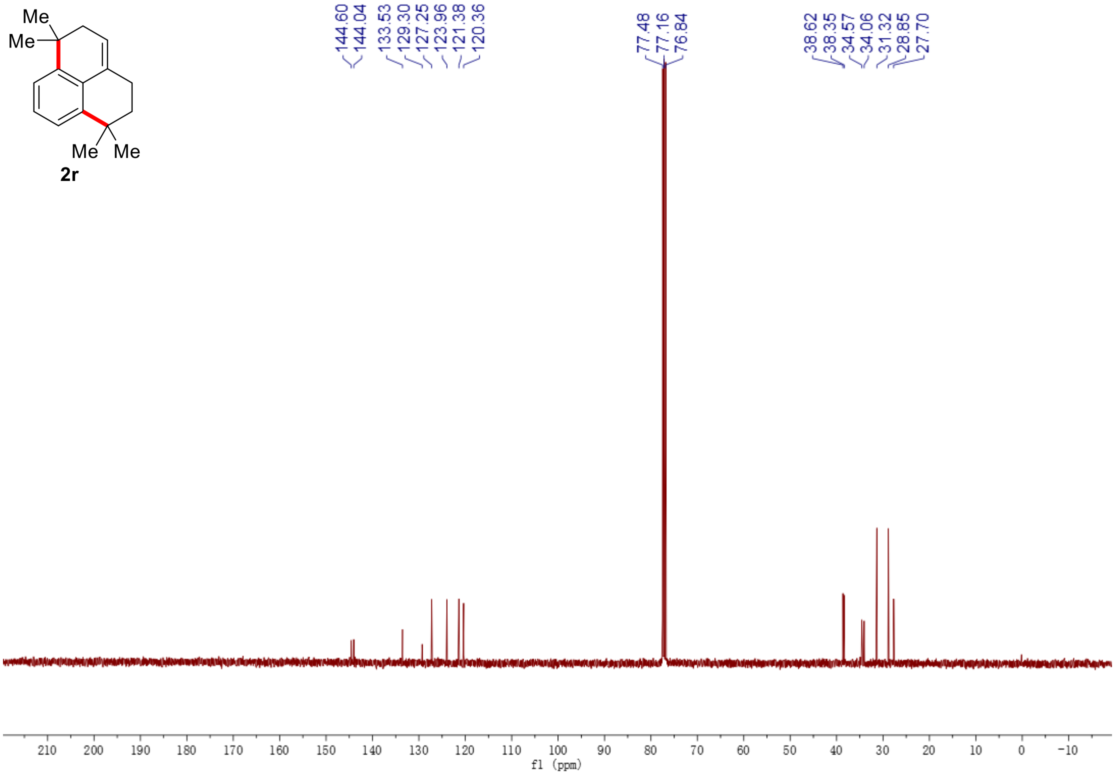
Me
Me
Me
Me
2r
144.60
144.04
133.53
129.30
127.25
123.96
121.38
120.36
77.48
77.16
76.84
38.62
38.35
34.57
34.06
31.32
28.85
27.70
f1 (ppm)

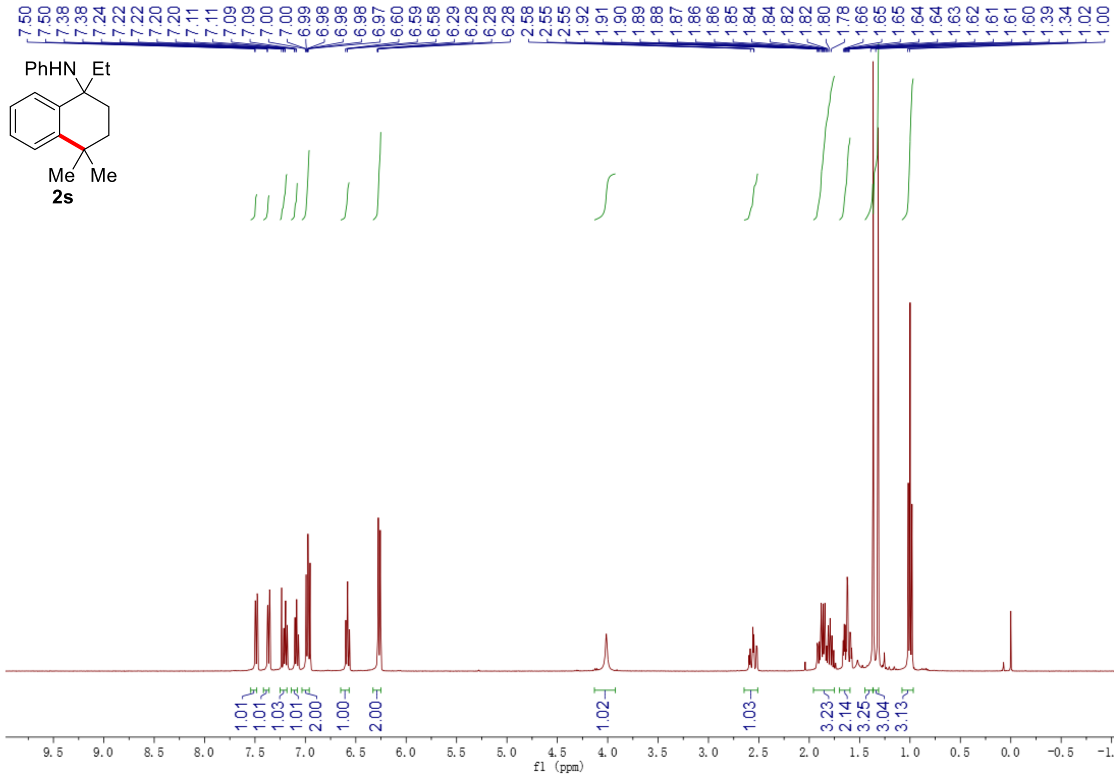
PhHN Et
Me Me
2s
7.50 7.50 7.38 7.24 7.22 7.22 7.20 7.20 7.11 7.09 7.00 7.00 6.99 6.98 6.98 6.97 6.60 6.59 6.58 6.29 6.28 6.28 6.28 2.58 2.55 2.55 1.92 1.91 1.90 1.89 1.88 1.87 1.86 1.86 1.85 1.84 1.84 1.82 1.82 1.80 1.78 1.66 1.65 1.65 1.64 1.64 1.63 1.62 1.61 1.61 1.60 1.39 1.34 1.02 1.00
1.01 1.01 1.03 1.01 2.00 1.00 2.00 1.02 1.03 3.23 2.14 3.25 3.04 3.13
f1 (ppm)

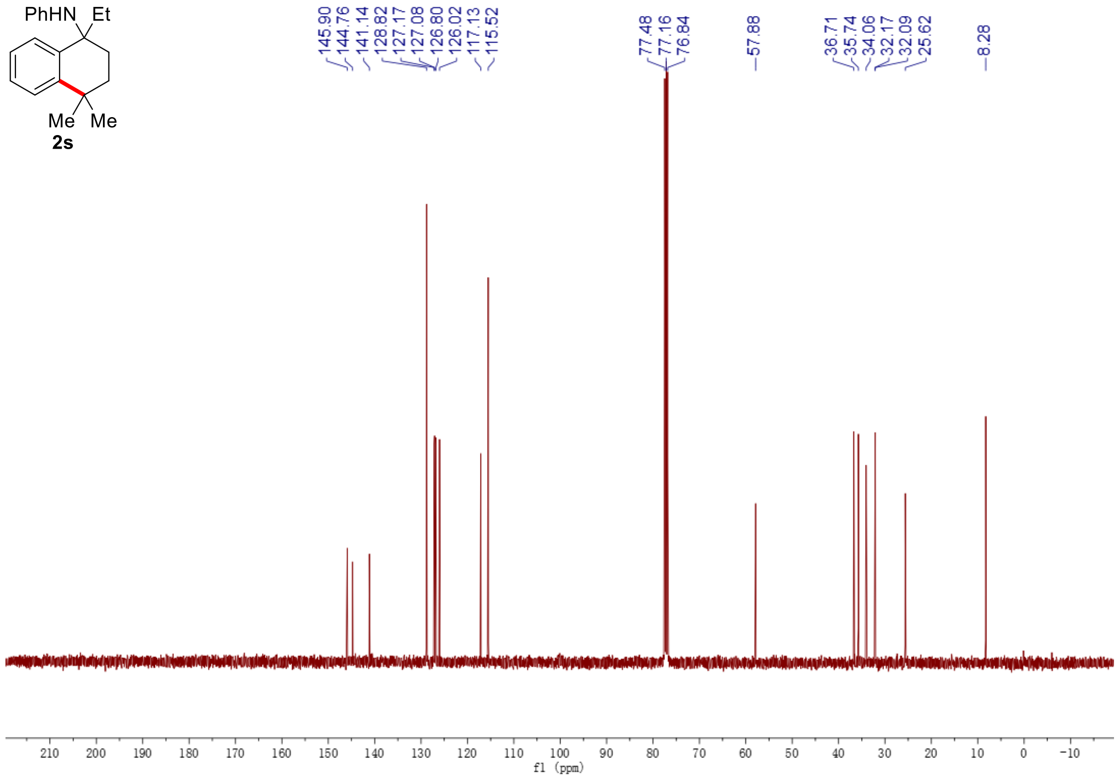
PhHN Et
Me Me
2s
145.90 144.76 141.14 128.82 127.17 127.08 126.80 126.02 117.13 115.52
77.48 77.16 76.84
57.88
36.71 35.74 34.06 32.17 32.09 25.62
8.28
f1 (ppm)

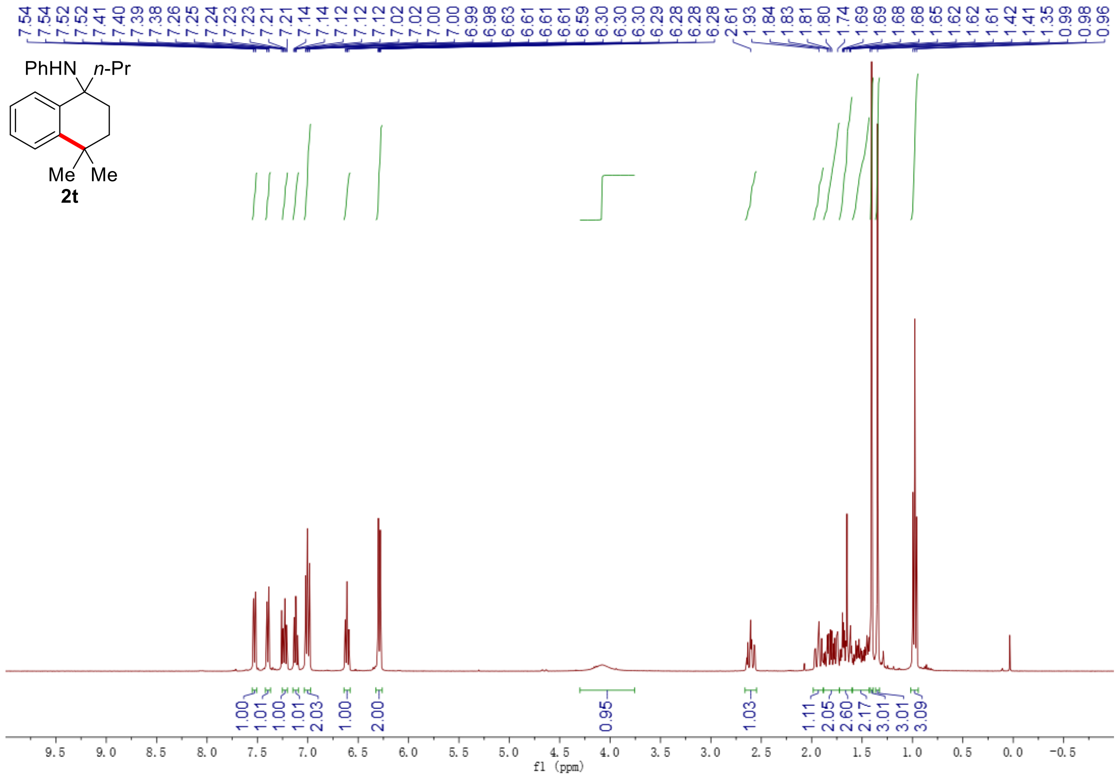
7.54 7.54 7.52 7.41 7.40 7.39 7.38 7.26 7.25 7.24 7.23 7.21 7.21 7.14 7.14 7.12 7.12 7.02 7.02 7.00 7.00 6.99 6.98 6.63 6.61 6.61 6.61 6.59 6.30 6.30 6.30 6.29 6.28 6.28 6.28 2.61 1.93 1.84 1.83 1.81 1.80 1.74 1.69 1.69 1.68 1.68 1.65 1.62 1.62 1.61 1.42 1.41 1.35 0.99 0.98 0.96
PhHN n-Pr
Me Me
2t
1.00 1.01 1.00 1.01 2.03 1.00 2.00 0.95 1.03 1.11 2.05 2.60 2.17 3.01 3.01 3.09
9.5 9.0 8.5 8.0 7.5 7.0 6.5 6.0 5.5 5.0 4.5 4.0 3.5 3.0 2.5 2.0 1.5 1.0 0.5 0.0 -0.5
f1 (ppm)

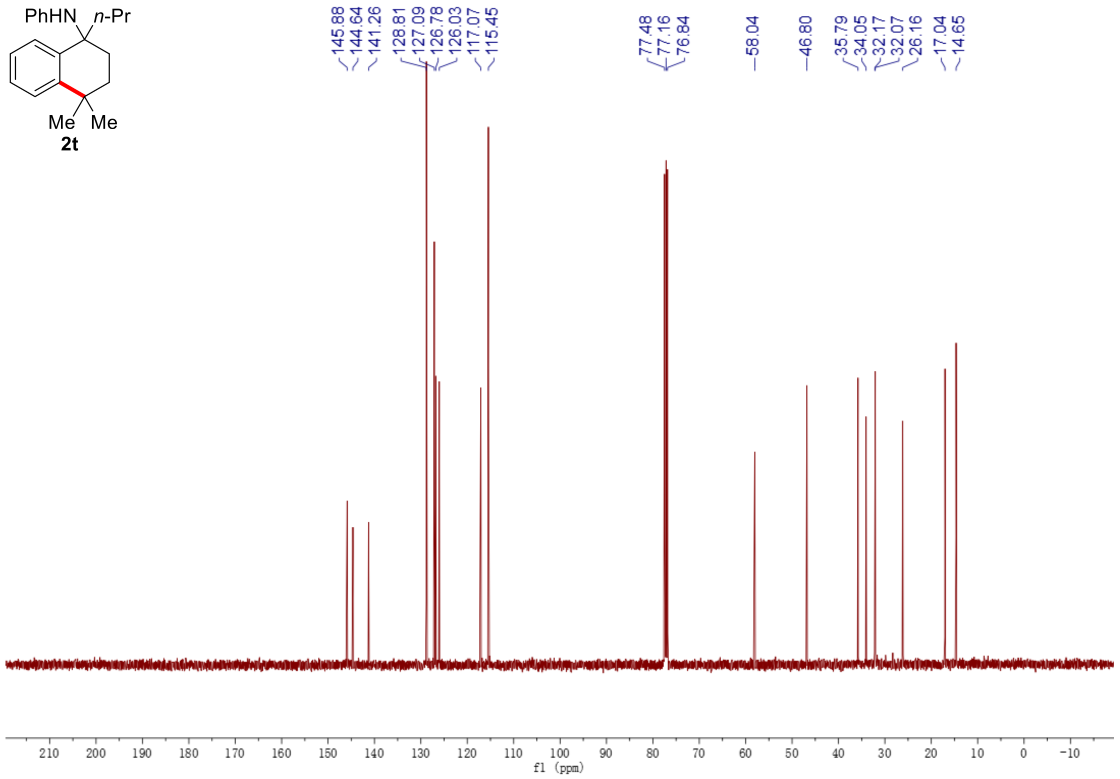
PhHN n-Pr
Me Me
2t
145.88 144.64 141.26 128.81 127.09 126.78 126.03 117.07 115.45
77.48 77.16 76.84
58.04
46.80
35.79 34.05 32.17 32.07 26.16 17.04 14.65
210 200 190 180 170 160 150 140 130 120 110 100 90 80 70 60 50 40 30 20 10 0 -10
f1 (ppm)

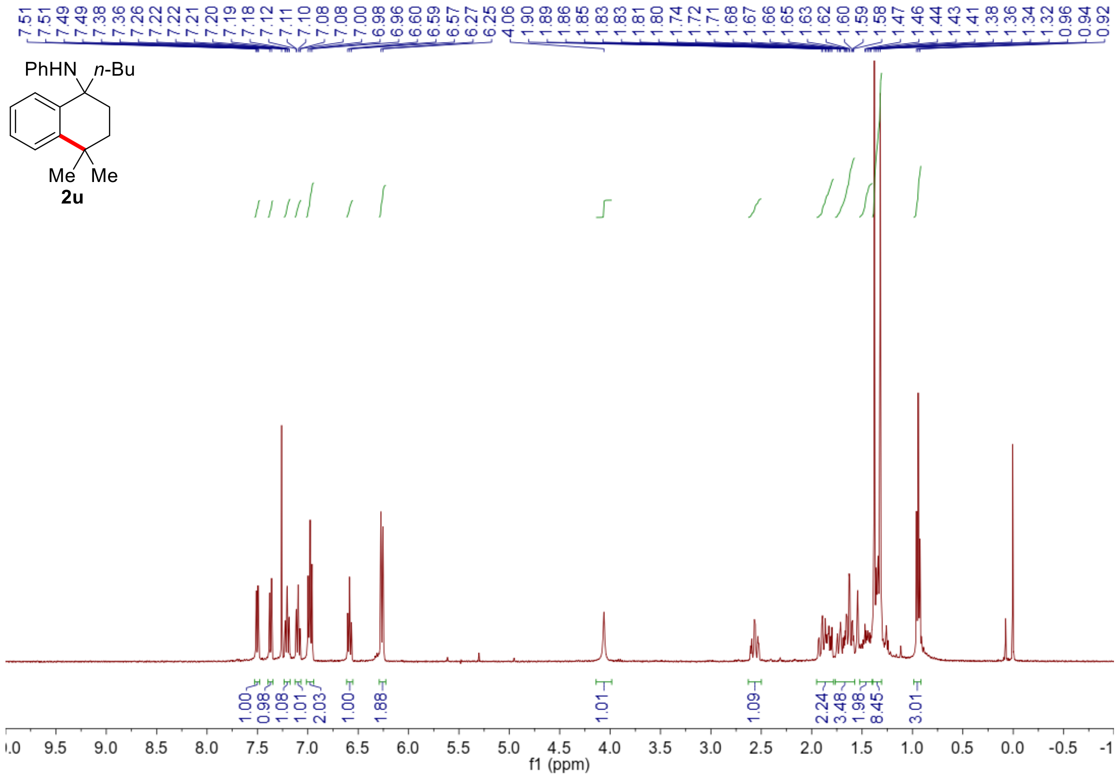
PhHN n-Bu
Me Me
2u
7.51 7.51 7.49 7.38 7.36 7.26 7.22 7.22 7.21 7.20 7.19 7.18 7.12 7.11 7.10 7.08 7.08 7.00 6.98 6.96 6.60 6.59 6.57 6.27 6.25 4.06 1.90 1.89 1.86 1.85 1.83 1.83 1.81 1.80 1.74 1.72 1.71 1.68 1.67 1.66 1.65 1.63 1.62 1.60 1.59 1.58 1.47 1.46 1.44 1.43 1.41 1.38 1.36 1.34 1.32 0.96 0.94 0.92
1.00 0.98 1.08 1.01 2.03 1.00 1.88 1.01 1.09 2.24 3.48 1.98 8.45 3.01
f1 (ppm)

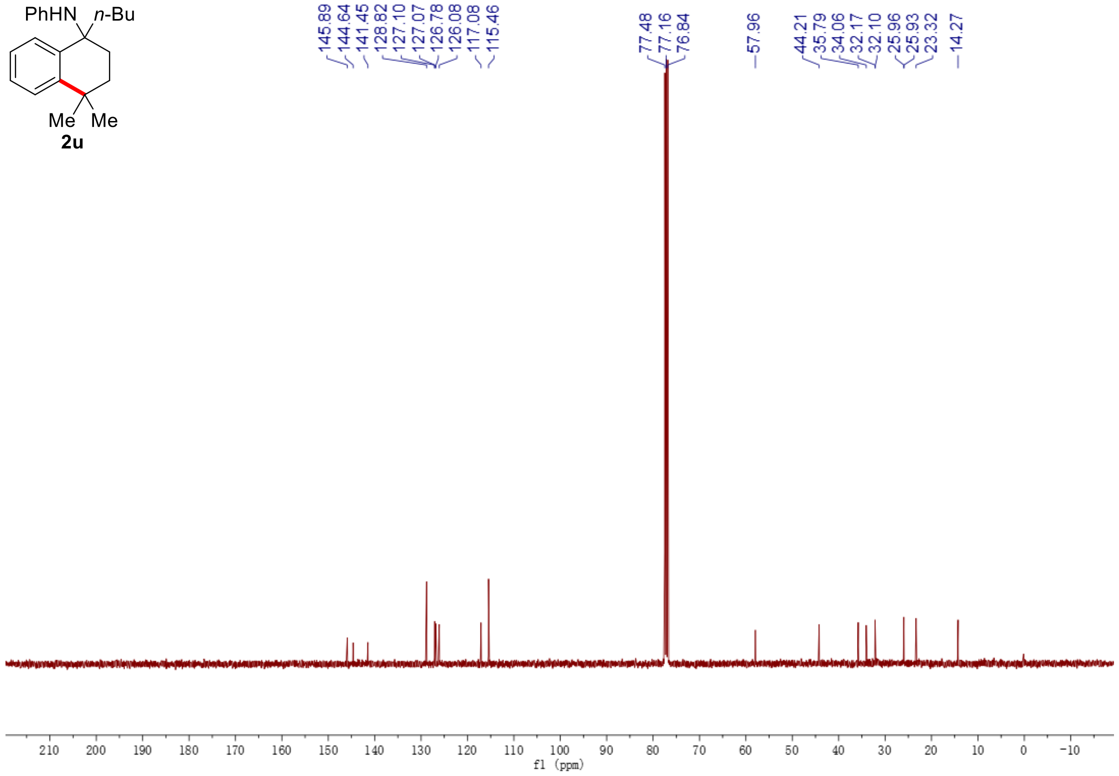
PhHN n-Bu
Me Me
2u
145.89 144.64 141.45 128.82 127.10 127.07 126.78 126.08 117.08 115.46
77.48 77.16 76.84
57.96
44.21 35.79 34.06 32.17 32.10 25.96 25.93 23.32
14.27
f1 (ppm)

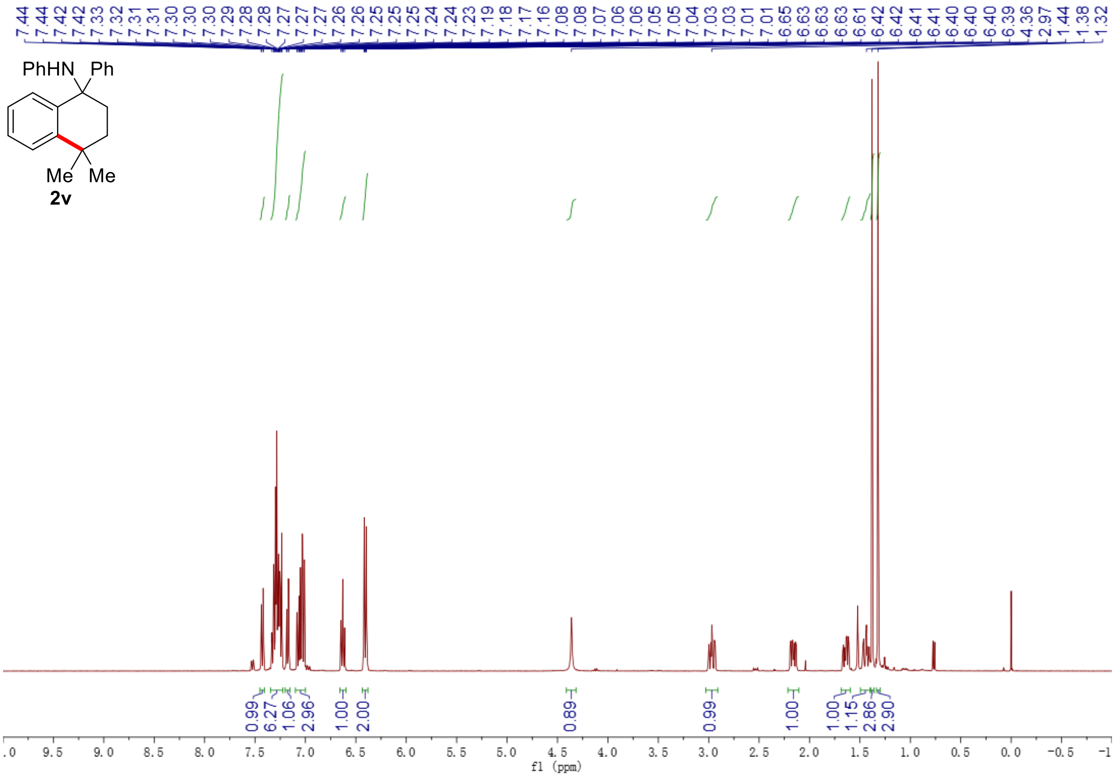
7.44 7.44 7.42 7.42 7.33 7.32 7.31 7.31 7.30 7.30 7.29 7.28 7.28 7.27 7.27 7.27 7.26 7.26 7.25 7.25 7.25 7.24 7.24 7.23 7.19 7.18 7.17 7.16 7.08 7.08 7.07 7.06 7.06 7.05 7.05 7.04 7.03 7.03 7.01 7.01 6.65 6.63 6.63 6.63 6.61 6.42 6.42 6.41 6.41 6.40 6.40 6.40 6.39 4.36 2.97 1.44 1.38 1.32
PhHN Ph
Me Me
2v
0.99 6.27 1.06 2.96 1.00 2.00 0.89 0.99 1.00 1.00 1.15 2.86 2.90
f1 (ppm)

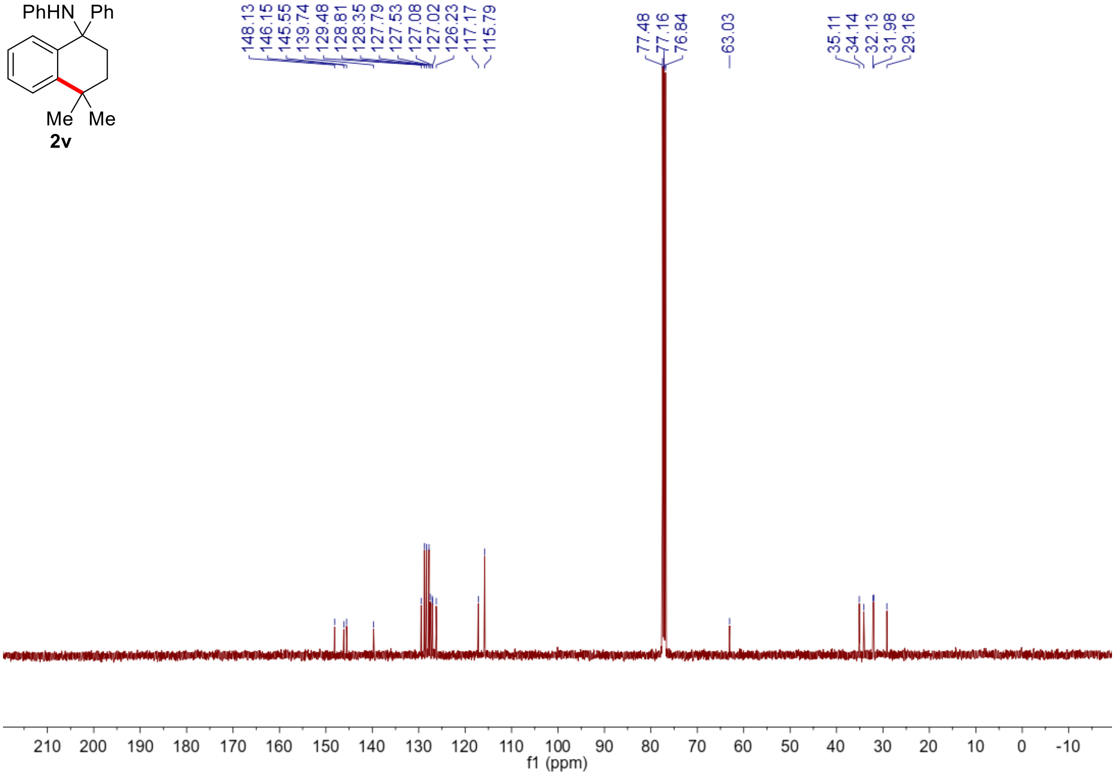
148.13 146.15 145.55 139.74 129.48 128.81 128.35 127.79 127.53 127.08 127.02 126.23 117.17 115.79
77.48 77.16 76.84
63.03
35.11 34.14 32.13 31.98 29.16
PhHN Ph
Me Me
2v
f1 (ppm)

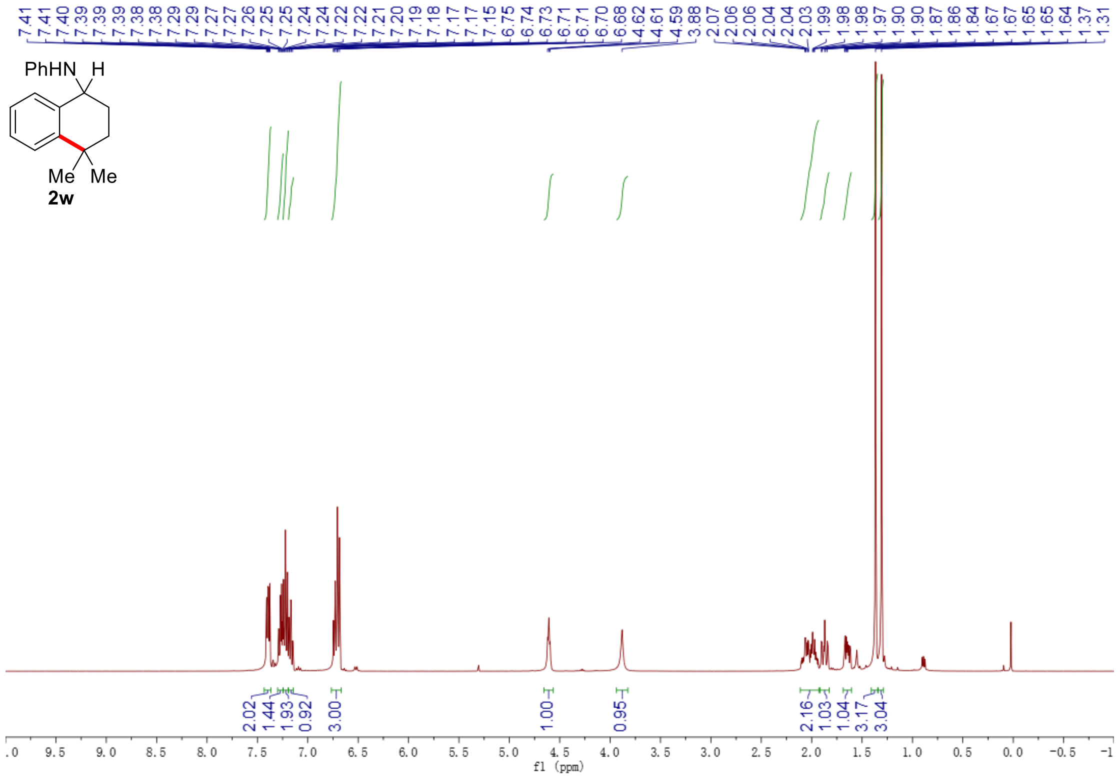

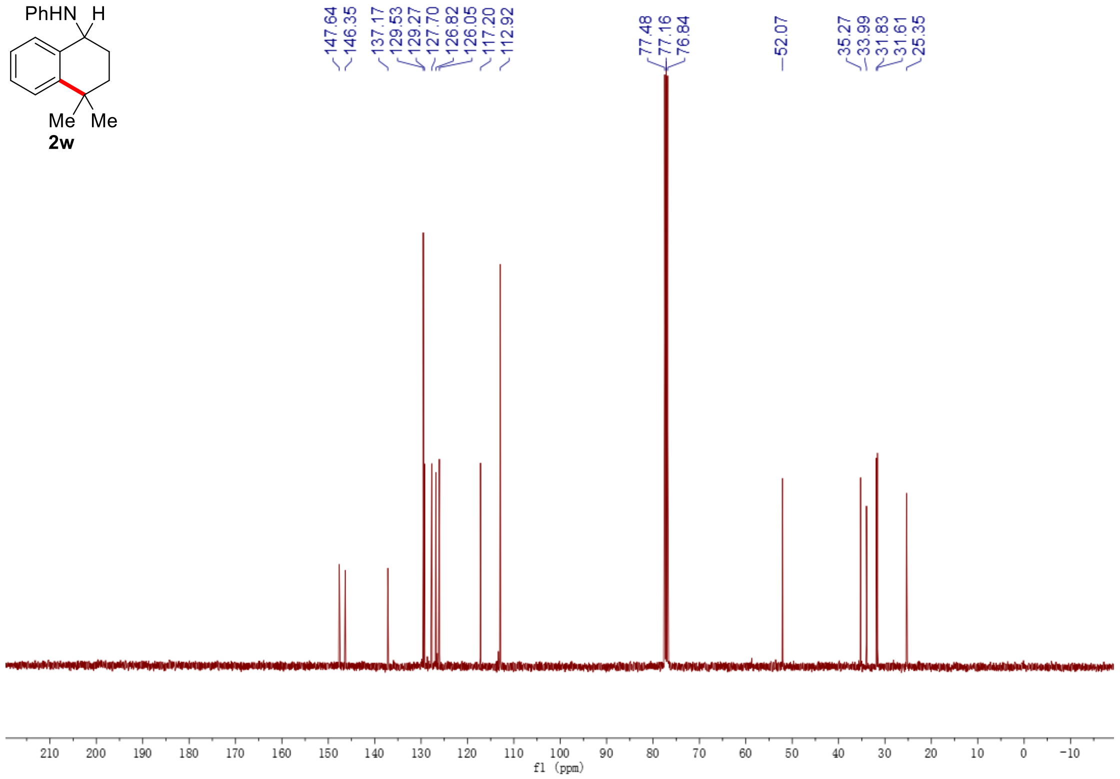

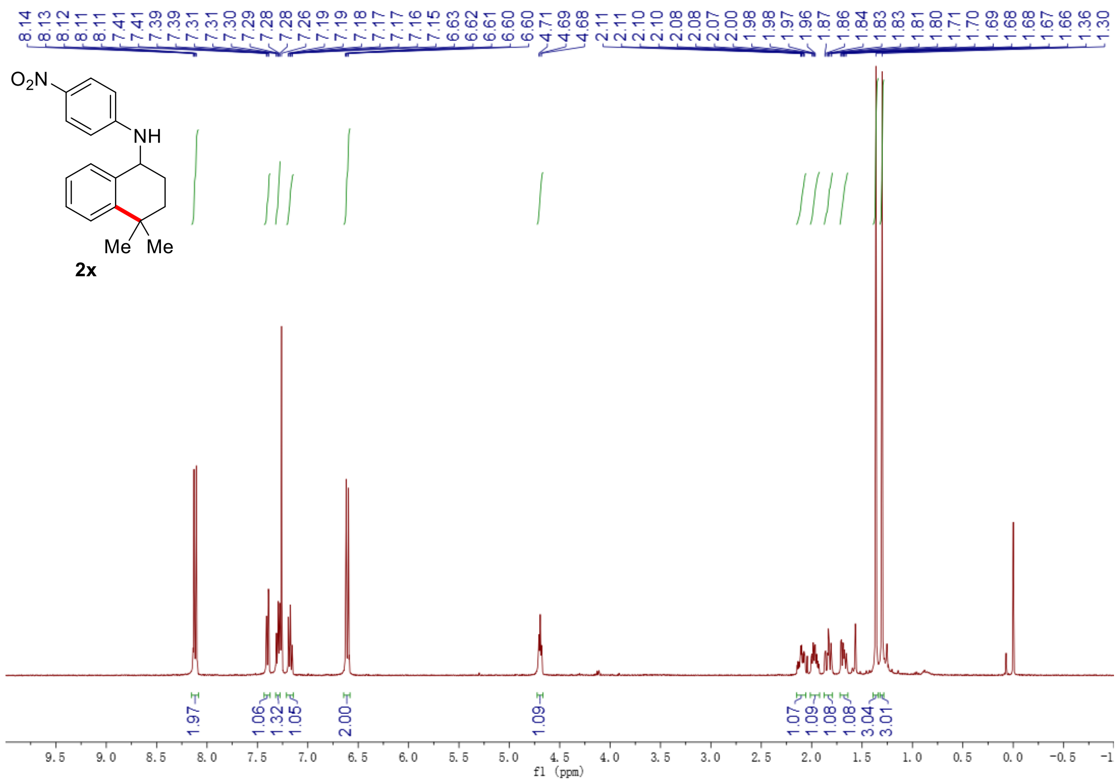
8.14
8.13
8.12
8.11
7.41
7.41
7.39
7.39
7.31
7.31
7.30
7.29
7.28
7.28
7.26
7.19
7.19
7.18
7.17
7.17
7.16
7.15
6.63
6.62
6.61
6.60
6.60
4.71
4.69
4.68
2.11
2.11
2.10
2.10
2.08
2.08
2.07
2.00
1.98
1.98
1.97
1.96
1.87
1.86
1.84
1.83
1.83
1.81
1.80
1.71
1.70
1.69
1.68
1.68
1.67
1.66
1.36
1.30
O2N
NH
Me
Me
2x
1.97
1.06
1.32
1.05
2.00
1.09
1.07
1.09
1.08
1.08
3.04
3.01
9.5 9.0 8.5 8.0 7.5 7.0 6.5 6.0 5.5 5.0 4.5 4.0 3.5 3.0 2.5 2.0 1.5 1.0 0.5 0.0 -0.5 -1
f1 (ppm)

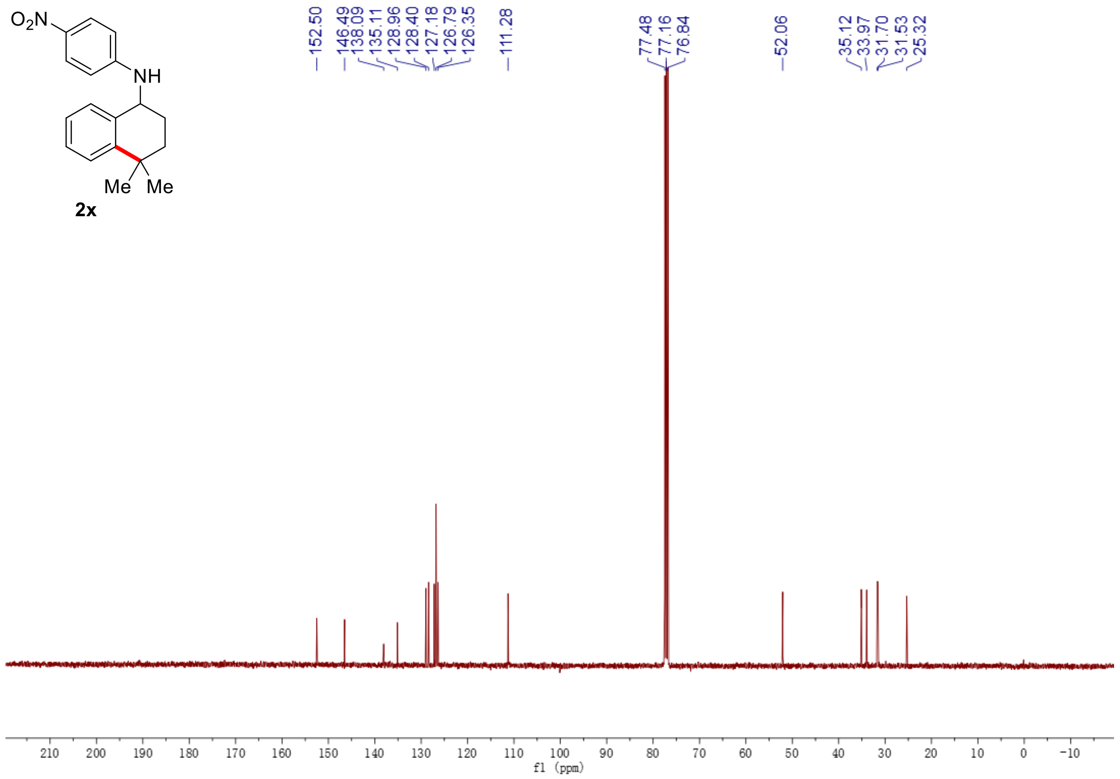
O2N
NH
Me
Me
2x
152.50
146.49
138.09
135.11
128.96
128.40
127.18
126.79
126.35
111.28
77.48
77.16
76.84
52.06
35.12
33.97
31.70
31.53
25.32
210 200 190 180 170 160 150 140 130 120 110 100 90 80 70 60 50 40 30 20 10 0 -10
f1 (ppm)

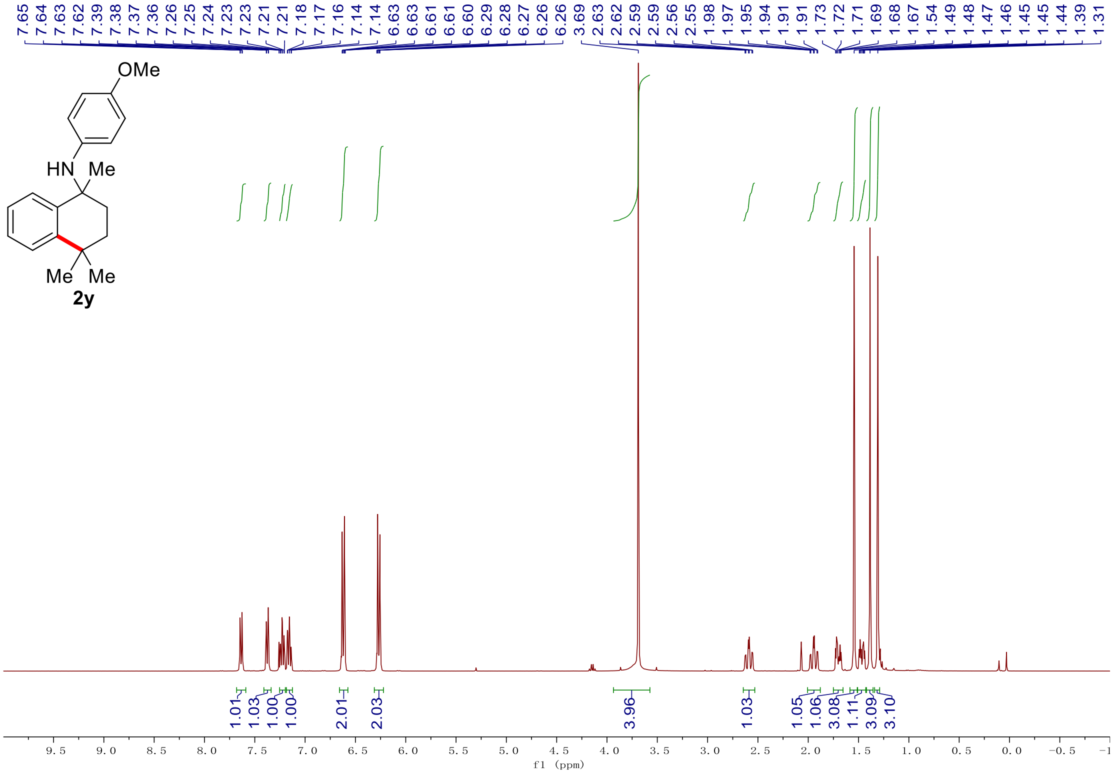
7.65 7.64 7.63 7.62 7.39 7.38 7.37 7.36 7.26 7.25 7.24 7.23 7.23 7.21 7.21 7.18 7.17 7.16 7.14 7.14 6.63 6.61 6.61 6.60 6.29 6.28 6.27 6.26 6.26 3.69 2.63 2.62 2.59 2.59 2.56 2.55 1.98 1.97 1.95 1.94 1.91 1.91 1.73 1.72 1.71 1.69 1.68 1.67 1.54 1.49 1.48 1.47 1.46 1.45 1.44 1.39 1.31
OMe
HN Me
Me Me
2y
1.01 1.03 1.00 1.00 2.01 2.03 3.96 1.03 1.05 1.06 3.08 1.11 3.09 3.10
9.5 9.0 8.5 8.0 7.5 7.0 6.5 6.0 5.5 5.0 4.5 4.0 3.5 3.0 2.5 2.0 1.5 1.0 0.5 0.0 -0.5 -1
f1 (ppm)

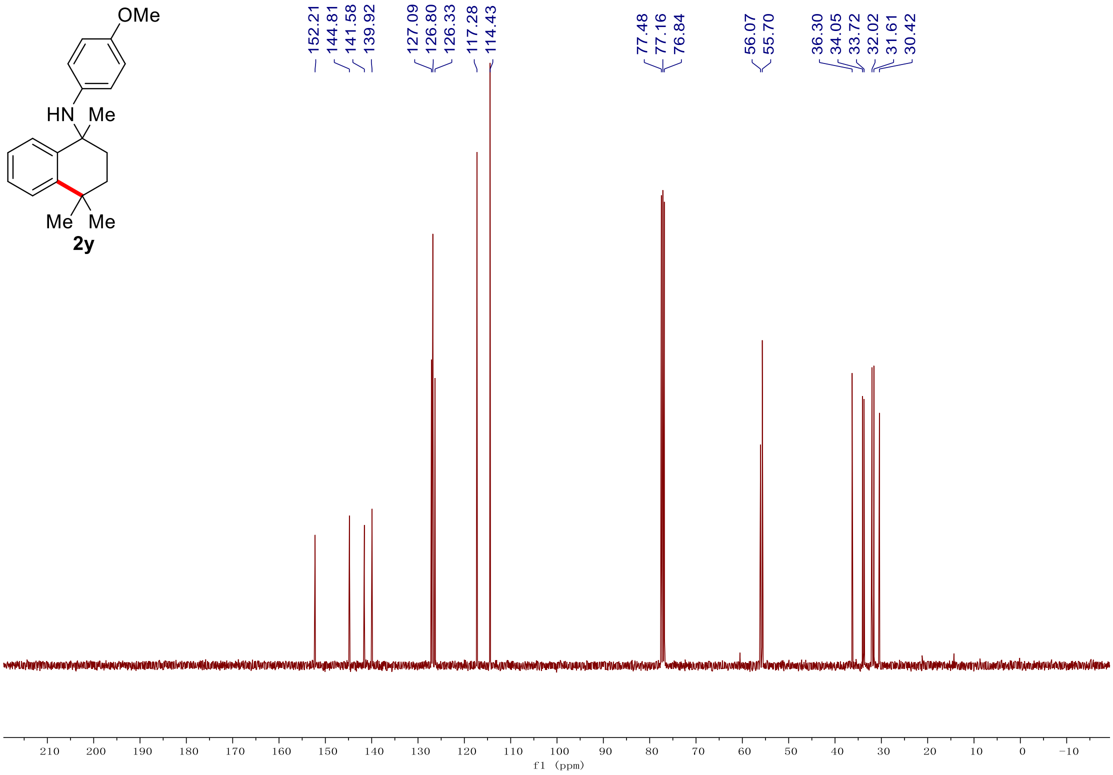
152.21 144.81 141.58 139.92 127.09 126.80 126.33 117.28 114.43 77.48 77.16 76.84 56.07 55.70 36.30 34.05 33.72 32.02 31.61 30.42
OMe
HN Me
Me Me
2y
210 200 190 180 170 160 150 140 130 120 110 100 90 80 70 60 50 40 30 20 10 0 -10
f1 (ppm)

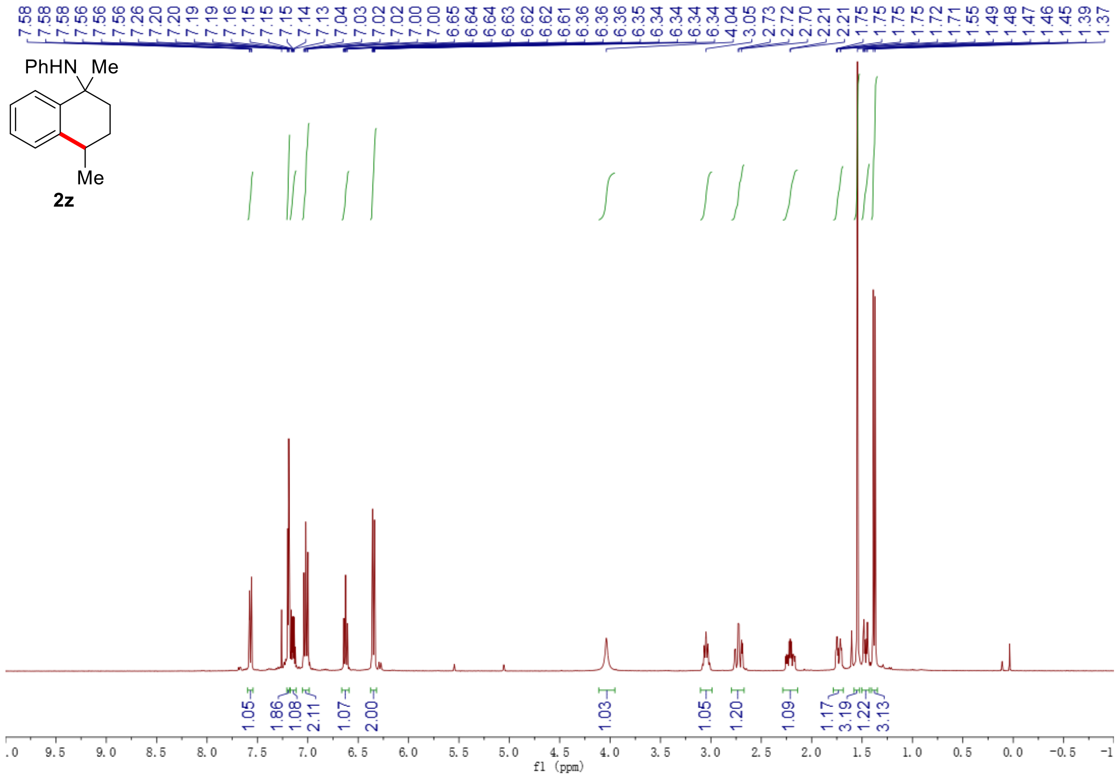
7.58
7.58
7.56
7.56
7.26
7.20
7.20
7.19
7.19
7.16
7.15
7.15
7.15
7.14
7.13
7.04
7.03
7.02
7.02
7.00
7.00
6.65
6.64
6.64
6.63
6.62
6.62
6.61
6.36
6.36
6.35
6.34
6.34
6.34
6.34
4.04
3.05
2.73
2.72
2.70
2.21
2.21
1.75
1.75
1.75
1.75
1.72
1.71
1.55
1.49
1.48
1.47
1.46
1.45
1.39
1.37
PhHN Me
Me
2z
1.05
1.86
1.08
2.11
1.07
2.00
1.03
1.05
1.20
1.09
1.17
3.19
1.22
3.13
f1 (ppm)

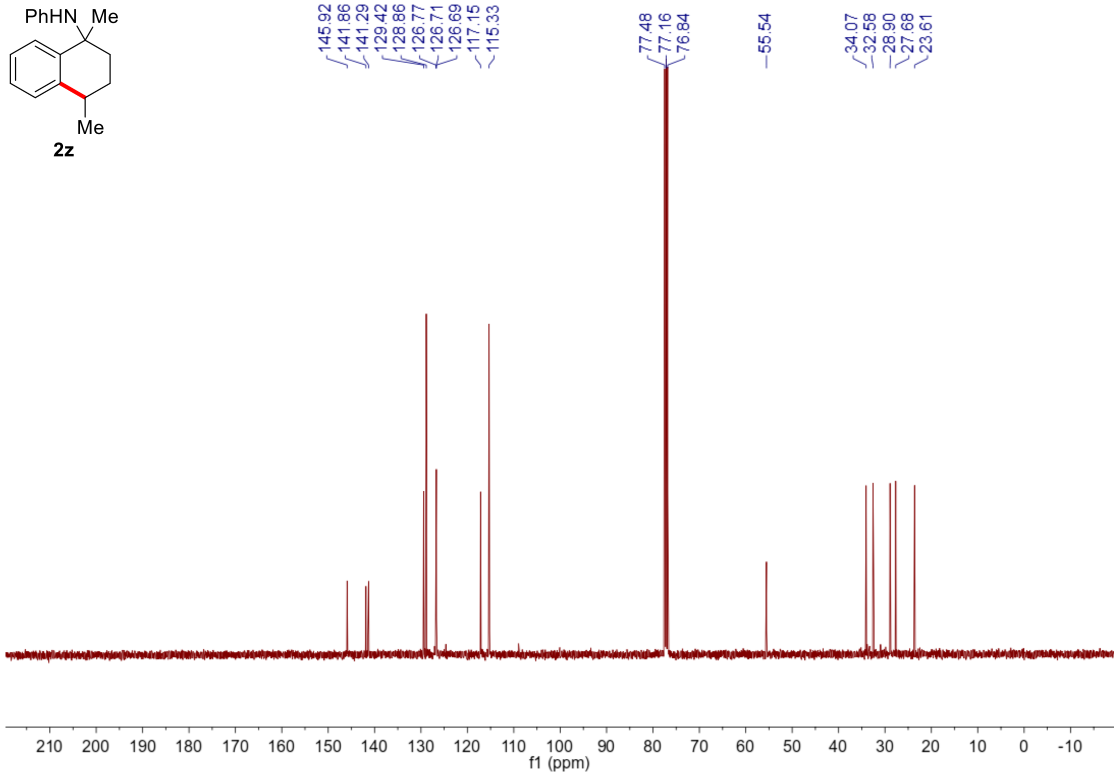
PhHN Me
Me
2z
145.92
141.86
141.29
129.42
128.86
126.77
126.71
126.69
117.15
115.33
77.48
77.16
76.84
55.54
34.07
32.58
28.90
27.68
23.61
f1 (ppm)

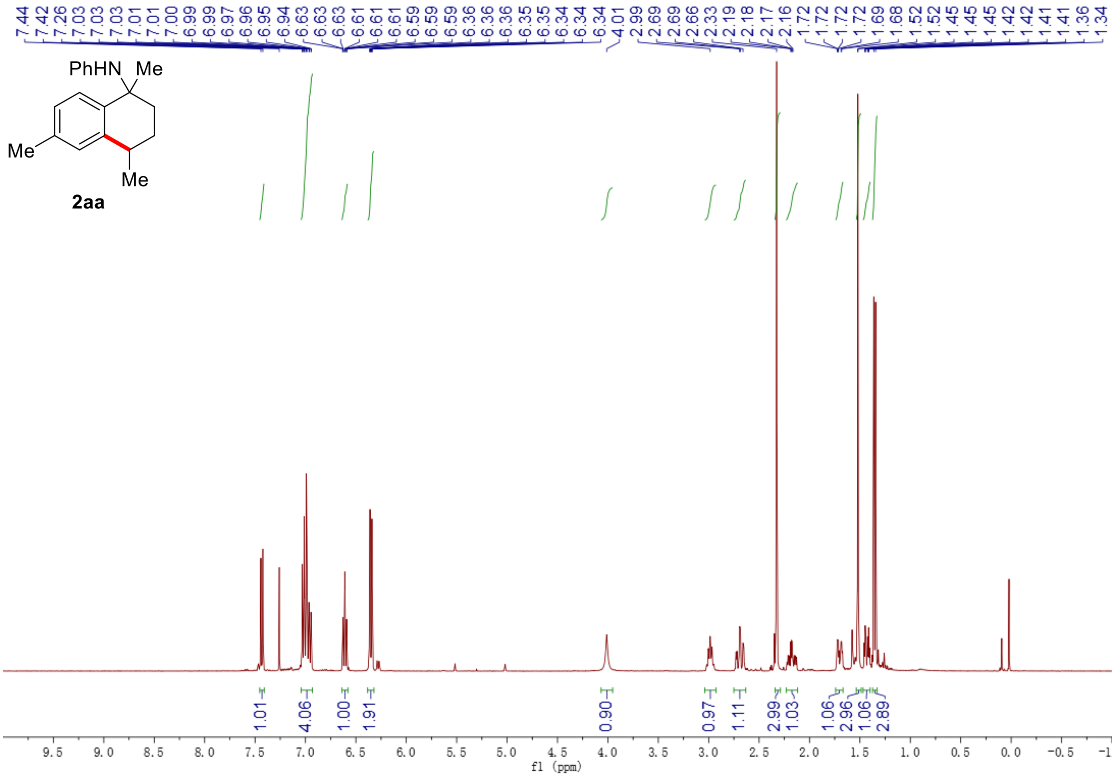
PhHN
Me
Me
Me
2aa
f1 (ppm)

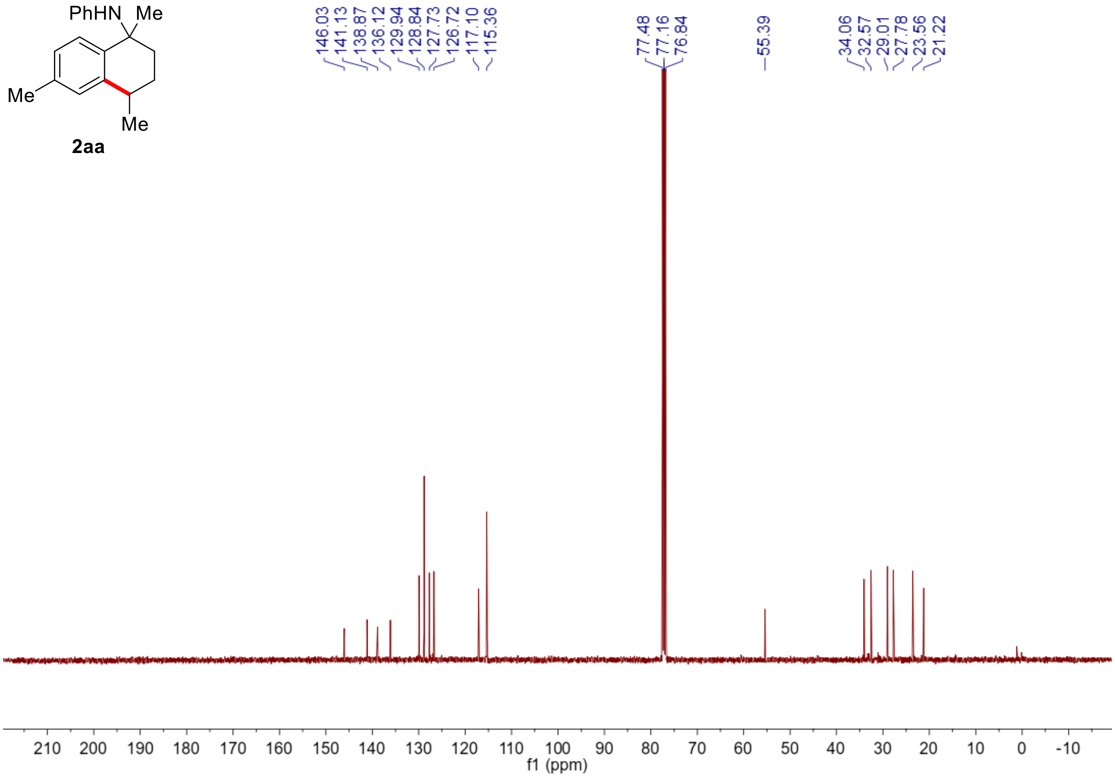
PhHN
Me
Me
Me
2aa
146.03
141.13
138.87
136.12
129.94
128.84
127.73
126.72
117.10
115.36
77.48
77.16
76.84
55.39
34.06
32.57
29.01
27.78
23.56
21.22
f1 (ppm)

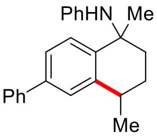
PhHN
Me
Ph
Me

2ab

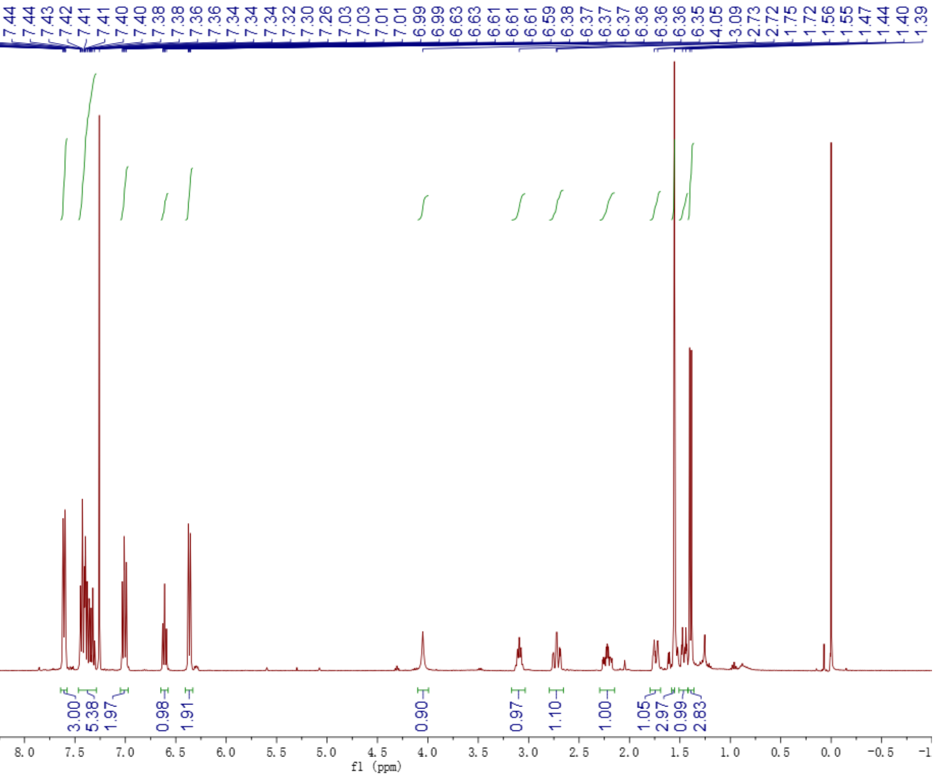
7.62
7.62
7.61
7.60
7.60
7.59
7.45
7.45
7.44
7.44
7.43
7.42
7.41
7.41
7.40
7.38
7.38
7.36
7.36
7.34
7.34
7.34
7.32
7.30
7.26
7.03
7.03
7.01
7.01
6.99
6.99
6.63
6.63
6.61
6.61
6.61
6.59
6.38
6.37
6.37
6.37
6.36
6.36
6.36
6.35
4.05
3.09
2.73
2.72
1.75
1.72
1.56
1.55
1.47
1.44
1.40
1.39
3.00
5.38
1.97
0.98
1.91
0.90
0.97
1.10
1.00
1.05
2.97
0.99
2.83
9.5 9.0 8.5 8.0 7.5 7.0 6.5 6.0 5.5 5.0 4.5 4.0 3.5 3.0 2.5 2.0 1.5 1.0 0.5 0.0 -0.5 -1
f1 (ppm)

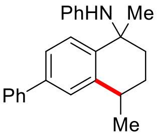
PhHN
Me
Ph
Me

2ab

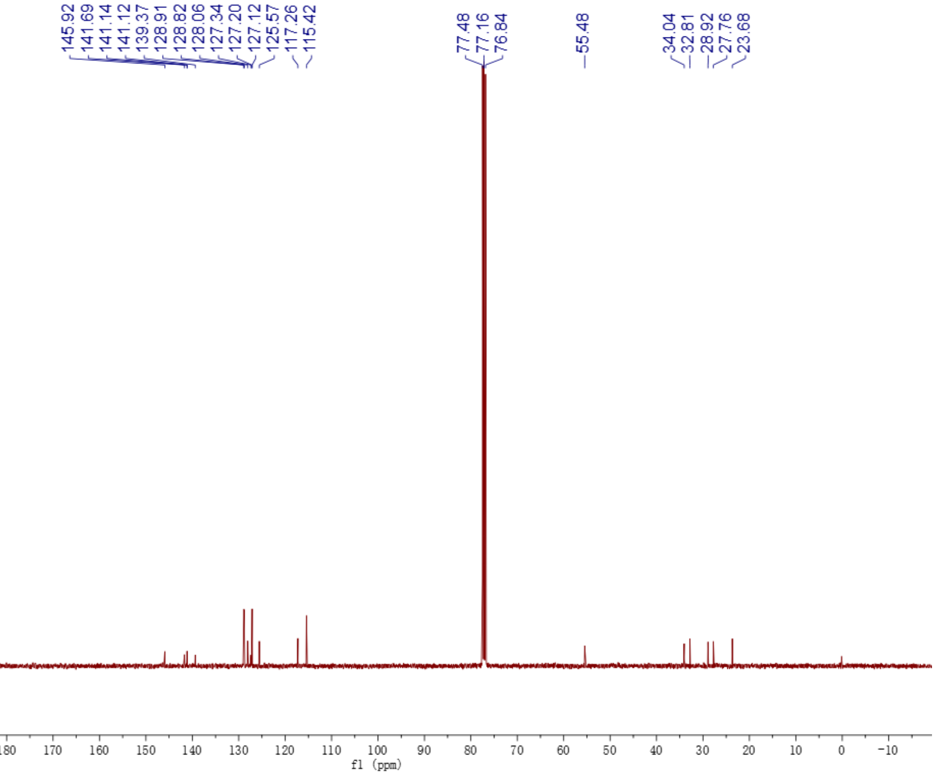
145.92
141.69
141.14
141.12
139.37
128.91
128.82
128.06
127.34
127.20
127.12
125.57
117.26
115.42
77.48
77.16
76.84
55.48
34.04
32.81
28.92
27.76
23.68
210 200 190 180 170 160 150 140 130 120 110 100 90 80 70 60 50 40 30 20 10 0 -10
f1 (ppm)

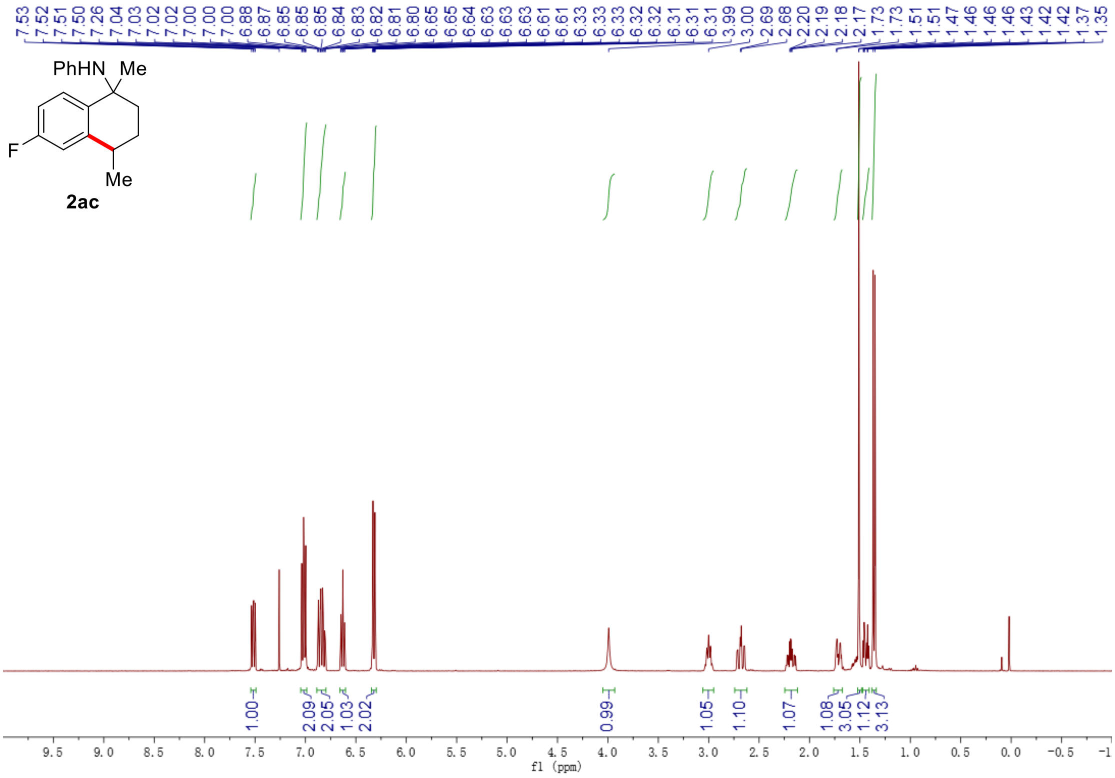
7.53 7.52 7.51 7.50 7.26 7.04 7.03 7.02 7.02 7.00 7.00 7.00 6.88 6.87 6.85 6.85 6.85 6.84 6.83 6.82 6.81 6.80 6.65 6.65 6.64 6.63 6.63 6.63 6.61 6.61 6.33 6.33 6.32 6.32 6.31 6.31 6.31 3.99 3.00 2.69 2.68 2.20 2.19 2.18 2.17 1.73 1.73 1.51 1.51 1.47 1.46 1.46 1.43 1.42 1.37 1.35
PhHN Me
F
Me
2ac
1.00 2.09 2.05 1.03 2.02 0.99 1.05 1.10 1.07 1.08 3.05 1.12 3.13
f1 (ppm)

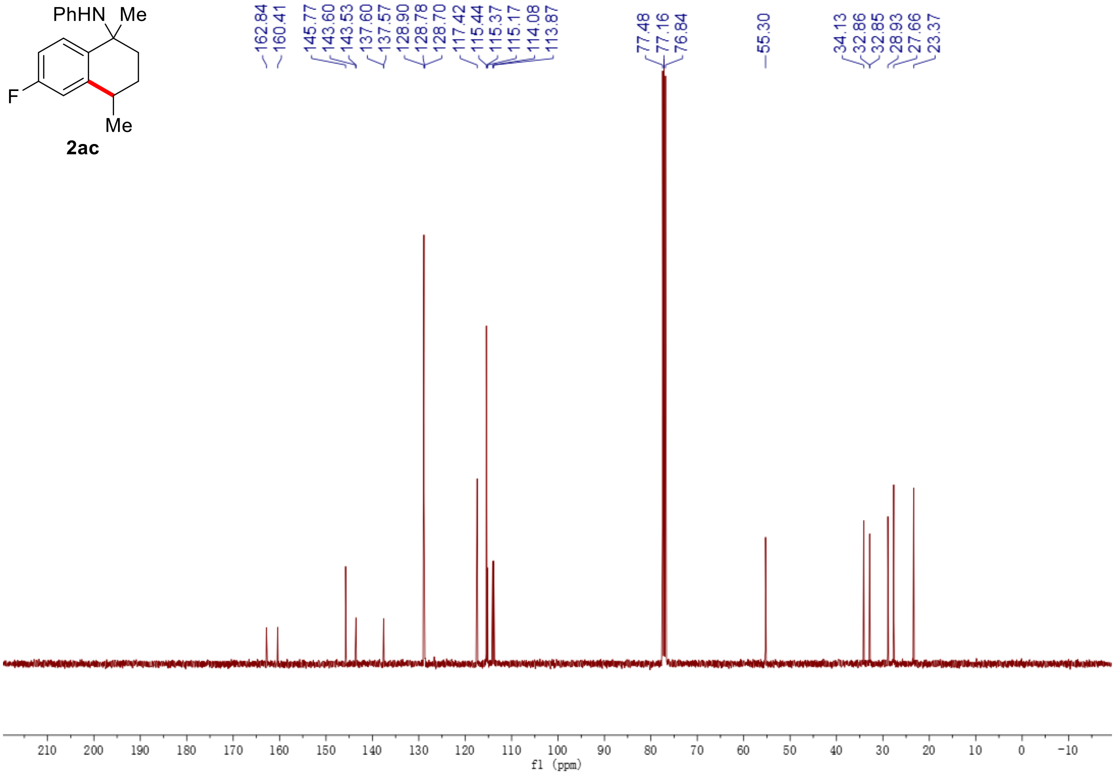
162.84 160.41 145.77 143.60 143.53 137.60 137.57 128.90 128.78 128.70 117.42 115.44 115.37 115.17 114.08 113.87 77.48 77.16 76.84 55.30 34.13 32.86 32.85 28.93 27.66 23.37
PhHN Me
F
Me
2ac
f1 (ppm)

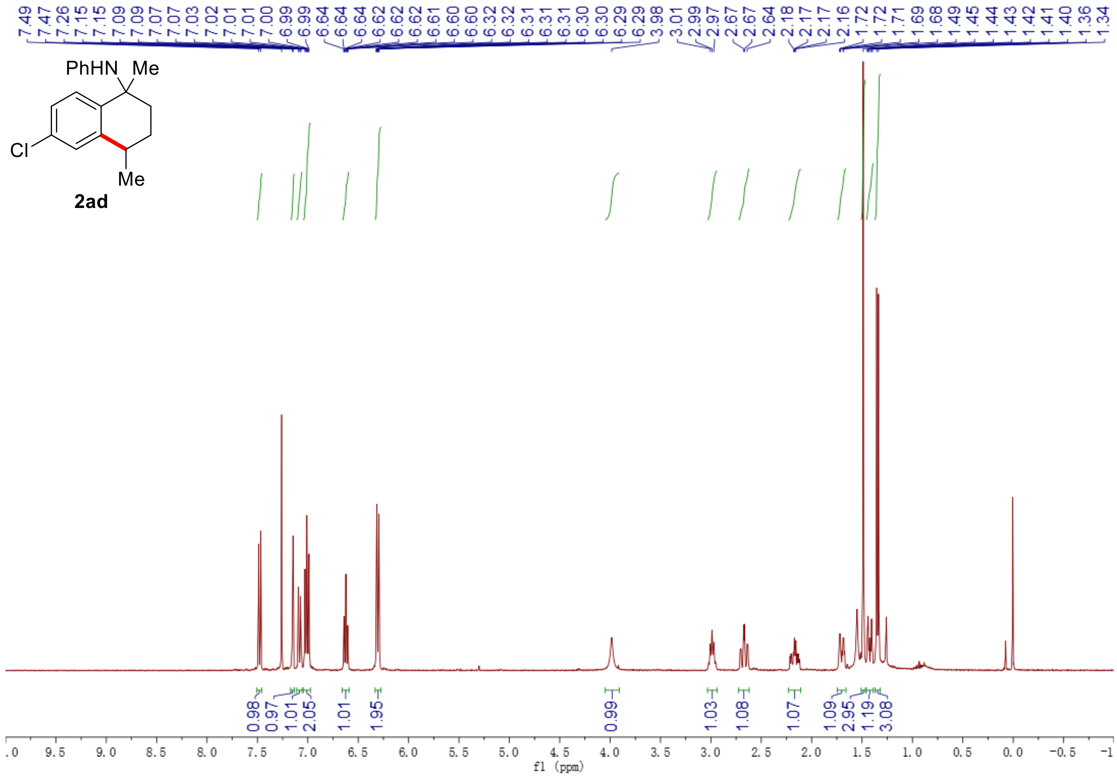
PhHN Me
Cl
Me
2ad
f1 (ppm)

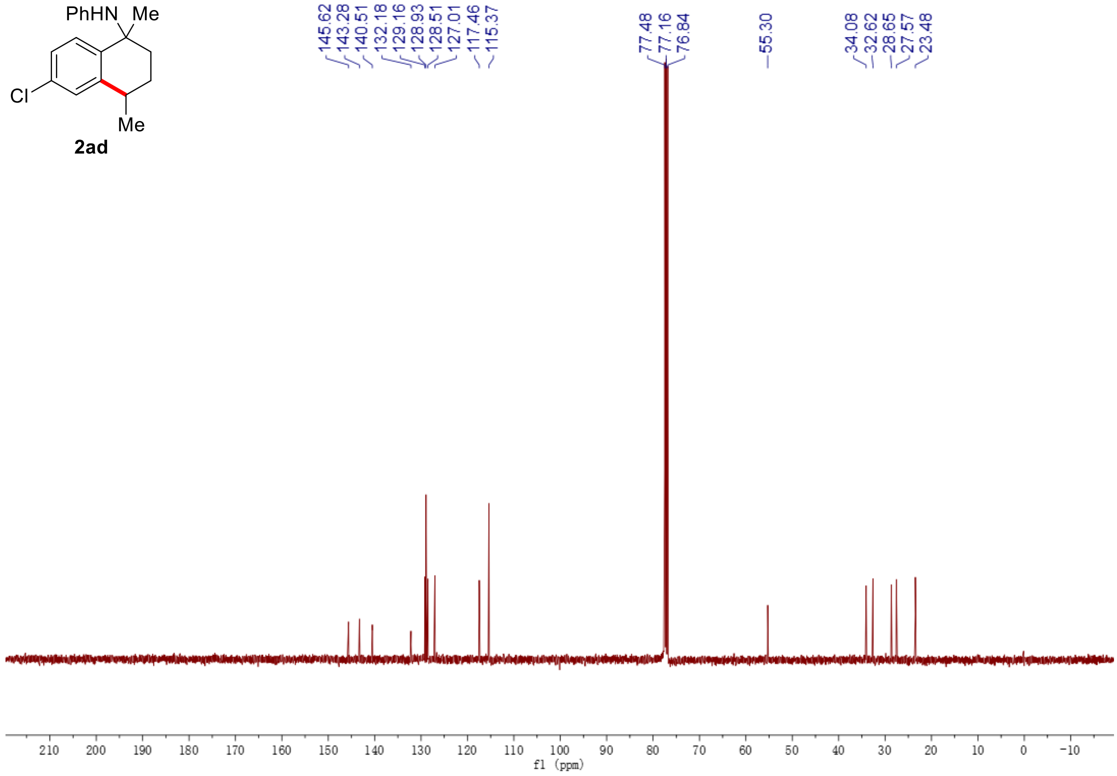
PhHN Me
Cl
Me
2ad
145.62
143.28
140.51
132.18
129.16
128.93
128.51
127.01
117.46
115.37
77.48
77.16
76.84
55.30
34.08
32.62
28.65
27.57
23.48
f1 (ppm)

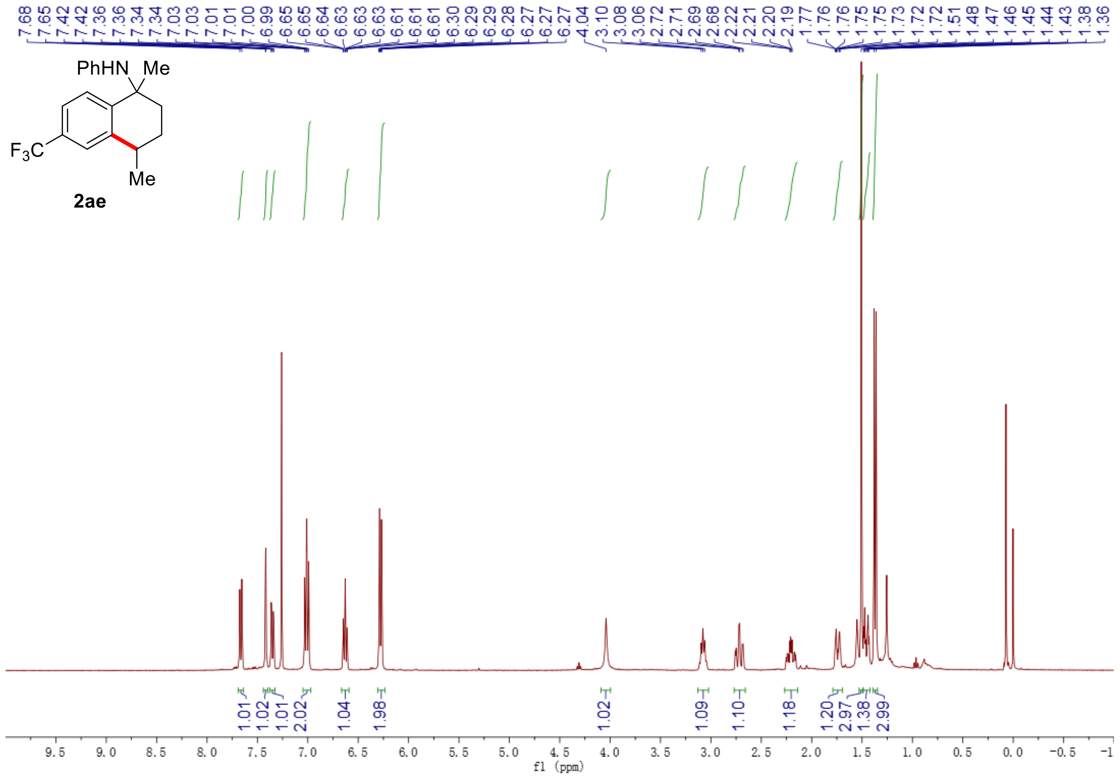

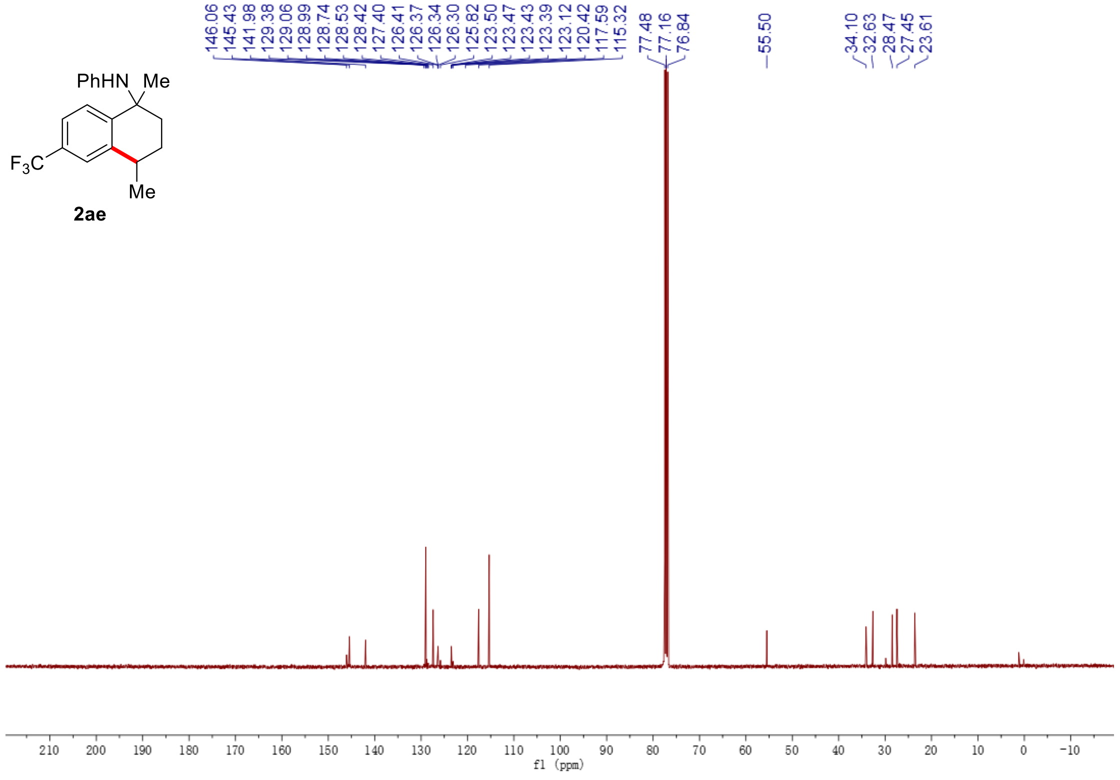

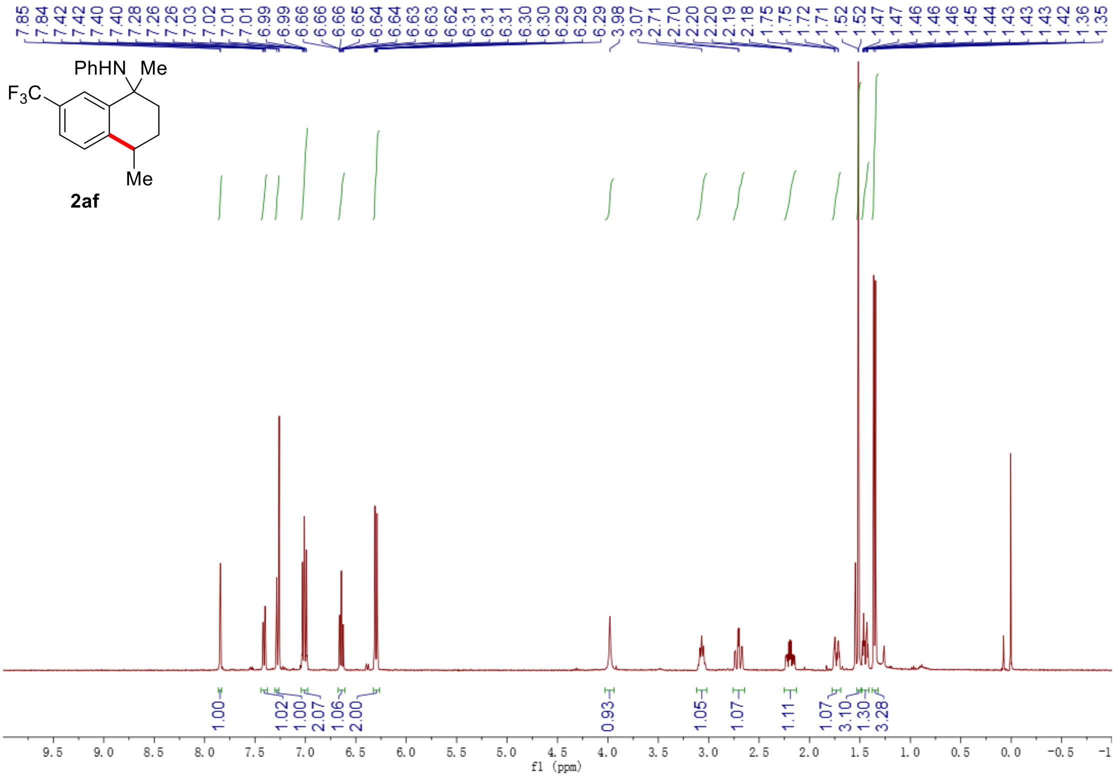
7.85 7.84 7.42 7.42 7.40 7.40 7.28 7.26 7.26 7.03 7.02 7.01 7.01 6.99 6.99 6.66 6.66 6.65 6.64 6.64 6.63 6.63 6.62 6.31 6.31 6.31 6.30 6.29 6.29 6.29 3.98 3.07 2.71 2.70 2.20 2.20 2.19 2.18 1.75 1.75 1.72 1.71 1.52 1.52 1.47 1.46 1.46 1.45 1.44 1.43 1.43 1.43 1.42 1.36 1.35
PhHN Me
F3C
Me
2af
1.00 1.02 1.00 2.07 1.06 2.00 0.93 1.05 1.07 1.11 1.07 3.10 1.30 3.28
9.5 9.0 8.5 8.0 7.5 7.0 6.5 6.0 5.5 5.0 4.5 4.0 3.5 3.0 2.5 2.0 1.5 1.0 0.5 0.0 -0.5 -1
f1 (ppm)

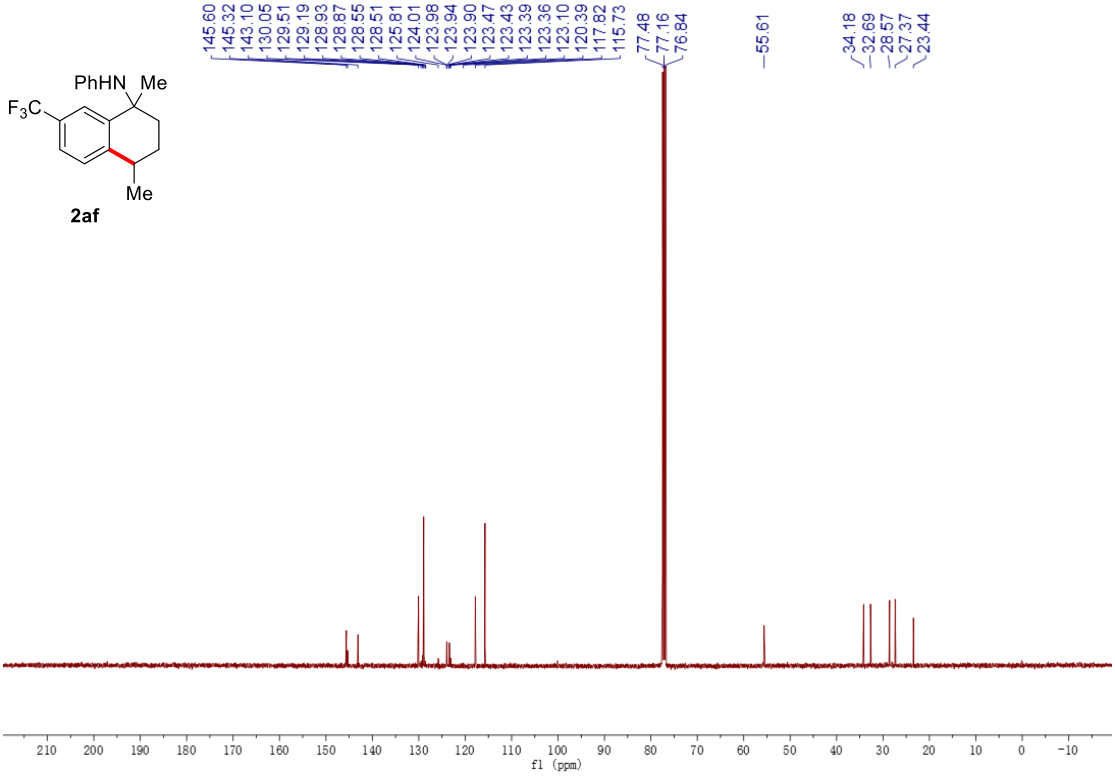
145.60 145.32 143.10 130.05 129.51 129.19 128.93 128.87 128.55 128.51 125.81 124.01 123.98 123.94 123.90 123.47 123.43 123.39 123.36 123.10 120.39 117.82 115.73 77.48 77.16 76.84 55.61 34.18 32.69 28.57 27.37 23.44
PhHN Me
F3C
Me
2af
210 200 190 180 170 160 150 140 130 120 110 100 90 80 70 60 50 40 30 20 10 0 -10
f1 (ppm)

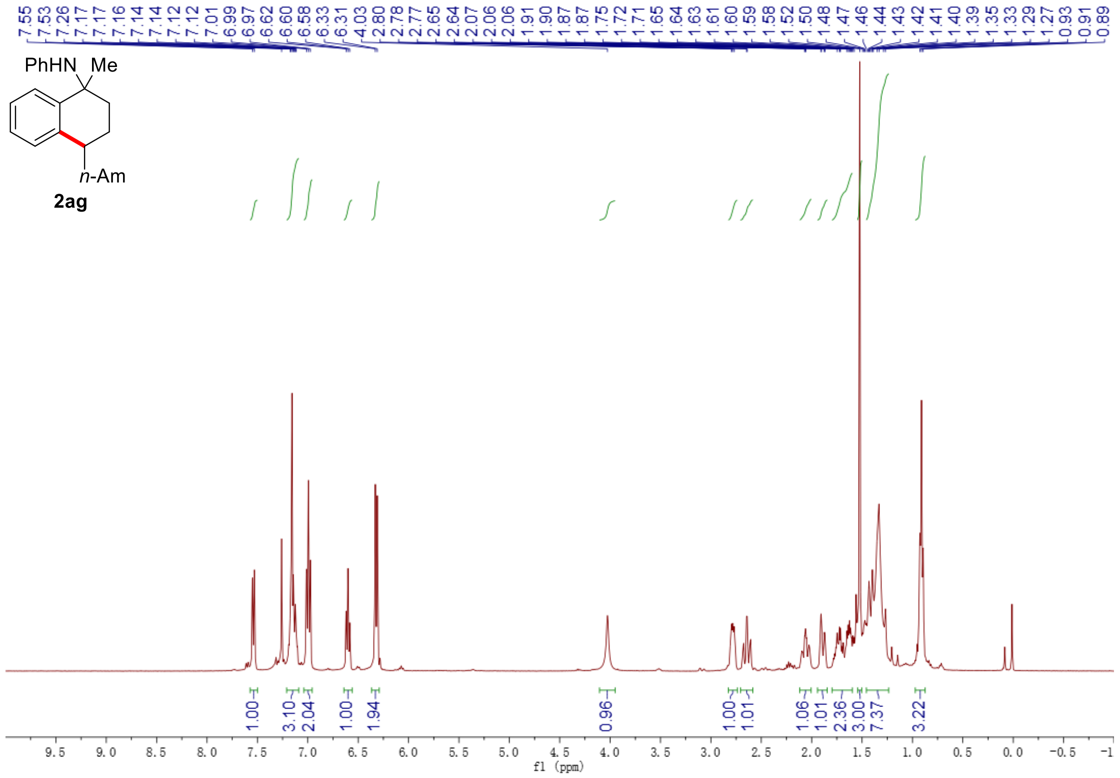
7.55 7.53 7.26 7.17 7.17 7.16 7.14 7.14 7.12 7.12 7.01 6.99 6.97 6.62 6.60 6.58 6.33 6.31 4.03 2.80 2.78 2.77 2.65 2.64 2.07 2.06 2.06 1.91 1.90 1.87 1.87 1.75 1.72 1.71 1.65 1.64 1.63 1.61 1.60 1.59 1.58 1.52 1.50 1.48 1.47 1.46 1.44 1.43 1.42 1.41 1.40 1.39 1.35 1.33 1.29 1.27 0.93 0.91 0.89
PhHN
Me
n-Am
2ag
1.00 3.10 2.04 1.00 1.94 0.96 1.00 1.01 1.06 1.01 2.36 3.00 7.37 3.22
f1 (ppm)

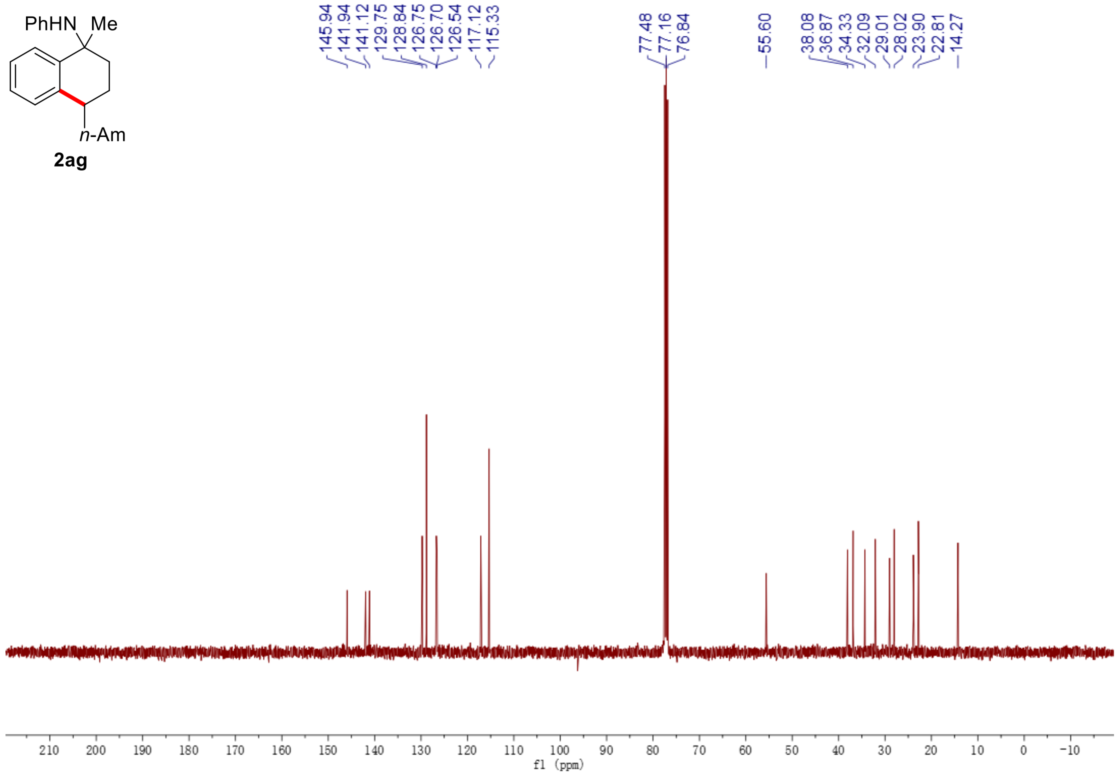
145.94 141.94 141.12 129.75 128.84 126.75 126.70 126.54 117.12 115.33 77.48 77.16 76.84 55.60 38.08 36.87 34.33 32.09 29.01 28.02 23.90 22.81 14.27
PhHN
Me
n-Am
2ag
f1 (ppm)

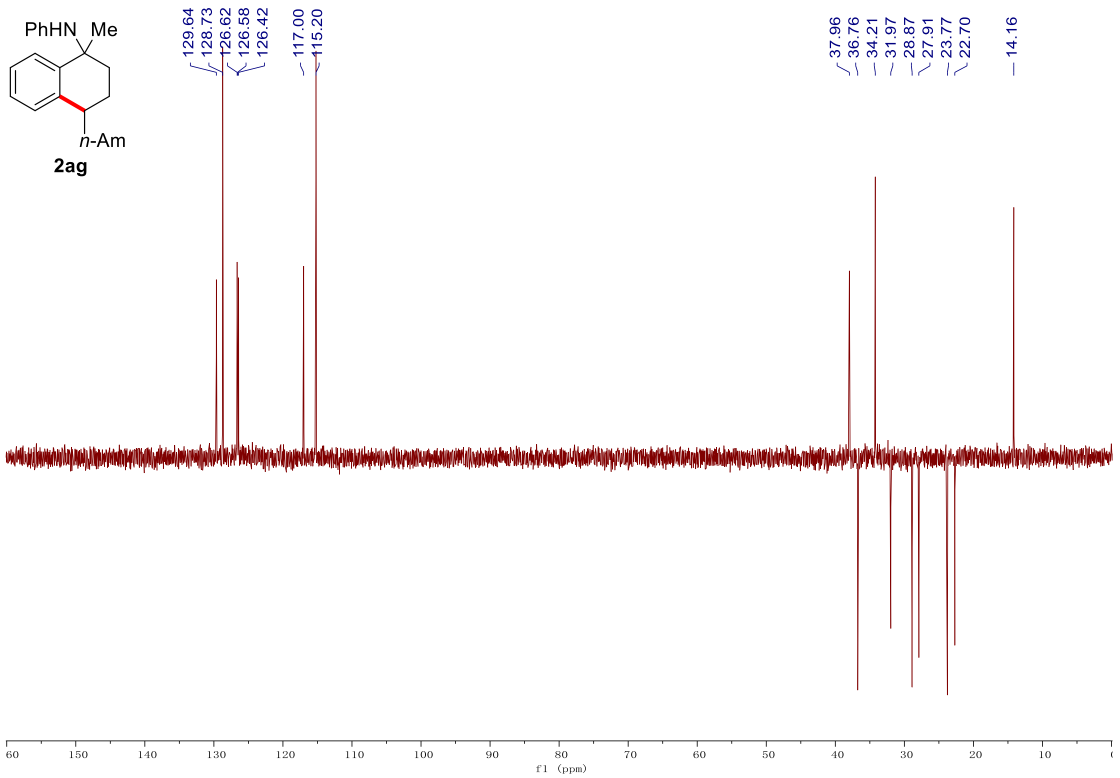
PhHN Me
n-Am
2ag
f1 (ppm)

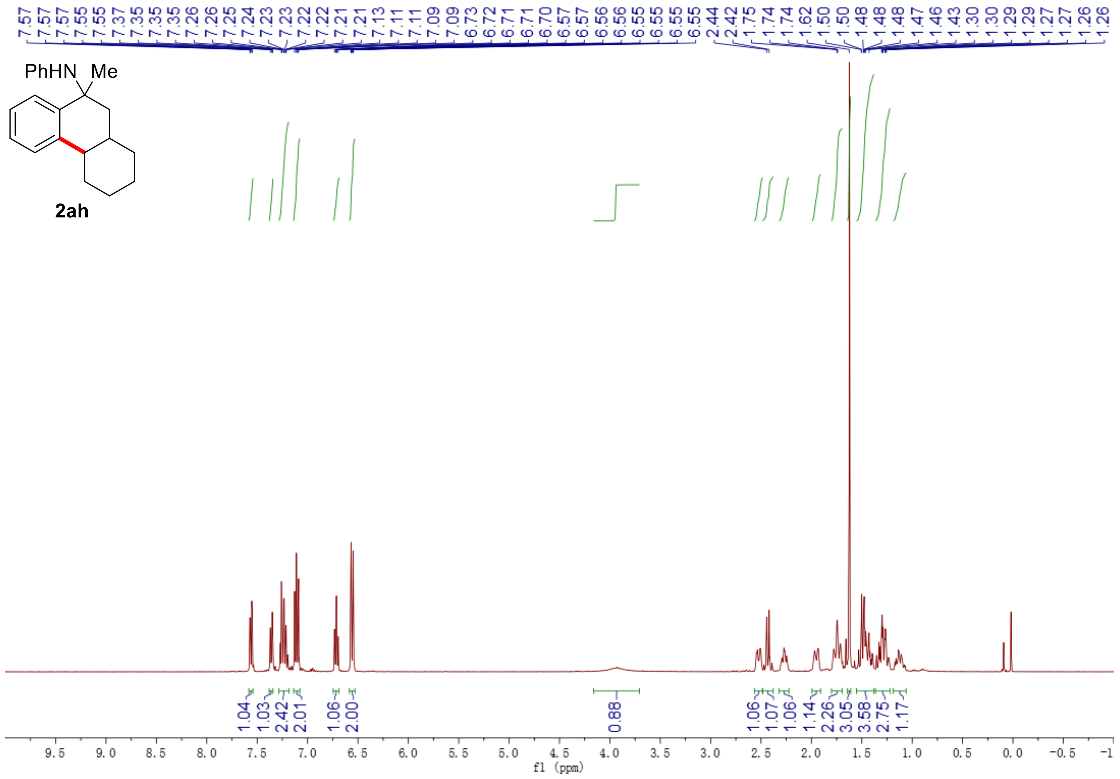
PhHN Me
2ah
f1 (ppm)

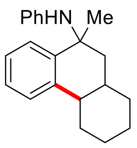
PhHN Me

2ah

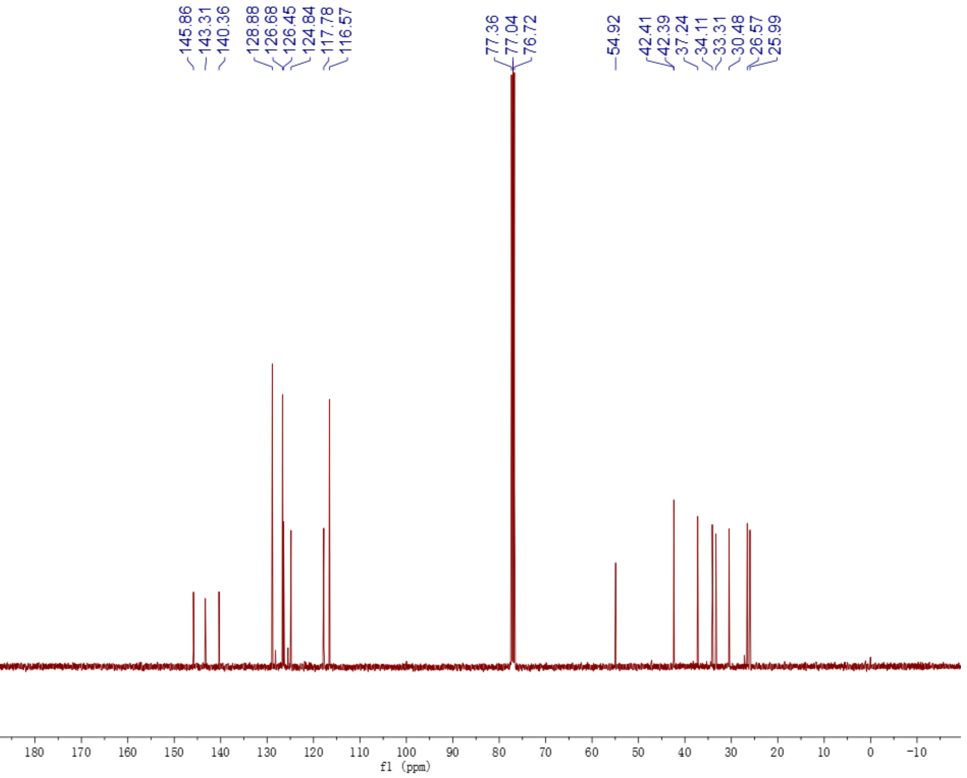
145.86
143.31
140.36
128.88
126.68
126.45
124.84
117.78
116.57
77.36
77.04
76.72
54.92
42.41
42.39
37.24
34.11
33.31
30.48
26.57
25.99
210 200 190 180 170 160 150 140 130 120 110 100 90 80 70 60 50 40 30 20 10 0 -10
f1 (ppm)

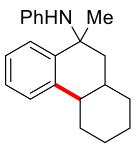
PhHN Me

2ah

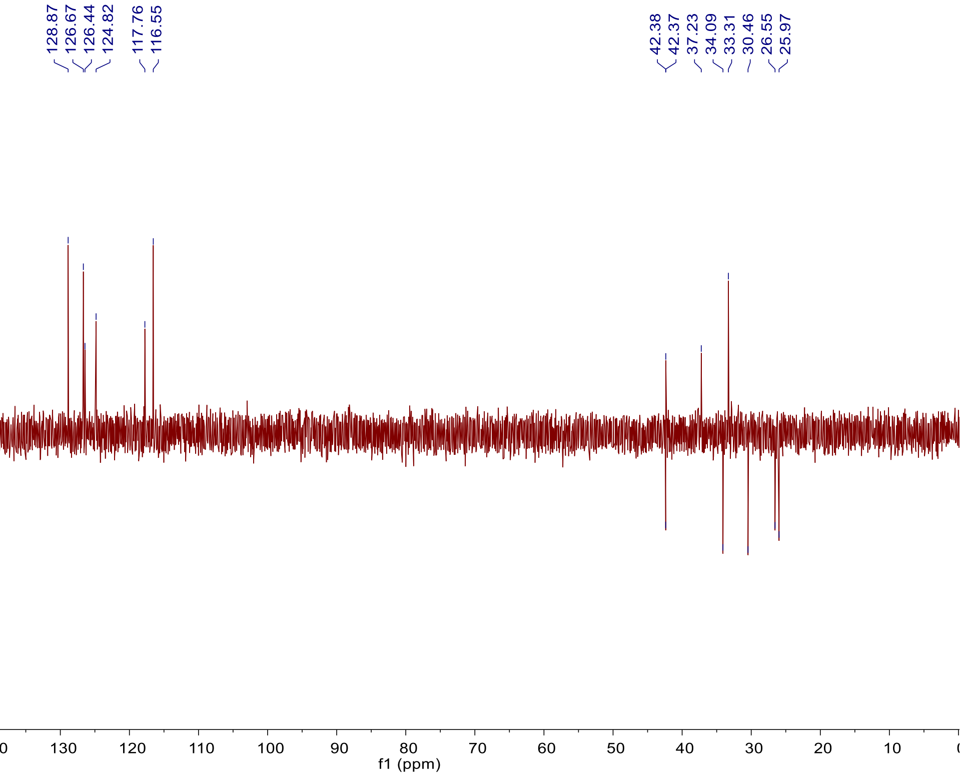
128.87
126.67
126.44
124.82
117.76
116.55
42.38
42.37
37.23
34.09
33.31
30.46
26.55
25.97
150 140 130 120 110 100 90 80 70 60 50 40 30 20 10
f1 (ppm)

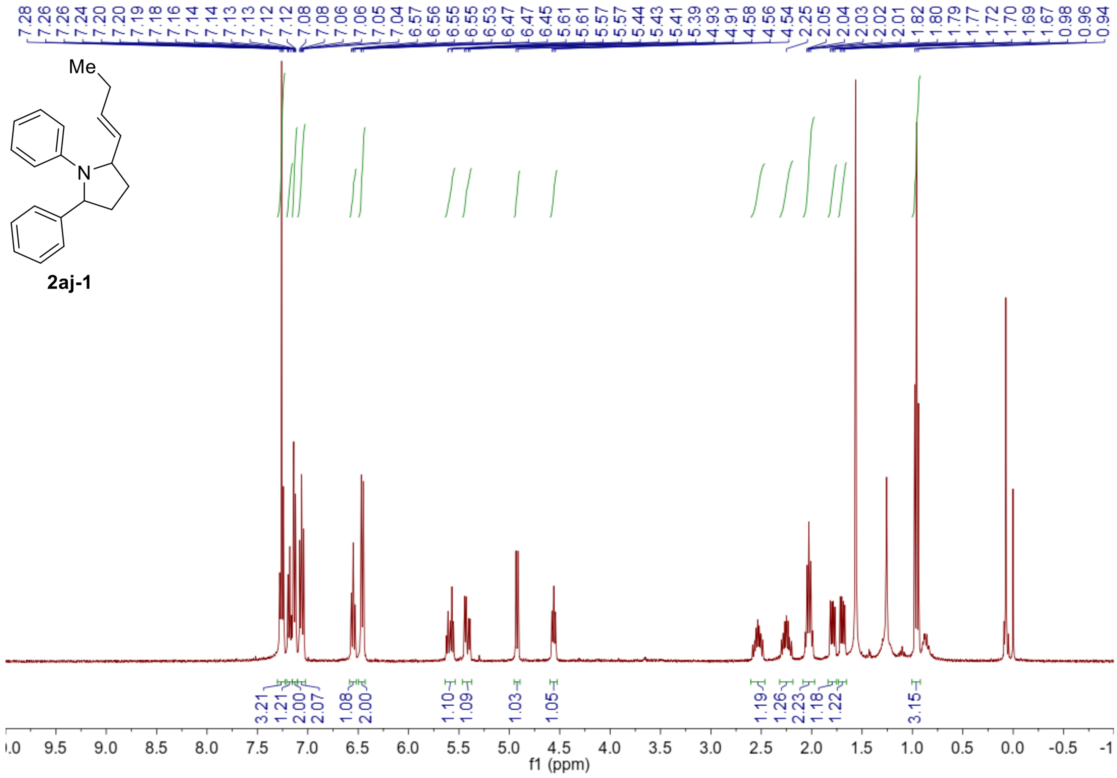
Me
N
2aj-1
7.28 7.26 7.26 7.24 7.20 7.20 7.19 7.18 7.16 7.14 7.14 7.13 7.13 7.12 7.12 7.08 7.08 7.06 7.06 7.05 7.04 6.57 6.56 6.55 6.55 6.53 6.47 6.47 6.45 5.61 5.61 5.57 5.57 5.44 5.43 5.41 5.39 4.93 4.91 4.58 4.56 4.54 2.25 2.05 2.04 2.03 2.02 2.01 1.82 1.80 1.79 1.77 1.72 1.70 1.69 1.67 0.98 0.96 0.94
3.21 1.21 2.00 2.07 1.08 2.00 1.10 1.09 1.03 1.05 1.19 1.26 2.23 1.18 1.22 3.15
f1 (ppm)

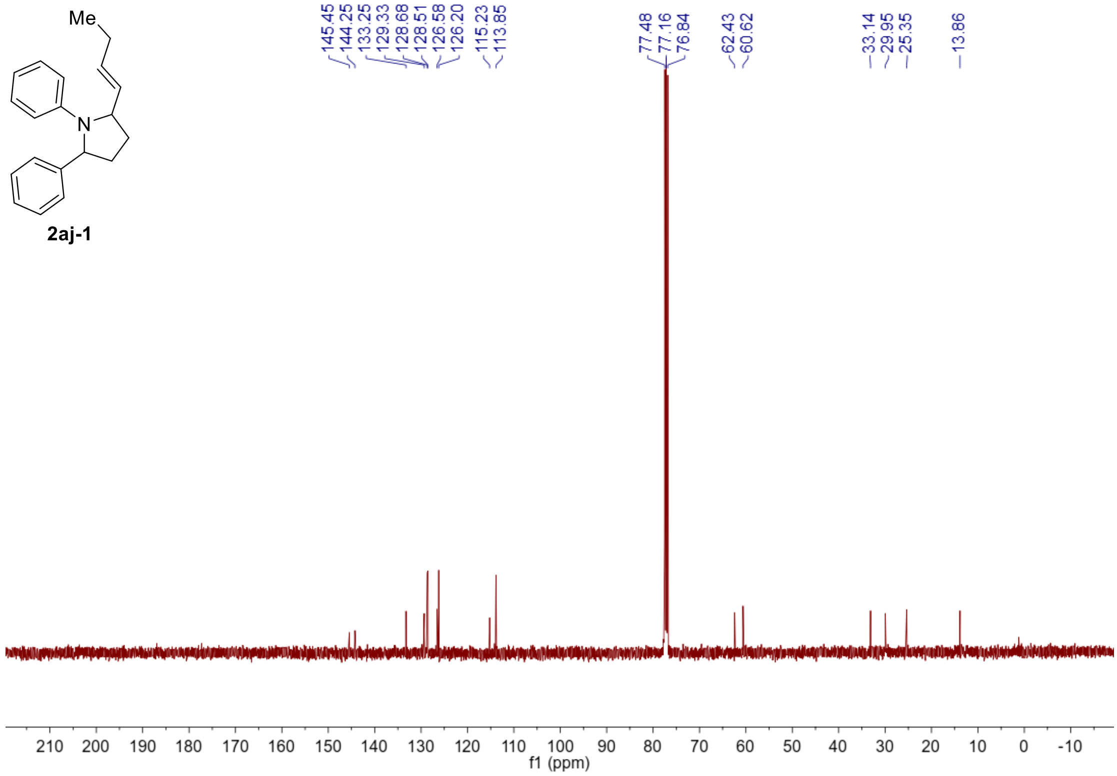
Me
N
2aj-1
145.45 144.25 133.25 129.33 128.68 128.51 126.58 126.20 115.23 113.85
77.48 77.16 76.84
62.43 60.62
33.14 29.95 25.35
13.86
f1 (ppm)

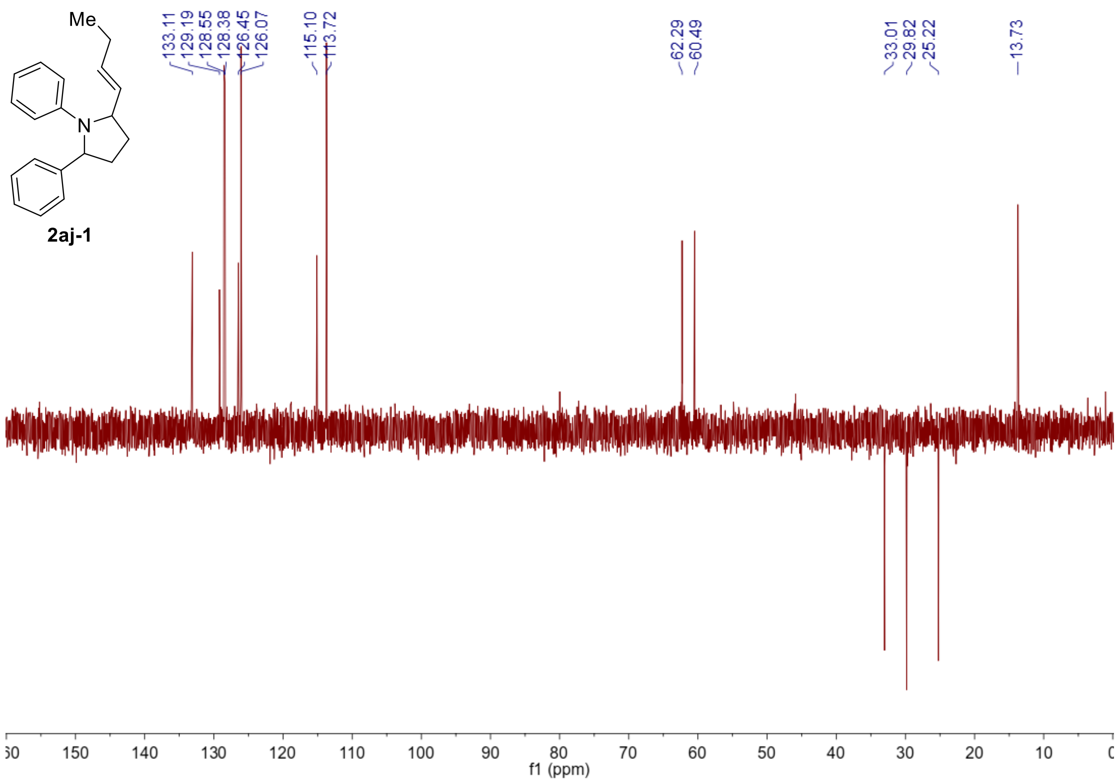
Me
N
2aj-1
133.11
129.19
128.55
128.38
126.45
126.07
115.10
113.72
62.29
60.49
33.01
29.82
25.22
13.73
f1 (ppm)

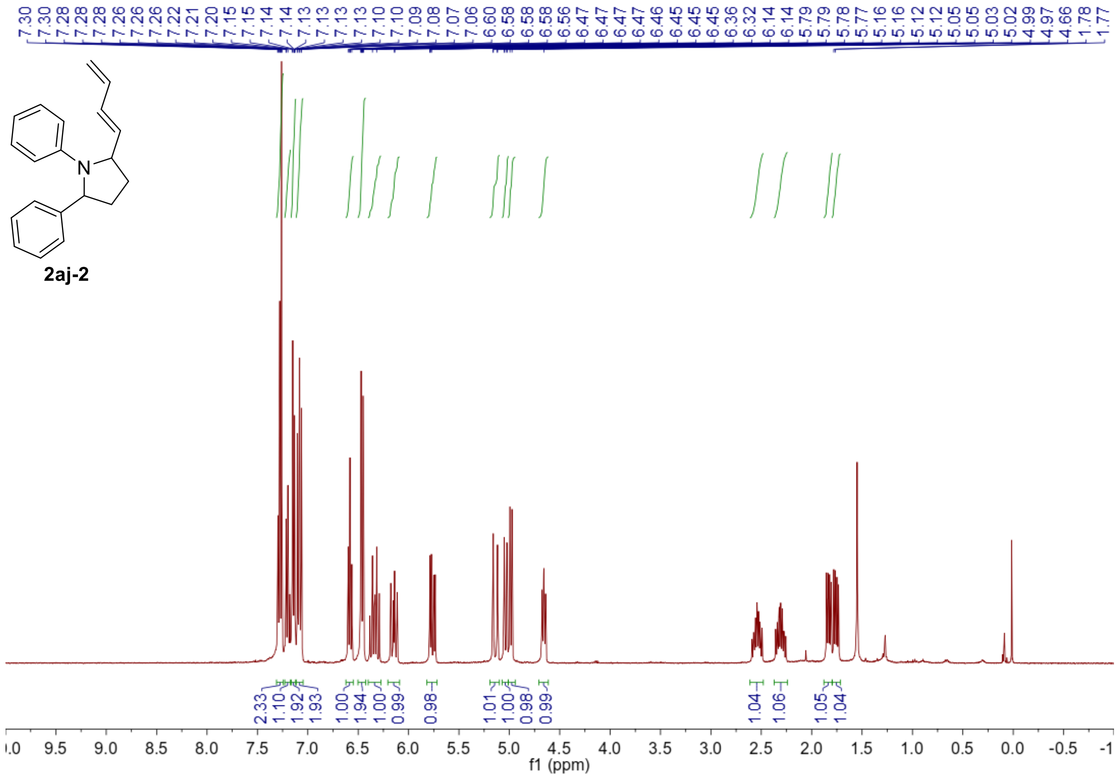
N
2aj-2
f1 (ppm)

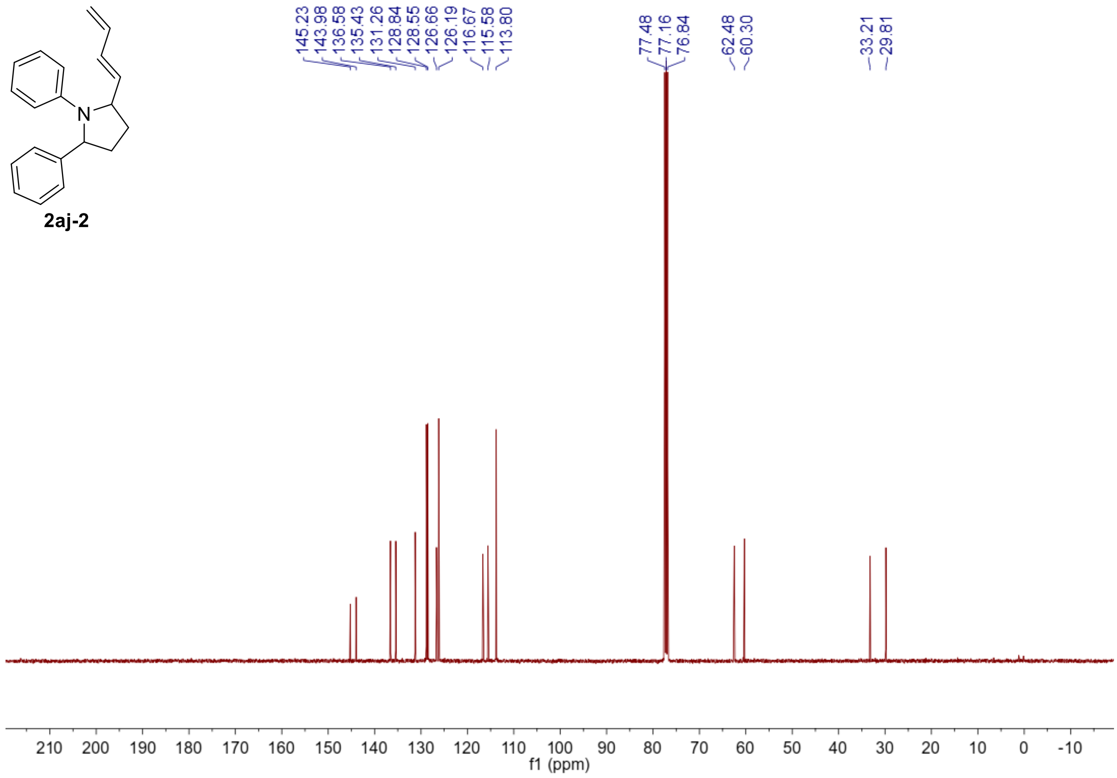
145.23
143.98
136.58
135.43
131.26
128.84
128.55
126.66
126.19
116.67
115.58
113.80
77.48
77.16
76.84
62.48
60.30
33.21
29.81
N
f1 (ppm)

2aj-2

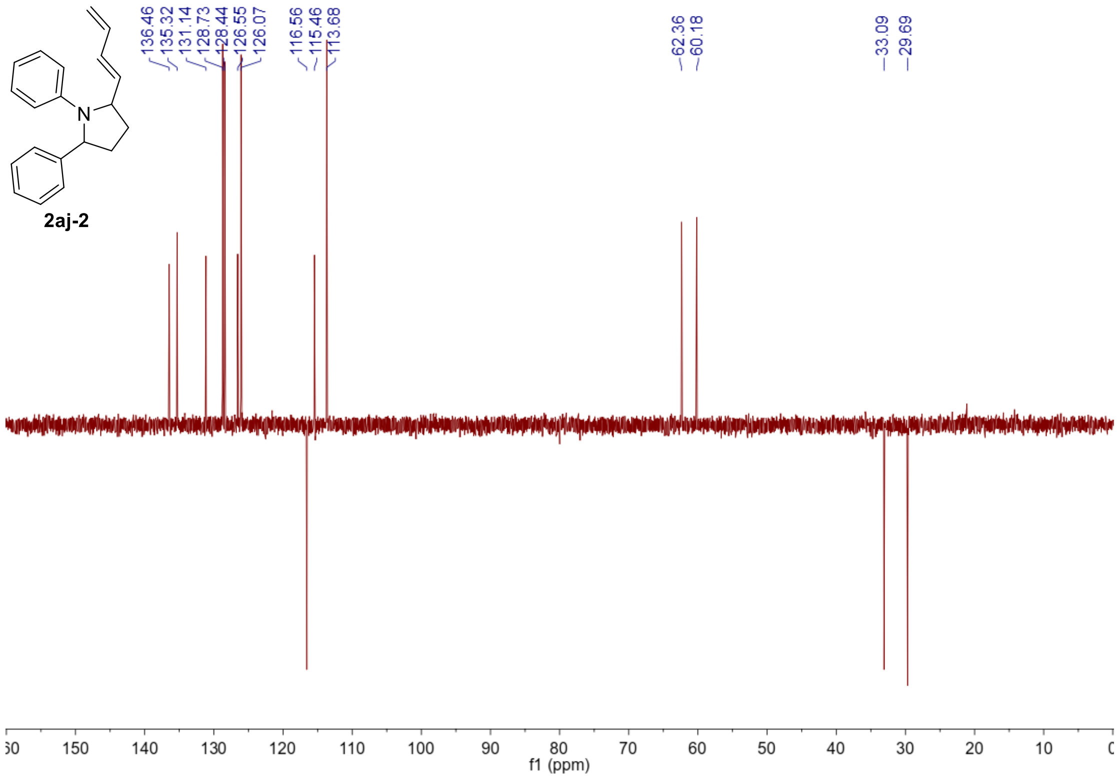
136.46
135.32
131.14
128.73
128.44
126.55
126.07
116.56
115.46
113.68
62.36
60.18
33.09
29.69
N
f1 (ppm)

2aj-2

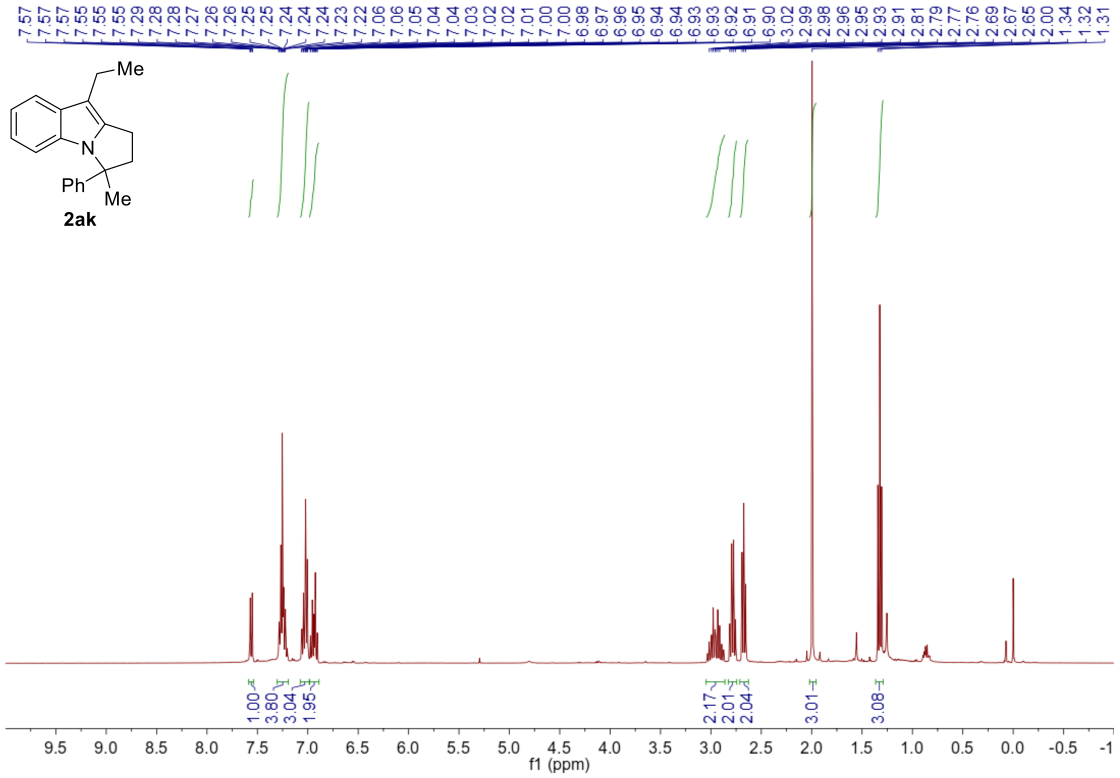
7.57
7.57
7.55
7.55
7.55
7.29
7.28
7.28
7.27
7.26
7.26
7.25
7.25
7.24
7.24
7.24
7.23
7.22
7.06
7.06
7.05
7.04
7.04
7.03
7.02
7.02
7.01
7.00
7.00
6.98
6.97
6.96
6.95
6.94
6.94
6.93
6.93
6.92
6.91
6.90
3.02
2.99
2.98
2.96
2.95
2.93
2.91
2.81
2.79
2.77
2.76
2.69
2.67
2.65
2.00
1.34
1.32
1.31
Me
N
Ph
Me
1.00
3.80
3.04
1.95
2.17
2.01
2.04
3.01
3.08
9.5 9.0 8.5 8.0 7.5 7.0 6.5 6.0 5.5 5.0 4.5 4.0 3.5 3.0 2.5 2.0 1.5 1.0 0.5 0.0 -0.5 -1
f1 (ppm)

2ak

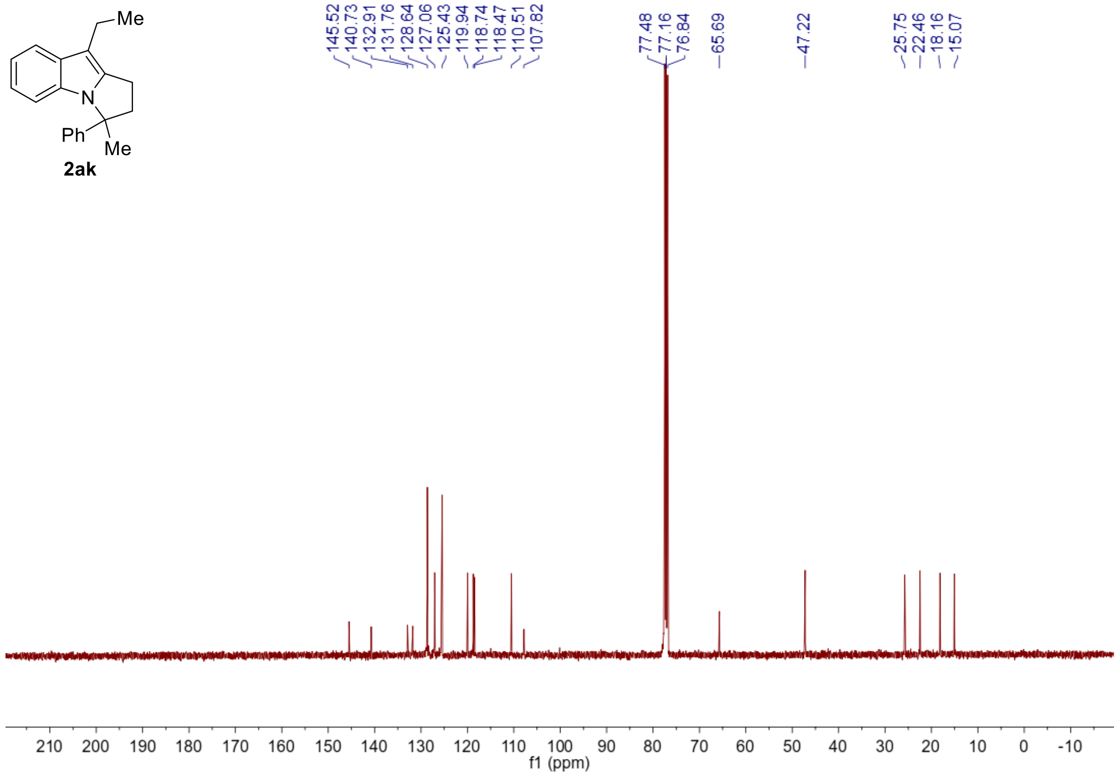
145.52
140.73
132.91
131.76
128.64
127.06
125.43
119.94
118.74
118.47
110.51
107.82
77.48
77.16
76.84
65.69
47.22
25.75
22.46
18.16
15.07
Me
N
Ph
Me
210 200 190 180 170 160 150 140 130 120 110 100 90 80 70 60 50 40 30 20 10 0 -10
f1 (ppm)

2ak

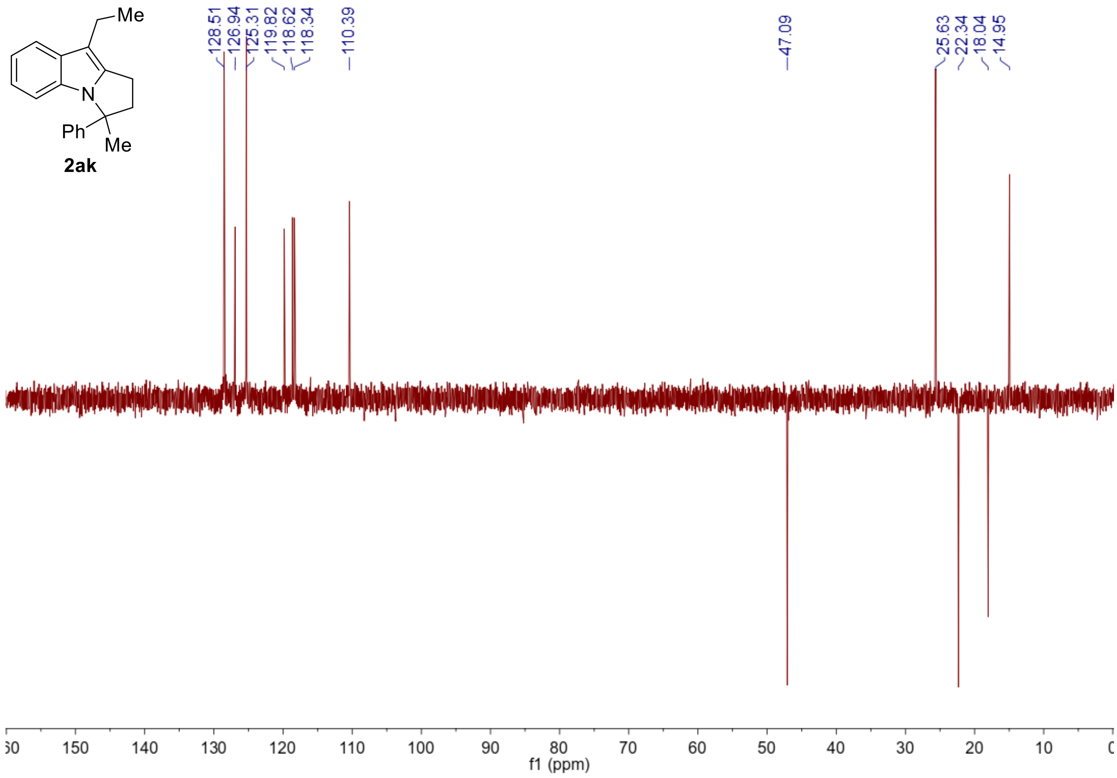
2ak
128.51
126.94
125.31
119.82
118.62
118.34
110.39
47.09
25.63
22.34
18.04
14.95
f1 (ppm)

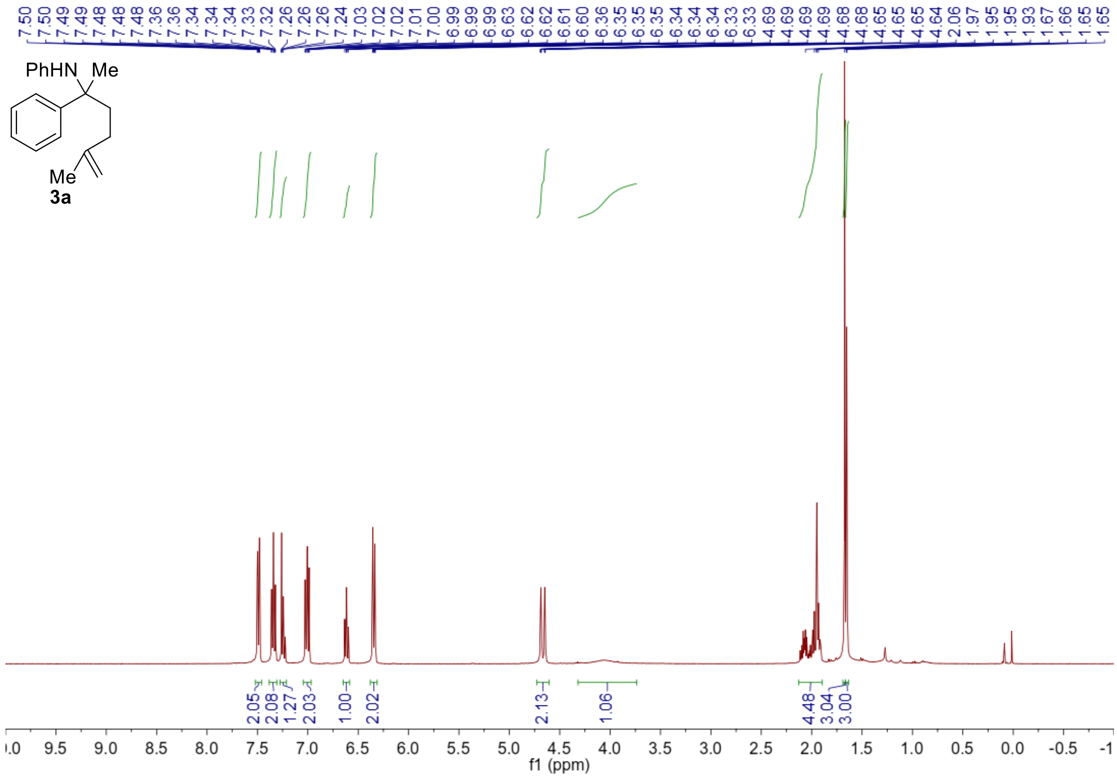
3a
2.05
2.08
1.27
2.03
1.00
2.02
2.13
1.06
4.48
3.04
3.00
f1 (ppm)

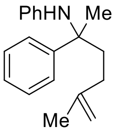
PhHN
Me
Me

3a

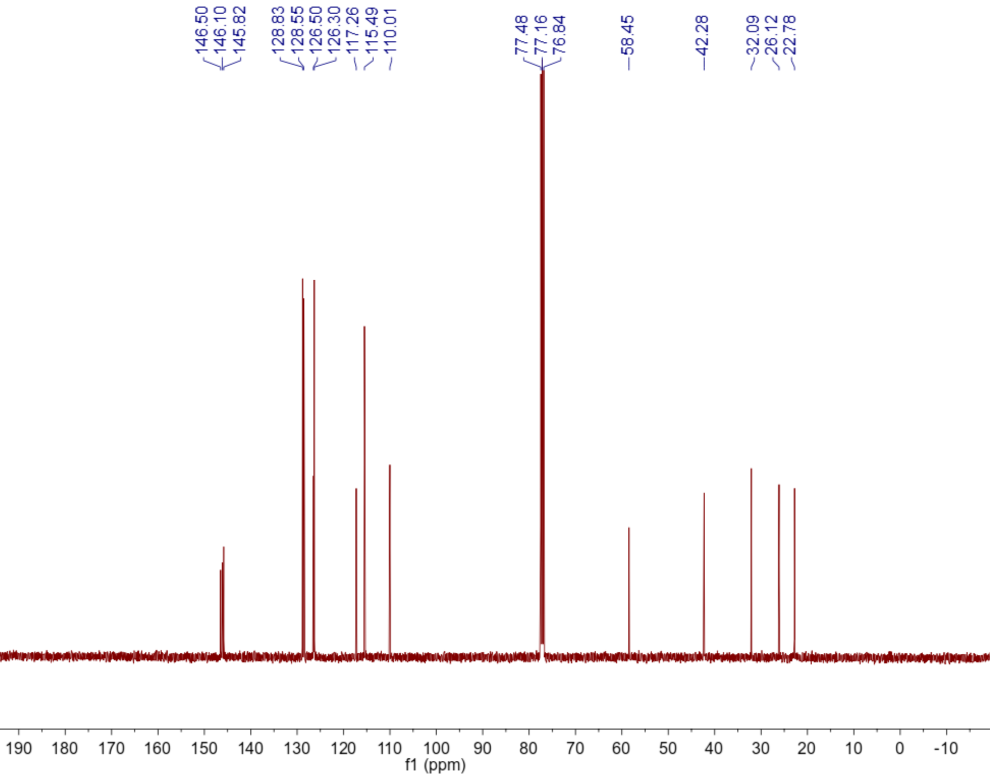
146.50
146.10
145.82
128.83
128.55
126.50
126.30
117.26
115.49
110.01
77.48
77.16
76.84
58.45
42.28
32.09
26.12
22.78
210 200 190 180 170 160 150 140 130 120 110 100 90 80 70 60 50 40 30 20 10 0 -10
f1 (ppm)
